# Regioselective Amidine-Directed Iridium-Catalyzed C(sp^3^)–H Borylation by Attuning Boronic Esters

**DOI:** 10.1021/acs.orglett.6c02012

**Published:** 2026-07-06

**Authors:** Anshu Yadav, Milton R. Smith, Robert E. Maleczka

**Affiliations:** Department of Chemistry, 3078 Michigan State University, 578 South Shaw Lane, East Lansing, Michigan 48824, United States

## Abstract

The first Ir-catalyzed C–H borylation (CHB) of amidine aliphatic bonds is reported. The products can be isolated as boronic esters as well as air stable difluoroborate molecules. Selectivity for primary as well as secondary N-adjacent C­(sp^3^)–H bonds is demonstrated even in the presence of sterically accessible C­(sp^2^)–H and other C­(sp^3^)–H bonds. Monoborylation occurs when the amidine is the limiting reagent versus the boron source.

## Graphical abstract

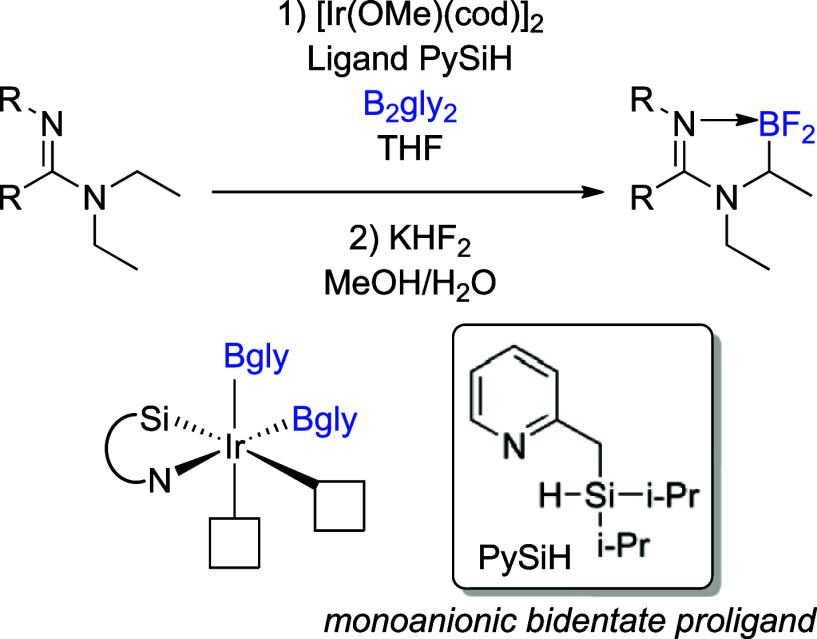

C–H bond functionalization using transition metal catalysis is among the key emergent methods to achieve site-selective functionalization, including in complex molecules with multiple C–H bonds.^1–4^ Transition metal-mediated C–H borylations using Rh,^5–8^ Pd,^9,10^ Ru,^6,11^ and Ni^12^ catalysts have been employed for sp^3^ C–H bonds. Among transition metals, iridium-based catalytic systems that enable sp^3^ C–H borylation have been applied to a variety of substrate classes.^6,7,13–35^ Early examples of sp^3^ Ir-catalyzed C–H borylation (CHB) involved sterically available methyl C–H bonds.^24^ Additives like *t*-BuOK^27^ have been utilized for CHB of sterically hindered secondary C–H bonds, along with cyclic ethers and cyclic amines.^19,22,29^ The 2-methylphenanthroline ligand developed by Hartwig has enabled undirected borylation of secondary C–H bonds in carbocycles, saturated heterocycles,^20^ and tertiary C–H bonds in the strained rings of bicyclo[1.1.1]­pentanes and bicyclo[2.1.1]­hexanes.^33,49^ The same group also reported on the successful hydrosilane-directed Ir-catalyzed CHB of benzylic bonds by silyl borane reagents in the presence of electron deficient phenanthroline ligands.^16,25,31^ Clark’s group showed that the phosphine of aryl phosphines could direct borylation of benzylic C–H bonds and enable activation of benzylic C–O and C–N bonds.^13^ Sawamura and co-workers introduced a heterogeneous silica-supported monophosphine (silica-SMAP) iridium catalyst system that selectively activated secondary C–H bonds in the presence of benzylic C–H bonds by using pyridine as a directing group.^18^ Amide-directed CHB is possible through the employment of a quinoline–silyl^30^ and pyridyl–silyl ligands.^32^ Such sp^3^ borylations could be made asymmetric by using a chiral bidentate boryl ligand (CBL).^26^ Recently, a modified CBL has been used in the CHB of masked primary alcohols,^28^ pyrazoles,^34^ aminocyclopropanes,^35^ and azacycles.^45^ To date, amidine-directed Ir-catalyzed CHBs have been absent from the chemical literature.

Amidines are found in nature and have been isolated from fungi, marine invertebrates, and plants. Fromiamycalin, a complex amidine, was isolated from *Fromia monilis*.^36–38^ Functionalized amidines are present in compounds with potential for use against intracellular parasitic protozoa^39^ and those that have shown antibiotic activity, e.g., bottromycin, a macrocyclic peptide isolated from *Streptomyces bottropensis*.^40–42^ Amidine natural products are utilized in medicinal chemistry, some notable examples of which with their respective functions are displayed in Figure 1.^43^

**Figure 1:**
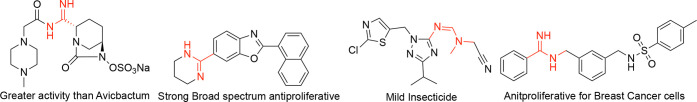
Notable amidine compounds and their respective functions.

In 2022, our group showed a highly active homogeneous iridium catalyst based on a bidentate monoanionic ligand that enabled amide-directed CHB (Scheme 1).^32^ Inspired by this work, we hypothesized amidine-directed iridium-catalyzed CHB, in which the nitrogen atom at the double bond would serve as a directing group to guide the borylation at other N-adjacent γ positions. This approach would enable the functionalization of sp^3^ carbon centers that are typically more challenging to activate owing to their relatively inert and nonpolar nature compared to their sp^2^ counterparts. Herein, we disclose the first amidine-directed C­(sp^3^)–H borylation at primary and secondary C–H bonds, where the PySiH proligand likely installs the bidentate monoanionic PySi ligand by oxidative addition.

**Scheme 1:**
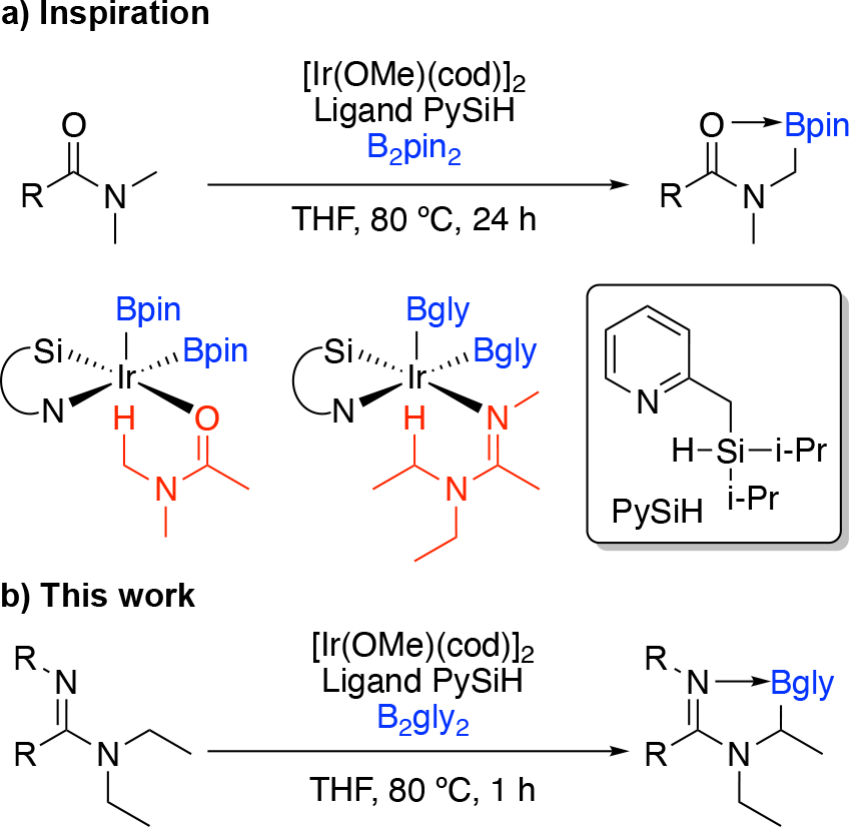
Iridium-Catalyzed CHB of Amidine

We initiated our amidine borylation experiments using (*E*)-*N*′-(*sec*-butyl)-*N,N*-dimethylacetimidamide (**1a**) with 1.5 equiv of B_2_pin_2_ (bis­(pinacolato)­diboron) in the presence of 1.5 mol % [Ir­(OMe)­cod]_2_ (cod: 1,4-cyclooctadiene) and monoanionic bidentate 3 mol % pyridyl silyl proligand (PySiH) in THF at 80 °C. Complete conversion of (*E*)-*N′*-(*sec*-butyl)-*N,N*-dimethylacetimidamide was achieved within 1 h according to ^1^H NMR in CDCl_3_.

With these conditions in hand, we explored substrate scope (Scheme 2). We first examined **1b**, which is the smallest amidine with competing methyl groups at both nitrogen atoms. CHB furnished product **2b** in an excellent yield. Then, we investigated substrates with various alkyl substituents at different positions. The CHB of **1c** afforded **2c** in 81% yield.

**Scheme 2:**
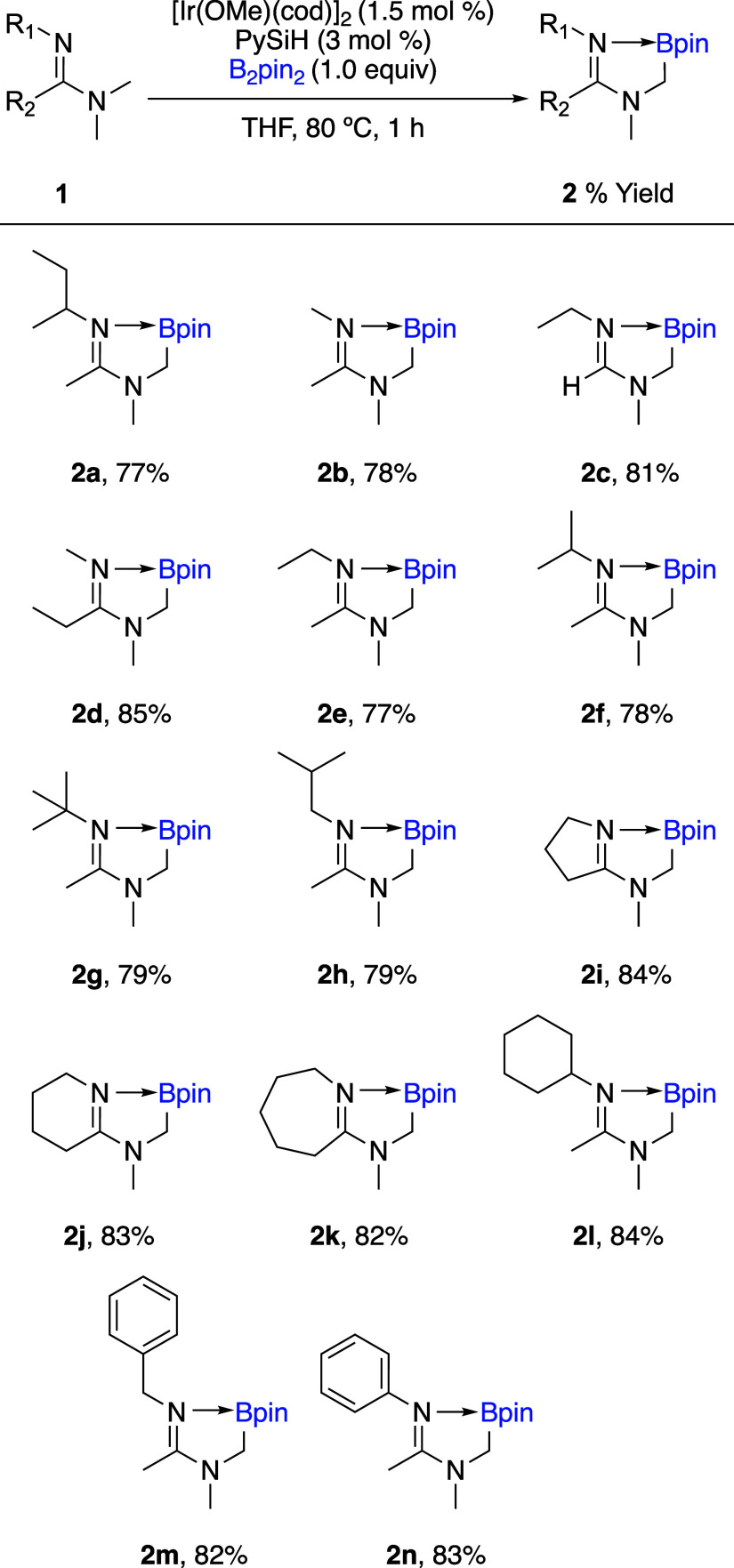
Substrate Scope of CHB on Primary C–H Bonds in Amidine^a^

a Reaction conditions: amidine (1.0 equiv, 1.0 mmol), B_2_pin_2_ (1.0 equiv, 1.0 mmol), [Ir­(OMe)­cod]_2_ (1.5 mol %, 0.015 mmol), and PySiH (3 mol %, 0.03 mmol) in 3 mL of THF for 1 h.

Interestingly, compound **1d** exhibited perfect *N*-methyl regioselectivity despite having three primary C­(sp^3^)–H bonds equidistant from the directing imine that could potentially undergo C–H activation. In an amidine resonance structure, delocalization of the sp^3^-hybridized nitrogen lone pair into the π-adjacent system forms a C=N bond with a positive charge nitrogen. This charge separation increases the polarity of the neighboring C–H bond, imparting electrophilic character to the N-adjacent C–H bond. Such highly polarized C–H bonds are more susceptible to oxidative addition with metal complexes, decreasing the activation energy required for C–H bond cleavage relative to less polar C–H bonds, resulting in exclusive regioselectivity to the N-adjacent C–H site. Increasing the steric hindrance with groups ranging from ethyl to isopropyl to *tert*-butyl **1e**–**1h** had no detrimental effect on the reaction. Cyclic amidines (**1i**–**1k**) with five-, six-, and seven-membered rings, respectively, were well-tolerated. Introducing cyclohexane at the imine nitrogen (**1l**) gave the product in excellent yield. Iridium-based catalysts are known to favor C­(sp^2^)–H activation over C­(sp^3^)–H bonds.^24,29,30^ This prompted us to examine (*E*)-*N,N*-dimethyl-*N*′-phenylacetimidamide (**1m**). To our surprise, exclusive regioselectivity was observed for C­(sp^3^)–H borylation and no C­(sp^2^)–H activation occurred. We attribute this to the strong σ donor nitrogen forming a coordinate bond with iridium and in turn facilitating C­(sp^3^)–H borylation. Decreasing the distance by removing the methylene linker between C­(sp^2^)–H and C­(sp^3^)–H bonds yielded perfect selectivity for C­(sp^3^)–H borylation, demonstrating this method’s tolerance of unsaturated C–H bonds.

Next, we further evaluated the feasibility of secondary C–H bonds. Applying our optimization reaction conditions to (*E*)-*N,N*-diethyl-*N′*-methylacetimidamide (**3a**) with 1.0 equiv of B_2_pin_2_ resulted in a complex mixture (Scheme 3). Historically, efforts to achieve high selectivity in CHB reactions have primarily concentrated on thorough ligand screening, with the role of the chosen diboron reagent being a less investigated aspect. Since the pyridyl silyl ligand showed a strong preference for amidine CHB at primary positions, we decided to vary the diboron source. One of the most used boron sources in iridium-catalyzed CHB, HBpin, also gave poor results. Increasing the size of the glycolate chain from five to six members (B_2_mbg_2_) was of no avail. Here, steric hindrance arising from the amidine methyl group with methyls of B_2_pin_2_, HBpin, and bulky B_2_mbg_2_ likely reduced reactivity, promoting the formation of a complex mixture. Removing three glycolate alkyl substituents (B_2_bg_2_) provided steric accessibility to the C–H site, resulting in a 15% conversion of the starting material. Substituting the Et group in B_2_bg_2_ with Me or H resulted in complete conversion of the amidine. Previous hydroboration studies have demonstrated enhanced reactivity with B_2_eg_2_ relative to other diboron reagents.^46–48^ Thus, it is also possible that the Lewis acidity of B_2_eg_2_, being highest among the diboron reagents examined, may have suppressed undesired side reactions while accelerating the C–H activation.

**Scheme 3:**
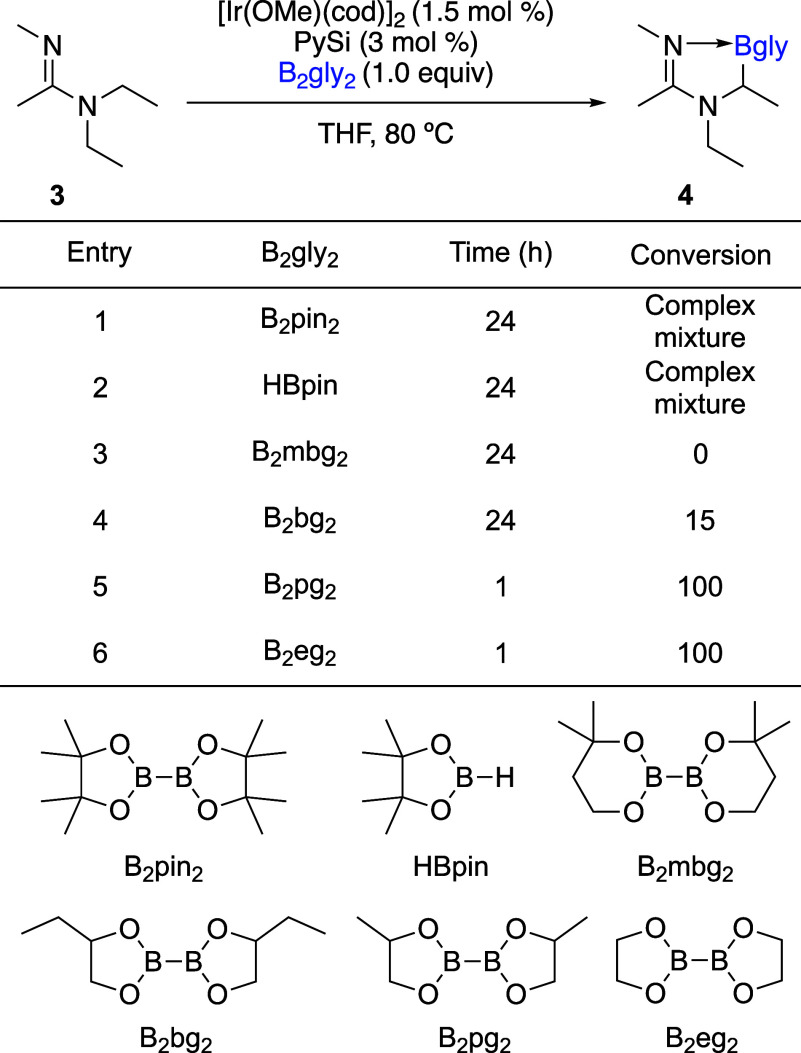
Optimization of the Reaction Conditions for CHB of Amidine on Secondary C–H Bonds^a^

a Reaction conditions: amidine (1.0 equiv, 1.0 mmol), B_2_gly_2_ (1.0 equiv, 1.0 mmol), [Ir­(OMe)­cod]_2_ (1.5 mol %, 0.015 mmol), and PySiH (3 mol %, 0.03 mmol) in 3 mL of THF. Conversion of the starting material was based on the ^1^H NMR spectrum of the crude product.

We then investigated the substrate scope with B_2_eg_2_ (Scheme 4). CHB of **3a** gave an excellent yield. Exclusive regioselectivity was observed at secondary C–H bonds in **3b**, where primary and secondary positions are available equidistant from the directing group. Competing secondary positions at both nitrogen atoms in **3c** and **3d** gave the corresponding products in excellent yields. Reactivity at the *N*-ethyl position was observed in **3e**. Cyclic amidines (**3f**–**3h**) with five-, six-, and seven-membered rings, respectively, were also tolerated. Amidines **3e** and **3i** showed no adverse effects when the substituent was elongated in the form of a ring. Amidine **3i** is also notable for its morpholine moiety.

**Scheme 4:**
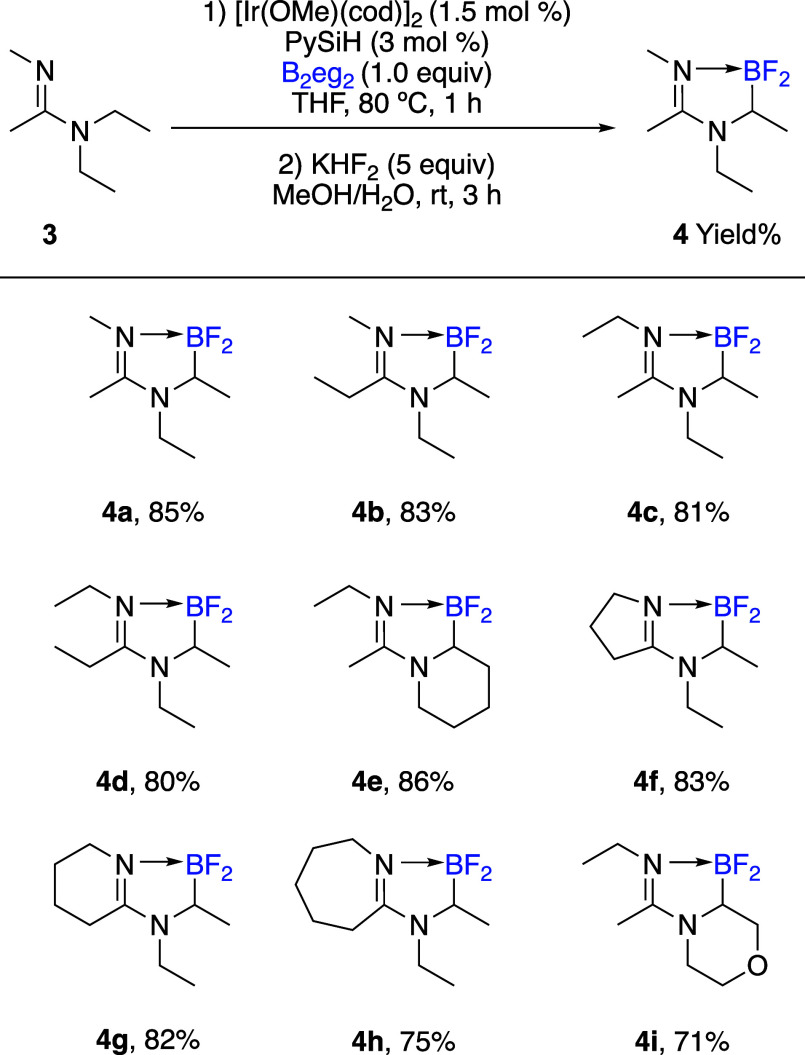
Substrate Scope of CHB on Secondary C–H Bonds in Amidine^a^

a Reaction conditions: amidine (1.0 equiv, 1.0 mmol), B_2_eg_2_ (1.0 equiv, 1.0 mmol), [Ir­(OMe)­cod]_2_ (1.5 mol %, 0.015 mmol), and PySiH (3 mol %, 0.03 mmol) in 3 mL of THF for 1 h. KHF_2_ (5.0 equiv, 5.0 mmol) in a 1:1 MeOH/H_2_O mixture in 20 mL for 3 h.

A noteworthy aspect of these borylations was the challenge encountered during isolation. ^1^H NMR of the crude reaction mixture indicated 100% conversion of the starting material. However, no borylated amidines were recovered following silica gel column chromatography. Switching to neutral alumina and attempts to isolate by sublimation went in vain. As the decomposition of Beg CHB products on silica gel is well documented,^44^ we sought to form compounds **4a**–**4h** as their potassium trifluoro borate salts. In practice, the reaction of CHB amidine **3a** with KHF_2_ gave a mixture of the potassium trifluoroborate salt and the difluoroborate of the amidine that were challenging to separate. Difficulties were also observed during the synthesis of secondary amidine **3i**, in which tetraethyl urea was formed as a byproduct. Silica column chromatography of this mixture afforded only the tetraethyl urea. Amidine **3i** was never recovered, even when using 100% methanol as an eluent. These results suggested that the amidines were retained on silica gel because of their basic nature. Compounds **4a–4i** were isolated with basic alumina gel column chromatography, yielding the difluoroborates as a single CHB amidine product.^45^ Accordingly, purification was carried out using basic alumina for **2a**–**2n**, as well. We were able to convert primary amidine **2b** into the difluoroborate compound, as well (Scheme 5).

**Scheme 5:**
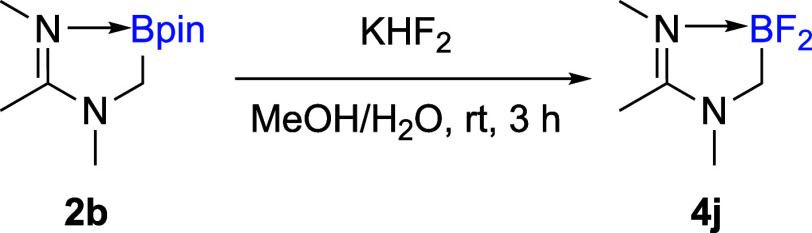
Conversion of the Boronic Ester into a BF_2_ Compound

In conclusion, we have demonstrated the first successful C­(sp^3^)–H borylation of amidines at both primary and secondary C–H bonds. The study establishes the effectiveness of nitrogen atoms in the amidine double bond as a directing group for borylation at N-adjacent positions. Excellent regioselectivity was observed across various alkyl substituents and cyclic structures. The chemistry showcased remarkable tolerance for different functional groups and preferential C­(sp^3^)–H activation over C­(sp^2^)–H bonds. The investigation of secondary C–H bonds revealed the critical role of diboron reagent choice in achieving the desired reactivity. The research also highlighted the challenges in isolating the borylated amidines, leading to innovative approaches such as conversion to difluoroborates and the use of basic alumina gel for chromatography. The findings pave the way for further exploration of amidine-directed borylations and open new avenues for selective C–H activation strategies.

## Supplementary Material



## Data Availability

The data underlying this study are available in the published article and its Supporting Information.

## Notes

Experimental procedures, including preparation of starting materials and compound characterization data (PDF)

## Abbreviations

CHB: Ir-catalyzed CH borylation
eg: ethane-1,2-diol
pg: propane-1,2-diol
bg: butane-1,2-diol
mbg: 3-methylbutane-1,3-diol
