# Supplementary material for: Regioselective Amidine-Directed Iridium-Catalyzed C(sp3)–H Borylation by Attuning Boronic Esters
Source: Org Lett. 2026 Jul 6;28(28):8783–7. doi: 10.1021/acs.orglett.6c02012 (PMC13386528; doi:10.1021/acs.orglett.6c02012)

Supporting Information

Regioselective amidine directed iridium catalyzed $C(sp^3)$–H borylation by attuning boronic esters

Anshu Yadav, Milton R. Smith, III and Robert E. Maleczka, Jr.*

Department of Chemistry, Michigan State University, 578 South Shaw Lane, East Lansing, Michigan 48824-1322 USA

Email: maleczka@chemistry.msu.edu

Table of Contents

General Information S4
Preparation of Ligand PySi S5
Synthesis of (E)-N'-(sec-butyl)-N,N-dimethylacetimidamide (1a) S5
Synthesis of (E)-N,N,N'-trimethylacetimidamide (1b) S6
Synthesis of (E)-N'-ethyl-N,N-dimethylformimidamide (1c) S6
Synthesis of (E)-N,N,N'-trimethylpropionimidamide (1d) S7
Synthesis of (E)-N'-ethyl-N,N-dimethylacetimidamide (1e) S8
Synthesis of (E)-N'-isopropyl-N,N-dimethylacetimidamide (1f) S9
Synthesis of (E)-N'-(tert-butyl)-N,N-dimethylacetimidamide (1g) S9
Synthesis of (E)-N'-isobutyl-N,N-dimethylacetimidamide (1h) S10
Synthesis of N,N-dimethyl-3,4-dihydro-2H-pyrrol-5-amine (1i) S11
Synthesis of N,N-dimethyl-3,4,5,6-tetrahydro-2H-azepin-7-amine (1k) S12
Synthesis of (E)-N'-cyclohexyl-N,N-dimethylacetimidamide (1l) S13
Synthesis of (E)-N'-benzyl-N,N-dimethylacetimidamide (1m) S14
Synthesis of (E)-N,N-dimethyl-N'-phenylacetimidamide (1n) S14
CHB of (E)-N'-(sec-butyl)-N,N-dimethylacetimidamide (2a) S15
CHB of (E)-N,N,N'-trimethylacetimidamide (2b) S16
CHB of (E)-N'-ethyl-N,N-dimethylformimidamide (2c) S16
CHB of (E)-N,N,N'-trimethylpropionimidamide (2d) S17
CHB of (E)-N'-ethyl-N,N-dimethylacetimidamide (2e) S18
CHB of (E)-N'-isopropyl-N,N-dimethylacetimidamide (2f) S19
CHB of (E)-N'-(tert-butyl)-N,N-dimethylacetimidamide (2g) S19
CHB of N,N-dimethyl-3,4-dihydro-2H-pyrrol-5-amine (2i) S21
CHB of N,N-dimethyl-3,4,5,6-tetrahydropyridin-2-amine (2j) S22
CHB of N,N-dimethyl-3,4,5,6-tetrahydro-2H-azepin-7-amine (2k) S22
CHB of (E)-N'-cyclohexyl-N,N-dimethylacetimidamide (2l) S23
CHB of (E)-N'-benzyl-N,N-dimethylacetimidamide (2m) S24
CHB of (E)-N,N-dimethyl-N'-phenylacetimidamide (2n) S25
Synthesis of (E)-N,N-diethyl-N'-methylacetimidamide (3a) S25
Synthesis of (E)-N,N-diethyl-N'-methylpropionimidamide (3b) S26
Synthesis of (E)-N,N,N'-triethylacetimidamide (3c) S27
Synthesis of (E)-N,N,N'-triethylacetimidamide (3d) S28
Synthesis of (E)-N-ethyl-1-(piperidin-1-yl)ethan-1-imine (3e) S29
Synthesis of N,N-diethyl-3,4-dihydro-2H-pyrrol-5-amine (3f) S29
Synthesis of N,N-diethyl-3,4,5,6-tetrahydropyridin-2-amine (3g) S30
Synthesis of N,N-diethyl-3,4,5,6-tetrahydropyridin-2-amine (3h) S31
Synthesis of B_2eg_2 (2,2'-bi(1,3,2-dioxaborolane)) S32
Synthesis of B_2pg_2 (4,4'-dimethyl-2,2'-bi(1,3,2-dioxaborolane)) S33
Synthesis of B_2bg_2 (4,4'-diethyl-2,2'-bi(1,3,2-dioxaborolane)) S33
Synthesis of B_2mbg_2 (4,4,4',4'-tetramethyl-2,2'-bi(1,3,2-dioxaborinane)) S34
Optimization of Reaction Conditions S35
CHB of (E)-N,N-diethyl-N'-methylacetimidamide with B_2pin_2 ..... S35
CHB of (E)-N,N-diethyl-N'-methylacetimidamide with HBpin S35
CHB of (E)-N,N-diethyl-N'-methylacetimidamide with B_2mbg_2 ..... S36
CHB of (E)-N,N-diethyl-N'-methylacetimidamide with B_2bg_2 ..... S36

CHB of (E)-N,N-diethyl-N’-methylacetimidamide with B_2pg_2 .... S37
CHB of (E)-N,N-diethyl-N’-methylacetimidamide with B_2eg_2 .... S37
CHB of (E)-N,N-diethyl-N'-methylacetimidamide (4a) S38
CHB of (E)-N,N-diethyl-N'-methylpropionimidamide (4b) S39
CHB of (E)-N,N,N'-triethylacetimidamide (4c) S40
CHB of (E)-N,N,N'-triethylpropionimidamide (4d) S41
CHB of (E)-N-ethyl-1-(piperidin-1-yl)ethan-1-imine (4e) S42
CHB of N,N-diethyl-3,4-dihydro-2H-pyrrol-5-amine (4f) S43
CHB of N,N-diethyl-3,4,5,6-tetrahydropyridin-2-amine (4g) S44
CHB of N,N-diethyl-3,4,5,6-tetrahydro-2H-azepin-7-amine (4h) S45
CHB of (E)-N-ethyl-1-morpholinoethan-1-imine (4i) S46
Synthesis of (E)-N-((difluoroboraneyl)methyl)-N,N'-dimethylacetimidamide (4j) S47
REFERENCES S48
NMR SPECTRUM S50

General Information

All commercially available chemicals were used as received unless otherwise indicated. Bis(pinacolato)diboron (B_2pin_2) was generously supplied by BoroPharm, Inc. Bis(η^4-1,5-cyclooctadiene)-di-μ-methoxy-diiridium(I) $[Ir(cod)(OMe)]_2$ was made by a literature procedure[1] or purchased from Sigma-Aldrich. Tetrahydrofuran (THF) was refluxed over sodium/benzo phenone ketyl, distilled and degassed before use.

Column chromatography was performed on 240–400 mesh Silica P-Flash silica gel. Thin layer chromatography was performed on 0.25 mm thick aluminum–backed silica gel plates and visualized with ultraviolet light (λ = 254 nm) and alizarin stain to visualize boronic esters. Sublimations were conducted with a water-cooled cold finger.

^{1}H, ^{13}C, ^{11}B and ^{19}F NMR spectra were recorded on a Varian 500 MHz DD2 Spectrometer equipped with a ^{1}H-^{19}F/^{15}N-^{31}P 5 mm Pulsed Field Gradient (PFG) Probe, or an Innova 300 MHz spectrometer equipped with a QUAD (^{1}H/^{19}F and ^{11}B) PFG probe. Spectra were taken in $CDCl_3$ referenced to 7.26 ppm in ^{1}H NMR and 77.2 ppm in ^{13}C NMR. Resonances for the boron-bearing carbon atom were not observed due to quadrupolar relaxation. All coupling constants are apparent J values measured at the indicated field strengths in Hertz (s = singlet, d = doublet, t = triplet, q = quartet, dd = doublet of doublets, ddd = doublet of doublet of doublets, bs = broad singlet). NMR spectra were processed for display using the MNova software program with only phasing and baseline corrections applied.

High-resolution mass spectra (HRMS) were obtained at the Mass spectrometry analysis was performed at the Molecular Metabolism and Disease Mass Spectrometry Core facility at Michigan State University using electrospray ionization (ESI+ or ESI-) on quadrupole time-of-flight (Q-

TOF) instruments. Melting points were measured in a capillary melting point apparatus and are uncorrected.

Preparation of Ligand PySi

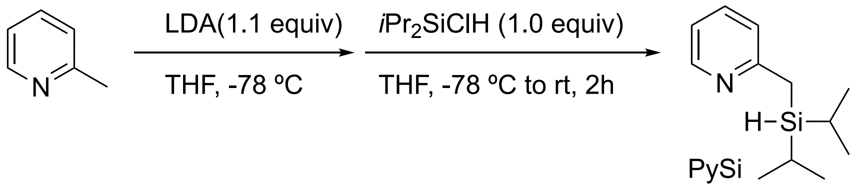

Ligand PySiH was prepared in similar yield following the previously reported procedure.[2]

Synthesis of (E)-N'-(sec-butyl)-N,N-dimethylacetimidamide (1a)

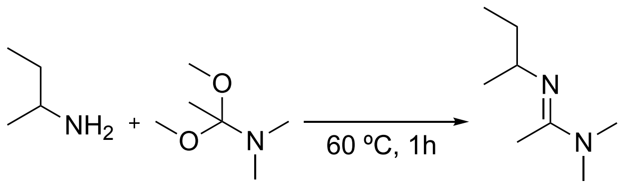

In a nitrogen filled glove box, a 100 mL oven dried round bottom flask equipped with a stir bar was charged with 1,1-dimethoxy-*N*,*N*-dimethylethan-1-amine (3.6 mL, 0.025 mol, 1 equiv) and butan-2-amine (2.5 mL, 0.025 mol, 1 equiv). The round bottom flask was sealed, taken out of the glove box and placed into a pre-heated oil bath at 60 °C. After stirring for 1 h, the mixture was concentrated under reduced pressure and purified by vacuum distillation at 55 ºC at 15 torr pressure. The fractions containing product were collected to yield 2.3 g of **1a** as a colorless liquid (67% yield).

^{1}H NMR (500 MHz, $CDCl_3$): δ 3.15 (m, 1H), 2.84 (s, 6H), 1.85 (s, 3H), 1.40 (m, 2H), 1.01 (d, J = 6.2 Hz, 3H), 0.81 (t, J = 7.4 Hz, 3H). ^{13}C NMR{^{1}H} (126 MHz, $CDCl_3$): δ 156.9, 55.4, 38.2, 32.4, 22.8, 12.6, 11.4. HRMS (ESI) *m/z* calc for $C_8H_{19}N_2$ $[M+H]^+$ 143.1548, found 143.1540.

Synthesis of (*E*)-*N*,*N*,*N*'-trimethylacetimidamide (1b)

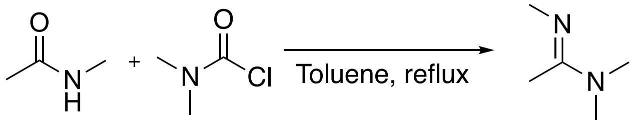

1b, 89% yield

In a 100 mL oven dried round bottom flask equipped with a stir bar was charged with *N*-methylacetamide (9.1 mL, 0.125 mol, 1 equiv), Dimethylcarbamic chloride (5.93 mL, 0.125 mol, 1 equiv) in toluene (2.6 mL) was added dropwise over 1 h period with stirring. The flask was placed in an oil bath which was heated to allow for the contents to reflux. After 6 h, the mixture was concentrated under reduced pressure and then on high vacuum. The residue was covered with a layer of ether (15 mL). While cooling in ice, potassium carbonate (2.6 g) was added and then a solution of sodium hydroxide (2.6 g in 15 mL water) was added dropwise. The ether phase was separated and the aqueous phase was extracted three times with ether. The organics were combined and dried over magnesium sulfate. After filtration and concentration, the residue was purified by vacuum distillation at 139 ºC at 760 torr pressure. The fractions containing product were collected to yield 11.1 g of **1b** as a colorless liquid (89% yield). NMR spectra of the product matched previously reported data.[3]

^{1}H NMR (500 MHz, $CDCl_3$): δ 2.96 (s, 3H), 2.84 (s, 6H), 1.86 (s, 3H). ^{13}C NMR{^{1}H} (126 MHz, $CDCl_3$): δ 161.0, 38.2, 37.2, 12.4. HRMS (ESI) *m/z* calc for $C_5H_{13}N_2$ $[M+H]^+$ 101.1079, found 101.1073.

Synthesis of (*E*)-*N*'-ethyl-*N*,*N*-dimethylformimidamide (1c)

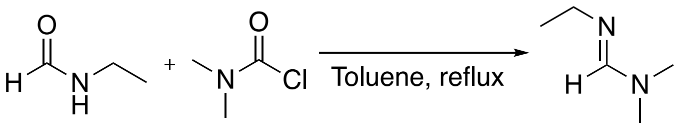

1c, 85% yield

In a 100 mL oven dried round bottom flask equipped with a stir bar was charged with *N*-ethylformamide (4.7 mL, 0.065 mol, 1 equiv), Dimethylcarbamic chloride (5.93 mL, 0.065 mol, 1 equiv) in toluene (2.6 mL) was added dropwise over 1 h period with stirring. The flask was placed in an oil bath which was heated to allow for the contents to reflux. After 6 h, the mixture was concentrated under reduced pressure and then on high vacuum. The residue was covered with a layer of ether (15 mL). While cooling in ice, potassium carbonate (2.6 g) was added and then a solution of sodium hydroxide (2.6 g in 15 mL water) was added dropwise. The ether phase was separated and the aqueous phase was extracted three times with ether. The organics were combined and dried over magnesium sulfate. After filtration and concentration, the residue was purified by vacuum distillation at 57 ºC at 6 torr pressure. The fractions containing product were collected to yield 5.5 g of **1c** as a colorless liquid (85% yield). Title compound has been reported before but no spectroscopic data was provided.[4]

^{1}H NMR (500 MHz, $CDCl_3$): δ 7.27 (s, 1H), 3.24 (q, J = 7.2 Hz, 2H), 2.81 (s, 6H), 1.11 (t, J = 7.2 Hz, 3H). ^{13}C NMR{^{1}H} (126 MHz, $CDCl_3$): δ 154.8, 50.5, 38.7, 18.4. HRMS (ESI) *m/z* calc for $C_5H_{13}N_2$ $[M+H]^+$ 101.1079, found 101.1071.

Synthesis of (*E*)-*N*,*N*,*N'*-trimethylpropionimidamide (1d)

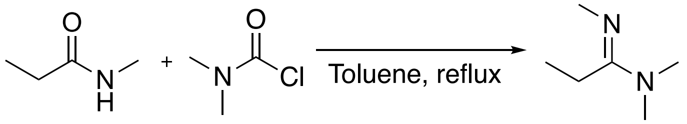

1d, 67% yield

In a 100 mL oven dried round bottom flask equipped with a stir bar was charged with *N*-methylpropionamide (5.6 mL, 0.065 mol, 1 equiv), Dimethylcarbamic chloride (5.93 mL, 0.065 mol, 1 equiv) in toluene (2.6 mL) was added dropwise over 1 h period with stirring. The flask was placed in an oil bath which was heated to allow for the contents to reflux. After 6 h, the mixture was concentrated under reduced pressure and then on high vacuum. The residue was covered with

a layer of ether (15 mL). While cooling in ice, potassium carbonate (2.6 g) was added and then a solution of sodium hydroxide (2.6 g in 15 mL water) was added dropwise. The ether phase was separated and the aqueous phase was extracted three times with ether. The organics were combined and dried over magnesium sulfate. After filtration and concentration, the residue was purified by vacuum distillation at 56 ºC at 6 torr pressure. The fractions containing product were collected to yield 4.96 g of **1d** as a colorless liquid (67% yield). Title compound has been reported before but no spectroscopic data was provided.[5]

^{1}H NMR (500 MHz, $CDCl_3$): δ 3.03 (s, 3H), 2.87 (s, 6H), 2.37 (q, *J* = 7.7 Hz, 2H), 1.07 (t, *J* = 7.7 Hz, 3H). ^{13}C NMR{^{1}H} (126 MHz, $CDCl_3$): δ 165.1, 38.1, 36.3, 19.0, 10.8. HRMS (ESI) *m/z* calc for $C_6H_{15}N_2$ $[M+H]^+$ 115.1235, found 115.1227.

Synthesis of (*E*)-*N*'-ethyl-*N*,*N*-dimethylacetimidamide (1e)

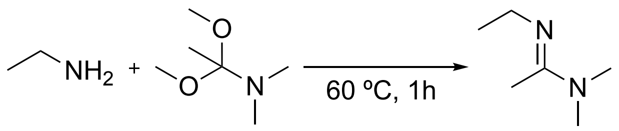

1e, 52% yield

In a nitrogen filled glove box, a 100 mL oven dried round bottom flask equipped with a stir bar was charged with 1,1-dimethoxy-*N*,*N*-dimethylethan-1-amine (3.6 mL, 0.025 mol, 1 equiv) and 2M solution of ethanamine (13.6 mL, 0.027 mol, 1.1 equiv) in methanol. The round bottom flask was sealed, taken out of the glove box and placed into a pre-heated oil bath at 60 °C. After stirring for 1 h, the mixture was concentrated under reduced pressure and purified by vacuum distillation at 58 ºC at 6 torr pressure. The fractions containing product were collected to yield 1.5 g of **1e** as a colorless liquid (52% yield). Title compound has been reported before but no spectroscopic data was provided.[6]

^{1}H NMR (500 MHz, $CDCl_3$): δ 3.23 (q, J = 7.2 Hz, 2H), 2.87 (s, 6H), 1.88 (s, 3H), 1.11 (t, J = 7.3 Hz, 3H). ^{13}C NMR{^{1}H} (126 MHz, $CDCl_3$): δ 159.2, 44.1, 38.1, 17.4, 12.4. HRMS (ESI) *m/z* calc for $C_6H_{15}N_2$ $[M+H]^+$ 115.1235, found 115.1226.

Synthesis of (*E*)-*N*'-isopropyl-*N*,*N*-dimethylacetimidamide (1f)

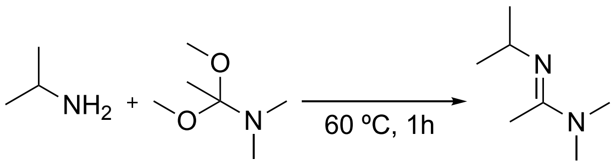

1f, 35% yield

In a nitrogen filled glove box, a 100 mL oven dried round bottom flask equipped with a stir bar was charged with 1,1-dimethoxy-*N*,*N*-dimethylethan-1-amine (3.6 mL, 0.025 mol, 1 equiv) and propan-2-amine (2.1 mL, 0.025 mol, 1 equiv). The round bottom flask was sealed, taken out of the glove box and placed into a pre-heated oil bath at 60 °C. After stirring for 1 h, the mixture was concentrated under reduced pressure and purified by vacuum distillation at 120 ºC at 760 torr pressure. The fractions containing product were collected to yield 1.1 g of **1f** as a colorless liquid (35% yield) that matched previously reported data.[7]

^{1}H NMR (500 MHz, $CDCl_3$): δ 3.46 (hept, J = 6.2 Hz, 1H), 2.85 (s, 6H), 1.87 (s, 3H), 1.06 (d, J = 6.3 Hz, 6H). ^{13}C NMR{^{1}H} (126 MHz, $CDCl_3$): δ 156.8, 49.1, 38.1, 25.3, 12.5. HRMS (ESI) *m/z* calc for $C_7H_{17}N_2$ $[M+H]^+$ 129.1392, found 129.1382.

Synthesis of (*E*)-*N*'-(*tert*-butyl)-*N*,*N*-dimethylacetimidamide (1g)

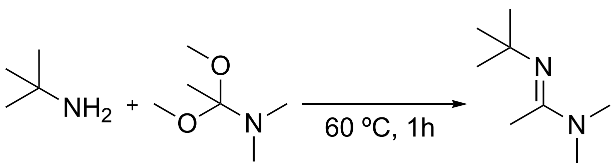

1g, 56% yield

In a nitrogen filled glove box, a 100 mL oven dried round bottom flask equipped with a stir bar was charged with 1,1-dimethoxy-*N*,*N*-dimethylethan-1-amine (3.6 mL, 0.025 mol, 1 equiv) and 2-

methylpropan-2-amine (2.9 mL, 0.025 mol, 1 equiv). The round bottom flask was sealed, taken out of the glove box and placed into a pre-heated oil bath at 60 °C. After stirring for 1 h, the mixture was concentrated under reduced pressure and purified by vacuum distillation at 60 ºC at 19 torr pressure. The fractions containing product were collected to yield 2.0 g of **1g** as a colorless liquid (56% yield). Title compound has been reported before but no spectroscopic data was provided.[6]

^{1}H NMR (500 MHz, $CDCl_3$): δ 2.82 (s, 6H), 1.97 (s, 3H), 1.23 (s, 9H). ^{13}C NMR{^{1}H} (126 MHz, $CDCl_3$): δ 155.9, 51.8, 38.1, 31.9, 16.6. HRMS (ESI) *m/z* calc for $C_8H_{19}N_2$ $[M+H]^+$ 143.1548, found 143.1539.

Synthesis of (*E*)-*N'*-isobutyl-*N*,*N*-dimethylacetimidamide (1h)

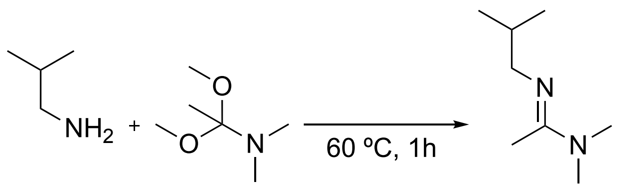

1h, 79% yield

In a nitrogen filled glove box, a 100 mL oven dried round bottom flask equipped with a stir bar was charged with 1,1-dimethoxy-*N*,*N*-dimethylethan-1-amine (3.6 mL, 0.025 mol, 1 equiv) and 2-methylpropan-1-amine (2.5 mL, 0.025 mol, 1 equiv). The round bottom flask was sealed, taken out of the glove box and placed into a pre-heated oil bath at 60 °C. After stirring for 1 h, the mixture was concentrated under reduced pressure and purified by vacuum distillation at 62 ºC at 40 torr pressure. The fractions containing product were collected to yield 2.8 g of **1h** as a colorless liquid (79% yield) that matched previously reported data.[7]

^{1}H NMR (500 MHz, $CDCl_3$): δ 2.97 (d, J = 6.9 Hz, 2H), 2.86 (s, 6H), 1.85 (s, 3H), 1.73 (m, 1H), 0.90 (d, J = 6.6 Hz, 6H). ^{13}C NMR{^{1}H} (126 MHz, $CDCl_3$): δ 158.5, 58.3, 38.1, 31.0, 20.8, 12.7. HRMS (ESI) *m/z* calc for $C_8H_{19}N_2$ $[M+H]^+$ 143.1548, found 143.1538.

Synthesis of *N,N*-dimethyl-3,4-dihydro-2*H*-pyrrol-5-amine (1i)

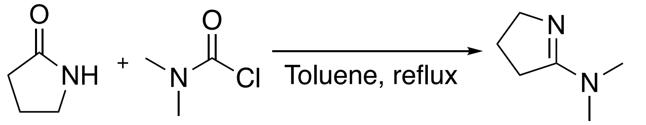

1i, 81% yield

In a 100 mL oven dried round bottom flask equipped with a stir bar was charged with pyrrolidin-2-one (4.93 mL, 0.065 mol, 1 equiv), Dimethylcarbamic chloride (5.93 mL, 0.065 mol, 1 equiv) in toluene (2.6 mL) was added dropwise over 1 h period with stirring. The flask was placed in an oil bath which was heated to allow for the contents to reflux. After 6 h, the mixture was concentrated under reduced pressure and then on high vacuum. The residue was covered with a layer of ether (15 mL). While cooling in ice, potassium carbonate (2.6 g) was added and then a solution of sodium hydroxide (2.6 g in 15 mL water) was added dropwise. The ether phase was separated and the aqueous phase was extracted three times with ether. The organics were combined and dried over magnesium sulfate. After filtration and concentration, the residue was purified by vacuum distillation at 62 ºC at 6 torr pressure. The fractions containing product were collected to yield 5.87 g of **1i** as a colorless liquid (81% yield) that matched previously reported data.[8]

^{1}H NMR (500 MHz, $CDCl_3$): δ 3.66 (t, *J* = 8.0 Hz, 2H), 2.91 (s, 6H), 2.49 (t, *J* = 8.3 Hz, 2H), 1.95 (m, 2H). ^{13}C NMR{^{1}H} (126 MHz, $CDCl_3$): δ 169.1, 56.9, 38.7, 31.8, 24.2. HRMS (ESI) *m/z* calc for $C_6H_{13}N_2$ $[M+H]^+$ 113.1079, found 113.1072.

Synthesis of *N,N*-dimethyl-3,4,5,6-tetrahydropyridin-2-amine (1j)

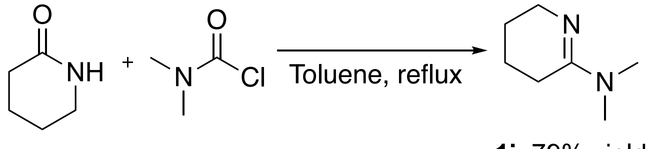

1j, 79% yield

In a 100 mL oven dried round bottom flask equipped with a stir bar was charged with piperidin-2-one (6.43 g, 0.065 mol, 1 equiv), Dimethylcarbamic chloride (5.93 mL, 0.065 mol, 1 equiv) in

toluene (2.6 mL) was added dropwise over 1 h period with stirring. The flask was placed in an oil bath which was heated to allow for the contents to reflux. After 6 h, the mixture was concentrated under reduced pressure and then on high vacuum. The residue was covered with a layer of ether (15 mL). While cooling in ice, potassium carbonate (2.6 g) was added and then a solution of sodium hydroxide (2.6 g in 15 mL water) was added dropwise. The ether phase was separated and the aqueous phase was extracted three times with ether. The organics were combined and dried over magnesium sulfate. After filtration and concentration, the residue was purified by vacuum distillation at 72 °C at 6 torr pressure. The fractions containing product were collected to yield 6.44 g of **1j** as a colorless liquid (79% yield) that matched previously reported data.[9]

^{1}H NMR (500 MHz, $CDCl_3$): δ 3.46 (t, J = 5.8 Hz, 2H), 2.80 (s, 6H), 2.18 (t, J = 6.7 Hz, 2H), 1.68 (m, 2H), 1.48 (m, 2H). ^{13}C NMR{^{1}H} (126 MHz, $CDCl_3$): δ 159.2, 47.5, 37.4, 25.2, 22.6, 20.9. HRMS (ESI) *m/z* calc for $C_7H_{15}N_2$ $[M+H]^+$ 127.1235, found 127.1239.

Synthesis of *N,N*-dimethyl-3,4,5,6-tetrahydro-2H-azepin-7-amine (1k)

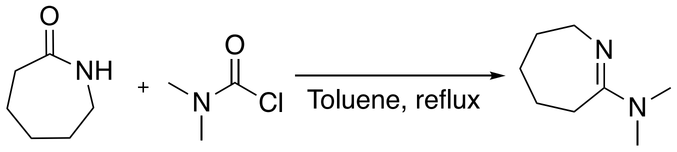

1k, 82% yield

In a 100 mL oven dried round bottom flask equipped with a stir bar was charged with azepan-2-one (7.35 g, 0.065 mol, 1 equiv), Dimethylcarbamic chloride (5.93 mL, 0.065 mol, 1 equiv) in toluene (2.6 mL) was added dropwise over 1 h period with stirring. The flask was placed in an oil bath which was heated to allow for the contents to reflux. After 6 h, the mixture was concentrated under reduced pressure and then on high vacuum. The residue was covered with a layer of ether (15 mL). While cooling in ice, potassium carbonate (2.6 g) was added and then a solution of sodium hydroxide (2.6 g in 15 mL water) was added dropwise. The ether phase was separated and

the aqueous phase was extracted three times with ether. The organics were combined and dried over magnesium sulfate. After filtration and concentration, the residue was purified by vacuum distillation at 74 ºC at 6 torr pressure. The fractions containing product were collected to yield 7.47 g of **1k** as a colorless liquid (82% yield) that matched previously reported data.[8]

^{1}H NMR (500 MHz, $CDCl_3$): δ 3.44 (m, 2H), 2.85 (s, 6H), 2.49 (m, 2H), 1.71 (m, 2H), 1.53 (m, 2H), 1.46 (m, 2H). ^{13}C NMR{^{1}H} (126 MHz, $CDCl_3$): δ 168.3, 49.7, 38.8, 31.2, 28.9, 27.5, 23.7. HRMS (ESI) *m/z* calc for $C_8H_{17}N_2$ $[M+H]^+$ 141.1392, found 141.1381.

Synthesis of (*E*)-N'-cyclohexyl-N,N-dimethylacetimidamide (1l)

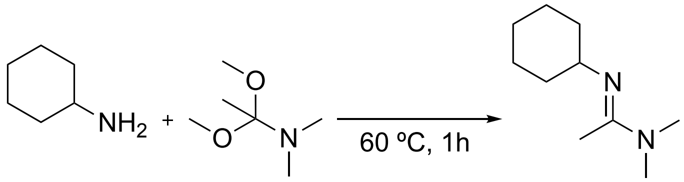

1l, 74% yield

In a nitrogen filled glove box, a 100 mL oven dried round bottom flask equipped with a stir bar was charged with 1,1-dimethoxy-*N*,*N*-dimethylethan-1-amine (3.6 mL, 0.025 mol, 1 equiv) and cyclohexanamine (3.1 mL, 0.025 mol, 1 equiv). The round bottom flask was sealed, taken out of the glove box and placed into a pre-heated oil bath at 60 ˚C. After stirring for 1 h, the mixture was concentrated under reduced pressure and purified by vacuum distillation at 92 ºC at 6 torr pressure. The fractions containing product were collected to yield 3.1 g of **1l** as a colorless liquid (74% yield) that matched previously reported data.[7]

^{1}H NMR (500 MHz, $CDCl_3$): δ 3.05 (m, 1H), 2.85 (s, 6H), 1.87 (s, 3H), 1.74 (m, 2H), 1.59 (m, 3H), 1.30 (m, 4H), 1.19 (m, 1H). ^{13}C NMR{^{1}H} (126 MHz, $CDCl_3$): δ 156.8, 58.0, 38.0, 35.6, 26.0, 25.5, 12.6. HRMS (ESI) *m/z* calc for $C_{10}H_{21}N_2$ $[M+H]^+$ 169.1705, found 169.1694.

Synthesis of (*E*)-*N'*-benzyl-*N*,*N*-dimethylacetimidamide (1m)

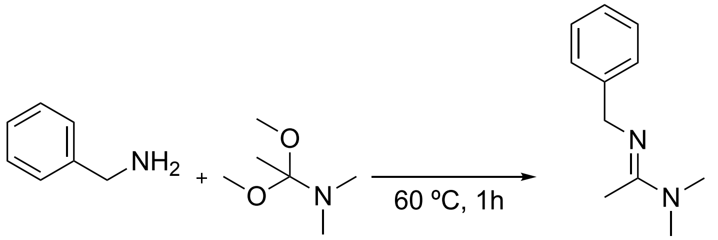

1m, 64% yield

In a nitrogen filled glove box, a 100 mL oven dried round bottom flask equipped with a stir bar was charged with 1,1-dimethoxy-*N*,*N*-dimethylethan-1-amine (3.6 mL, 0.025 mol, 1 equiv) and benzylamine (3.0 mL, 0.025 mol, 1 equiv). The round bottom flask was sealed, taken out of the glove box and placed into a pre-heated oil bath at 60 °C. After stirring for 1 h, the mixture was concentrated under reduced pressure and purified by vacuum distillation at 130 ºC at 6 torr pressure. The fractions containing product were collected to yield 2.8 g of **1m** as a colorless liquid (64% yield) that matched previously reported data.[7]

^{1}H NMR (500 MHz, $CDCl_3$): δ 7.32 (m, 4H), 7.19 (t, *J* = 7.1 Hz, 1H), 4.50 (s, 2H), 2.96 (s, 6H), 1.92 (s, 3H). ^{13}C NMR{^{1}H} (126 MHz, $CDCl_3$): δ 160.2, 142.9, 128.2, 127.2, 126.0, 53.4, 38.3, 13.1. HRMS (ESI) *m/z* calc for $C_{11}H_{17}N_2$ $[M+H]^+$ 177.1392, found 177.1382.

Synthesis of (*E*)-*N*,*N*-dimethyl-*N'*-phenylacetimidamide (1n)

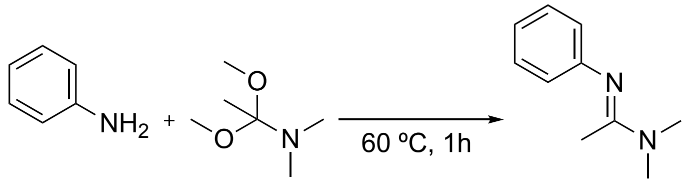

1n, 82% yield

In a nitrogen filled glove box, a 100 mL oven dried round bottom flask equipped with a stir bar was charged with 1,1-dimethoxy-*N*,*N*-dimethylethan-1-amine (3.6 mL, 0.025 mol, 1 equiv) and aniline (2.5 mL, 0.025 mol, 1 equiv). The round bottom flask was sealed, taken out of the glove

box and placed into a pre-heated oil bath at 60 °C. After stirring for 1 h, the mixture was concentrated under reduced pressure and purified by vacuum distillation at 120 ºC at 6 torr pressure. The fractions containing product were collected to yield 3.31 g of **1n** as a colorless liquid (82% yield) that matched previously reported data.[10]

^{1}H NMR (500 MHz, $CDCl_3$): δ 7.23 (t, *J* = 8.0 Hz, 2H), 6.93 (t, *J* = 7.4 Hz, 1H), 6.70 (d, *J* = 7.6 Hz, 2H), 3.02 (s, 6H), 1.86 (s, 3H). ^{13}C NMR{^{1}H} (126 MHz, $CDCl_3$): δ 157.4, 152.4, 128.8, 122.6, 121.4, 38.0, 15.0. HRMS (ESI) *m/z* calc for $C_{10}H_{15}N_2$ $[M+H]^+$ 163.1235, found 163.1225.

CHB of (*E*)-*N'*-(*sec*-butyl)-*N*,*N*-dimethylacetimidamide (2a)

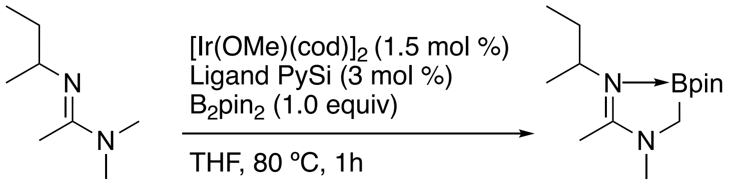

2a, 77% yield

In a nitrogen filled glove box, a 5.0 mL conical vial was charged with $[Ir(OMe)cod]_2$ (10 mg, 1.5 mol %), ligand PySi (6.2 mg, 3.0 mol %), B_2pin_2 (254 mg, 1.0 mmol, 1.0 equiv) and (*E*)-*N'*-(*sec*-butyl)-*N*,*N*-dimethylacetimidamide (142 mg, 1.0 mmol, 1 equiv) in dry THF (3.0 mL). The vial was sealed, taken out of the glove box and stirred in a pre-heated aluminum block at 80 °C. After 1 h, the mixture was concentrated under reduced pressure and purified by gradient column chromatography with activated basic alumina gel (ethyl acetate/methanol 100:0 → ethyl acetate/methanol 95:05). The fractions containing product were collected to yield 206 mg of **2a** as a white solid (77% yield, mp = 121-123 ºC).

^{1}H NMR (500 MHz, $CDCl_3$): δ 3.65 (dt, *J* = 8.8, 6.7 Hz, 1H), 2.92 (s, 3H), 2.29 (s, 2H), 2.02 (s, 3H), 1.74 (m, 1H), 1.60 (m, 1H), 1.28 (d, *J* = 7.1 Hz, 3H), 1.17 (s, 6H), 1.10 (s, 6H), 0.92 (t, *J* = 7.4 Hz, 3H). ^{13}C NMR{^{1}H} (126 MHz, $CDCl_3$): δ 165.0, 78.4, 36.2, 29.7, 26.9, 26.7, 26.4, 20.4,

13.8, 12.3. ^{11}B NMR (160 MHz, $CDCl_3$): δ 8.6. HRMS (ESI) *m/z* calc for $C_{14}H_{30}BN_2O_2$ $[M+H]^+$ 269.2400, found 269.2409.

CHB of (*E*)-*N*,*N*,*N'*-trimethylacetimidamide (2b)

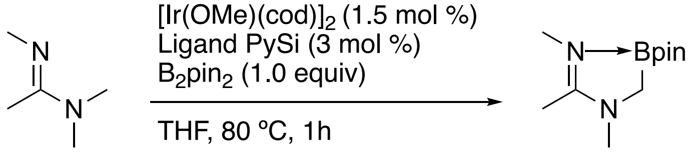

2b, 78% yield

In a nitrogen filled glove box, a 5.0 mL conical vial was charged with $[Ir(OMe)cod]_2$ (10 mg, 1.5 mol %), ligand PySi (6.2 mg, 3.0 mol %), B_2pin_2 (254 mg, 1.0 mmol, 1.0 equiv) and (*E*)-*N*,*N*,*N'*-trimethylacetimidamide (100 mg, 1.0 mmol, 1 equiv) in dry THF (3.0 mL). The vial was sealed, taken out of the glove box and stirred in a pre-heated aluminum block at 80 °C. After 1 h, the mixture was concentrated under reduced pressure and purified by gradient column chromatography with activated basic alumina gel (ethyl acetate/methanol 100:0 → ethyl acetate/methanol 95:05). The fractions containing product were collected to yield 176 mg of **2b** as a white solid (78% yield, mp = 170-173 ºC).

^{1}H NMR (500 MHz, $CDCl_3$): δ 2.96 (s, 3H), 2.88 (s, 3H), 2.32 (s, 2H), 1.93 (s, 3H), 1.18 (s, 6H), 1.10 (s, 6H). ^{13}C NMR{^{1}H} (126 MHz, $CDCl_3$): δ 165.4, 78.4, 36.3, 29.6, 26.3, 25.9, 11.5. ^{11}B NMR (160 MHz, $CDCl_3$): δ 8.3. HRMS (ESI) *m/z* calc for $C_{11}H_{24}BN_2O_2$ $[M+H]^+$ 227.1931, found 227.1935.

CHB of (*E*)-*N'*-ethyl-*N*,*N*-dimethylformimidamide (2c)

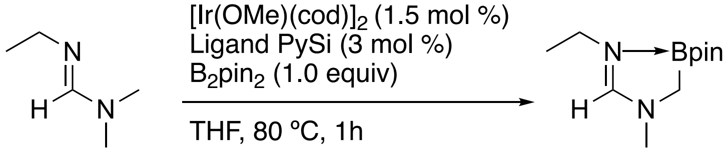

2c, 81% yield

In a nitrogen filled glove box, a 5.0 mL conical vial was charged with $[Ir(OMe)cod]_2$ (10 mg, 1.5 mol %), ligand PySi (6.2 mg, 3.0 mol %), B_2pin_2 (254 mg, 1.0 mmol, 1.0 equiv) and (*E*)-*N'*-ethyl-*N*,*N*-dimethylformimidamide (100 mg, 1.0 mmol, 1 equiv) in dry THF (3.0 mL). The vial was sealed, taken out of the glove box and stirred in a pre-heated aluminum block at 80 °C. After 1 h, the mixture was concentrated under reduced pressure and purified by gradient column chromatography with activated basic alumina gel (ethyl acetate/methanol 100:0 → ethyl acetate/methanol 95:05). The fractions containing product were collected to yield 183 mg of **2c** as a white solid (81% yield, mp = 96-98 ºC).

^{1}H NMR (500 MHz, $CDCl_3$): δ 7.10 (s, 1H), 3.32 (q, J = 7.2 Hz, 2H), 2.95 (s, 3H), 2.32 (s, 2H), 1.26 (t, J = 7.3 Hz, 3H), 1.13 (d, J = 19.8 Hz, 12H). ^{13}C NMR{^{1}H} (126 MHz, $CDCl_3$): δ 157.8, 78.7, 39.3, 38.1, 25.8, 16.7. ^{11}B NMR (160 MHz, $CDCl_3$): δ 9.5. HRMS (ESI) *m/z* calc for $C_{11}H_{24}BN_2O_2$ $[M+H]^+$ 227.1931, found 227.1929.

CHB of (*E*)-*N*,*N*,*N'*-trimethylpropionimidamide (2d)

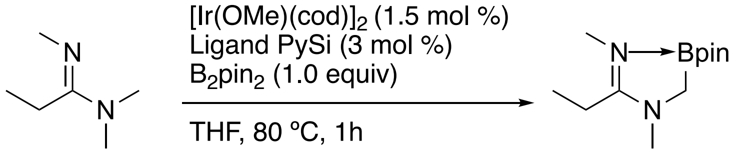

2d, 85% yield

In a nitrogen filled glove box, a 5.0 mL conical vial was charged with $[Ir(OMe)cod]_2$ (10 mg, 1.5 mol %), ligand PySi (6.2 mg, 3.0 mol %), B_2pin_2 (254 mg, 1.0 mmol, 1.0 equiv) and (*E*)-*N*,*N*,*N'*-trimethylpropionimidamide (114 mg, 1.0 mmol, 1 equiv) in dry THF (3.0 mL). The vial was sealed, taken out of the glove box and stirred in a pre-heated aluminum block at 80 °C. After 1 h, the mixture was concentrated under reduced pressure and purified by gradient column chromatography with activated basic alumina gel (ethyl acetate/methanol 100:0 → ethyl

acetate/methanol 95:05). The fractions containing product were collected to yield 204 mg of **2d** as a white solid (85% yield, mp = 97-99 ºC).

^{1}H NMR (500 MHz, $CDCl_3$): δ 2.97 (s, 3H), 2.90 (s, 3H), 2.34 (q, *J* = 7.8 Hz, 2H), 2.31 (s, 2H), 1.18 (s, 6H), 1.10 (s, 6H), 1.10 (t, *J* = 7.7 Hz, 3H). ^{13}C NMR{^{1}H} (126 MHz, $CDCl_3$): δ 169.1, 78.4, 36.0, 29.0, 26.3, 25.9, 18.3, 9.7. ^{11}B NMR (160 MHz, $CDCl_3$): δ 8.2. HRMS (ESI) *m/z* calc for $C_{12}H_{26}BN_2O_2$ $[M+H]^+$ 241.2087, found 241.2087.

CHB of (*E*)-*N'*-ethyl-*N*,*N*-dimethylacetimidamide (2e)

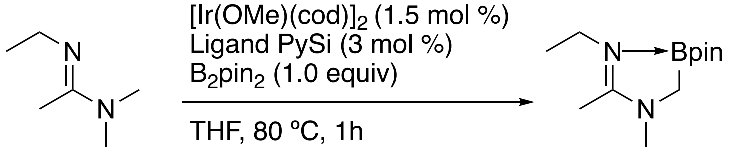

2e, 77% yield

In a nitrogen filled glove box, a 5.0 mL conical vial was charged with $[Ir(OMe)cod]_2$ (10 mg, 1.5 mol %), ligand PySi (6.2 mg, 3.0 mol %), B_2pin_2 (254 mg, 1.0 mmol, 1.0 equiv) and (*E*)-*N'*-ethyl-*N*,*N*-dimethylacetimidamide (114 mg, 1.0 mmol, 1 equiv) in dry THF (3.0 mL). The vial was sealed, taken out of the glove box and stirred in a pre-heated aluminum block at 80 °C. After 1 h, the mixture was concentrated under reduced pressure and purified by gradient column chromatography with activated basic alumina gel (ethyl acetate/methanol 100:0 → ethyl acetate/methanol 95:05). The fractions containing product were collected to yield 184 mg of **2e** as a white solid (77% yield, mp = 167-170 ºC).

^{1}H NMR (500 MHz, $CDCl_3$): δ 3.28 (q, *J* = 7.2 Hz, 2H), 2.94 (s, 3H), 2.30 (s, 2H), 1.95 (s, 3H), 1.19 (t, *J* = 7.2 Hz, 3H), 1.18 (s, 6H), 1.10 (s, 6H). ^{13}C NMR{^{1}H} (126 MHz, $CDCl_3$): δ 165.1, 78.5, 37.5, 36.3, 31.1, 26.3, 25.9, 16.7, 11.2. ^{11}B NMR (160 MHz, $CDCl_3$): δ 8.5. HRMS (ESI) *m/z* calc for $C_{12}H_{26}BN_2O_2$ $[M+H]^+$ 241.2087, found 241.2086.

CHB of (*E*)-*N'*-isopropyl-*N*,*N*-dimethylacetimidamide (2f)

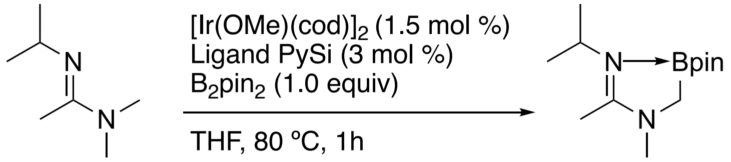

2f, 78% yield

In a nitrogen filled glove box, a 5.0 mL conical vial was charged with $[Ir(OMe)cod]_2$ (10 mg, 1.5 mol %), ligand PySi (6.2 mg, 3.0 mol %), B_2pin_2 (254 mg, 1.0 mmol, 1.0 equiv) and (*E*)-*N'*-isopropyl-*N*,*N*-dimethylacetimidamide (128 mg, 1.0 mmol, 1 equiv) in dry THF (3.0 mL). The vial was sealed, taken out of the glove box and stirred in a pre-heated aluminum block at 80 °C. After 1 h, the mixture was concentrated under reduced pressure and purified by gradient column chromatography with activated basic alumina gel (ethyl acetate/methanol 100:0 → ethyl acetate/methanol 95:05). The fractions containing product were collected to yield 198 mg of **2f** as a white solid (78% yield, mp = 152-155 °C).

^1H NMR (500 MHz, $CDCl_3$): δ 3.99 (hept, *J* = 7.2 Hz, 1H), 2.92 (s, 3H), 2.29 (s, 2H), 2.05 (s, 3H), 1.32 (d, *J* = 7.2 Hz, 6H), 1.18 (s, 6H), 1.10 (s, 6H). ^{13}C NMR{^1H} (126 MHz, $CDCl_3$): δ 164.9, 78.4, 43.9, 36.1, 29.4, 26.7, 26.4, 22.8, 13.7. ^{11}B NMR (160 MHz, $CDCl_3$): δ 8.6. HRMS (ESI) *m/z* calc for $C_{13}H_{28}BN_2O_2$ $[M+H]^+$ 255.2244, found 255.2250.

CHB of (*E*)-*N'*-(*tert*-butyl)-*N*,*N*-dimethylacetimidamide (2g)

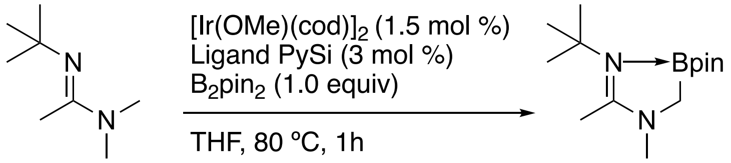

2g, 79% yield

In a nitrogen filled glove box, a 5.0 mL conical vial was charged with $[Ir(OMe)cod]_2$ (10 mg, 1.5 mol %), ligand PySi (6.2 mg, 3.0 mol %), B_2pin_2 (254 mg, 1.0 mmol, 1.0 equiv) and (*E*)-*N'*-(*tert*-

butyl)-*N*,*N*-dimethylacetimidamide (142 mg, 1.0 mmol, 1 equiv) in dry THF (3.0 mL). The vial was sealed, taken out of the glove box and stirred in a pre-heated aluminum block at 80 °C. After 1 h, the mixture was concentrated under reduced pressure and purified by gradient column chromatography with basic alumina gel (ethyl acetate/methanol 100:0 → ethyl acetate/methanol 95:05). The fractions containing product were collected to yield 211 mg of **2g** as a white solid (79% yield, mp = 80-82 ºC).

^{1}H NMR (500 MHz, $CDCl_3$): δ 2.91 (s, 3H), 2.27 (s, 2H), 2.13 (s, 3H), 1.51 (s, 9H), 1.21 (s, 6H), 1.18 (s, 6H). ^{13}C NMR{^{1}H} (126 MHz, $CDCl_3$): δ 166.9, 78.5, 75.1, 54.7, 37.0, 31.6, 25.0, 16.3. ^{11}B NMR (160 MHz, $CDCl_3$): δ 9.0. HRMS (ESI) *m/z* calc for $C_{14}H_{30}BN_2O_2$ $[M+H]^+$ 269.2400, found 269.2409.

CHB of (*E*)-*N'*-isobutyl-*N*,*N*-dimethylacetimidamide (2h)

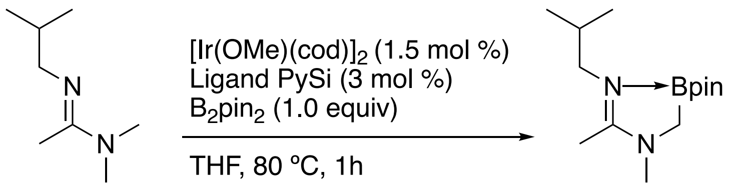

2h, 79% yield

In a nitrogen filled glove box, a 5.0 mL conical vial was charged with $[Ir(OMe)cod]_2$ (10 mg, 1.5 mol %), ligand PySi (6.2 mg, 3.0 mol %), B_2pin_2 (254 mg, 1.0 mmol, 1.0 equiv) and (*E*)-*N'*-isobutyl-*N*,*N*-dimethylacetimidamide (142 mg, 1.0 mmol, 1 equiv) in dry THF (3.0 mL). The vial was sealed, taken out of the glove box and stirred in a pre-heated aluminum block at 80 °C. After 1 h, the mixture was concentrated under reduced pressure and purified by gradient column chromatography with activated basic alumina gel (ethyl acetate/methanol 100:0 → ethyl acetate/methanol 95:05). The fractions containing product were collected to yield 211 mg of **2h** as a white solid (79% yield, mp = 110-112 ºC).

^1H NMR (500 MHz, $CDCl_3$); δ 3.03 (d, J = 7.2 Hz, 2H), 2.95 (s, 3H), 2.31 (s, 2H), 2.18 (m, 1H), 1.94 (s, 3H), 1.15 (s, 6H), 1.09 (s, 6H), 0.86 (d, J = 6.8 Hz, 6H). ^{13}C NMR{^1H} (126 MHz, $CDCl_3$): δ 165.5, 78.5, 50.9, 36.5, 29.6, 26.3, 25.9, 20.7, 12.1. ^{11}B NMR (160 MHz, $CDCl_3$): δ 8.6. HRMS (ESI) *m/z* calc for $C_{14}H_{30}BN_2O_2$ $[M+H]^+$ 269.2400, found 269.2408.

CHB of *N*,*N*-dimethyl-3,4-dihydro-2*H*-pyrrol-5-amine (2i)

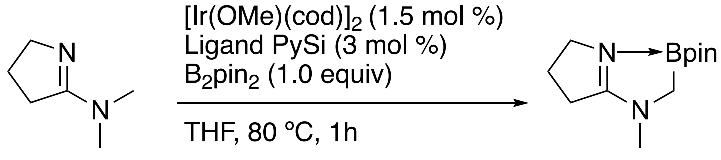

2i, 84% yield

In a nitrogen filled glove box, a 5.0 mL conical vial was charged with $[Ir(OMe)cod]_2$ (10 mg, 1.5 mol %), ligand PySi (6.2 mg, 3.0 mol %), B_2pin_2 (254 mg, 1.0 mmol, 1.0 equiv) and *N*,*N*-dimethyl-3,4-dihydro-2*H*-pyrrol-5-amine (112 mg, 1.0 mmol, 1 equiv) in dry THF (3.0 mL). The vial was sealed, taken out of the glove box and stirred in a pre-heated aluminum block at 80 °C. After 1 h, the mixture was concentrated under reduced pressure and purified by gradient column chromatography with activated basic alumina gel (ethyl acetate/methanol 100:0 → ethyl acetate/methanol 95:05). The fractions containing product were collected to yield 200 mg of **2i** as a white solid (84% yield, mp = 98-100 ºC).

^1H NMR (500 MHz, $CDCl_3$): δ 3.54 (t, J = 7.1 Hz, 2H), 2.89 (s, 3H), 2.60 (s, 2H), 2.44 (t, J = 8.0 Hz, 2H), 2.29 (p, J = 7.7 Hz, 2H), 1.14 (s, 6H), 1.08 (s, 6H). ^{13}C NMR{^1H} (126 MHz, $CDCl_3$): δ 174.7, 78.5, 45.4, 36.4, 29.4, 25.7, 25.4, 25.3, 25.0. ^{11}B NMR (160 MHz, $CDCl_3$): δ 7.4. HRMS (ESI) *m/z* calc for $C_{12}H_{24}BN_2O_2$ $[M+H]^+$ 239.1931, found 239.1935.

CHB of *N,N*-dimethyl-3,4,5,6-tetrahydropyridin-2-amine (2j)

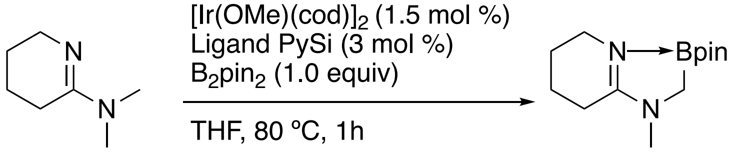

2j, 83% yield

In a nitrogen filled glove box, a 5.0 mL conical vial was charged with $[Ir(OMe)cod]_2$ (10 mg, 1.5 mol %), ligand PySi (6.2 mg, 3.0 mol %), B_2pin_2 (254 mg, 1.0 mmol, 1.0 equiv) and *N,N*-dimethyl-3,4,5,6-tetrahydropyridin-2-amine (126 mg, 1.0 mmol, 1 equiv) in dry THF (3.0 mL). The vial was sealed, taken out of the glove box and stirred in a pre-heated aluminum block at 80 °C. After 1 h, the mixture was concentrated under reduced pressure and purified by gradient column chromatography with activated basic alumina gel (ethyl acetate/methanol 100:0 → ethyl acetate/methanol 95:05). The fractions containing product were collected to yield 209 mg of **2j** as a white solid (83% yield, mp = 205-207 ºC).

^1H NMR (500 MHz, $CDCl_3$): δ 3.36 (t, J = 5.7 Hz, 2H), 2.86 (s, 3H), 2.28 (s, 2H), 2.24 (t, J = 6.3 Hz, 2H), 1.69 (m, 4H), 1.15 (s, 6H), 1.09 (s, 6H). ^{13}C NMR{^1H} (126 MHz, $CDCl_3$): δ 164.5, 78.3, 39.8, 34.9, 26.1, 25.7, 23.0, 22.0, 19.1. ^{11}B NMR (160 MHz, $CDCl_3$): δ 8.4. HRMS (ESI) *m/z* calc for $C_{13}H_{26}BN_2O_2$ $[M+H]^+$ 253.2087, found 253.2093.

CHB of *N,N*-dimethyl-3,4,5,6-tetrahydro-2H-azepin-7-amine (2k)

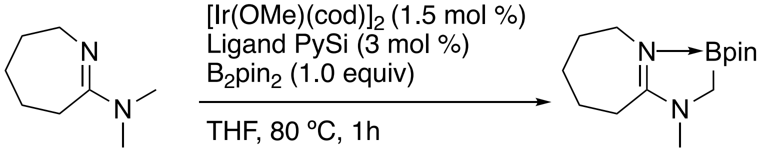

2k, 82% yield

In a nitrogen filled glove box, a 5.0 mL conical vial was charged with $[Ir(OMe)cod]_2$ (10 mg, 1.5 mol %), ligand PySi (6.2 mg, 3.0 mol %), B_2pin_2 (254 mg, 1.0 mmol, 1.0 equiv) and *N,N*-dimethyl-3,4,5,6-tetrahydro-2H-azepin-7-amine (140 mg, 1.0 mmol, 1 equiv) in dry THF (3.0 mL). The vial

was sealed, taken out of the glove box and stirred in a pre-heated aluminum block at 80 °C. After 1 h, the mixture was concentrated under reduced pressure and purified by gradient column chromatography with activated basic alumina gel (ethyl acetate/methanol 100:0 → ethyl acetate/methanol 95:05). The fractions containing product were collected to yield 218 mg of **2k** as a white solid (82% yield, mp = 145-147 ºC).

^{1}H NMR (500 MHz, $CDCl_3$): δ 3.35 (m, 2H), 2.99 (s, 3H), 2.45 (m, 2H), 2.35 (s, 2H), 1.73 (m, 2H), 1.60 (m, 4H), 1.16 (s, 6H), 1.09 (s, 6H). ^{13}C NMR{^{1}H} (126 MHz, $CDCl_3$): δ 171.1, 78.2, 42.9, 36.5, 30.8, 28.8, 26.6, 26.1, 25.5, 23.4. ^{11}B NMR (160 MHz, $CDCl_3$): δ 8.1. HRMS (ESI) *m/z* calc for $C_{14}H_{28}BN_2O_2$ $[M+H]^+$ 267.2244, found 267.2252.

CHB of (*E*)-N'-cyclohexyl-N,N-dimethylacetimidamide (2l)

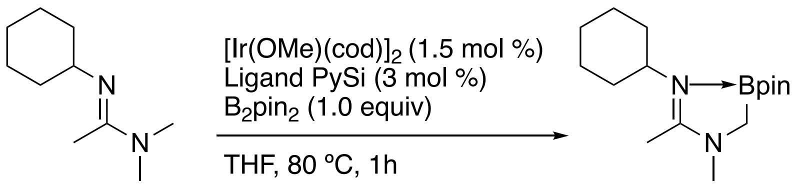

2l, 84% yield

In a nitrogen filled glove box, a 5.0 mL conical vial was charged with $[Ir(OMe)cod]_2$ (10 mg, 1.5 mol %), ligand PySi (6.2 mg, 3.0 mol %), B_2pin_2 (254 mg, 1.0 mmol, 1.0 equiv) and (*E*)-N'-cyclohexyl-N,N-dimethylacetimidamide (168 mg, 1.0 mmol, 1 equiv) in dry THF (3.0 mL). The vial was sealed, taken out of the glove box and stirred in a pre-heated aluminum block at 80 °C. After 1 h, the mixture was concentrated under reduced pressure and purified by gradient column chromatography with activated basic alumina gel (ethyl acetate/methanol 100:0 → ethyl acetate/methanol 95:05). The fractions containing product were collected to yield 247 mg of **2l** as a white solid (84% yield, mp = 154-156 ºC).

^{1}H NMR (500 MHz, $CDCl_3$): δ 3.50 (tt, J = 12.6, 3.8 Hz, 1H), 2.91 (s, 3H), 2.27 (s, 2H), 2.04 (s, 3H), 1.81 (m, 5H), 1.65 (m, 3H), 1.28 (qt, J = 13.0, 3.6 Hz, 2H), 1.18 (s, 6H), 1.09 (s, 6H). ^{13}C

NMR{^{1}H} (126 MHz, $CDCl_3$): δ 164.8, 78.4, 36.3, 32.8, 26.8, 26.7, 26.5, 25.9, 13.8. ^{11}B NMR (160 MHz, $CDCl_3$): δ 8.6. HRMS (ESI) *m/z* calc for $C_{16}H_{32}BN_2O_2$ $[M+H]^+$ 295.2557, found 295.2565.

CHB of (*E*)-*N*'-benzyl-*N*,*N*-dimethylacetimidamide (2m)

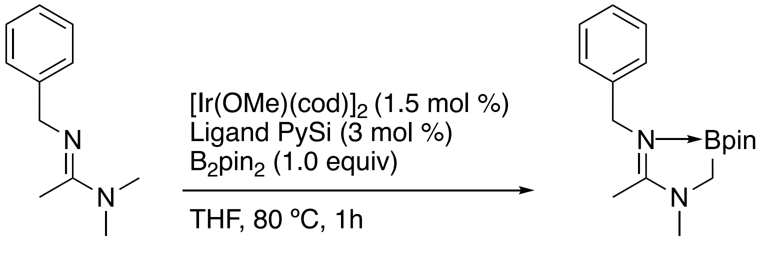

2m, 82% yield

In a nitrogen filled glove box, a 5.0 mL conical vial was charged with $[Ir(OMe)cod]_2$ (10 mg, 1.5 mol %), ligand PySi (6.2 mg, 3.0 mol %), B_2pin_2 (254 mg, 1.0 mmol, 1.0 equiv) and (*E*)-*N*'-benzyl-*N*,*N*-dimethylacetimidamide (176 mg, 1.0 mmol, 1 equiv) in dry THF (3.0 mL). The vial was sealed, taken out of the glove box and stirred in a pre-heated aluminum block at 80 °C. After 1 h, the mixture was concentrated under reduced pressure and purified by gradient column chromatography with activated basic alumina gel (ethyl acetate/methanol 100:0 → ethyl acetate/methanol 90:10). The fractions containing product were collected to yield 247 mg of **2m** as a white solid (82% yield, mp = 157-159 ºC).

^{1}H NMR (500 MHz, $CDCl_3$): δ 7.45 (d, *J* = 7.6 Hz, 2H), 7.30 (t, *J* = 7.6 Hz, 2H), 7.20 (t, *J* = 7.5 Hz, 1H), 4.47 (s, 2H), 2.95 (s, 3H), 2.44 (s, 2H), 1.80 (s, 3H), 1.13 (s, 6H), 1.10 (s, 6H). ^{13}C NMR{^{1}H} (126 MHz, $CDCl_3$): δ 139.8, 128.6, 127.6, 126.8, 78.7, 46.8, 36.4, 26.3, 26.0, 12.2. ^{11}B NMR (160 MHz, $CDCl_3$): δ 8.4. HRMS (ESI) *m/z* calc for $C_{17}H_{28}BN_2O_2$ $[M+H]^+$ 303.2244, found 303.2250.

CHB of (*E*)-*N*,*N*-dimethyl-*N'*-phenylacetimidamide (2n)

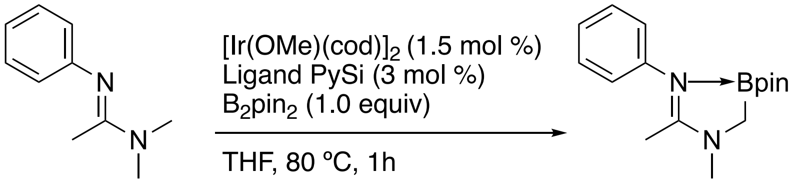

2n, 83% yield

In a nitrogen filled glove box, a 5.0 mL conical vial was charged with $[Ir(OMe)cod]_2$ (10 mg, 1.5 mol %), ligand PySi (6.2 mg, 3.0 mol %), B_2pin_2 (254 mg, 1.0 mmol, 1.0 equiv) and (*E*)-*N*,*N*-dimethyl-*N'*-phenylacetimidamide (162 mg, 1.0 mmol, 1 equiv) in dry THF (3.0 mL). The vial was sealed, taken out of the glove box and stirred in a pre-heated aluminum block at 80 °C. After 1 h, the mixture was concentrated under reduced pressure and purified by gradient column chromatography with activated basic alumina gel (ethyl acetate/methanol 100:0 → ethyl acetate/methanol 95:05). The fractions containing product were collected to yield 239 mg of **2n** as a white solid (83% yield, mp = 163-165 ºC).

^{1}H NMR (500 MHz, $CDCl_3$): δ 7.32 (t, *J* = 7.6 Hz, 2H), 7.23 (m, 1H), 7.13 (d, *J* = 7.3 Hz, 2H), 3.03 (s, 3H), 2.48 (s, 2H), 1.79 (s, 3H), 1.02 (s, 6H), 0.59 (s, 6H). ^{13}C NMR{^{1}H} (126 MHz, $CDCl_3$): δ 164.9, 141.2, 128.6, 128.5, 126.6, 78.8, 36.4, 25.9, 25.4, 12.8. ^{11}B NMR (160 MHz, $CDCl_3$): δ 9.0. HRMS (ESI) *m/z* calc for $C_{16}H_{26}BN_2O_2$ $[M+H]^+$ 289.2087, found 289.2096.

Synthesis of (*E*)-*N*,*N*-diethyl-*N'*-methylacetimidamide (3a)

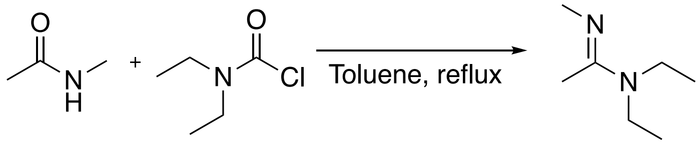

3a, 85% yield

In a 100 mL oven dried round bottom flask equipped with a stir bar was charged with N-methylacetamide (5.0 mL, 0.065 mol, 1 equiv), Diethylcarbamic chloride (8.3 mL, 0.065 mol, 1 equiv) in toluene (2.6 mL) was added dropwise over 1 h period with stirring. The flask was placed

in an oil bath which was heated to allow for the contents to reflux. After 6 h, the mixture was concentrated under reduced pressure and then on high vacuum. The residue was covered with a layer of ether (15 mL). While cooling in ice, potassium carbonate (2.6 g) was added and then a solution of sodium hydroxide (2.6 g in 15 mL water) was added dropwise. The ether phase was separated and the aqueous phase was extracted three times with ether. The organics were combined and dried over magnesium sulfate. After filtration and concentration, the residue was purified by distillation at 160 ºC at 760 torr pressure. The fractions containing product were collected to yield 7.0 g of **3a** as a colorless liquid (85% yield). Title compound has been reported before but no spectroscopic data was provided.[4]

^{1}H NMR (500 MHz, $CDCl_3$); δ 3.29 (q, J = 7.1 Hz, 4H), 2.97 (s, 3H), 1.89 (s, 3H), 1.08 (t, J = 7.1 Hz, 6H). ^{13}C NMR{^{1}H} (126 MHz, $CDCl_3$): δ 159.1, 41.7, 37.0, 13.9, 12.3. HRMS (ESI) *m/z* calc for $C_7H_{17}N_2$ [M+H]$^+$ 129.1391, found 129.1386

Synthesis of (*E*)-*N*,*N*-diethyl-*N'*-methylpropionimidamide (3b)

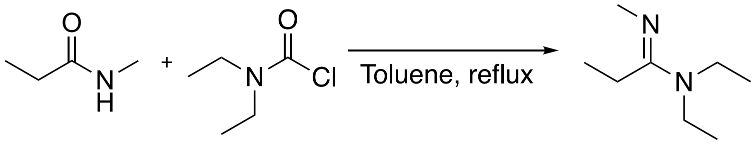

3b, 87% yield

In a 100 mL oven dried round bottom flask equipped with a stir bar was charged with N-methylpropionamide (5.84 mL, 0.065 mol, 1 equiv), Diethylcarbamic chloride (8.3 mL, 0.065 mol, 1 equiv) in toluene (2.6 mL) was added dropwise over 1 h period with stirring. The flask was placed in an oil bath which was heated to allow for the contents to reflux. After 6 h, the mixture was concentrated under reduced pressure and then on high vacuum. The residue was covered with a layer of ether (15 mL). While cooling in ice, potassium carbonate (2.6 g) was added and then a solution of sodium hydroxide (2.6 g in 15 mL water) was added dropwise. The ether phase was

separated and the aqueous phase was extracted three times with ether. The organics were combined and dried over magnesium sulfate. After filtration and concentration, the residue was purified by vacuum distillation at 92 ºC at 40 torr pressure. The fractions containing product were collected to yield 8.0 g of **3b** as a colorless liquid (87% yield).

^{1}H NMR (500 MHz, $CDCl_3$); δ 3.25 (q, J = 7.1 Hz, 4H), 3.0 (s, 3H), 2.30 (q, J = 7.7 Hz, 2H), 1.05 (t, J = 6.8 Hz, 6H), 1.04 (t, J = 7.6 Hz, 3H). ^{13}C NMR{^{1}H} (126 MHz, $CDCl_3$): δ 163.2, 41.1, 36.2, 18.8, 14.0, 11.3. HRMS (ESI) *m/z* calc for $C_8H_{19}N_2$ $[M+H]^+$ 143.1548, found 143.1545

Synthesis of (*E*)-*N*,*N*,*N'*-triethylacetimidamide (3c)

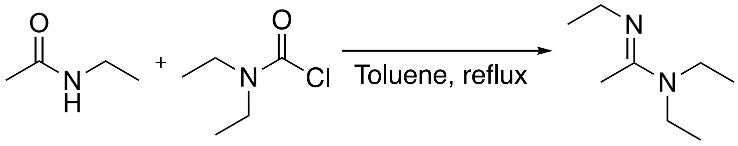

3c, 41% yield

In a 100 mL oven dried round bottom flask equipped with a stir bar was charged with N-ethylacetamide (6.1 mL, 0.065 mol, 1 equiv), Diethylcarbamic chloride (8.3 mL, 0.065 mol, 1 equiv) in toluene (2.6 mL) was added dropwise over 1 h period with stirring. The flask was placed in an oil bath which was heated to allow for the contents to reflux. After 6 h, the mixture was concentrated under reduced pressure and then on high vacuum. The residue was covered with a layer of ether (15 mL). While cooling in ice, potassium carbonate (2.6 g) was added and then a solution of sodium hydroxide (2.6 g in 15 mL water) was added dropwise. The ether phase was separated and the aqueous phase was extracted three times with ether. The organics were combined and dried over magnesium sulfate. After filtration and concentration, the residue was purified by vacuum distillation at 60 ºC at 6 torr pressure. The fractions containing product were collected to yield 3.79 g of **3c** as a colorless liquid (41% yield). Title compound has been reported before but no spectroscopic data was provided.[11]

^{1}H NMR (500 MHz, $CDCl_3$); δ 3.29 (q, J = 7.1 Hz, 4H), 3.22 (q, J = 7.4 Hz, 2H), 1.87 (s, 3H), 1.09 (t, J = 7.3 Hz, 3H), 1.07 (t, J = 7.0 Hz, 6H). ^{13}C NMR{^{1}H} (126 MHz, $CDCl_3$): δ 157.0, 44.0, 41.6, 17.4, 13.9, 12.1. HRMS (ESI) *m/z* calc for $C_8H_{19}N_2$ $[M+H]^+$ 143.1548, found 143.1544

Synthesis of (*E*)-*N*,*N*,*N*'-triethylacetimidamide (3d)

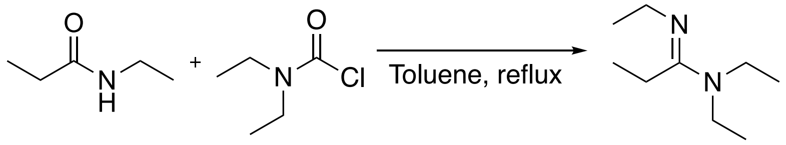

3d, 81% yield

In a 100 mL oven dried round bottom flask equipped with a stir bar was charged with N-ethylpropionamide (6.57 mL, 0.065 mol, 1 equiv), Diethylcarbamic chloride (8.3 mL, 0.065 mol, 1 equiv) in toluene (2.6 mL) was added dropwise over 1 h period with stirring. The flask was placed in an oil bath which was heated to allow for the contents to reflux. After 6 h, the mixture was concentrated under reduced pressure and then on high vacuum. The residue was covered with a layer of ether (15 mL). While cooling in ice, potassium carbonate (2.6 g) was added and then a solution of sodium hydroxide (2.6 g in 15 mL water) was added dropwise. The ether phase was separated and the aqueous phase was extracted three times with ether. The organics were combined and dried over magnesium sulfate. After filtration and concentration, the residue was purified by vacuum distillation at 62 ºC at 6 torr pressure. The fractions containing product were collected to yield 8.2 g of **3d** as a colorless liquid (81% yield). Title compound has been reported before but no spectroscopic data was provided.[12]

^{1}H NMR (500 MHz, $CDCl_3$): δ 3.27 (q, J = 7.0 Hz, 4H), 3.25 (q, J = 7.2 Hz, 2H), 2.28 (q, J = 7.6 Hz, 2H), 1.12 (t, J = 7.3 Hz, 3H), 1.07 (t, J = 7.1 Hz, 6H), 1.06 (t, J = 7.6 Hz, 3H). ^{13}C NMR{^{1}H} (126 MHz, $CDCl_3$): δ 161.2, 43.1, 41.2, 18.9, 18.0, 14.0, 12.1. HRMS (ESI) *m/z* calc for $C_9H_{21}N_2$ $[M+H]^+$ 157.1704, found 157.1689

Synthesis of (*E*)-*N*-ethyl-1-(piperidin-1-yl)ethan-1-imine (3e)

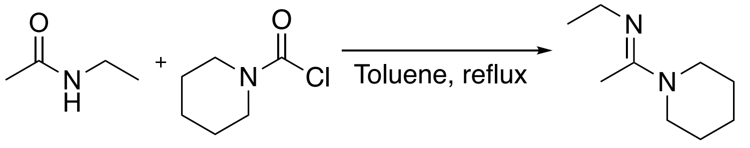

3e, 77% yield

In a 100 mL oven dried round bottom flask equipped with a stir bar was charged with N-ethylacetamide (4.75 mL, 0.065 mol, 1 equiv), Piperidine-1-carbonyl chloride (9.59 g, 0.065 mol, 1 equiv) in toluene (2.6 mL) was added dropwise over 1 h period with stirring. The flask was placed in an oil bath which was heated to allow for the contents to reflux. After 6 h, the mixture was concentrated under reduced pressure and then on high vacuum. The residue was covered with a layer of ether (15 mL). While cooling in ice, potassium carbonate (2.6 g) was added and then a solution of sodium hydroxide (2.6 g in 15 mL water) was added dropwise. The ether phase was separated and the aqueous phase was extracted three times with ether. The organics were combined and dried over magnesium sulfate. After filtration and concentration, the residue was purified by vacuum distillation at 72 ºC at 0.1 torr pressure. The fractions containing product were collected to yield 7.7 g of **3e** as a colorless liquid (77% yield).

^{1}H NMR (500 MHz, $CDCl_3$); δ 3.26 (m, 4H), 3.22 (q, *J* = 7.1 Hz, 2H), 1.85 (s, 3H), 1.53 (m, 6H), 1.11 (t, *J* = 7.3 Hz, 3H). ^{13}C NMR{^{1}H} (126 MHz, $CDCl_3$): δ 159.2, 46.4, 44.1, 26.0, 25.0, 17.3, 12.8. HRMS (ESI) *m/z* calc for $C_9H_{19}N_2$ $[M+H]^+$ 155.1548, found 155.1542

Synthesis of *N*,*N*-diethyl-3,4-dihydro-2*H*-pyrrol-5-amine (3f)

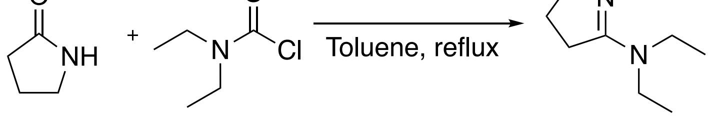

3f, 79% yield

In a 100 mL oven dried round bottom flask equipped with a stir bar was charged with pyrrolidin-2-one (10.3 mL, 0.125 mol, 1 equiv), Diethylcarbamic chloride (15.8 mL, 0.125 mol, 1 equiv) in toluene (2.6 mL) was added dropwise over 1 h period with stirring. The flask was placed in an oil bath which was heated to allow for the contents to reflux. After 6 h, the mixture was concentrated under reduced pressure and then on high vacuum. The residue was covered with a layer of ether (15 mL). While cooling in ice, potassium carbonate (5.2 g) was added and then a solution of sodium hydroxide (5.2 g in 30 mL water) was added dropwise. The ether phase was separated and the aqueous phase was extracted three times with ether. The organics were combined and dried over magnesium sulfate. After filtration and concentration, the residue was purified by vacuum distillation at 58 ºC at 0.1 torr pressure. The fractions containing product were collected to yield 13.8 g of **3f** as a colorless liquid (79% yield) that matched previously reported spectrum.[13]

^{1}H NMR (500 MHz, $CDCl_3$); δ 3.63 (t, *J* = 6.9 Hz, 2H), 3.27 (q, *J* = 7.1 Hz, 4H), 2.47 (t, *J* = 8.3 Hz, 2H), 1.92 (m, 2H), 1.09 (t, *J* = 7.1 Hz, 6H). ^{13}C NMR{^{1}H} (126 MHz, $CDCl_3$): δ 167.4, 56.6, 42.8, 42.4, 31.6, 24.0, 13.8, 13.4. HRMS (ESI) *m/z* calc for $C_8H_{17}N_2$ $[M+H]^+$ 141.1391, found 141.1387

Synthesis of N,N-diethyl-3,4,5,6-tetrahydropyridin-2-amine (3g)

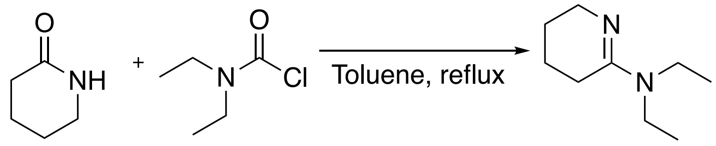

3g, 79% yield

In a 100 mL oven dried round bottom flask equipped with a stir bar was charged with piperidin-2-one (6.4 mL, 0.065 mol, 1 equiv), Diethylcarbamic chloride (8.3 mL, 0.065 mol, 1 equiv) in toluene (2.6 mL) was added dropwise over 1 h period with stirring. The flask was placed in an oil bath which was heated to allow for the contents to reflux. After 6 h, the mixture was concentrated

under reduced pressure and then on high vacuum. The residue was covered with a layer of ether (15 mL). While cooling in ice, potassium carbonate (2.6 g) was added and then a solution of sodium hydroxide (2.6 g in 15 mL water) was added dropwise. The ether phase was separated and the aqueous phase was extracted three times with ether. The organics were combined and dried over magnesium sulfate. After filtration and concentration, the residue was purified by vacuum distillation at 64 ºC at 0.1 torr pressure. The fractions containing product were collected to yield 7.9 g of **3g** as a colorless liquid (79% yield). Title compound has been reported before but no spectroscopic data was provided.[14]

^{1}H NMR (500 MHz, $CDCl_3$): δ 3.44 (m, 2H), 3.22 (m, 4H), 2.16 (m, 2H), 1.68 (m, 2H),1.46 (m, 2H), 1.03 (m, 6H). ^{13}C NMR{^{1}H} (126 MHz, $CDCl_3$): δ 157.3, 47.4, 42.4, 40.7, 25.0, 22.9, 21.0, 13.8, 13.4. HRMS (ESI) *m/z* calc for $C_9H_{19}N_2$ $[M+H]^+$ 155.1548, found 155.1540

Synthesis of N,N-diethyl-3,4,5,6-tetrahydropyridin-2-amine (3h)

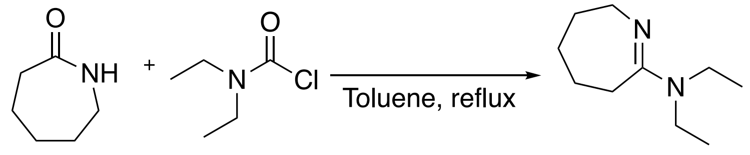

3h, 77% yield

In a 100 mL oven dried round bottom flask equipped with a stir bar was charged with azepan-2-one (7.35 g, 0.065 mol, 1 equiv), Diethylcarbamic chloride (8.3 mL, 0.065 mol, 1 equiv) in toluene (2.6 mL) was added dropwise over 1 h period with stirring. The flask was placed in an oil bath which was heated to allow for the contents to reflux. After 6 h, the mixture was concentrated under reduced pressure and then on high vacuum. The residue was covered with a layer of ether (15 mL). While cooling in ice, potassium carbonate (2.6 g) was added and then a solution of sodium hydroxide (2.6 g in 15 mL water) was added dropwise. The ether phase was separated and the aqueous phase was extracted three times with ether. The organics were combined and dried over

magnesium sulfate. After filtration and concentration, the residue was purified by vacuum distillation at 64 ºC at 0.1 torr pressure. The fractions containing product and tetraethyl urea (22%) were collected to yield 6.5 g of **3h** as a colorless liquid as determined by ^{1}H NMR (60% yield of 3h).

^{1}H NMR (500 MHz, $CDCl_3$): δ 3.43 (m, 2H), 3.27 (q, J = 6.9 Hz, 4H), 2.44 (m, 2H), 1.71 (m, 2H), 1.53 (m, 2H), 1.46 (m, 2H), 1.08 (t, J = 7.0 Hz, 6H). ^{13}C NMR{^{1}H} (126 MHz, $CDCl_3$): δ 166.1, 49.6, 42.5, 42.4, 31.1, 29.0, 27.0, 24.3, 14.3, 13.4 HRMS (ESI) *m/z* calc for $C_{10}H_{21}N_2$ $[M+H]^+$ 169.1704, found 169. 1691

Synthesis of B_2eg_2 (2,2'-bi(1,3,2-dioxaborolane))

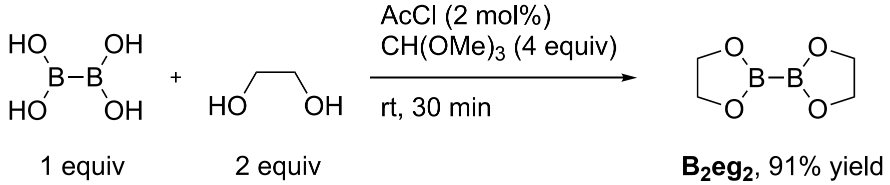

An oven dried round bottom flask was charged with $B_2(OH)_4$ (2.24 g, 25 mmol), $CH(OMe)_3$ (10.94 mL, 100 mmol) and a stir bar. AcCl (18 µL, 0.25 mmol) was added, the vessel was flushed with N_2 and sealed with a silicone septum and the mixture was stirred at room temperature until all solid material had dissolved (5 minutes). Ethylene glycol (2.8 mL, 50 mmol) was then added and the mixture was stirred for 30 minutes. Solvent was removed in vacuo via rotary evaporation and then on high vacuum to afford the title compound. This procedure afforded 3.22 g of **B_2eg_2** as a white solid (91%yield, mp = 163-164 ºC) that matched previously reported spectra.[15]

^{1}H NMR (500 MHz, $CDCl_3$): δ 4.18 (s, 8H); ^{13}C NMR (126 MHz, $CDCl_3$): δ 65.7; ^{11}B NMR (160 MHz, $CDCl_3$): δ 30.82. GC-MS (EI) *m/z* calcd for $C_4H_8B_2O_4$ [M] 142.06, found: 142.1

Synthesis of B_2pg_2 (4,4'-dimethyl-2,2'-bi(1,3,2-dioxaborolane))

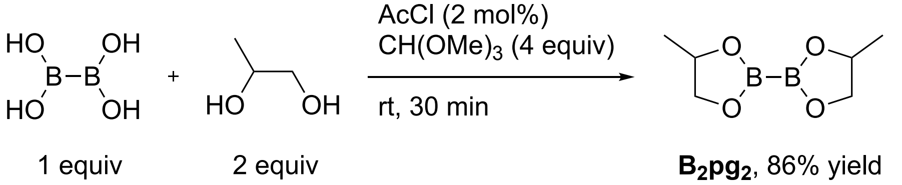

An oven dried round bottom flask was charged with $B_2(OH)_4$ (2.24 g, 25 mmol), $CH(OMe)_3$ (10.94 mL, 100 mmol) and a stir bar. AcCl (18 µL, 0.25 mmol) was added, the vessel was flushed with N_2 and sealed with a silicone septum and the mixture was stirred at room temperature until all solid material had dissolved (5 minutes). Propylene glycol (3.65 mL, 50 mmol) was then added and the mixture was stirred for 30 minutes. The mixture was concentrated and then distilled at 74 ºC at 0.1 torr pressure using a short path distillation apparatus to yield 3.64 g of **B_2pg_2** as a colorless oil that matched previously reported spectra (86% yield).[15]

^{1}H NMR (500 MHz, $CDCl_3$): δ 4.62 – 4.51 (m, 2H), 4.26 (dd, J = 9.0, 7.9 Hz, 2H), 3.71 (ddd, J = 9.0, 7.4, 1.0 Hz, 2H), 1.33 (dd, J = 6.2, 0.5 Hz, 6H). ^{13}C NMR{^{1}H} (126 MHz, $CDCl_3$): δ 73.6, 72.1, 21.8. ^{11}B NMR (160 MHz, $CDCl_3$): δ 30.7. HRMS (GC/ToF) *m/z* calc for $C_6H_{12}B_2O_4$ $[M]^+$ 170.0922, found: 170.0911

Synthesis of B_2bg_2 (4,4'-diethyl-2,2'-bi(1,3,2-dioxaborolane))

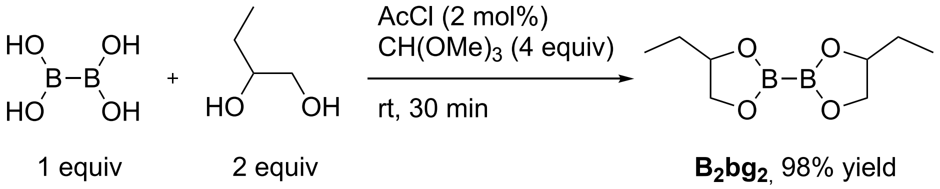

An oven dried round bottom flask was charged with $B_2(OH)_4$ (2.24 g, 25 mmol), $CH(OMe)_3$ (10.94 mL, 100 mmol) and a stir bar. AcCl (18 µL, 0.25 mmol) was added, the vessel was flushed with N_2 and sealed with a silicone septum and the mixture was stirred at room temperature until all solid material had dissolved (5 minutes). 1,2-butandiol (4.5 mL, 50 mmol) was then added and the

mixture was stirred for 30 minutes. The mixture was concentrated and then distilled at 82 ºC at 0.1 torr pressure using a short path distillation apparatus to yield 4.85 g of **B_2bg_2** as a colorless oil that matched previously reported spectra (98% yield).[15]

^{1}H NMR (500 MHz, $CDCl_3$): δ 4.40 – 4.30 (m, 2H), 4.23 (dd, J = 9.0, 8.1 Hz, 2H), 3.77 (ddd, J = 9.0, 7.3, 1.7 Hz, 2H), 1.73 – 1.50 (m, 4H), 0.94 (t, J = 7.4 Hz, 6H). ^{13}C NMR{^{1}H} (126 MHz, $CDCl_3$): δ 78.6, 70.4, 29.0, 9.3. ^{11}B NMR (160 MHz, $CDCl_3$): δ 30.7. HRMS (GC/ToF) *m/z* calc for $C_8H_{16}B_2O_4$ $[M]^+$ 198.1235, found: 198.1224

Synthesis of B_2mbg_2 (4,4,4',4'-tetramethyl-2,2'-bi(1,3,2-dioxaborinane))

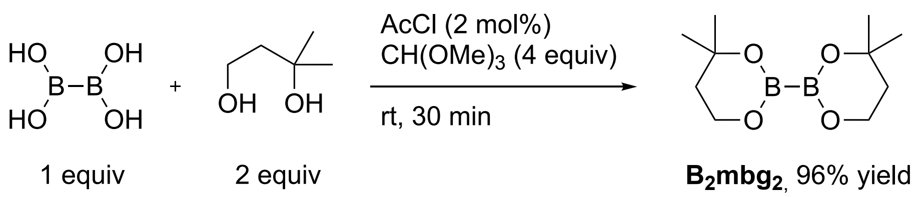

An oven dried round bottom flask was charged with $B_2(OH)_4$ (2.24 g, 25 mmol), $CH(OMe)_3$ (10.94 mL, 100 mmol) and a stir bar. AcCl (18 μL, 0.25 mmol) was added, the vessel was flushed with N_2 and sealed with a silicone septum and the mixture was stirred at room temperature until all solid material had dissolved (5 minutes). 3-methylbutane-1,3-diol (9.15 mL, 50 mmol) was then added and the mixture was stirred for 30 minutes. Solvent was removed in vacuo via rotary evaporation and then on high vacuum to afford the title compound. This procedure afforded 5.04 g of **B_2mbg_2** as a white solid (96%yield, mp = 62-64 ºC) that matched previously reported spectra.[15]

^{1}H NMR (500 MHz, $CDCl_3$): δ 3.99 (m, 4H), 1.78 (m, 4H), 1.31 (s, 12H); ^{13}C NMR (126 MHz, $CDCl_3$): δ 69.6, 58.7, 38.5, 29.5; ^{11}B NMR (160 MHz, $CDCl_3$): δ 28.01. GC-MS (EI) *m/z* calcd for $C_{10}H_{20}B_2O_4$ [M] 226.15, found: 226.15

Optimization of Reaction Conditions

CHB of *(E)-N,N*-diethyl-*N'*-methylacetimidamide with B_2pin_2

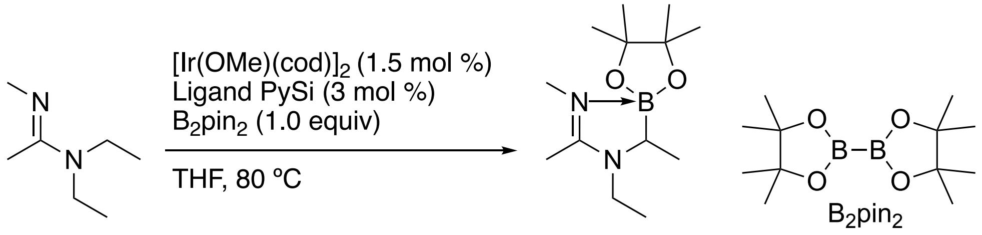

In a nitrogen filled glove box, a 5.0 mL conical vial was charged with $[Ir(OMe)cod]_2$ (10 mg, 1.5 mol %), ligand PySi (6.2 mg, 3.0 mol %), B_2pin_2 (254 mg, 1.0 mmol, 1.0 equiv) and (*E*)-*N,N*-diethyl-*N*'-methylacetimidamide (128 mg, 1.0 mmol, 1 equiv) in dry THF (3.0 mL). The vial was sealed, taken out of the glove box and stirred in a pre-heated aluminum block at 80 °C. After 1 h, 3 h, 6 h, 9 h, 12 h and 24 h, an aliquot was removed and ^{1}H NMR of crude material was collected. A complex mixture of products was observed.

CHB of (E)-N,N-diethyl-N'-methylacetimidamide with HBpin

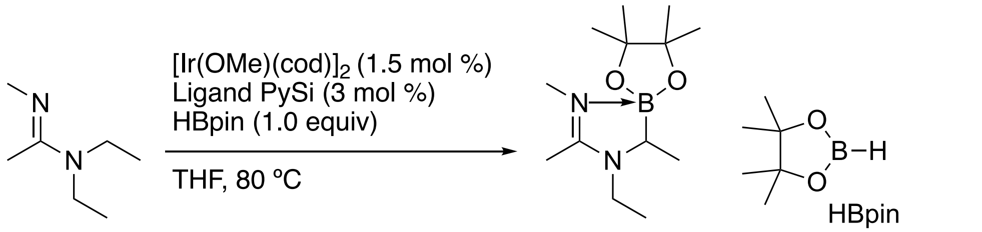

In a nitrogen filled glove box, a 5.0 mL conical vial was charged with $[Ir(OMe)cod]_2$ (10 mg, 1.5 mol %), ligand PySi (6.2 mg, 3.0 mol %), HBpin (128 mg, 1.0 mmol, 1.0 equiv) and (*E*)-*N,N*-diethyl-*N*'-methylacetimidamide (128 mg, 1.0 mmol, 1 equiv) in dry THF (3.0 mL). The vial was sealed, taken out of the glove box and stirred in a pre-heated aluminum block at 80 °C. After 19 h, an aliquot was removed and ^{1}H NMR of crude material was collected. A complex mixture of products was observed.

CHB of (E)-N,N-diethyl-N'-methylacetimidamide with B_2mbg_2

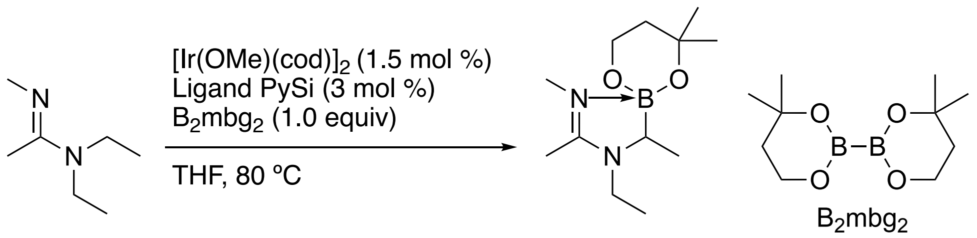

In a nitrogen filled glove box, a 5.0 mL conical vial was charged with $[Ir(OMe)cod]_2$ (10 mg, 1.5 mol %), ligand PySi (6.2 mg, 3.0 mol %), B_2mbg_2 (226 mg, 1.0 mmol, 1.0 equiv) and (*E*)-*N,N*-diethyl-*N'*-methylacetimidamide (128 mg, 1.0 mmol, 1 equiv) in dry THF (3.0 mL). The vial was sealed, taken out of the glove box and stirred in a pre-heated aluminum block at 80 °C. After 24 h, an aliquot was removed and ^1H NMR of crude material was collected. Only starting material was observed.

CHB of *(E)-N,N*-diethyl-*N'*-methylacetimidamide with B_2bg_2

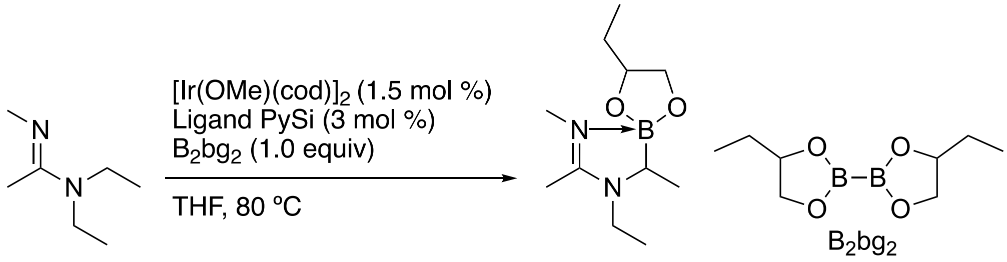

In a nitrogen filled glove box, a 5.0 mL conical vial was charged with $[Ir(OMe)cod]_2$ (10 mg, 1.5 mol %), ligand PySi (6.2 mg, 3.0 mol %), B_2bg_2 (198 mg, 1.0 mmol, 1.0 equiv) and (*E*)-*N,N*-diethyl-*N'*-methylacetimidamide (128 mg, 1.0 mmol, 1 equiv) in dry THF (3.0 mL). The vial was sealed, taken out of the glove box and stirred in a pre-heated aluminum block at 80 °C. After 24 h, an aliquot was removed and ^1H NMR of crude material was collected. 15% conversion of starting material was observed.

CHB of *(E)-N,N*-diethyl-*N'*-methylacetimidamide with B_2pg_2

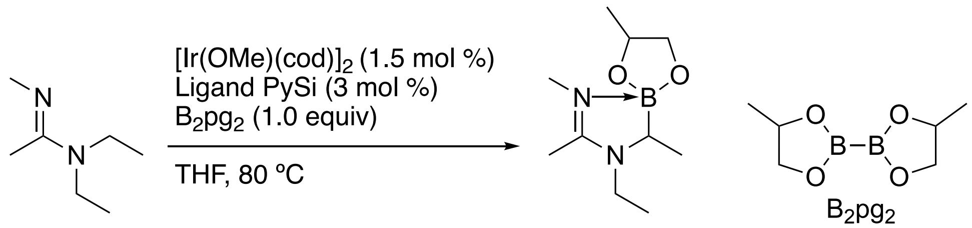

In a nitrogen filled glove box, a 5.0 mL conical vial was charged with $[Ir(OMe)cod]_2$ (10 mg, 1.5 mol %), ligand PySi (6.2 mg, 3.0 mol %), B_2pg_2 (170 mg, 1.0 mmol, 1.0 equiv) and (*E*)-*N,N*-diethyl-*N'*-methylacetimidamide (128 mg, 1.0 mmol, 1 equiv) in dry THF (3.0 mL). The vial was sealed, taken out of the glove box and stirred in a pre-heated aluminum block at 80 °C. After 1 h, an aliquot was removed and ^{1}H NMR of crude material was collected. 100% conversion of starting material was observed.

CHB of *(E)-N,N*-diethyl-*N'*-methylacetimidamide with B_2eg_2

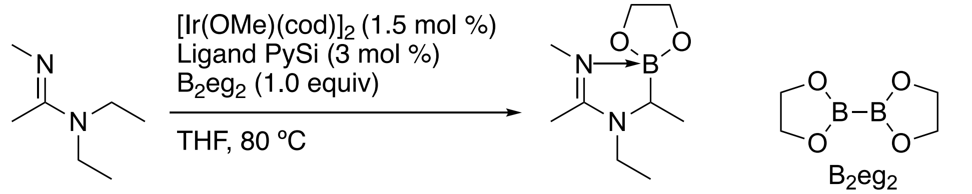

In a nitrogen filled glove box, a 5.0 mL conical vial was charged with $[Ir(OMe)cod]_2$ (10 mg, 1.5 mol %), ligand PySi (6.2 mg, 3.0 mol %), B_2eg_2 (142 mg, 1.0 mmol, 1.0 equiv) and (*E*)-*N,N*-diethyl-*N'*-methylacetimidamide (128 mg, 1.0 mmol, 1 equiv) in dry THF (3.0 mL). The vial was sealed, taken out of the glove box and stirred in a pre-heated aluminum block at 80 °C. After 1 h, an aliquot was removed and ^{1}H NMR of crude material was collected. 100% conversion of starting material was observed.

CHB of (*E*)-*N*,*N*-diethyl-*N'*-methylacetimidamide (4a)

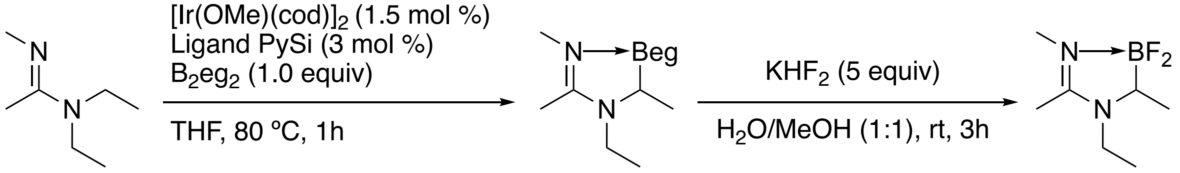

4a, 85% yield

In a nitrogen filled glove box, a 5.0 mL conical vial was charged with $[Ir(OMe)cod]_2$ (10 mg, 1.5 mol %), ligand PySi (6.2 mg, 3.0 mol %), B_2eg_2 (142 mg, 1.0 mmol, 1.0 equiv) and (*E*)-*N,N*-diethyl-*N'*-methylacetimidamide (128 mg, 1.0 mmol, 1 equiv) in dry THF (3.0 mL). The vial was sealed, taken out of the glove box and stirred in a pre-heated aluminum block at 80 °C. After 1 h, the mixture was concentrated under reduced pressure. Conversion to BF_2 molecule was followed by reported procedure.[16] Residue was transferred to a 100 mL flask charged with MeOH (10 mL), H_2O (10 mL) and KHF_2 (390.0 mg, 5.0 mmol) at room temperature. The resulting mixture was then allowed to stir at room temperature for 3h. After removal of the solvent, the residue was diluted with H_2O (30 mL) and extracted with EtOAc three times (3 X 30 mL). The combined organic phase was dried over $MgSO_4$. After removal of the solvent, the residue was purified by gradient column chromatography with activated basic alumina gel (ethyl acetate/methanol 100:0 → ethyl acetate/methanol 90:10). The fractions containing product were collected to yield 208 mg of **4a** as a white solid (85% yield, mp = 109-111 ºC).

^{1}H NMR (500 MHz, $CDCl_3$): δ 3.34 (m, 2H), 2.87 (s, 3H), 2.57 (m, 1H), 2.02 (s, 3H), 1.14 (t, J = 7.3 Hz, 3H), 1.07 (d, J = 7.2 Hz, 3H). ^{13}C NMR{^{1}H} (126 MHz, $CDCl_3$): δ 165.4, 39.0, 28.3, 14.1, 13.2 (t, J_{CF} = 4.8 Hz), 11.4. ^{11}B NMR (160 MHz, $CDCl_3$): δ 7.0. ^{19}F NMR (470 MHz, $CDCl_3$): δ -155.37, -165.60. HRMS (ESI) *m/z* calc for $C_7H_{15}BF_2N_2$ $[M-F]^+$ 157.1312, found 157.1314

CHB of (*E*)-*N*,*N*-diethyl-*N*'-methylpropionimidamide (4b)

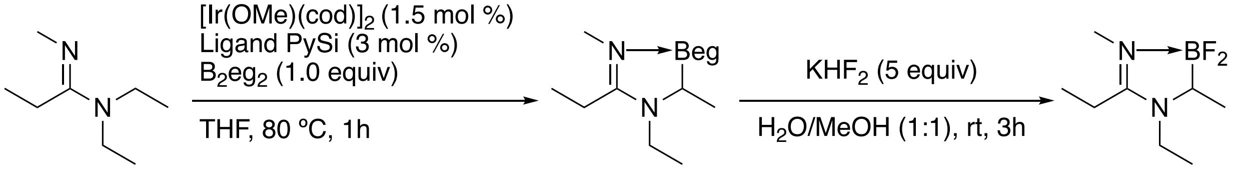

4b, 83% yield

In a nitrogen filled glove box, a 5.0 mL conical vial was charged with $[Ir(OMe)cod]_2$ (10 mg, 1.5 mol %), ligand PySi (6.2 mg, 3.0 mol %), B_2eg_2 (142 mg, 1.0 mmol, 1.0 equiv) and (*E*)-*N,N*-diethyl-*N*'-methylpropionimidamide (142 mg, 1.0 mmol, 1 equiv) in dry THF (3.0 mL). The vial was sealed, taken out of the glove box and stirred in a pre-heated aluminum block at 80 °C. After 1 h, the mixture was concentrated under reduced pressure. Conversion to BF_2 molecule was followed by reported procedure.[16] Residue was transferred to a 100 mL flask charged with MeOH (10 mL), H_2O (10 mL) and KHF_2 (390.0 mg, 5.0 mmol) at room temperature. The resulting mixture was then allowed to stir at room temperature for 3h. After removal of the solvent, the residue was diluted with H_2O (30 mL) and extracted with EtOAc three times (3 X 30 mL). The combined organic phase was dried over $MgSO_4$. After removal of the solvent, the residue was purified by gradient column chromatography with activated basic alumina gel (ethyl acetate/methanol 100:0 → ethyl acetate/methanol 90:10). The fractions containing product were collected to yield 157 mg of **4b** as a white solid (83% yield, mp = 113-115 ºC).

^{1}H NMR (500 MHz, $CDCl_3$); δ 3.34 (m, 2H), 2.90 (s, 3H), 2.60 (m, 1H), 2.40 (m, 2H), 1.18 (t, *J* = 7.8 Hz, 3H), 1.17 (t, *J* = 7.2 Hz, 3H), 1.09 (d, *J* = 7.3 Hz, 3H). ^{13}C NMR{^{1}H} (126 MHz, $CDCl_3$): δ 169.2, 38.4, 27.8, 18.2, 14.3, 13.1 (t, J_{CF} = 4.9 Hz), 10.2. ^{11}B NMR (160 MHz, $CDCl_3$): δ 7.1. ^{19}F NMR (470 MHz, $CDCl_3$): δ -155.61, -165.86. HRMS (ESI) *m/z* calc for $C_8H_{17}BF_2N_2$ $[M-F]^+$ 171.1468, found 171.1482

CHB of (*E*)-*N*,*N*,*N'*-triethylacetimidamide (4c)

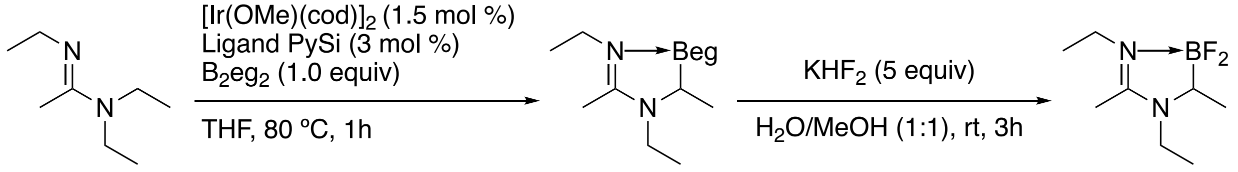

4c, 81% yield

In a nitrogen filled glove box, a 5.0 mL conical vial was charged with [Ir(OMe)cod]$_2$ (10 mg, 1.5 mol %), ligand PySi (6.2 mg, 3.0 mol %), B_2eg_2 (142 mg, 1.0 mmol, 1.0 equiv) and (*E*)-*N,N,N'*-triethylacetimidamide (142 mg, 1.0 mmol, 1 equiv) in dry THF (3.0 mL). The vial was sealed, taken out of the glove box and stirred in a pre-heated aluminum block at 80 °C. After 1 h, the mixture was concentrated under reduced pressure. Conversion to BF_2 molecule was followed by reported procedure.[16] Residue was transferred to a 100 mL flask charged with MeOH (10 mL), H_2O (10 mL) and KHF_2 (390.0 mg, 5.0 mmol) at room temperature. The resulting mixture was then allowed to stir at room temperature for 3h. After removal of the solvent, the residue was diluted with H_2O (30 mL) and extracted with EtOAc three times (3 X 30 mL). The combined organic phase was dried over $MgSO_4$. After removal of the solvent, the residue was purified by gradient column chromatography with activated basic alumina gel (ethyl acetate/methanol 100:0 → ethyl acetate/methanol 90:10). The fractions containing product were collected to yield 153 mg of **4c** as a white solid (81% yield, mp = 116-118 ºC).

^{1}H NMR (500 MHz, $CDCl_3$); δ 3.35 (m, 2H), 3.30 (q, *J* = 7.1 Hz, 2H), 2.57 (m, 1H), 2.04 (s, 3H), 1.21 (t, *J* = 7.4 Hz, 3H), 1.15 (t, *J* = 7.3 Hz, 3H), 1.09 (d, *J* = 7.3 Hz, 3H). ^{13}C NMR{^{1}H} (126 MHz, $CDCl_3$): δ 164.8, 39.0, 37.3, 29.9, 16.1, 14.1, 13.3 (t, J_{CF} = 4.8 Hz), 11.2. ^{11}B NMR (160 MHz, $CDCl_3$): δ 7.4. ^{19}F NMR (470 MHz, $CDCl_3$): δ -152.30, -163.23. HRMS (ESI) *m/z* calc for $C_8H_{17}BF_2N_2$ [M-F]$^+$ 171.1468, found 171.1470

CHB of (*E*)-*N,N,N′*-triethylpropionimidamide (4d)

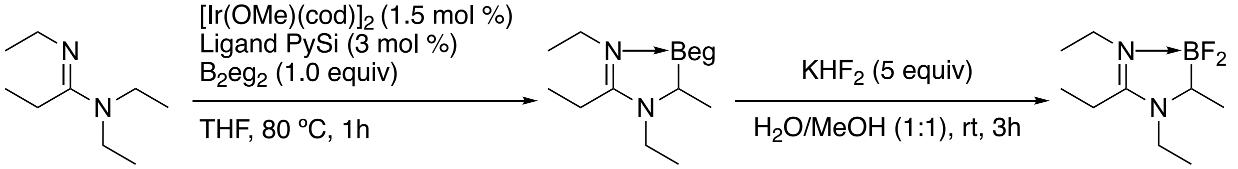

4d, 80% yield

In a nitrogen filled glove box, a 5.0 mL conical vial was charged with [Ir(OMe)cod]$_2$ (10 mg, 1.5 mol %), ligand PySi (6.2 mg, 3.0 mol %), B_2eg_2 (142 mg, 1.0 mmol, 1.0 equiv) and (*E*)-*N,N,N'*-triethylacetimidamide (142 mg, 1.0 mmol, 1 equiv) in dry THF (3.0 mL). The vial was sealed, taken out of the glove box and stirred in a pre-heated aluminum block at 80 °C. After 1 h, the mixture was concentrated under reduced pressure. Conversion to BF_2 molecule was followed by reported procedure.[16] Residue was transferred to a 100 mL flask charged with MeOH (10 mL), H_2O (10 mL) and KHF_2 (390.0 mg, 5.0 mmol) at room temperature. The resulting mixture was then allowed to stir at room temperature for 3h. After removal of the solvent, the residue was diluted with H_2O (30 mL) and extracted with EtOAc three times (3 X 30 mL). The combined organic phase was dried over $MgSO_4$. After removal of the solvent, the residue was purified by gradient column chromatography with activated basic alumina gel (ethyl acetate/methanol 100:0 → ethyl acetate/methanol 90:10). The fractions containing product were collected to yield 163 mg of **4d** as a white solid (80% yield, mp = 123-125 ºC).

^{1}H NMR (500 MHz, $CDCl_3$); δ 3.35 (m, 2H), 3.30 (q, *J* = 7.3 Hz, 2H), 2.57 (m, 1H), 2.39 (qd, J = 7.3, 5.0 Hz, 2H), 1.25 (t, *J* = 7.7 Hz, 3H), 1.19 (t, *J* = 7.7 Hz, 3H), 1.17 (t, *J* = 7.0 Hz, 3H), 1.06 (d, *J* = 7.3 Hz, 3H). ^{13}C NMR{^{1}H} (126 MHz, $CDCl_3$): δ 168.8, 38.3, 37.1, 29.9, 18.1, 16.4, 14.1, 13.0 (t, J_{CF} = 4.8 Hz), 11.1. ^{11}B NMR (160 MHz, $CDCl_3$): δ 7.5. ^{19}F NMR (470 MHz, $CDCl_3$): δ -151.75, -162.77. HRMS (ESI) *m/z* calc for $C_9H_{19}BF_2N_2$ [M-F]$^+$ 185.1625, found 185.1632

CHB of (*E*)-*N*-ethyl-1-(piperidin-1-yl)ethan-1-imine (4e)

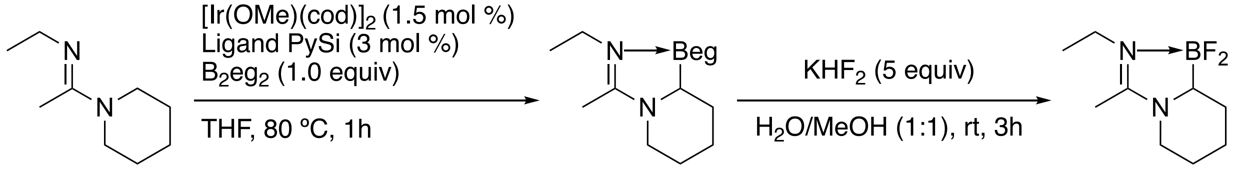

4e, 86% yield

In a nitrogen filled glove box, a 5.0 mL conical vial was charged with $[Ir(OMe)cod]_2$ (10 mg, 1.5 mol %), ligand PySi (6.2 mg, 3.0 mol %), B_2eg_2 (142 mg, 1.0 mmol, 1.0 equiv) and (*E*)-*N*-ethyl-1-(piperdin-1-yl)ethan-1-imine (154 mg, 1.0 mmol, 1 equiv) in dry THF (3.0 mL). The vial was sealed, taken out of the glove box and stirred in a pre-heated aluminum block at 80 °C. After 1 h, the mixture was concentrated under reduced pressure. Conversion to BF_2 molecule was followed by reported procedure.[16] Residue was transferred to a 100 mL flask charged with MeOH (10 mL), H_2O (10 mL) and KHF_2 (390.0 mg, 5.0 mmol) at room temperature. The resulting mixture was then allowed to stir at room temperature for 3h. After removal of the solvent, the residue was diluted with H_2O (30 mL) and extracted with EtOAc three times (3 X 30 mL). The combined organic phase was dried over $MgSO_4$. After removal of the solvent, the residue was purified by gradient column chromatography with activated basic alumina gel (ethyl acetate/methanol 100:0 → ethyl acetate/methanol 90:10). The fractions containing product were collected to yield 173 mg of **4e** as a white solid (86% yield, mp = 114-116 ºC).

^{1}H NMR (500 MHz, $CDCl_3$); δ 3.67 (m, 1H), 3.30 (q, J = 7.3 Hz, 2H), 3.02 (m, 1H), 2.30 (m, 1H), 2.01 (s, 3H), 1.85 (m, 1H), 1.74 (m, 2H), 1.37 (m, 3H), 1.21 (t, J = 7.3 Hz, 3H). ^{13}C NMR{^{1}H} (126 MHz, $CDCl_3$): δ 162.9, 45.2, 37.2, 26.7, 26.3 (t, J_{CF} = 4.2 Hz), 24.8, 16.2, 10.6. ^{11}B NMR (160 MHz, $CDCl_3$): δ 7.3. ^{19}F NMR (470 MHz, $CDCl_3$): δ -153.55, -161.72. HRMS (ESI) *m/z* calc for $C_9H_{17}BF_2N_2$ $[M-F]^+$ 183.1468, found 183.1459

CHB of *N,N*-diethyl-3,4-dihydro-2*H*-pyrrol-5-amine (4f)

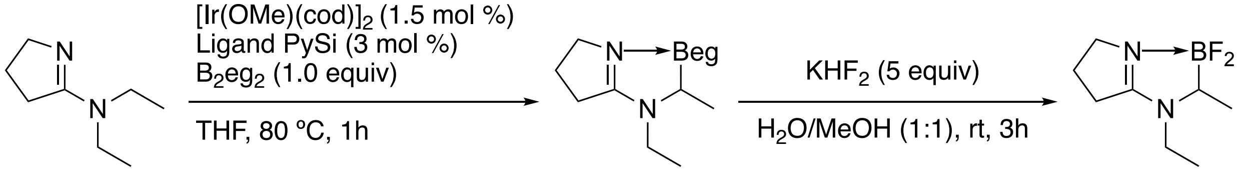

4f, 83% yield

In a nitrogen filled glove box, a 5.0 mL conical vial was charged with $[Ir(OMe)cod]_2$ (10 mg, 1.5 mol %), ligand PySi (6.2 mg, 3.0 mol %), B_2eg_2 (142 mg, 1.0 mmol, 1.0 equiv) and *N,N*-diethyl-3,4-dihydro-2*H*-pyrrol-5-amine (140 mg, 1.0 mmol, 1 equiv) in dry THF (3.0 mL). The vial was sealed, taken out of the glove box and stirred in a pre-heated aluminum block at 80 °C. After 1 h, the mixture was concentrated under reduced pressure. Conversion to BF_2 molecule was followed by reported procedure.[16] Residue was transferred to a 100 mL flask charged with MeOH (10 mL), H_2O (10 mL) and KHF_2 (390.0 mg, 5.0 mmol) at room temperature. The resulting mixture was then allowed to stir at room temperature for 3h. After removal of the solvent, the residue was diluted with H_2O (30 mL) and extracted with EtOAc three times (3 X 30 mL). The combined organic phase was dried over $MgSO_4$. After removal of the solvent, the residue was purified by gradient column chromatography with activated basic alumina gel (ethyl acetate/methanol 100:0 → ethyl acetate/methanol 90:10). The fractions containing product were collected to yield 156 mg of **4f** as a white solid (83% yield, mp = 85-87 ºC).

^1H NMR (500 MHz, $CDCl_3$); δ 3.56 (m, 2H), 3.30 (m, 1H), 3.21 (m, 1H), 2.93 (m, 1H), 2.54 (m, 2H), 2.32 (m, 2H), 1.15 (t, J = 7.4 Hz, 3H), 1.11 (d, J = 7.2 Hz, 3H). ^{13}C NMR{^1H} (126 MHz, $CDCl_3$): δ 174.4, 44.1, 39.5, 25.6, 24.6, 13.9, 13.0 (t, J_{CF} = 4.7 Hz). ^{11}B NMR (160 MHz, $CDCl_3$): δ 6.0. ^{19}F NMR (470 MHz, $CDCl_3$): δ -154.06, -163.09. HRMS (ESI) *m/z* calc for $C_8H_{15}BF_2N_2$ $[M-F]^+$ 169.1312, found 169.1315

CHB of *N,N*-diethyl-3,4,5,6-tetrahydropyridin-2-amine (4g)

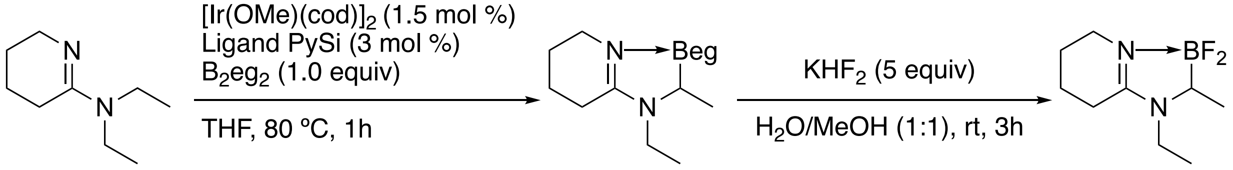

4g, 82% yield

In a nitrogen filled glove box, a 5.0 mL conical vial was charged with $[Ir(OMe)cod]_2$ (10 mg, 1.5 mol %), ligand PySi (6.2 mg, 3.0 mol %), B_2eg_2 (142 mg, 1.0 mmol, 1.0 equiv) and *N,N*-diethyl-3,4,5,6-tetrahydropyridin-2-amine (154 mg, 1.0 mmol, 1 equiv) in dry THF (3.0 mL). The vial was sealed, taken out of the glove box and stirred in a pre-heated aluminum block at 80 °C. After 1 h, the mixture was concentrated under reduced pressure. Conversion to BF_2 molecule was followed by reported procedure.[16] Residue was transferred to a 100 mL flask charged with MeOH (10 mL), H_2O (10 mL) and KHF_2 (390.0 mg, 5.0 mmol) at room temperature. The resulting mixture was then allowed to stir at room temperature for 3h. After removal of the solvent, the residue was diluted with H_2O (30 mL) and extracted with EtOAc three times (3 X 30 mL). The combined organic phase was dried over $MgSO_4$. After removal of the solvent, the residue was purified by gradient column chromatography with activated basic alumina gel (ethyl acetate/methanol 100:0 → ethyl acetate/methanol 90:10). The fractions containing product were collected to yield 165 mg of **4g** as a white solid (82% yield, mp = 130-132 ºC).

^{1}H NMR (500 MHz, $CDCl_3$); δ 3.33 (m, 2H), 3.26 (m, 2H), 2.58 (m, 1H), 2.35 (m, 2H), 1.75 (m, 4H), 1.12 (t, J = 7.3 Hz, 3H), 1.07 (d, J = 7.1 Hz, 3H). ^{13}C NMR{^{1}H} (126 MHz, $CDCl_3$): δ 164.3, 38.9, 37.5, 22.7, 21.8, 19.2, 13.7, 13.0 (t, J_{CF} = 4.8 Hz). ^{11}B NMR (160 MHz, $CDCl_3$): δ 7.1. ^{19}F NMR (470 MHz, $CDCl_3$): δ -156.21, -165.95. HRMS (ESI) *m/z* calc for $C_9H_{17}BF_2N_2$ $[M-F]^+$ 183.1468, found 183.1469

CHB of N,N-diethyl-3,4,5,6-tetrahydro-2H-azepin-7-amine (4h)

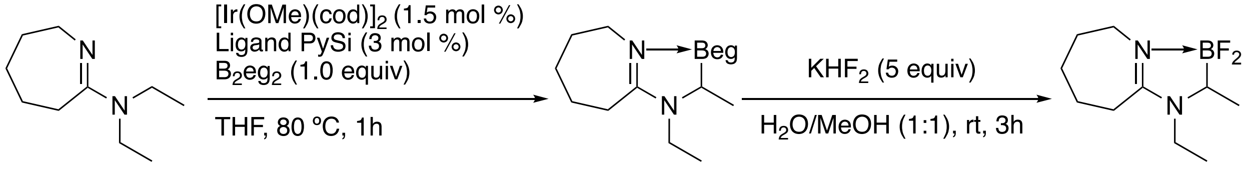

4h, 75% yield

In a nitrogen filled glove box, a 5.0 mL conical vial was charged with $[Ir(OMe)cod]_2$ (10 mg, 1.5 mol %), ligand PySi (6.2 mg, 3.0 mol %), B_2eg_2 (142 mg, 1.0 mmol, 1.0 equiv) and *N,N-diethyl-3,4,5,6-tetrahydro-2H-azepin-7-amine* (168 mg, 1.0 mmol, 1 equiv) in dry THF (3.0 mL). The vial was sealed, taken out of the glove box and stirred in a pre-heated aluminum block at 80 °C. After 1 h, the mixture was concentrated under reduced pressure. Conversion to BF_2 molecule was followed by reported procedure.[16] Residue was transferred to a 100 mL flask charged with MeOH (10 mL), H_2O (10 mL) and KHF_2 (390.0 mg, 5.0 mmol) at room temperature. The resulting mixture was then allowed to stir at room temperature for 3h. After removal of the solvent, the residue was diluted with H_2O (30 mL) and extracted with EtOAc three times (3 X 30 mL). The combined organic phase was dried over $MgSO_4$. After removal of the solvent, the residue was purified by gradient column chromatography with activated basic alumina gel (ethyl acetate/methanol 100:0 → ethyl acetate/methanol 90:10). The fractions containing product and tetraethyl urea (10%) were collected to yield 162.9 mg of **4h** as a white solid as determined by ^1H (75% yield of 4h, mp = 126-128 ºC).

^1H NMR (500 MHz, $CDCl_3$); δ 3.35 (m, 4H), 2.58 (m, 1H), 2.50 (m, 2H), 1.78 (m, 2H), 1.64 (m, 4H), 1.15 (t, J = 7.3 Hz, 3H), 1.10 (d, J = 7.1 Hz, 3H). ^{13}C NMR{^1H} (126 MHz, $CDCl_3$): δ 171.1, 42.5, 39.4, 30.8, 28.8, 25.9, 23.8, 15.3, 13.6 (t, J_{CF} = 5.1 Hz). ^{11}B NMR (160 MHz, $CDCl_3$): δ 6.9. ^{19}F NMR (470 MHz, $CDCl_3$): δ -154.21, -165.48. HRMS (ESI) *m/z* calc for $C_{10}H_{19}BF_2N_2$ $[M-F]^+$ 197.1625, found 197.1616

CHB of (*E*)-*N*-ethyl-1-morpholinoethan-1-imine (4i)

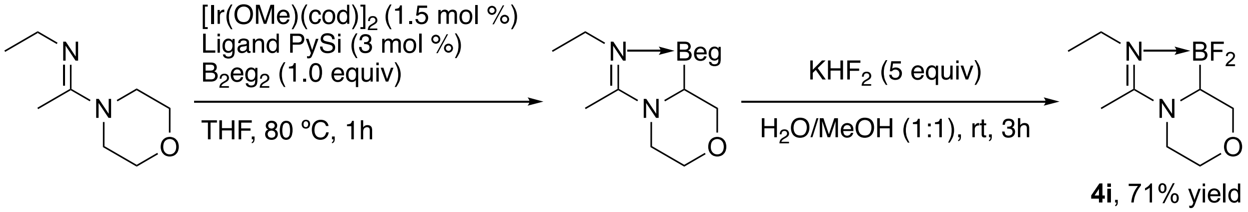

In a nitrogen filled glove box, a 5.0 mL conical vial was charged with $[Ir(OMe)cod]_2$ (10 mg, 1.5 mol %), ligand PySi (6.2 mg, 3.0 mol %), B_2eg_2 (142 mg, 1.0 mmol, 1.0 equiv) and (*E*)-*N*-ethyl-1-morpholinoethan-1-imine (156 mg, 1.0 mmol, 1 equiv) in dry THF (3.0 mL). The vial was sealed, taken out of the glove box and stirred in a pre-heated aluminum block at 80 °C. After 1 h, the mixture was concentrated under reduced pressure. Conversion to BF_2 molecule was followed by reported procedure.[16] Residue was transferred to a 100 mL flask charged with MeOH (10 mL), H_2O (10 mL) and KHF_2 (390.0 mg, 5.0 mmol) at room temperature. The resulting mixture was then allowed to stir at room temperature for 3h. After removal of the solvent, the residue was diluted with H_2O (30 mL) and extracted with EtOAc three times (3 X 30 mL). The combined organic phase was dried over $MgSO_4$. After removal of the solvent, the residue was purified by gradient column chromatography with activated basic alumina gel (ethyl acetate/methanol 100:0 → ethyl acetate/methanol 90:10). The fractions containing product were collected to yield 144 mg of **4i** as a white solid (71% yield, mp = 117-119 ºC).

^{1}H NMR (500 MHz, $CDCl_3$); δ 4.05 (dd, *J* = 8.0, 4.3 Hz, 1H), 3.93 (m, 1H), 3.68 (m, 1H), 3.38 (m, 2H), 3.31 (q, J = 7.2 Hz, 2H), 3.27 (m, 1H), 2.69 (m, 1H), 2.06 (s, 3H), 1.22 (t, *J* = 7.5 Hz, 3H). ^{13}C NMR{^{1}H} (126 MHz, $CDCl_3$): δ 163.5, 69.3, 66.8, 66.4, 47.4, 44.7, 37.3, 31.8, 29.9, 16.0, 14.3, 10.6. ^{11}B NMR (160 MHz, $CDCl_3$): δ 6.9. ^{19}F NMR (470 MHz, $CDCl_3$): δ -152.15, -160.74. HRMS (ESI) *m/z* calc for $C_8H_{15}BF_2N_2O$ $[M-F]^+$ 185.1261, found 185.1270

Synthesis of (*E*)-*N*-((difluoroboraneyl)methyl)-*N*,*N'*-dimethylacetimidamide (4j)

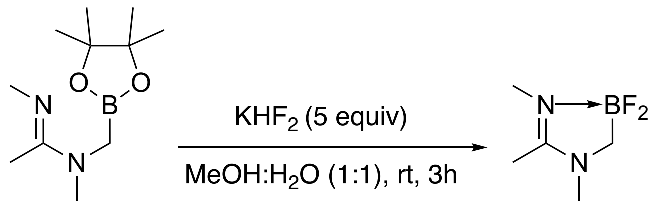

4i, 85% yield

To a 100 mL flask charged with **2a** (226.0 mg, 1.0 mmol), MeOH (10 mL), H_2O (10 mL) and KHF_2 (390.0 mg, 5.0 mmol) at room temperature. The resulting mixture was then allowed to stir at room temperature for 3h. After removal of the solvent, the residue was diluted with H_2O (30 mL) and extracted with EtOAc three times (3 X 30 mL). The combined organic phase was dried over $MgSO_4$. After removal of the solvent, the residue was purified by gradient column chromatography with basic alumina gel (ethyl acetate/methanol 100:0 → ethyl acetate/methanol 90:10). The fractions containing product were collected to yield 125 mg of **4i** as a white solid (mp = 102-104 ºC) (85% yield).

^{1}H NMR (500 MHz, $CDCl_3$): δ 3.02 (s, 3H), 2.88 (s, 3H), 2.40 (t, J = 11.7 Hz, 2H), 2.02 (s, 3H). ^{13}C NMR{^{1}H} (126 MHz, $CDCl_3$): δ 166.4, 36.3, 28.4, 11.3, 1.2. ^{11}B NMR (160 MHz, $CDCl_3$): δ 7.1. ^{19}F NMR (470 MHz, $CDCl_3$): δ -155.3. HRMS (ESI) *m/z* calc for $C_5H_{11}BFN_2$ $[M-F]^+$ 129.0999, found 129.1000

REFERENCES

1) Uson, R.; Oro, L. A.; Cabeza, J. A.; Bryndza, H. E.; Stepro, M. P. Dinuclear Methoxy, Cyclooctadiene, and Barrelene Complexes of Rhodium(I) and Iridium(I). In *Inorganic Syntheses*; John Wiley & Sons, Inc.: Hoboken, NJ, USA, 2007; 126.

2) Dannatt, J. E., Yadav, A., Smith III, M. R., & Maleczka Jr, R. E. Amide directed iridium C(sp^3)–H borylation catalysis with high N-methyl selectivity. *Tetrahedron* **2022**, *109*, 132578.

3) Perrin, C.L., Nunez O. Hydrolysis of unsymmetrical acetamidines: leaving abilities and stereoelectronic effects *J. Am. Chem. Soc.* **1987** *109*, 522.

4) Haug, E.; Kantlehner, W.; Speh, P.; Brauner, H. Eine einfache Synthese fur N,N,N'-trisubstituierte Amidine *Synthesis* **1983**, *1*, 35-37.

5) Takahide, F. Nao, N.; Takuma, O.; Ilhyong, R. Free-radical-mediated [2+2+1] cycloaddition of acetylene, amidines, and CO leading to five-membered A,B-unsaturated lactams *J. Am. Chem. Soc.* **2013** *135*, 1006-1008.

6) Raczynska, E. D.; Decouzon, M.; Gal, J. F.; Maria, P. C.; Gelbard, G.; Vielfaure-Joly, F. Gas-phase structural (internal) effects in strong organic nitrogen bases *J. Phys. Org. Chem.* **2001**, *14*, 25-34.

7) Jaroszewska-Manaj, J., Oszczapowicz, J., Makulski, W. Amidines. Part 41. Effects of substitution at the amidino carbon atom and at the iminonitrogen atom on the preferred configuration at the C-N bond in the 13C NMR spectra of *N,N*-dimethyl-*N*-alkylamidines *Perkin Trans 2* **2001**, *7*, 1186.

8) Eicher, T., Rohde, R. Zur Reaktion von Cyclopropenonen mit Azomethinen; VII. Diphenylcyclopropenon und cyclische Amidine: Synthese von biund tricyclischen Pyrrolinon-Derivaten *Synthesis* **1985**, *6*, 619.

9) Williams, S., Qi, L., Cox, R. J., Kumar, P., Xiao, J., Hydrogenation of functionalised pyridines with a rhodium oxide catalyst under mild conditions *Org. Biomol. Chem.* **2024**, *22*, 1010-1017.

10) Yao, T. Facile N-arylation of amidines and N,N-disubstituted amidines *Tet. Lett.* **2015**, *56*, 4623.

11) Komatsu, M.; Niishikaze, N.; Sakamoto, M.; Ohshiro, Y.; Agawa, T. Reaction of diaziridines with diphenylketene and isocyanates *J. Org. Chem.* **1974**, *39*, 3198.

12) Oxley, P.; Short, W. F. 305. Amidines. Part IX. Preparation of substituted amidines from ketoxime sulphonates and ammonia or amines *J. Chem. Soc.* **1948**, 1514-1522.

13) Flosser, D. A.; Olofson, R. A. Improved Synthesis of Dialkylaminopyrrolines *Synthetic Communications* **2003**, *33*, 2045-2052.

14) Freifelder, M.; Mattoon, R. W.; Ng, Y. H. Hydrogenation in the Pyridine Series. II. Catalytic Reduction of 2-Monoalkyl- and 2-Dialkylaminopyridines *J. Org. Chem.* **1964**, *29 (12)*, 3730-3732.

15) Fornwald, R. M.; Yadav, A.; Bastidas, J. R.; Smith, M. R., III; Maleczka, R. E., Jr. Simple and Green Preparation of Tetraalkoxydiborons and Diboron Diolates from tetrahydroxydiboron *J. Org. Chem.* **2024***, 89,* 6048-6052.

16) Chen, L.; Yang, Y.; Liu, L.; Gao, Q.; Xu, S Iridium-Catalyzed Enantioselective α-C(sp^3) –H Borylation of Azacycles *J. Am. Chem. Soc.* **2020**, *142*, 28, 12062-12068.

NMR SPECTRUM

^{1}H NMR of (*E*)-*N*'-(sec-butyl)-*N*,*N*-dimethylacetimidamide (1a) ($CDCl_3$, 500 MHz)

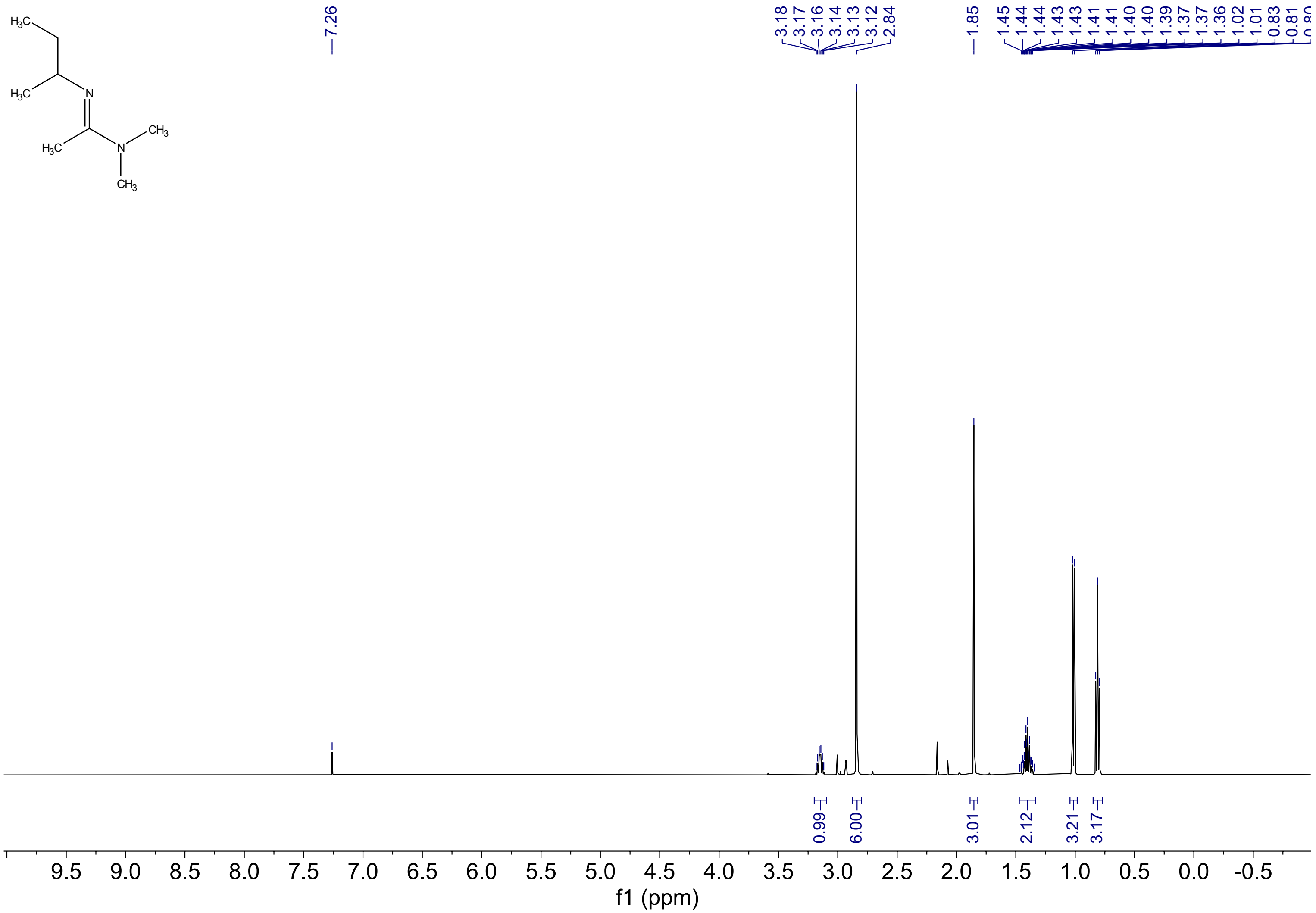

$^{13}C\{^1H\}$ NMR of (*E*)-*N*'-(sec-butyl)-*N*,*N*-dimethylacetimidamide (1a) ($CDCl_3$, 126 MHz)

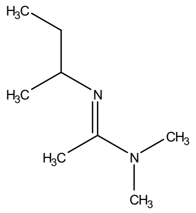

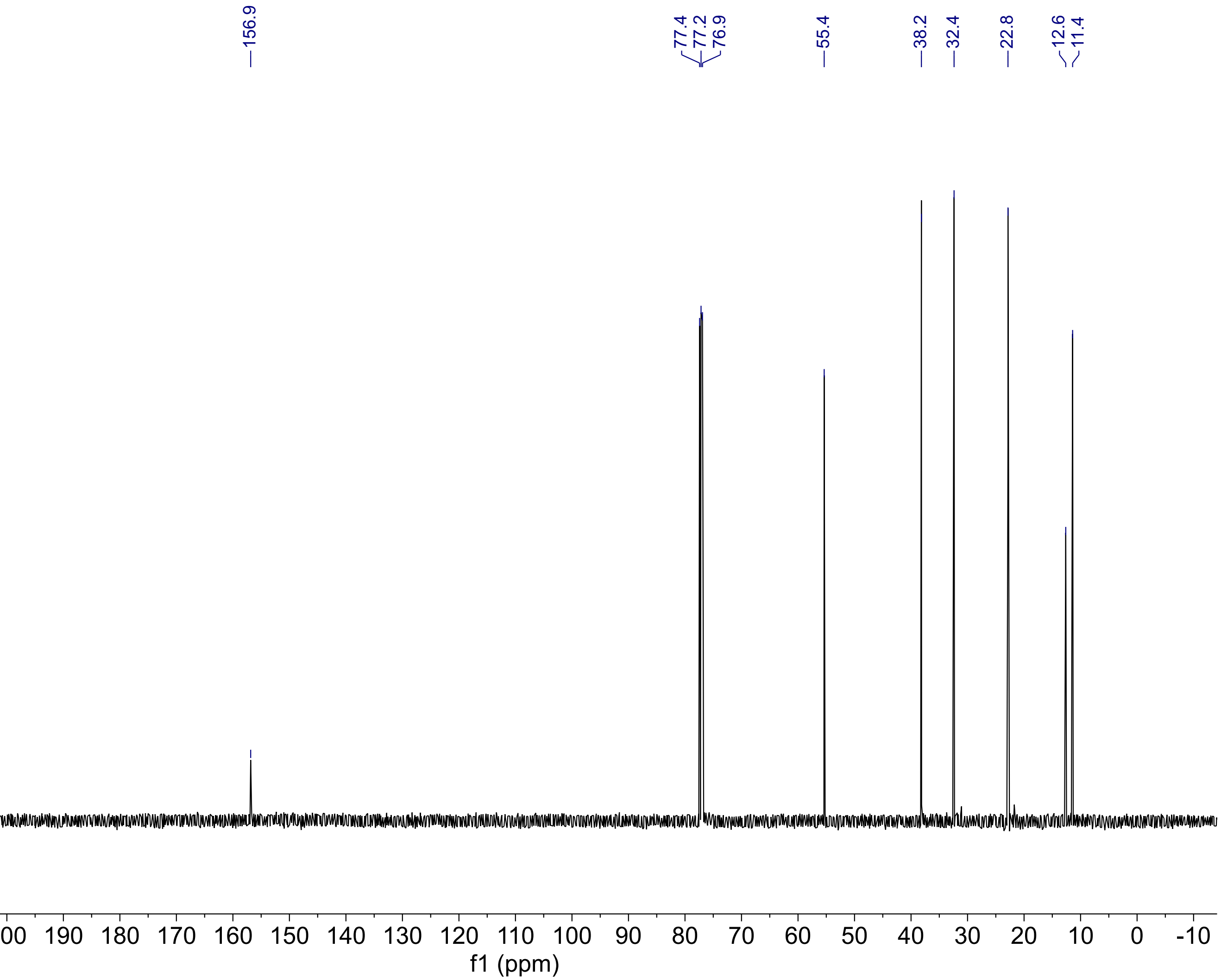

^{1}H NMR of (*E*)-*N*,*N*,*N'*-trimethylacetimidamide (1b) ($CDCl_3$, 500 MHz)

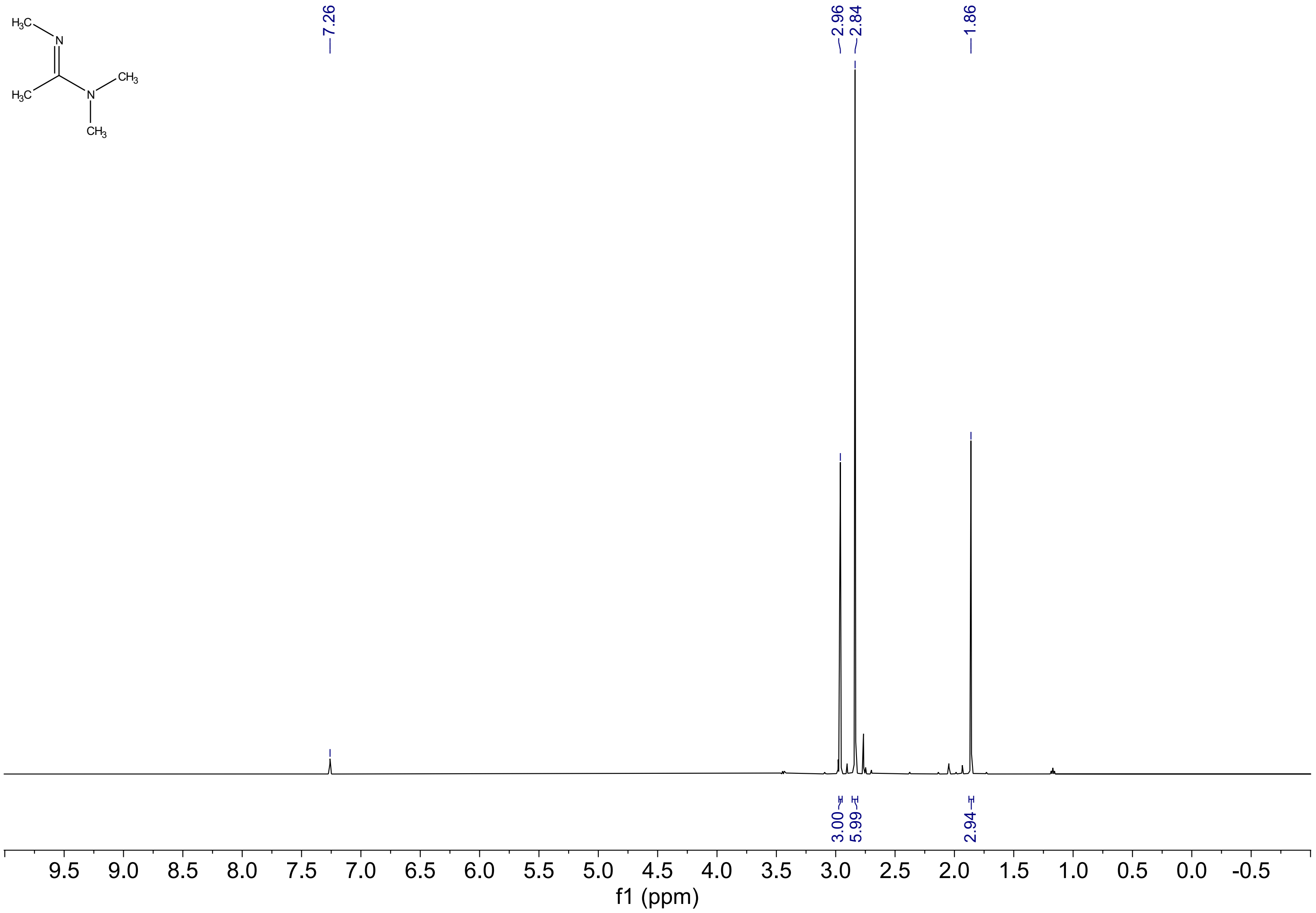

$^{13}C\{^{1}H\}$ NMR of (*E*)-*N*,*N*,*N'*-trimethylacetimidamide (1b) ($CDCl_3$, 126 MHz)

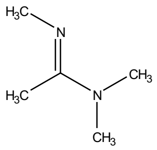

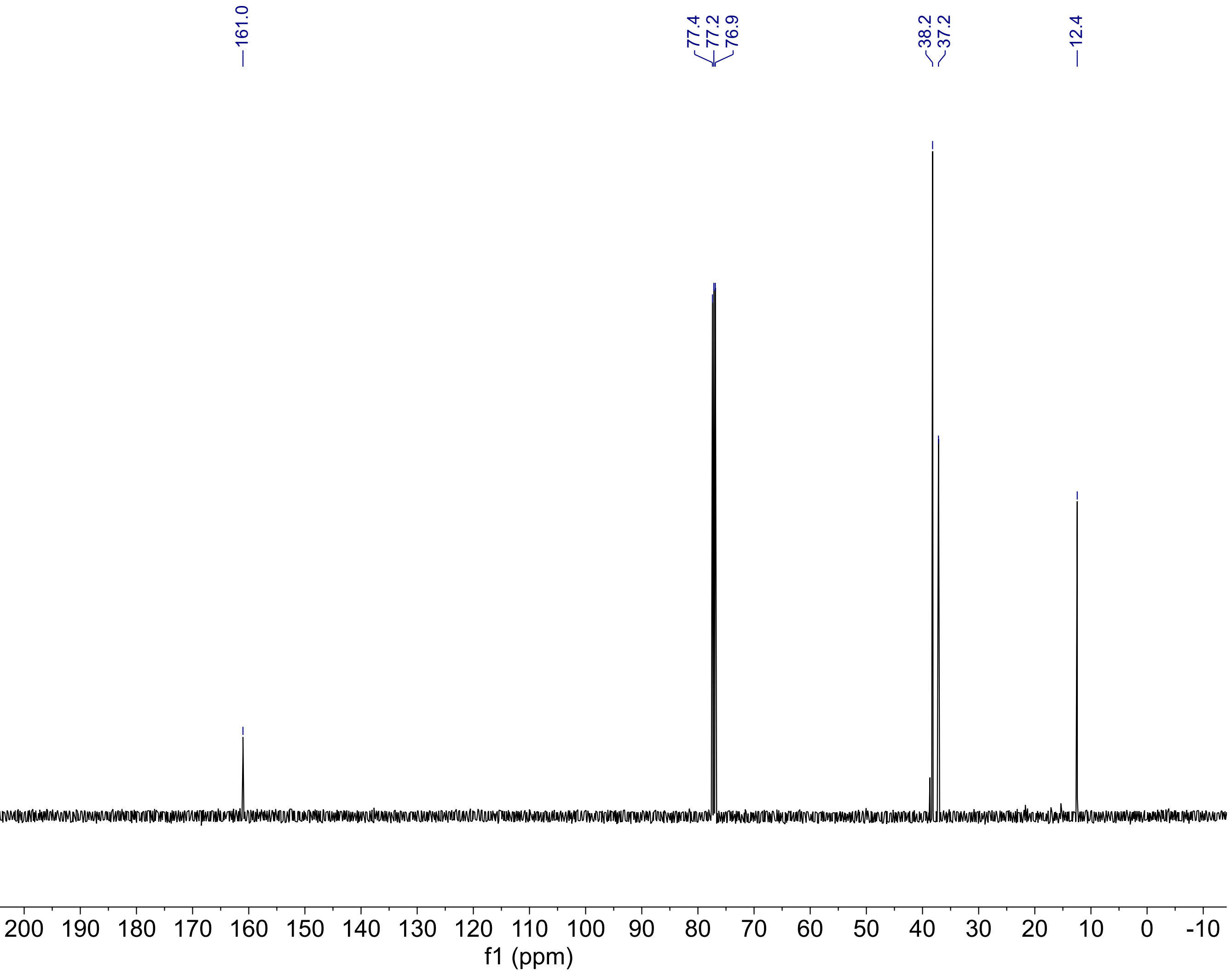

^{1}H NMR of (*E*)-*N'-ethyl-N*,*N*-dimethylformimidamide (1c) ($CDCl_3$, 500 MHz)

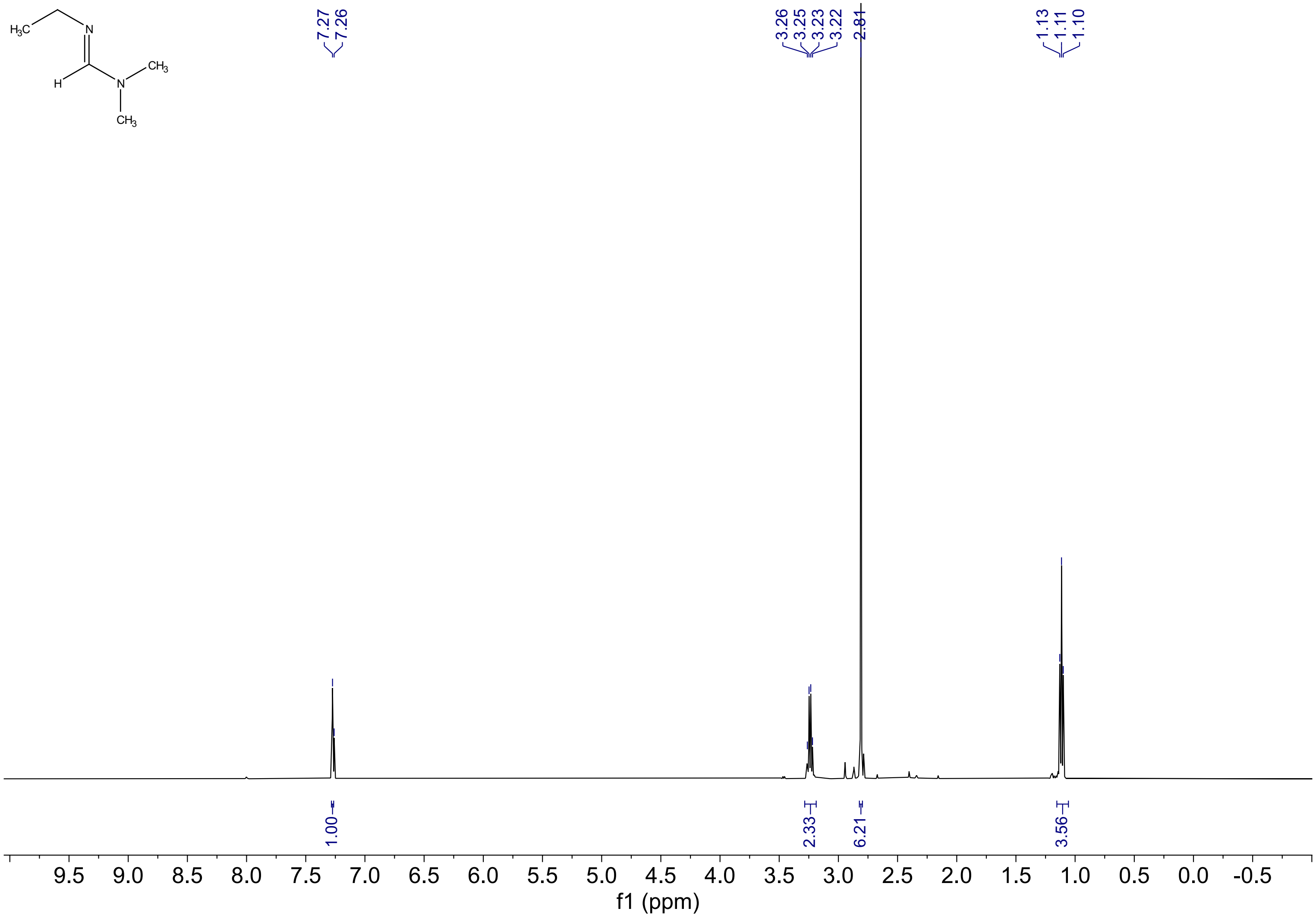

$^{13}C\{^{1}H\}$ NMR of (*E*)-*N'-ethyl-N*,*N*-dimethylformimidamide (1c) ($CDCl_3$, 126 MHz)

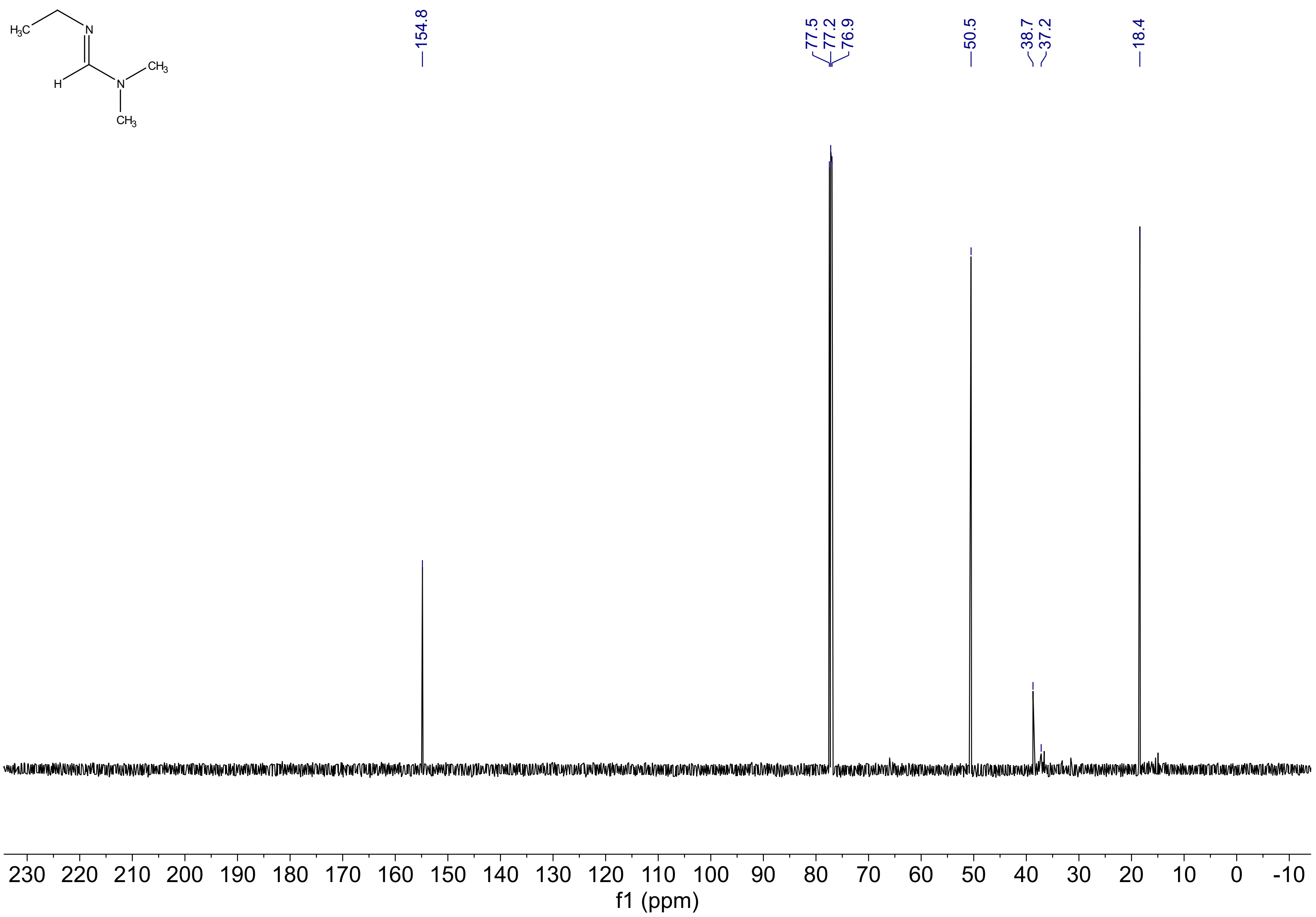

^{1}H NMR of (*E*)-*N*,*N*,*N’*-trimethylpropionimidamide (1d) ($CDCl_3$, 126 MHz)

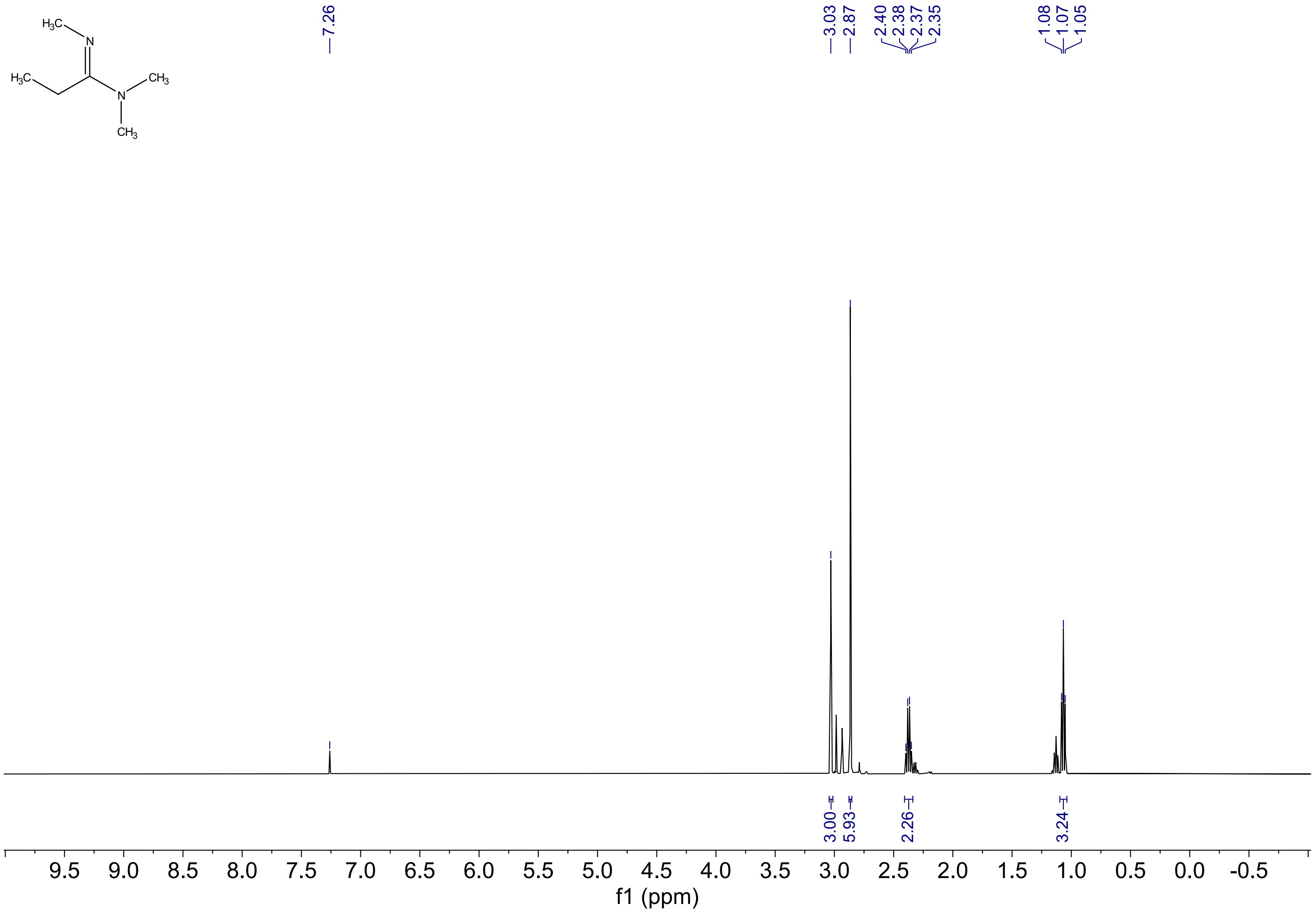

$^{13}C\{^{1}H\}$ NMR of (*E*)-*N*,*N*,*N'*-trimethylpropionimidamide (1d) ($CDCl_3$, 126 MHz)

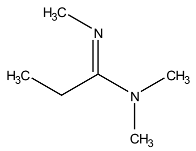

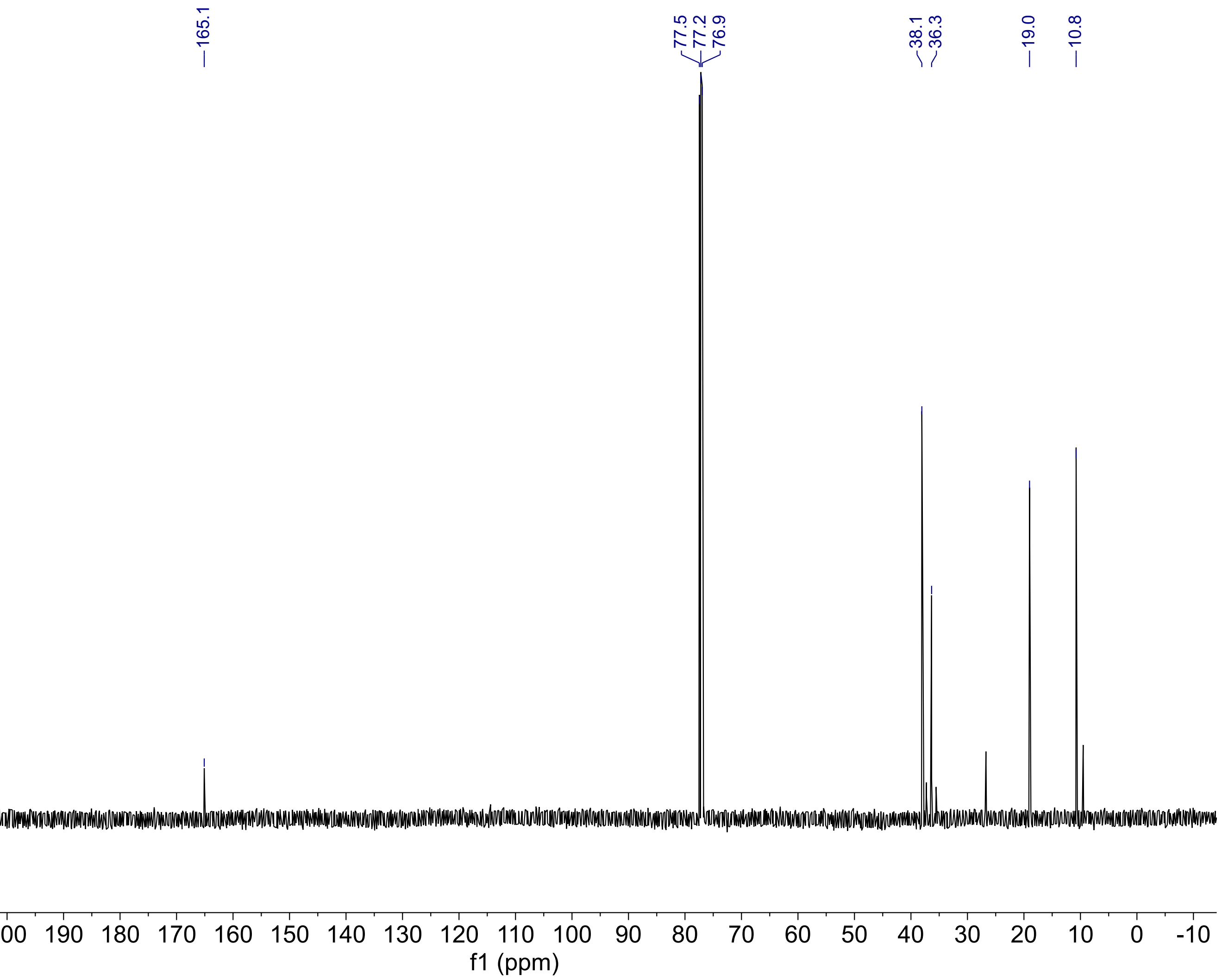

^{1}H NMR of (*E*)-*N*'-ethyl-*N*,*N*-dimethylacetimidamide (1e) ($CDCl_3$, 500 MHz)

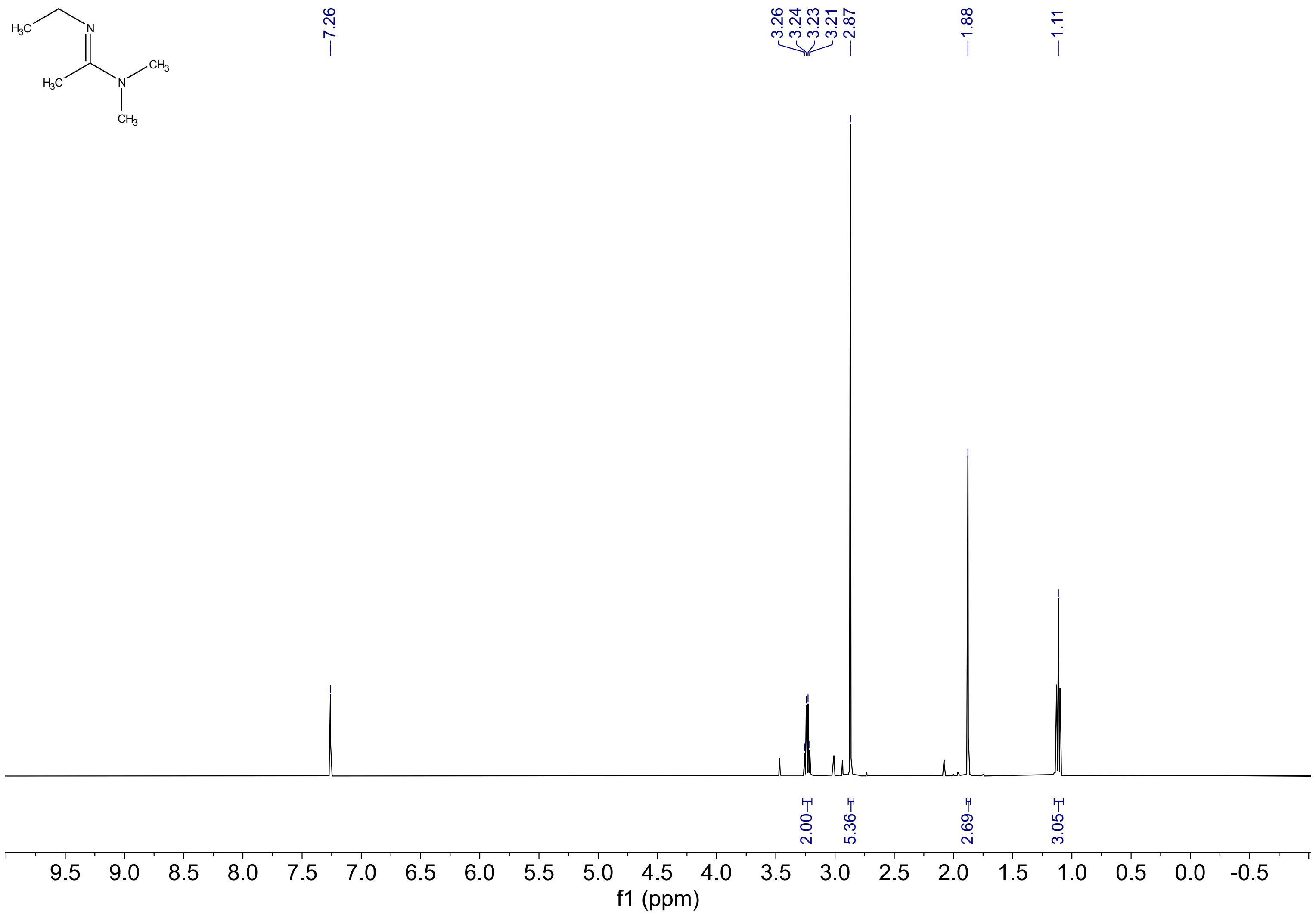

$^{13}C\{^1H\}$ NMR of (*E*)-*N*'-ethyl-*N*,*N*-dimethylacetimidamide (1e) ($CDCl_3$, 126 MHz)

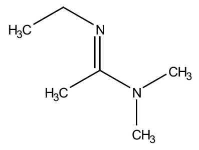

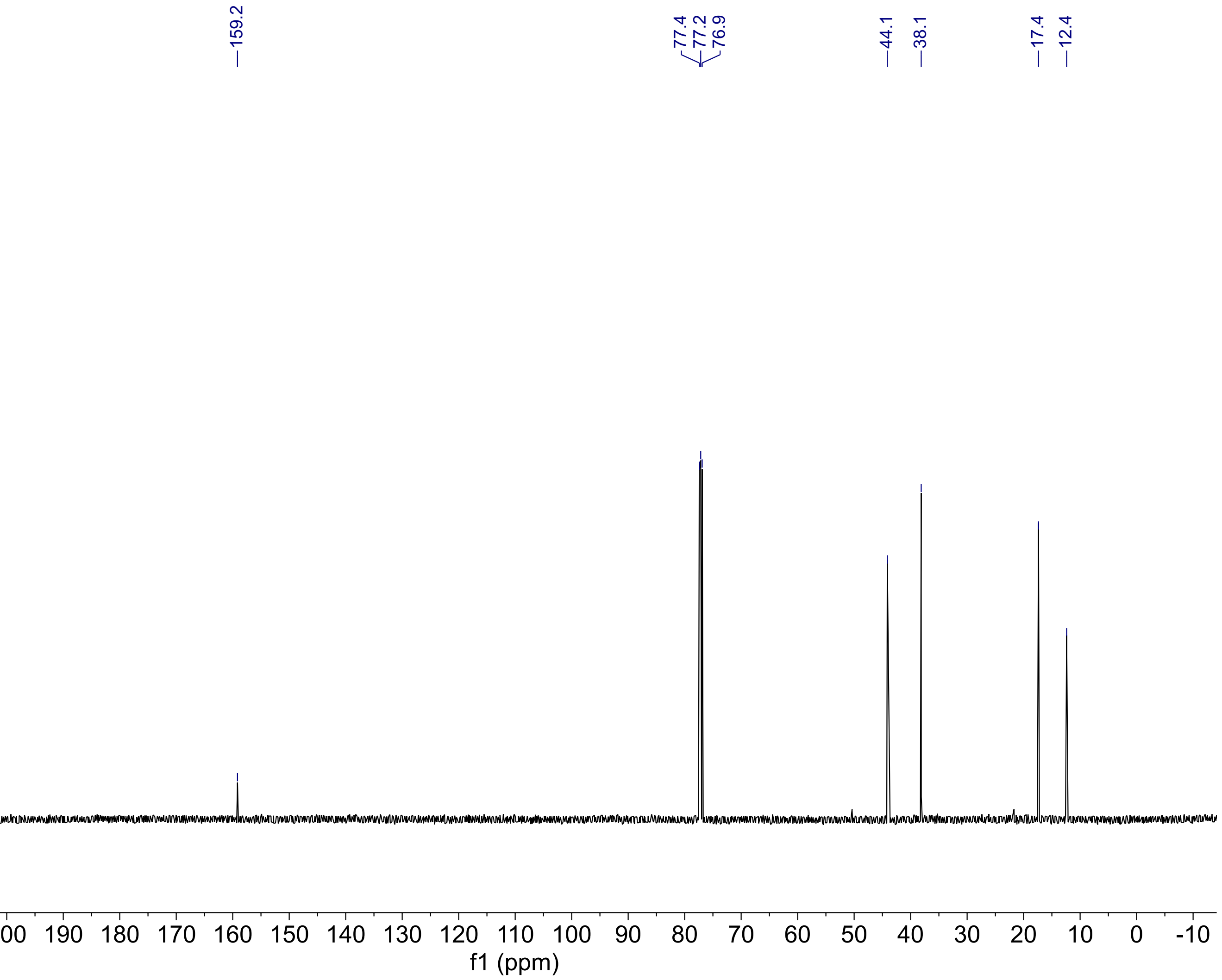

^{1}H NMR of (*E*)-*N*'-(isopropyl)-*N*,*N*-dimethylacetimidamide (1f) ($CDCl_3$, 500 MHz)

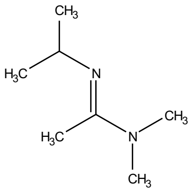

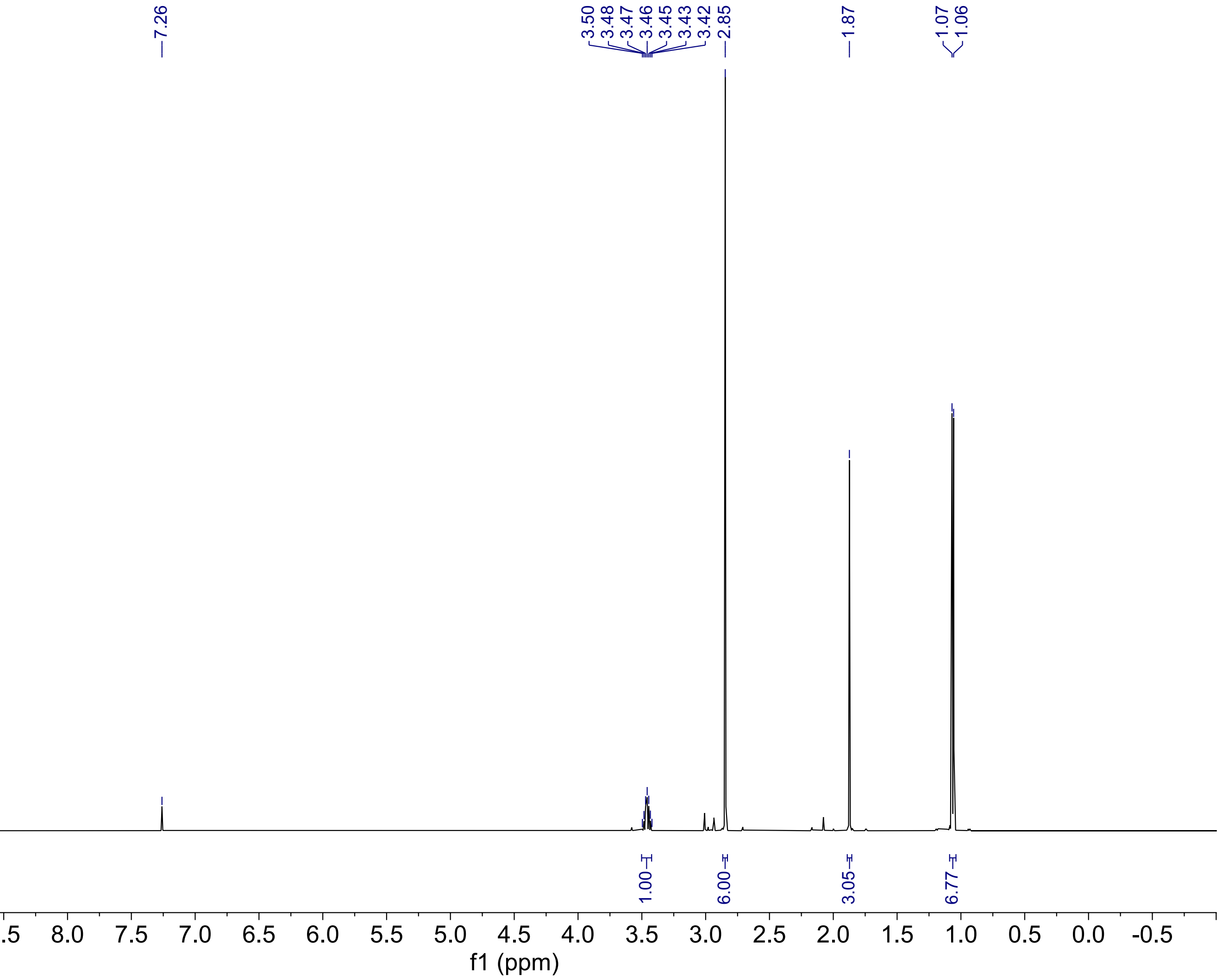

$^{13}C\{^1H\}$ NMR of (*E*)-*N*'-(isopropyl)-*N*,*N*-dimethylacetimidamide (1f) ($CDCl_3$, 126 MHz)

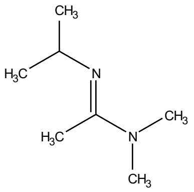

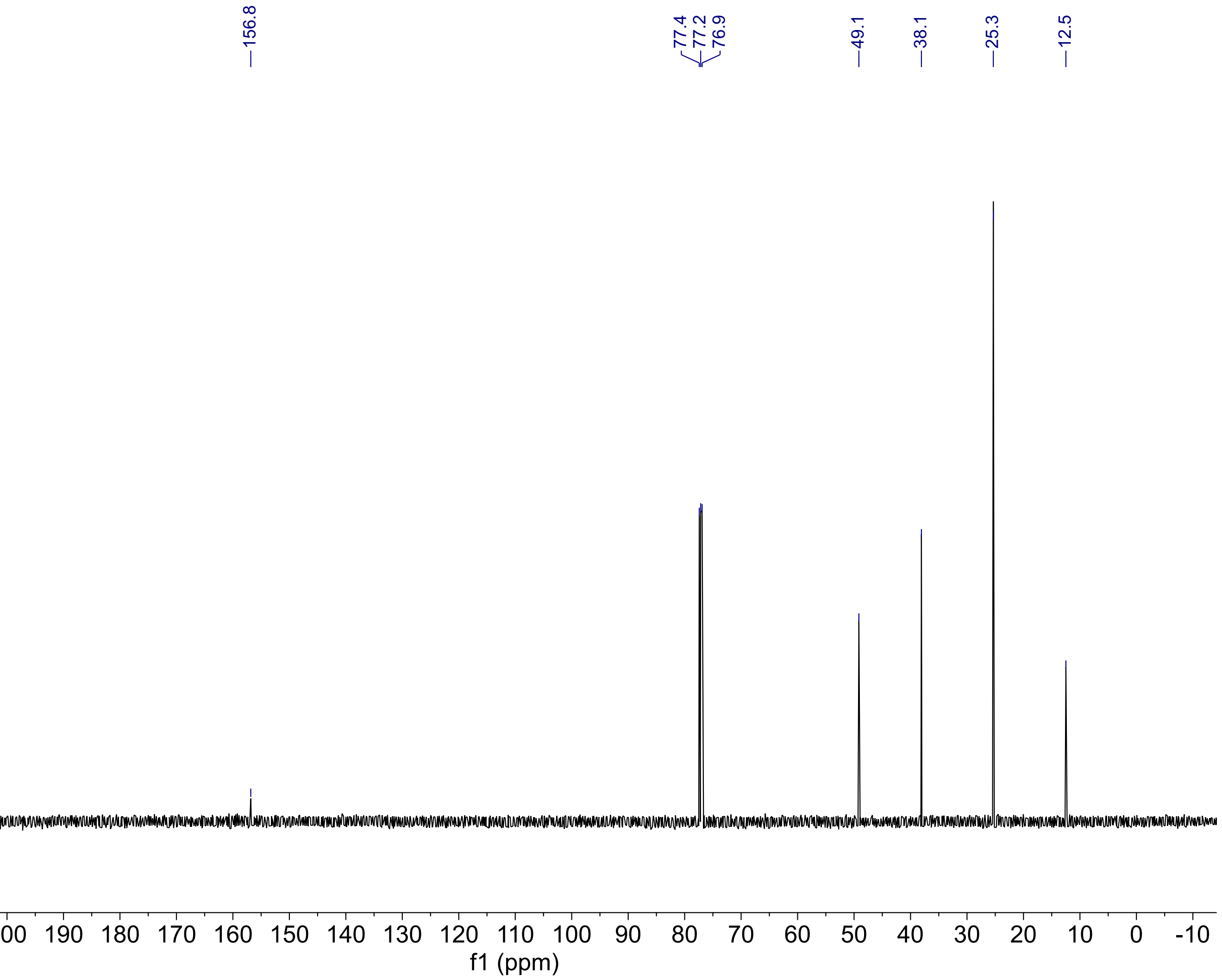

^{1}H NMR of (*E*)-*N*'-(tert-butyl)-*N*,*N*-dimethylacetimidamide (1g) ($CDCl_3$, 500 MHz)

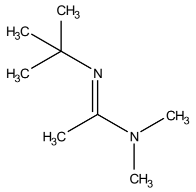

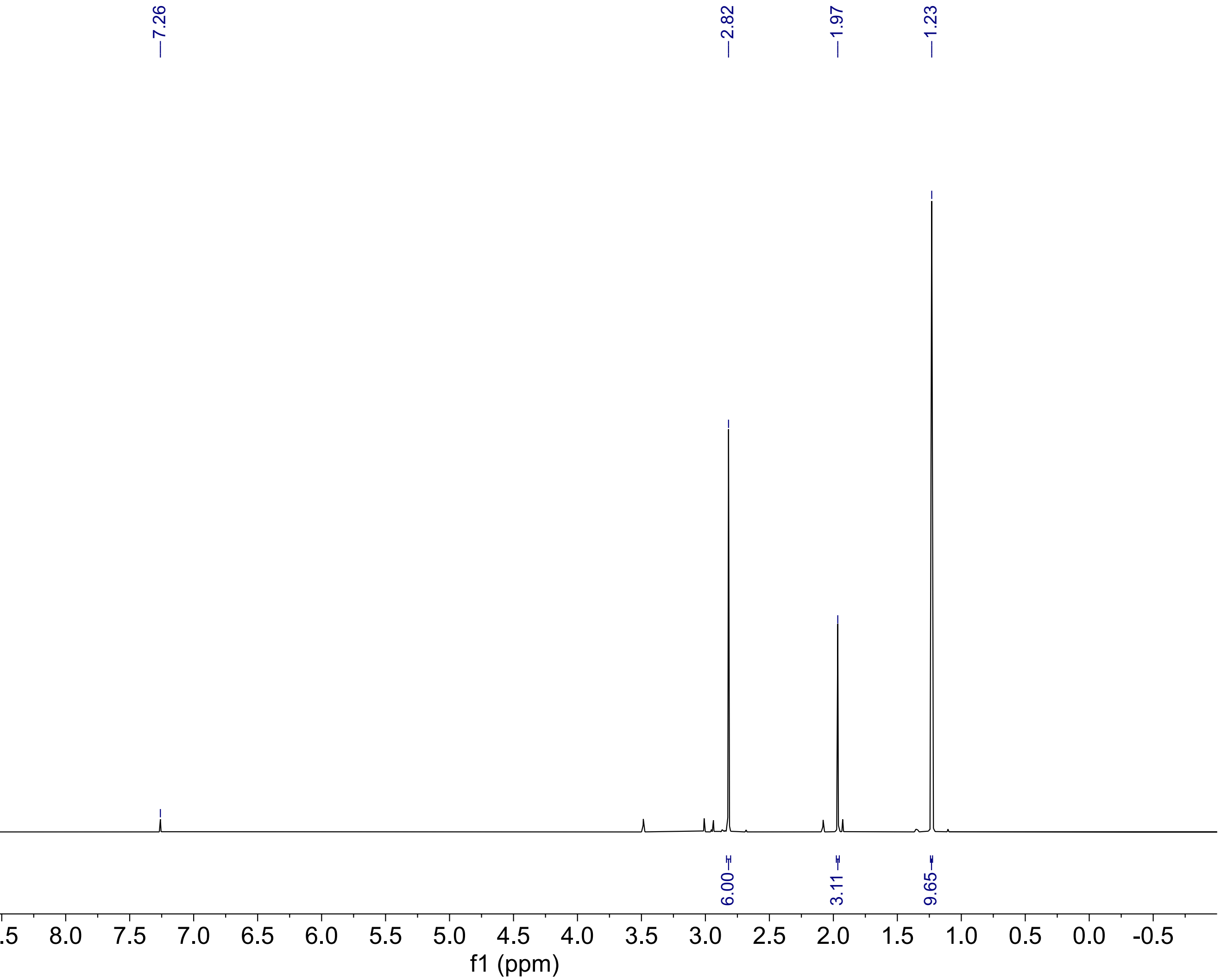

$^{13}C\{^1H\}$ NMR of (*E*)-*N*'-(tert-butyl)-*N*,*N*-dimethylacetimidamide (1g) ($CDCl_3$, 126 MHz)

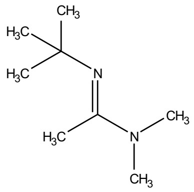

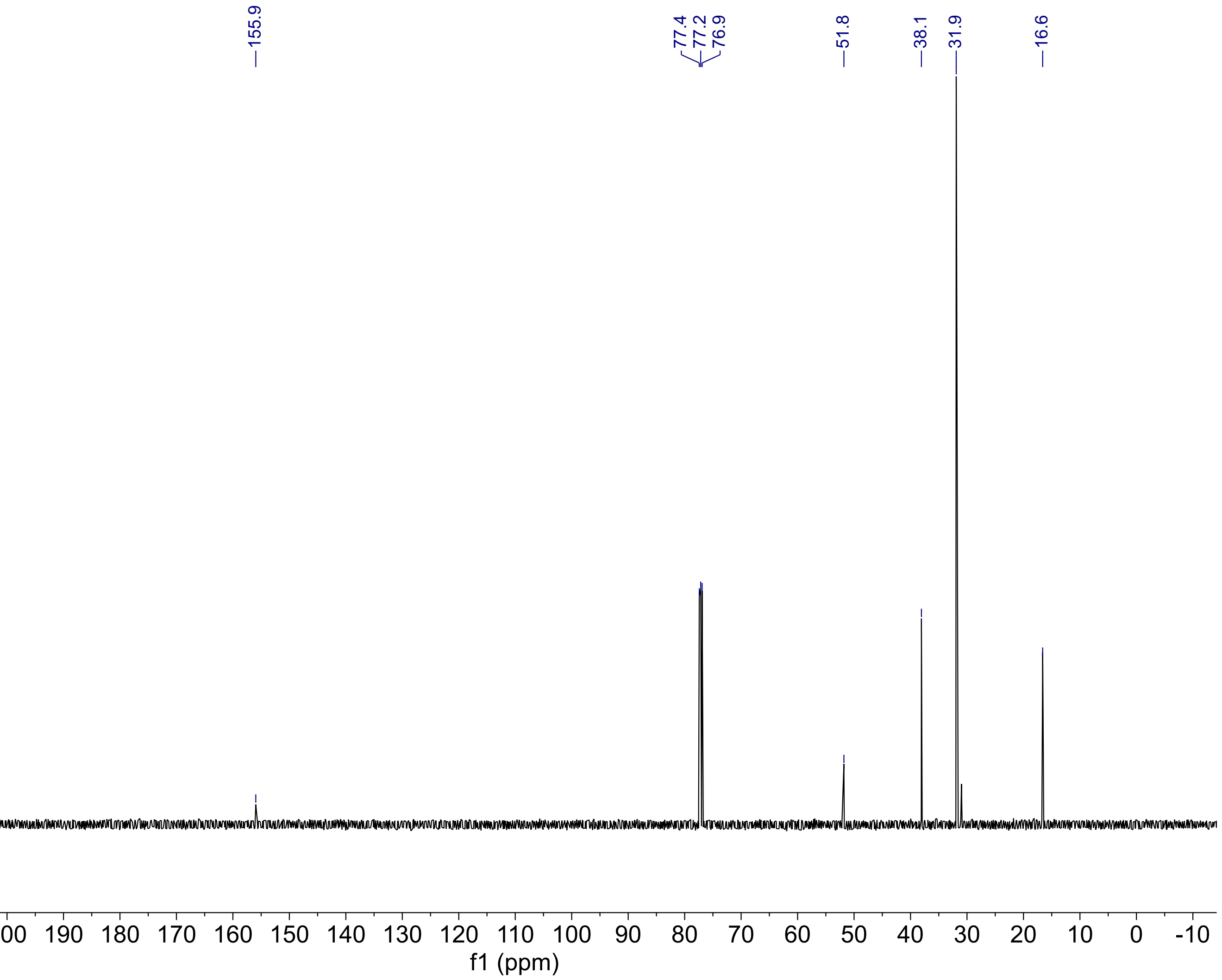

^{1}H NMR of (*E*)-*N*'-isobutyl-*N*,*N*-dimethylacetimidamide (1h) ($CDCl_3$, 500 MHz)

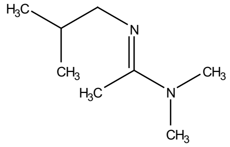

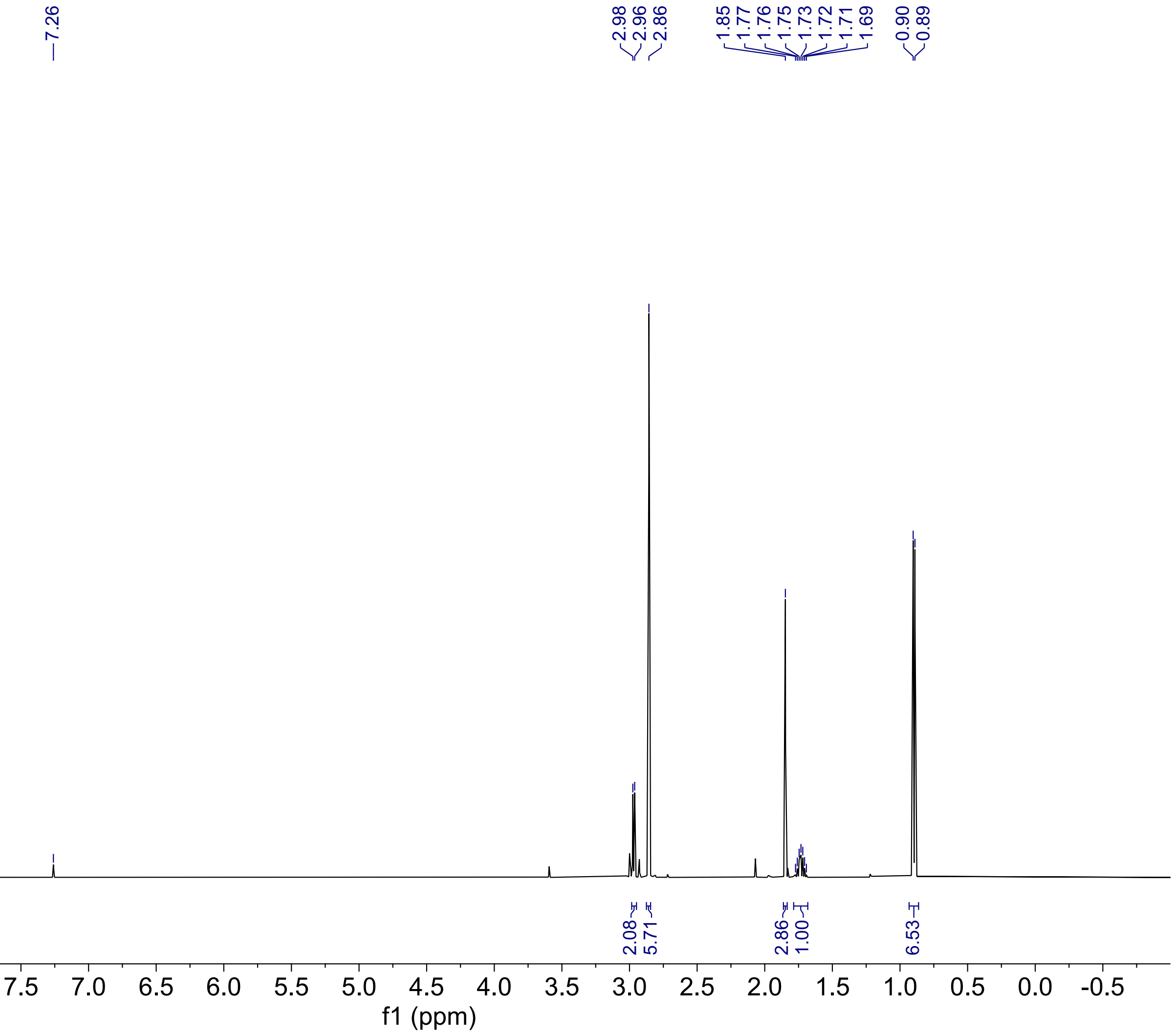

$^{13}C\{^1H\}$ NMR of (*E*)-*N*'-isobutyl-*N*,*N*-dimethylacetimidamide (1h) ($CDCl_3$, 126 MHz)

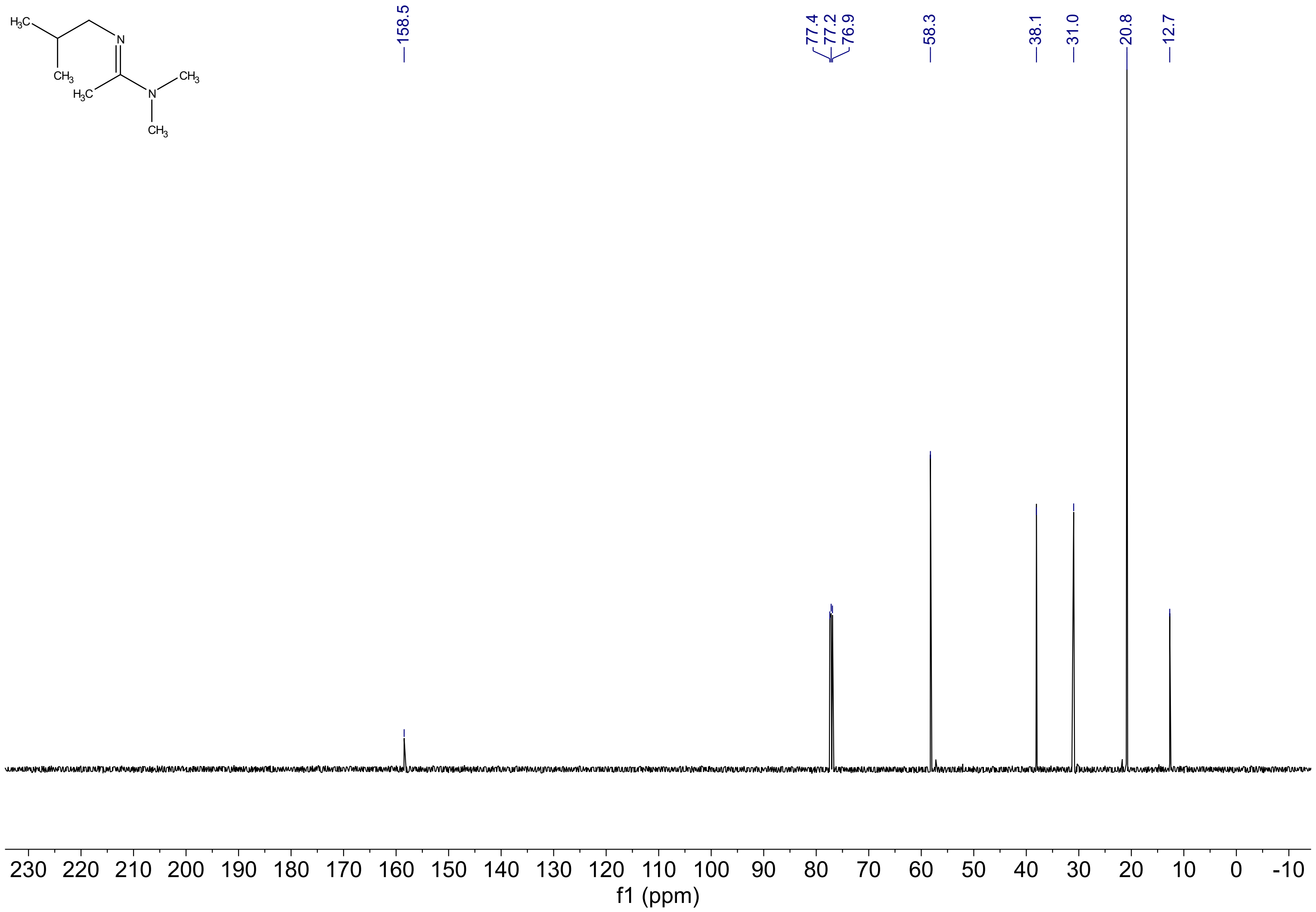

^{1}H NMR of *N,N*-dimethyl-3,4-dihydro-2*H*-pyrrol-5-amine (1i) ($CDCl_3$, 500 MHz)

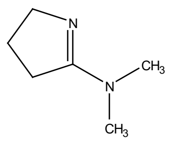

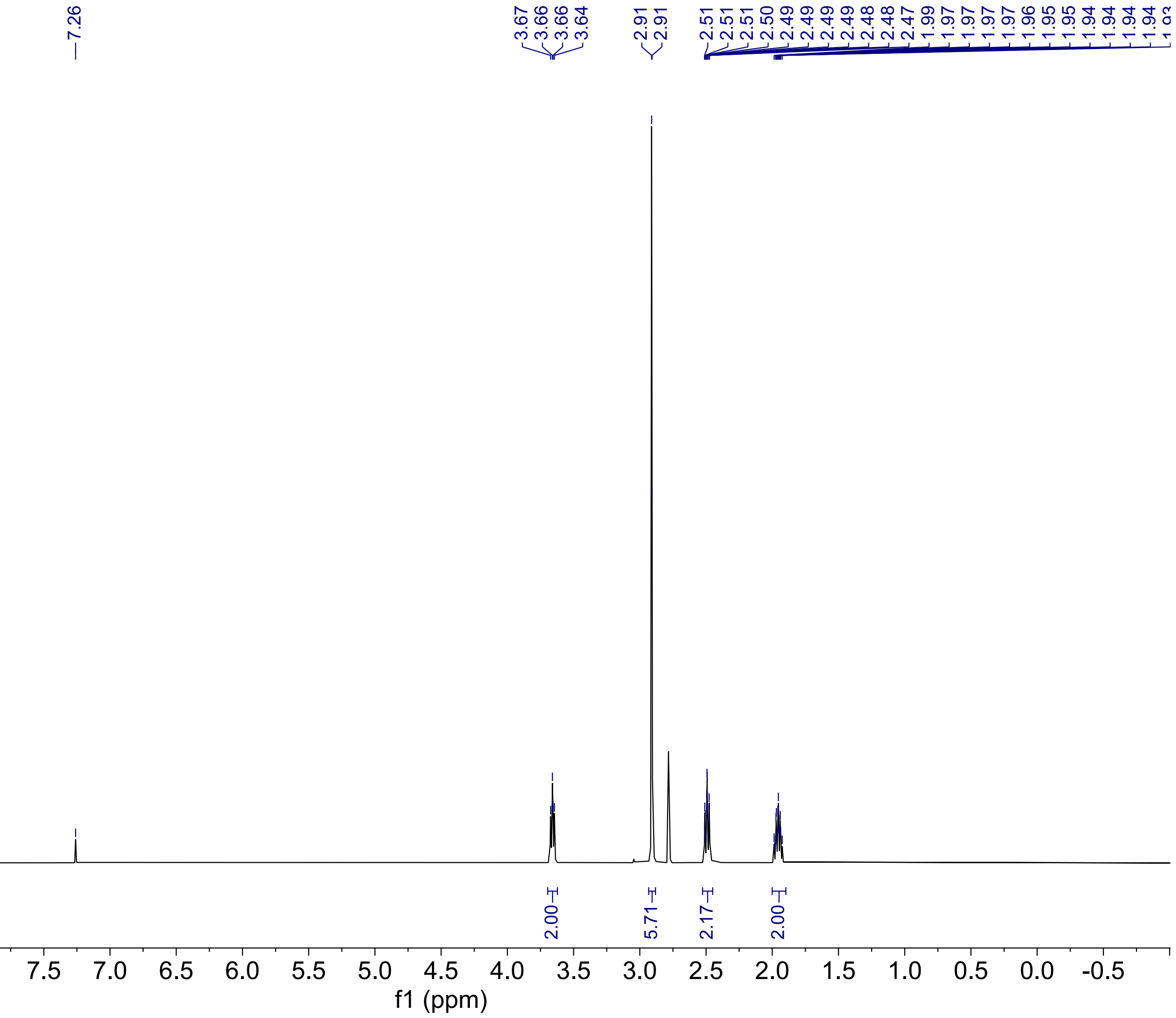

$^{13}C\{^{1}H\}$ NMR of *N,N*-dimethyl-3,4-dihydro-2*H*-pyrrol-5-amine (1i) ($CDCl_3$, 126 MHz)

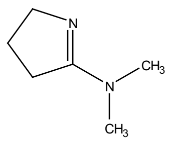

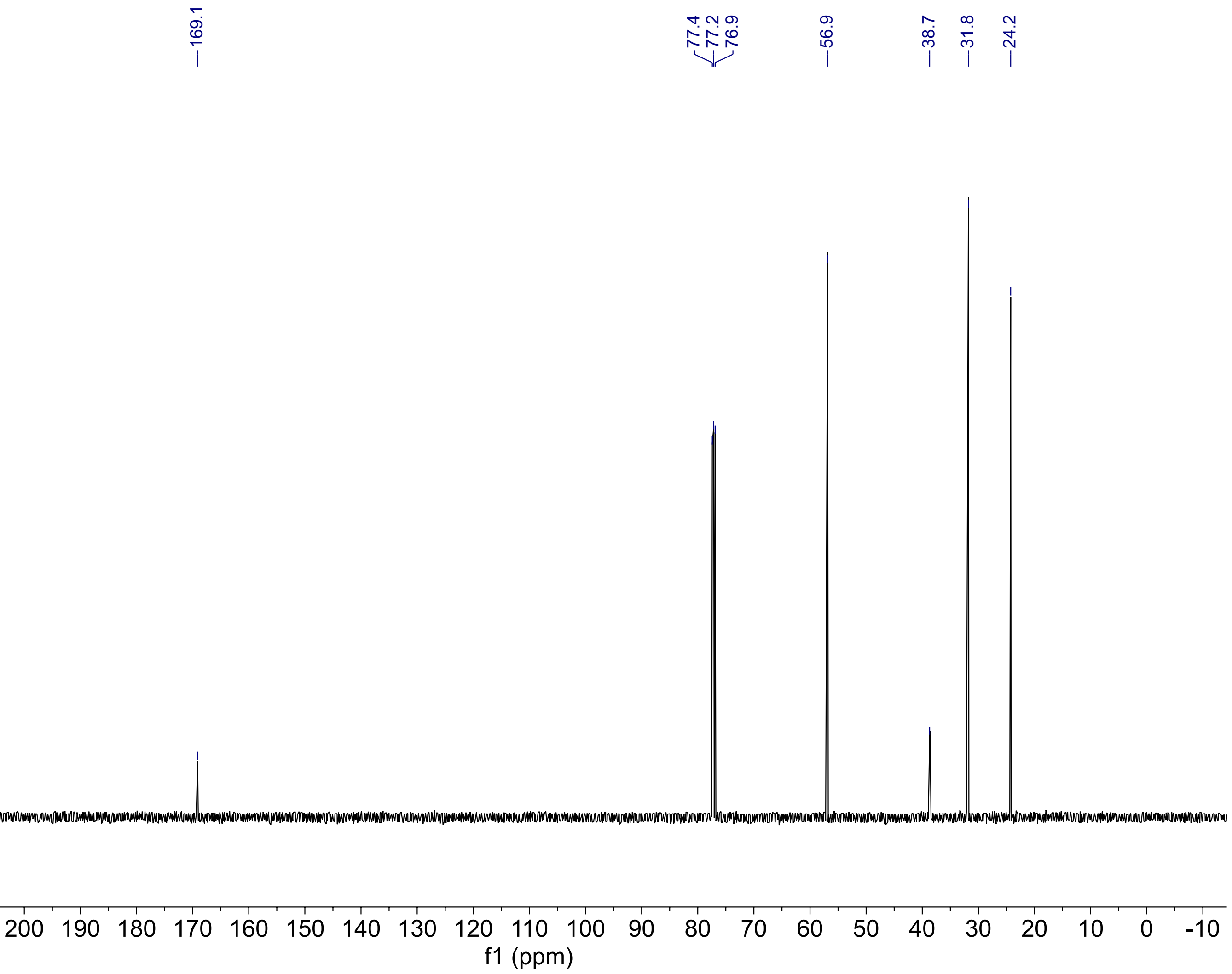

$^1H\{^1H\}$ NMR of *N,N*-dimethyl-3,4,5,6-tetrahydropyridin-2-amine (1j) ($CDCl_3$, 500 MHz)

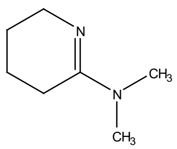

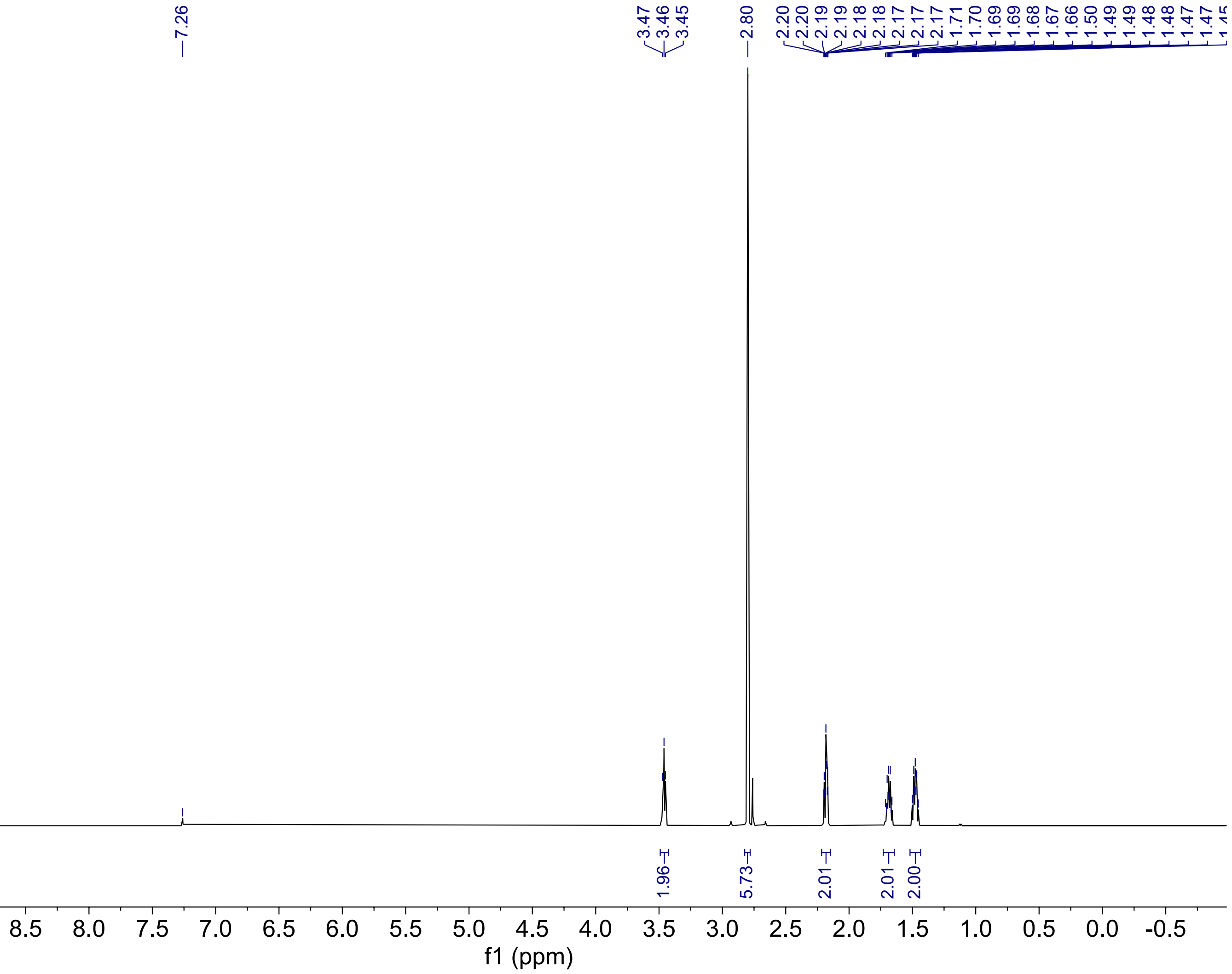

$^{13}C\{^1H\}$ NMR of *N,N*-dimethyl-3,4,5,6-tetrahydropyridin-2-amine (1j) ($CDCl_3$, 126 MHz)

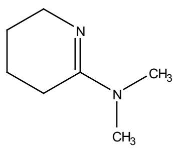

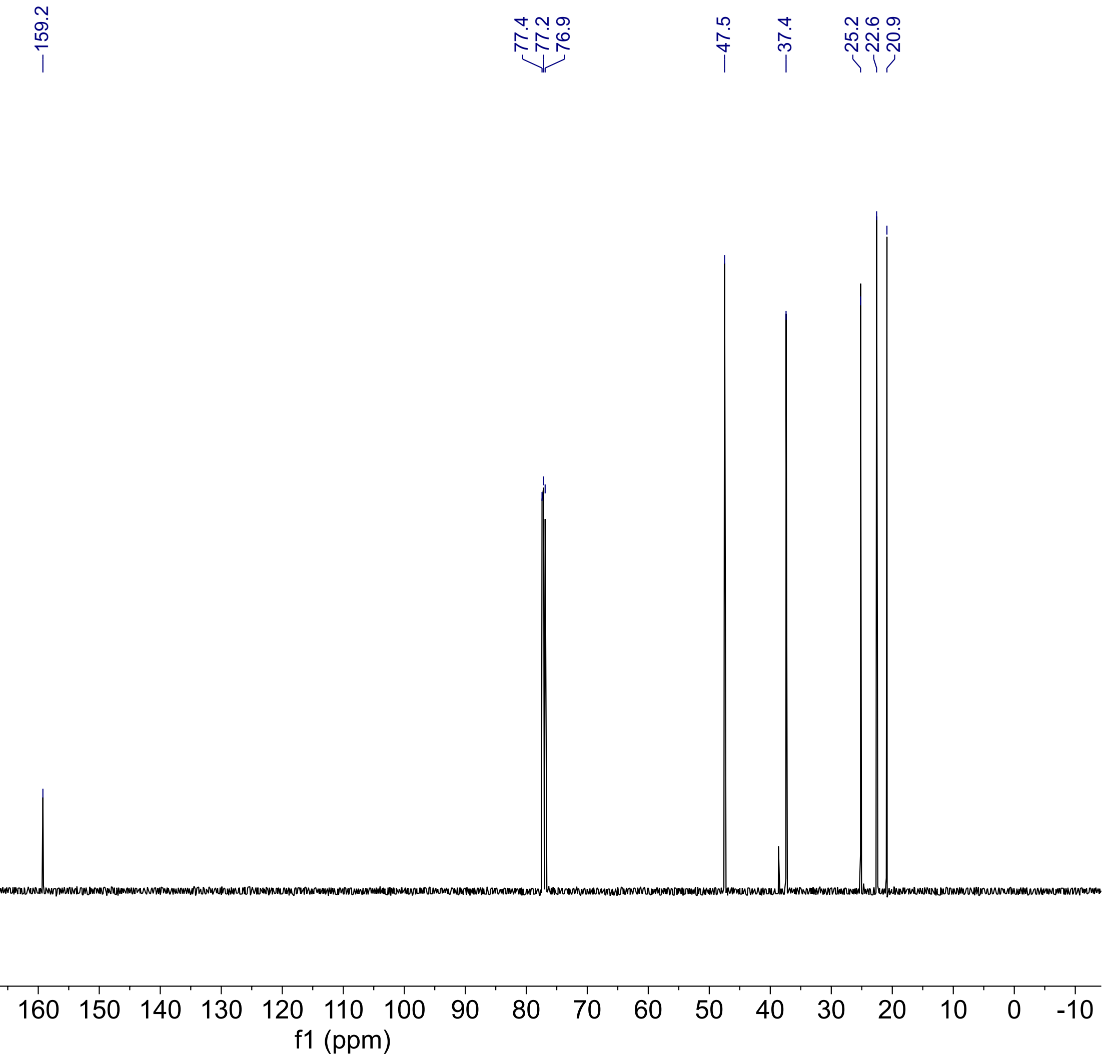

^{1}H NMR of *N,N*-dimethyl-3,4,5,6-tetrahydro-2*H*-azepin-7-amine (1k) ($CDCl_3$, 500 MHz)

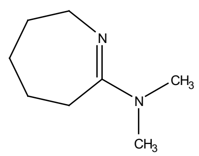

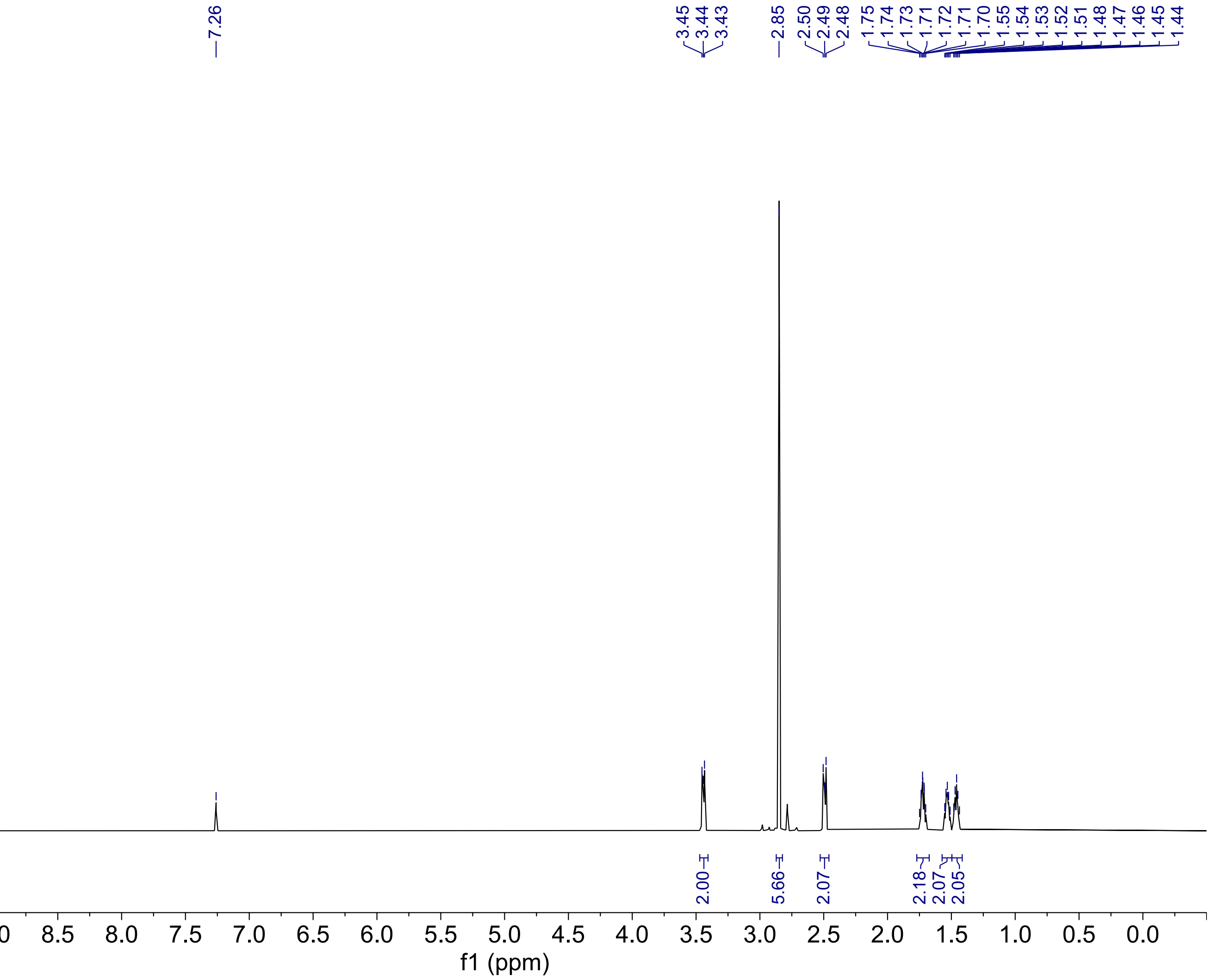

$^{13}C\{^{1}H\}$ NMR of *N,N*-dimethyl-3,4,5,6-tetrahydro-2*H*-azepin-7-amine (1k) ($CDCl_3$, 126 MHz)

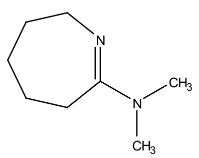

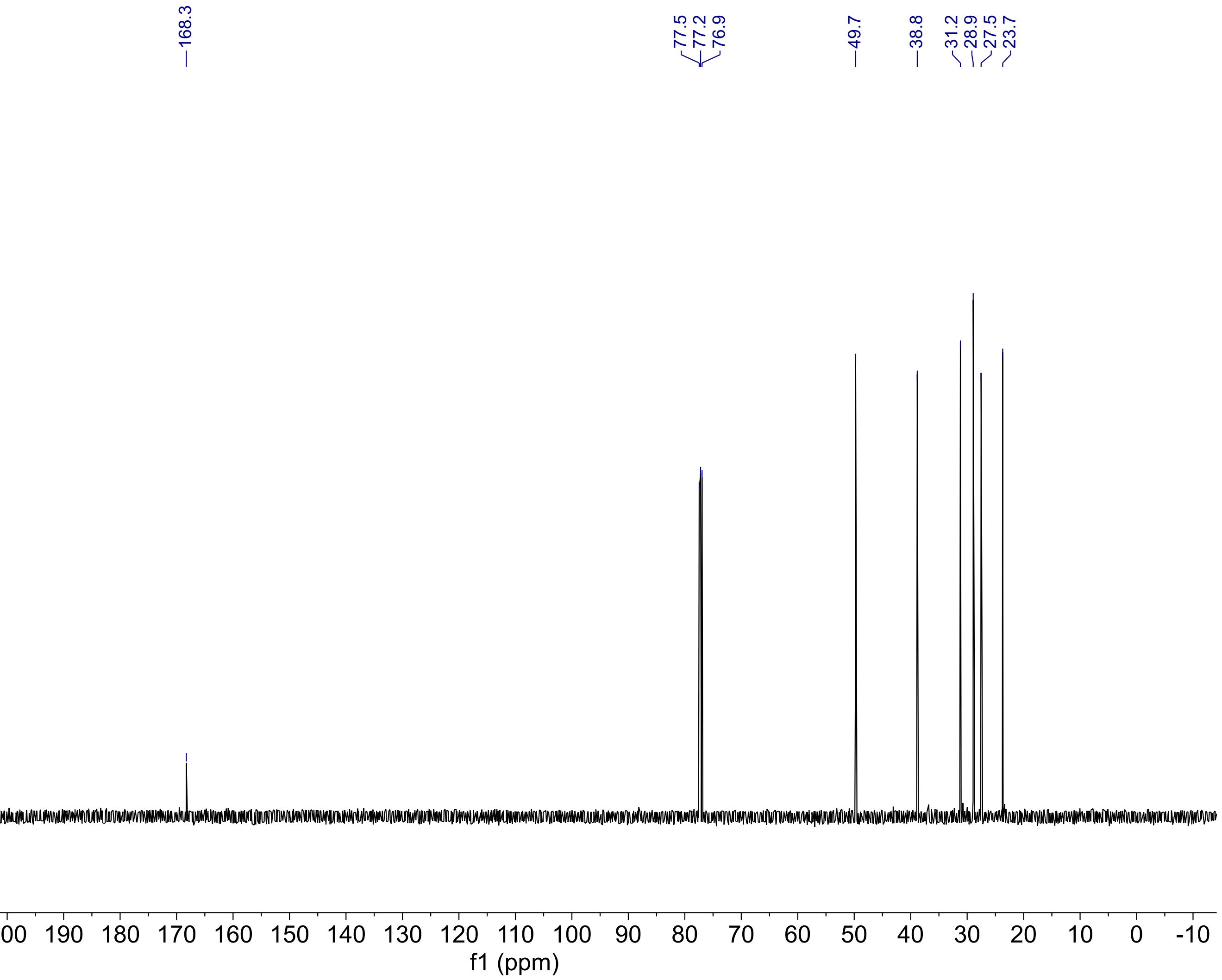

^{1}H NMR of (*E*)-*N'*-cyclohexyl-*N*,*N*-dimethylacetimidamide (1l) ($CDCl_3$, 500 MHz)

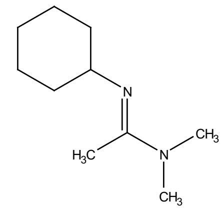

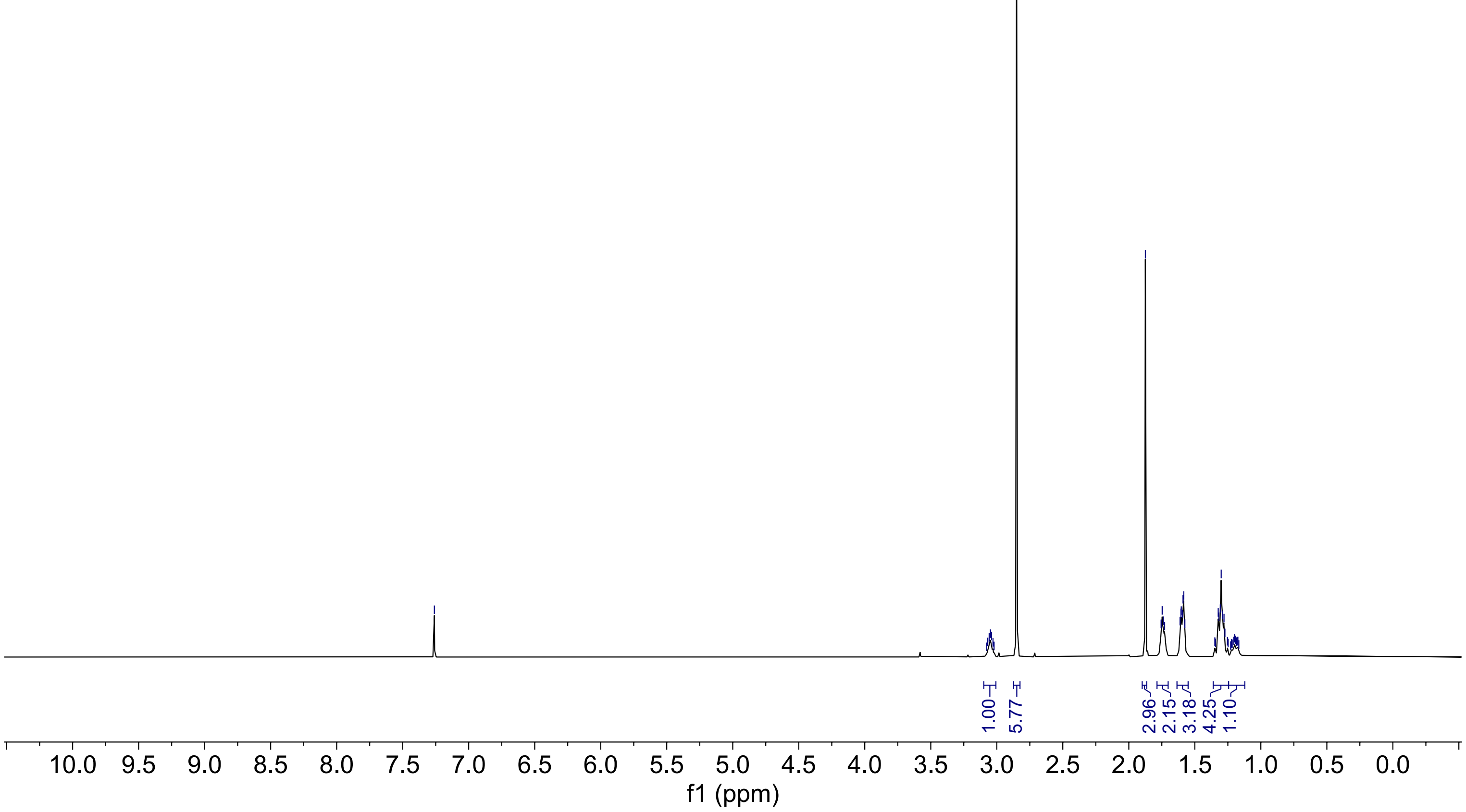

$^{13}C\{^{1}H\}$ NMR of (*E*)-*N*'-cyclohexyl-*N*,*N*-dimethylacetimidamide (1l) ($CDCl_3$, 126 MHz)

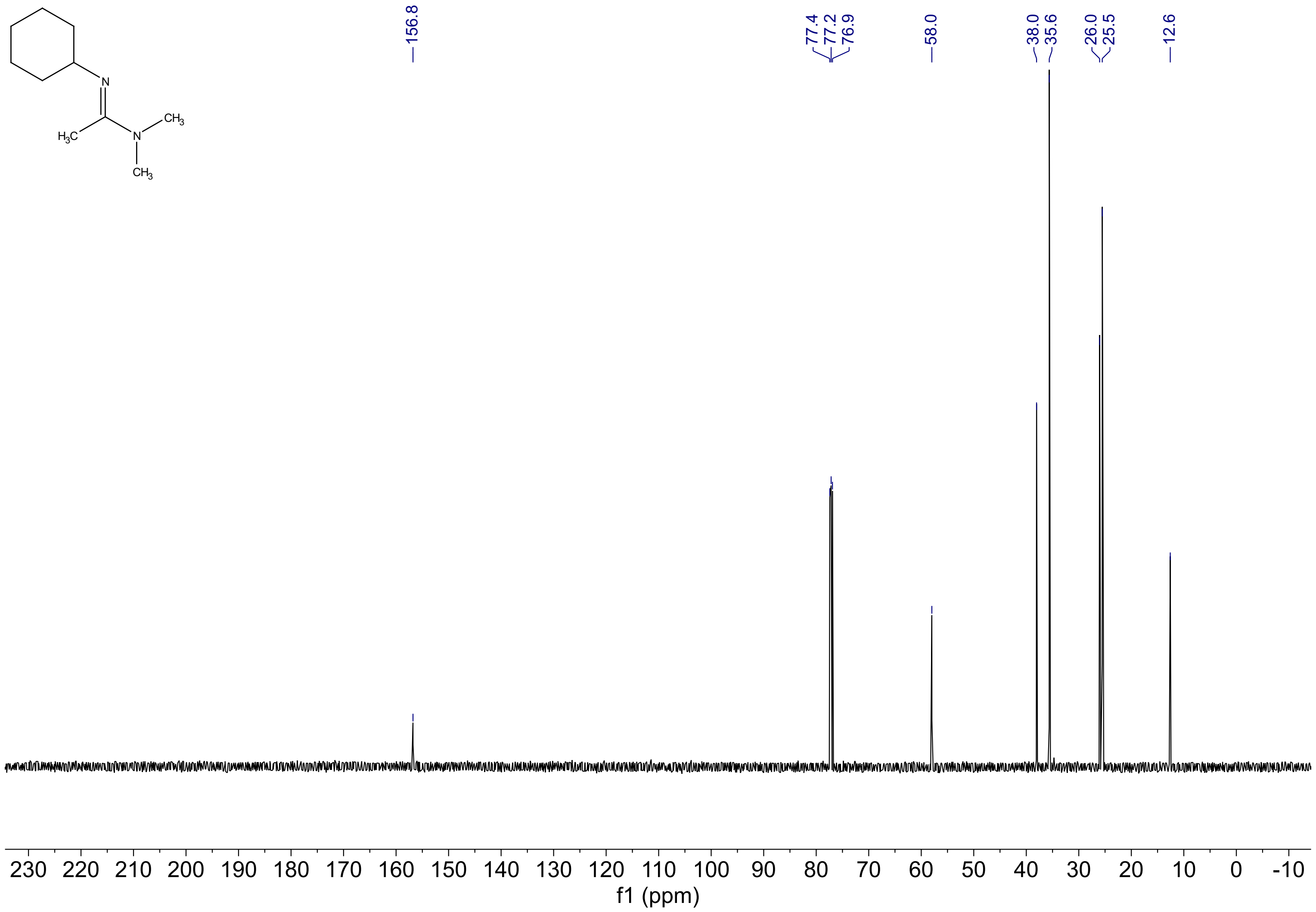

^{1}H NMR of (*E*)-*N*'-benzyl-*N*,*N*-dimethylacetimidamide (1m) ($CDCl_3$, 500 MHz)

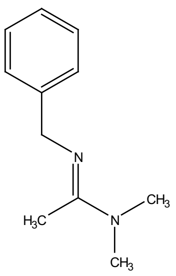

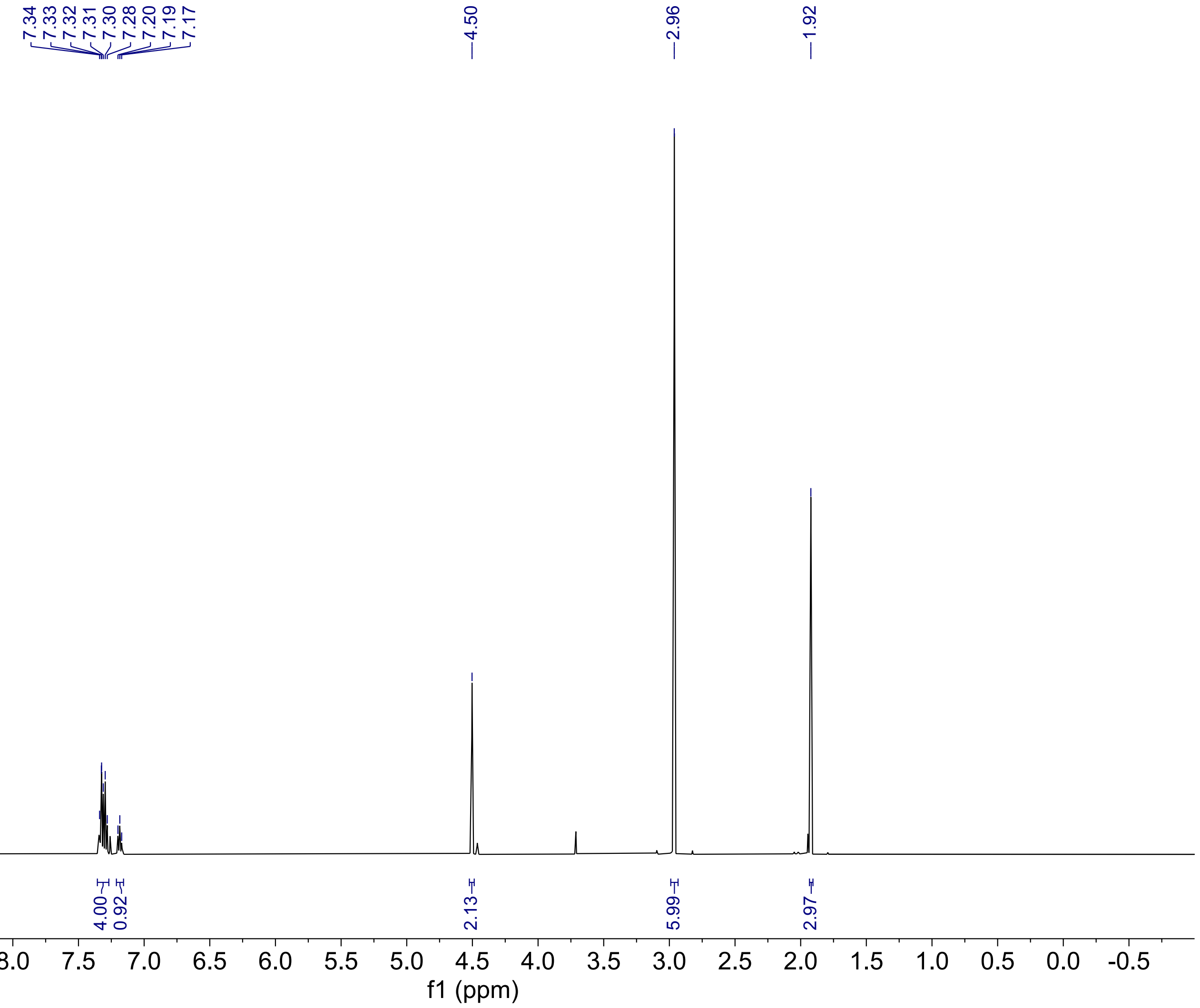

$^{13}C\{^1H\}$ NMR of (*E*)-*N*'-benzyl-*N*,*N*-dimethylacetimidamide (1m) ($CDCl_3$, 126 MHz)

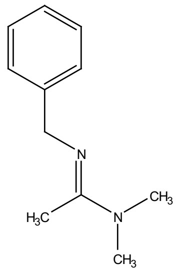

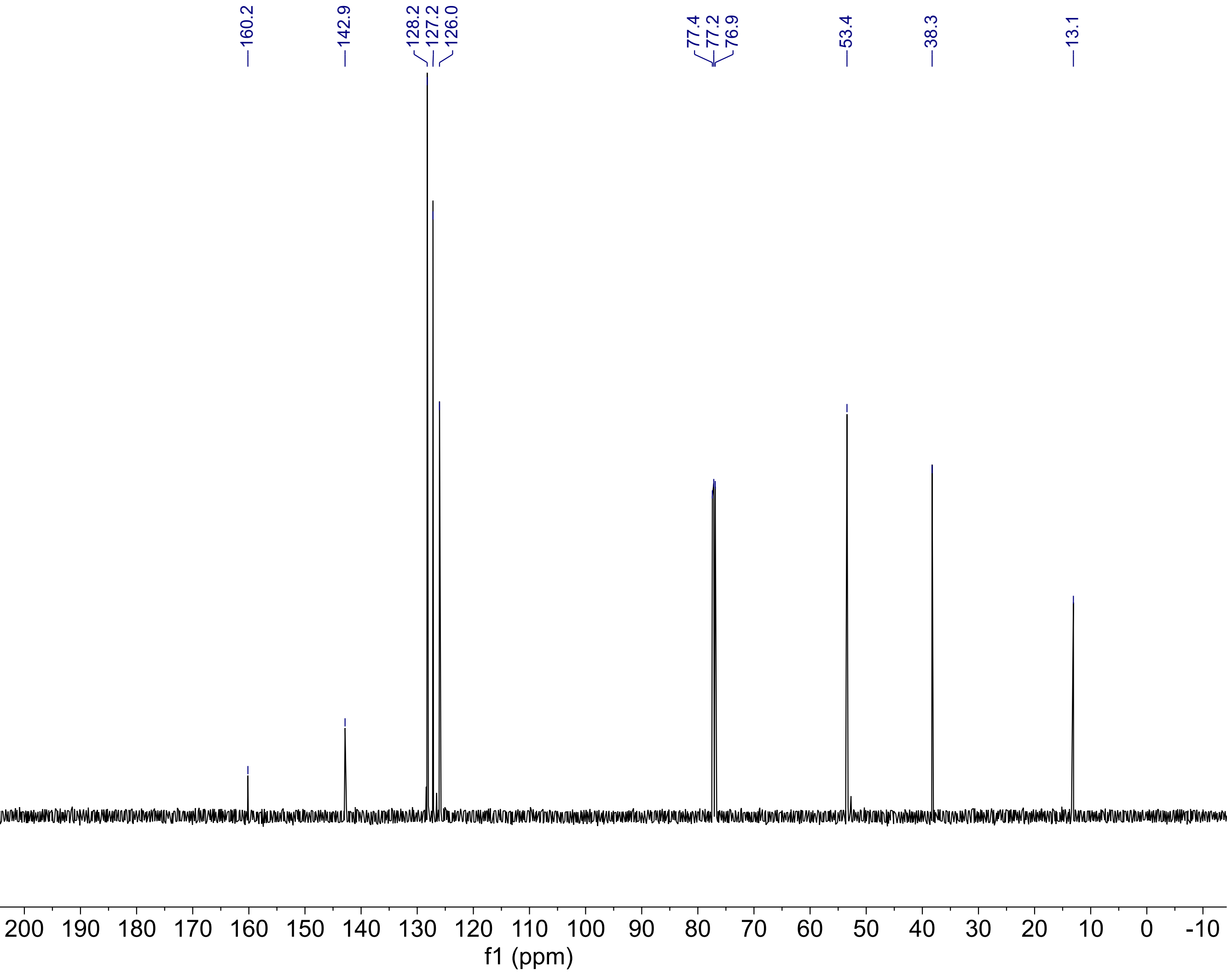

^{1}H NMR of (*E*)-*N,N*-dimethyl-*N'*-phenylacetimidamide (1n) ($CDCl_3$, 500 MHz)

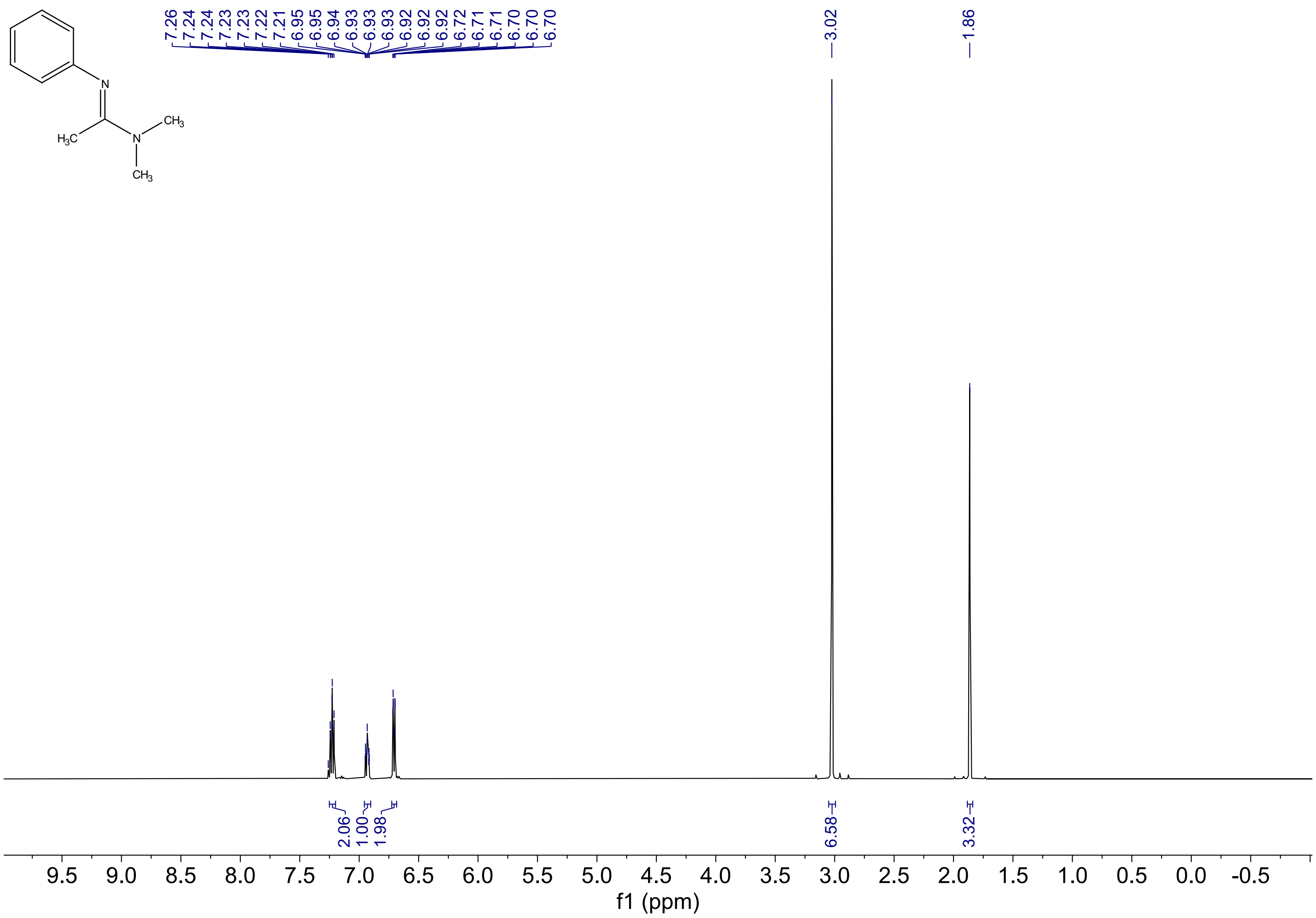

$^{13}C\{^{1}H\}$ NMR of (*E*)-*N,N*-dimethyl-*N'*-phenylacetimidamide (1n) ($CDCl_3$, 126 MHz)

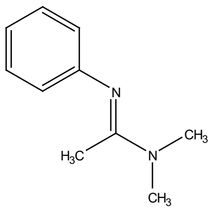

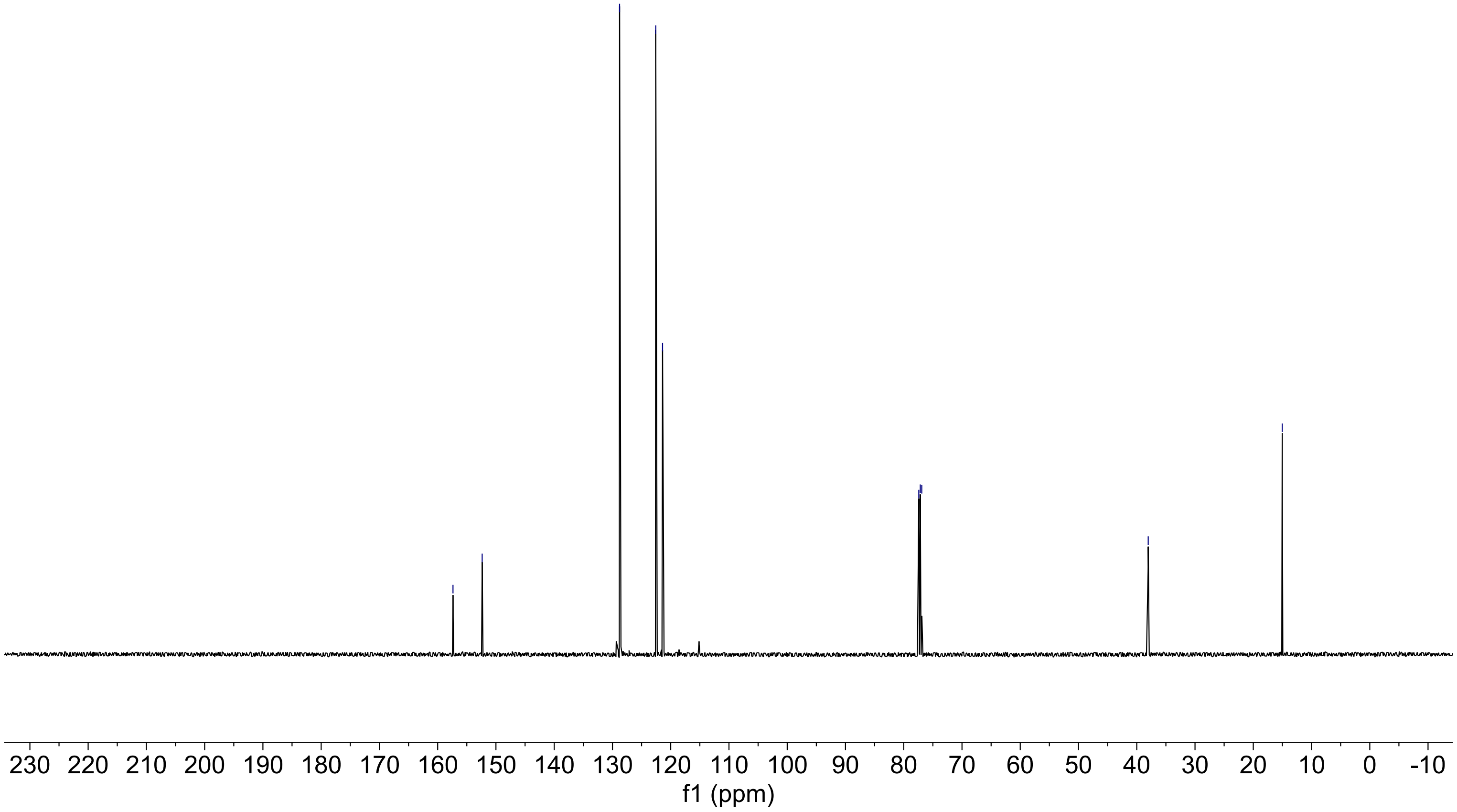

^{1}H NMR of (*E*)-*N'*-(*sec*-butyl)-*N*-methyl-*N*-((4,4,5,5-tetramethyl-1,3,2-dioxaborolan-2-yl)methyl)acetimidamide (2a) ($CDCl_3$, 500 MHz)

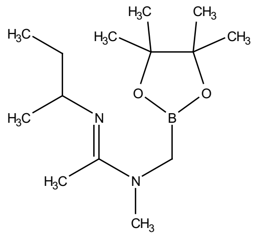

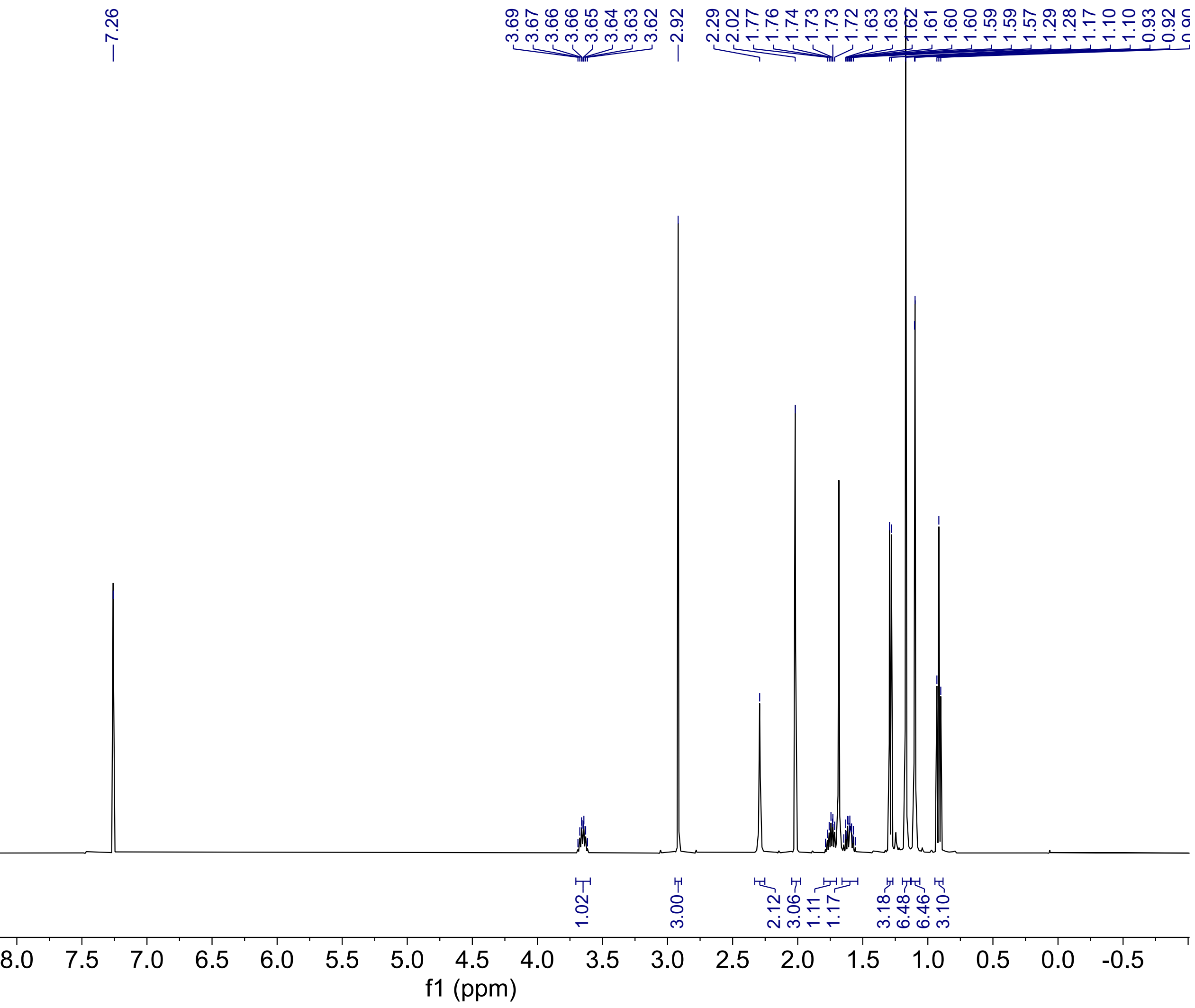

$^{13}C\{^1H\}$ NMR of (*E*)-*N'*-(*sec*-butyl)-*N*-methyl-*N*-((4,4,5,5-tetramethyl-1,3,2-dioxaborolan-2-yl)methyl)acetimidamide (2a) ($CDCl_3$, 126 MHz)

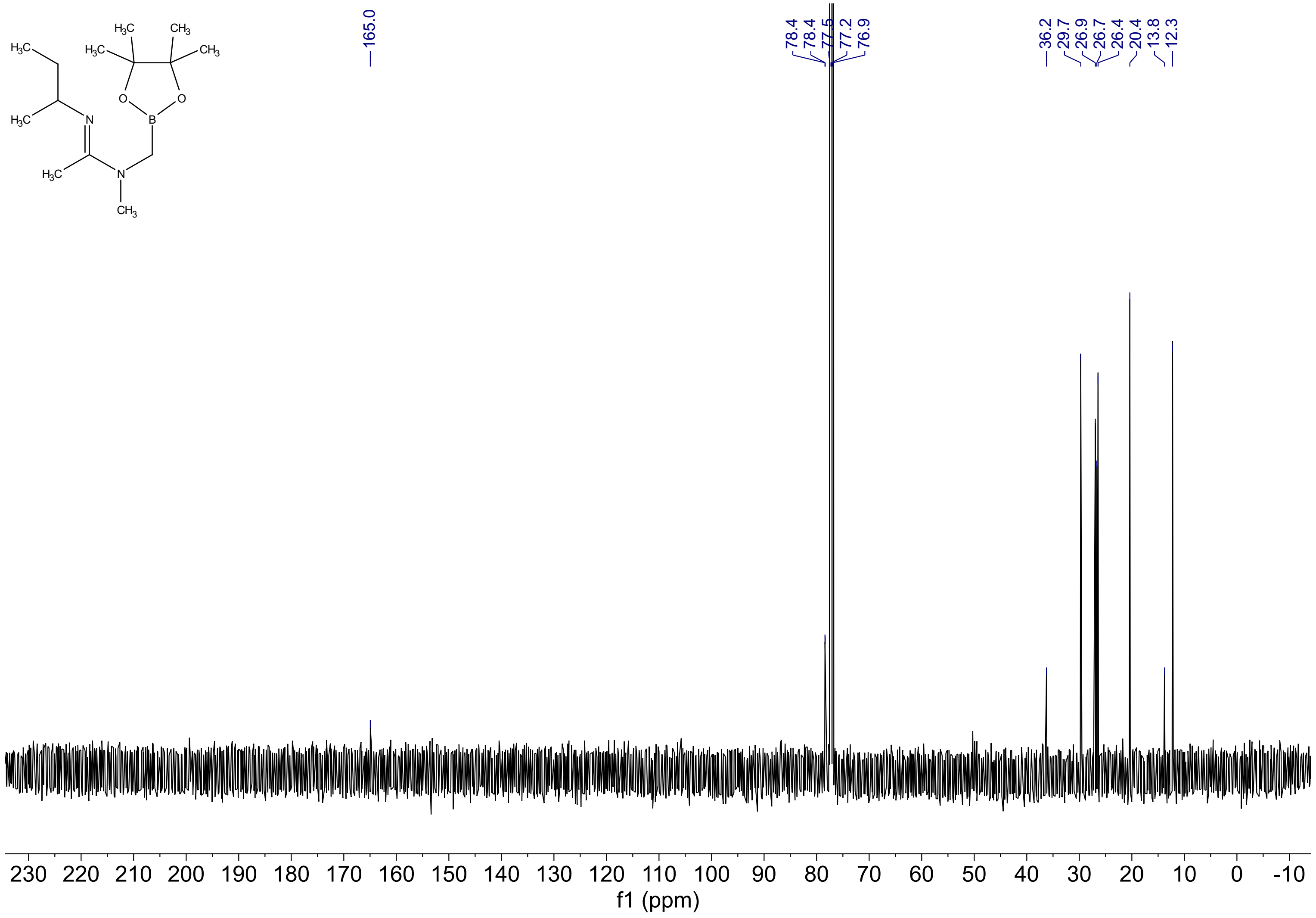

^{11}B NMR of (*E*)-*N'*-(*sec*-butyl)-*N*-methyl-*N*-((4,4,5,5-tetramethyl-1,3,2-dioxaborolan-2-yl)methyl)acetimidamide (2a) ($CDCl_3$, 160 MHz)

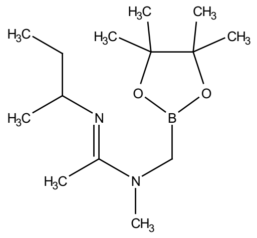

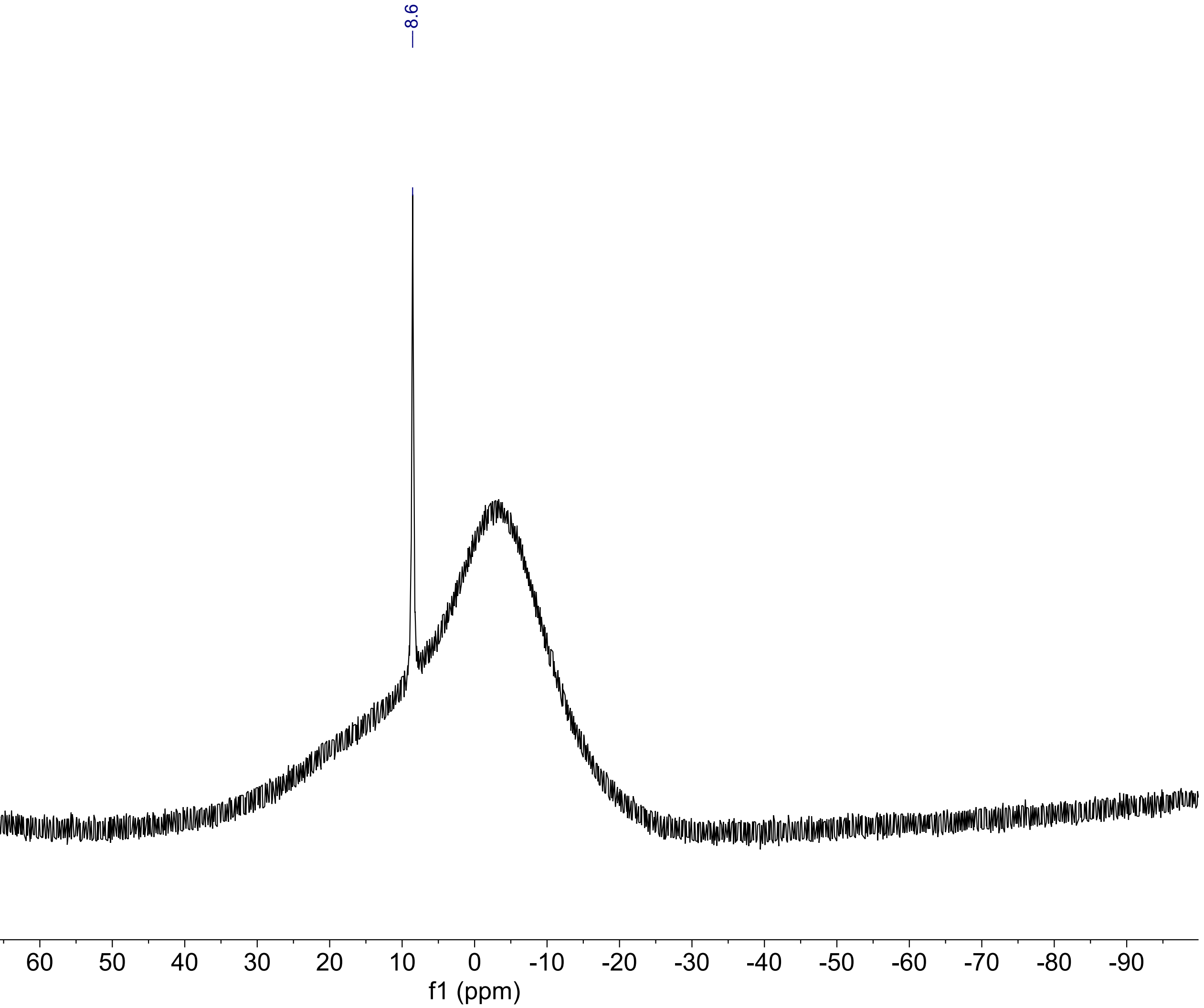

^{1}H NMR of (*E*)-*N*,*N’*-dimethyl-*N*-((4,4,5,5-tetramethyl-1,3,2-dioxaborolan-2-yl)methyl)acetimidamide (2b) ($CDCl_3$, 500 MHz)

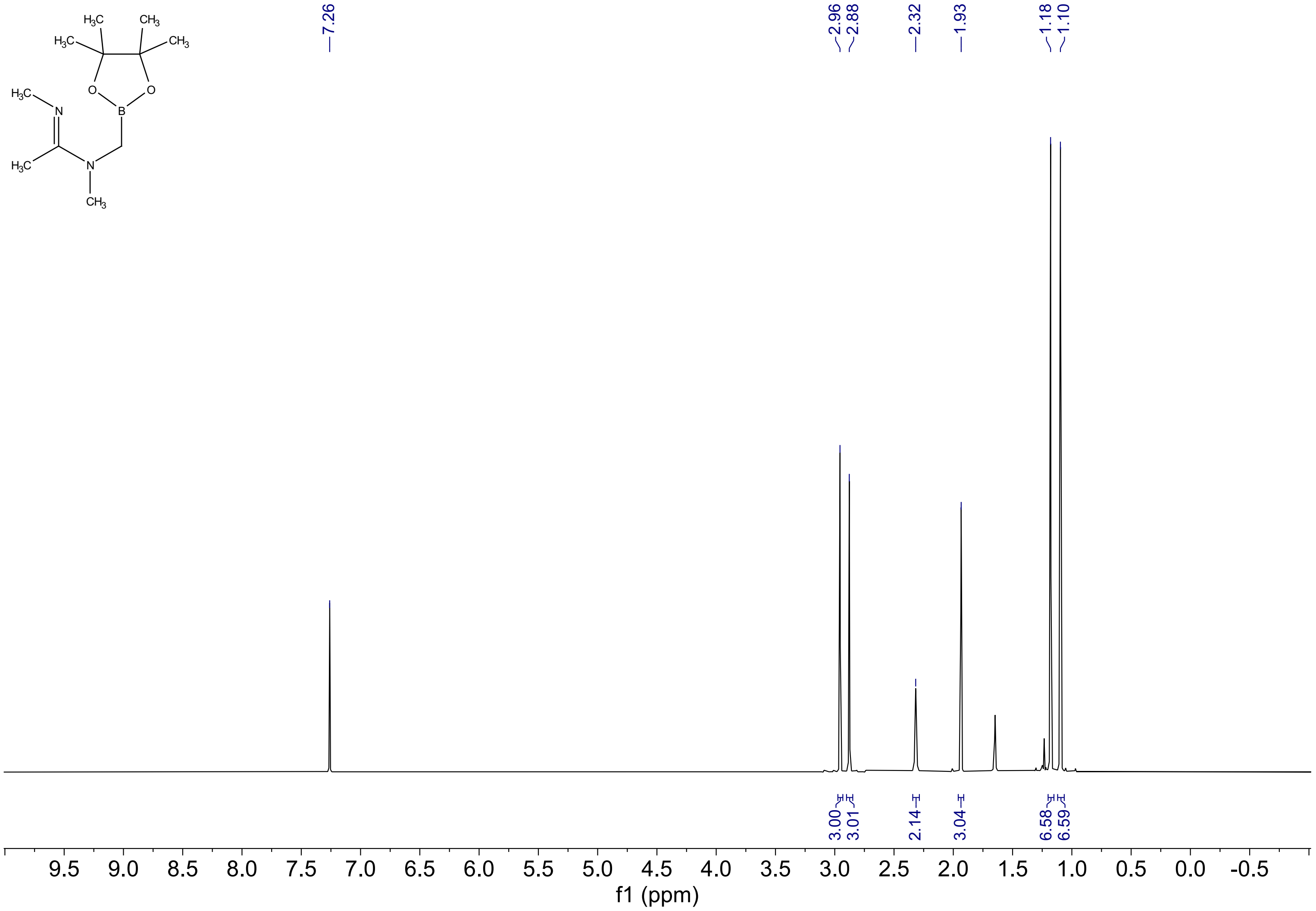

$^{13}C\{^1H\}$ NMR of (*E*)-*N*,*N'*-dimethyl-*N*-((4,4,5,5-tetramethyl-1,3,2-dioxaborolan-2-yl)methyl)acetimidamide (2b) ($CDCl_3$, 126 MHz)

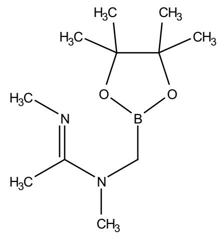

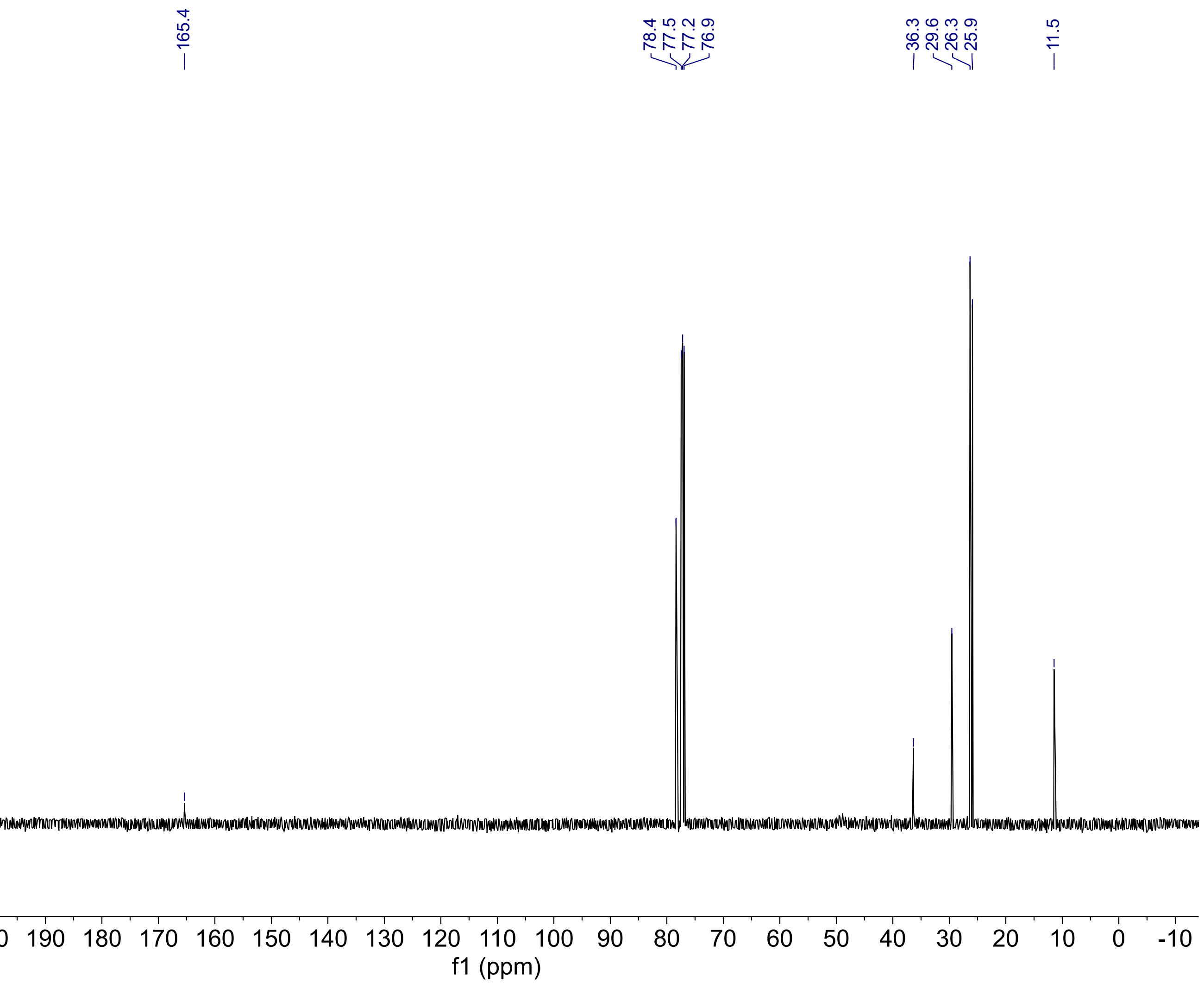

^{11}B NMR of (*E*)-*N*,*N'*-dimethyl-*N*-((4,4,5,5-tetramethyl-1,3,2-dioxaborolan-2-yl)methyl)acetimidamide (2b) ($CDCl_3$, 160 MHz)

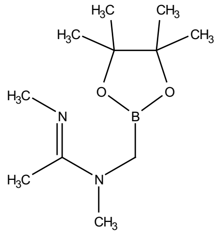

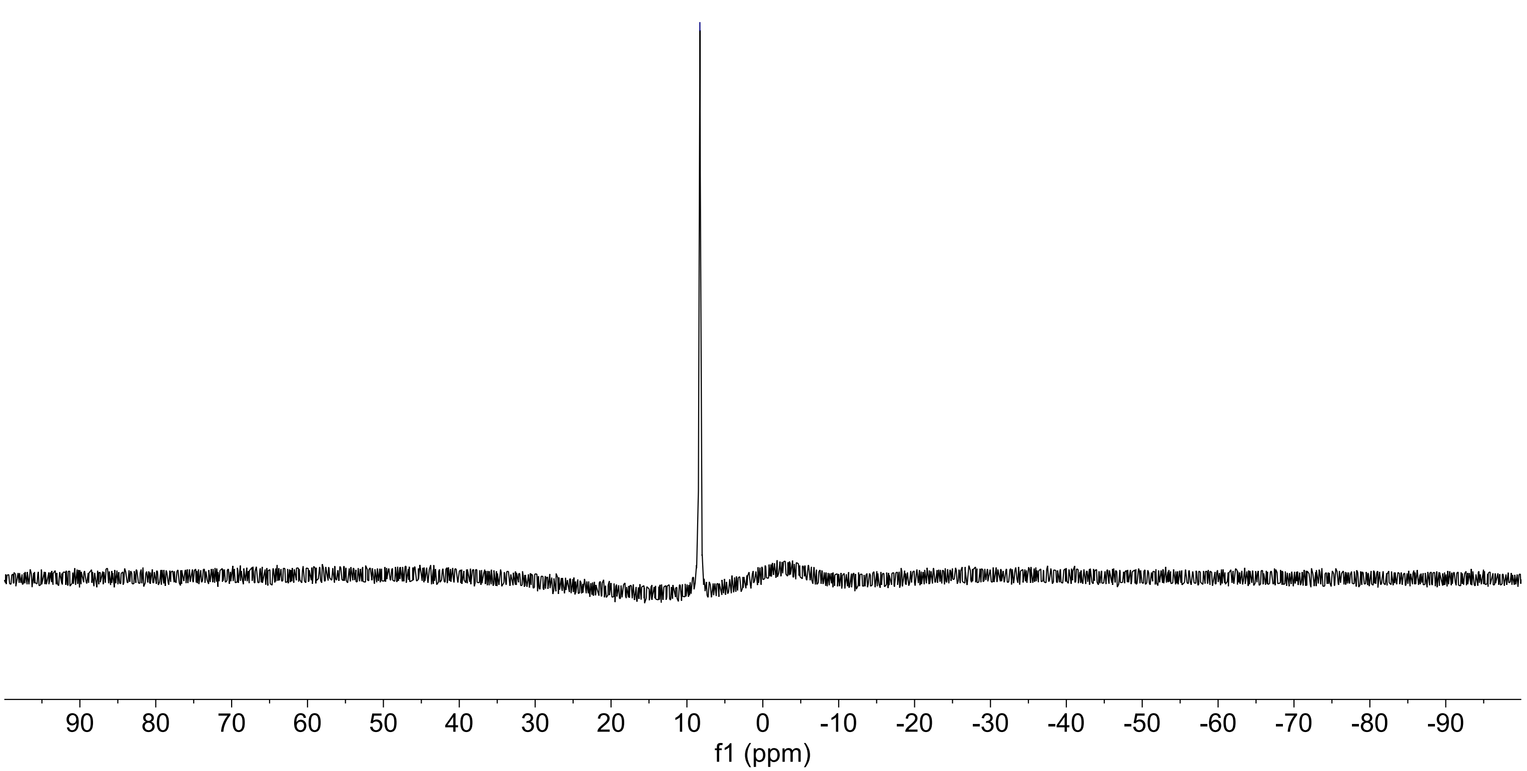

^{1}H NMR of (*E*)-*N'*-ethyl-*N*-methyl-*N*-((4,4,5,5-tetramethyl-1,3,2-dioxaborolan-2-yl)methyl)formimidamide (2c) ($CDCl_3$, 500 MHz)

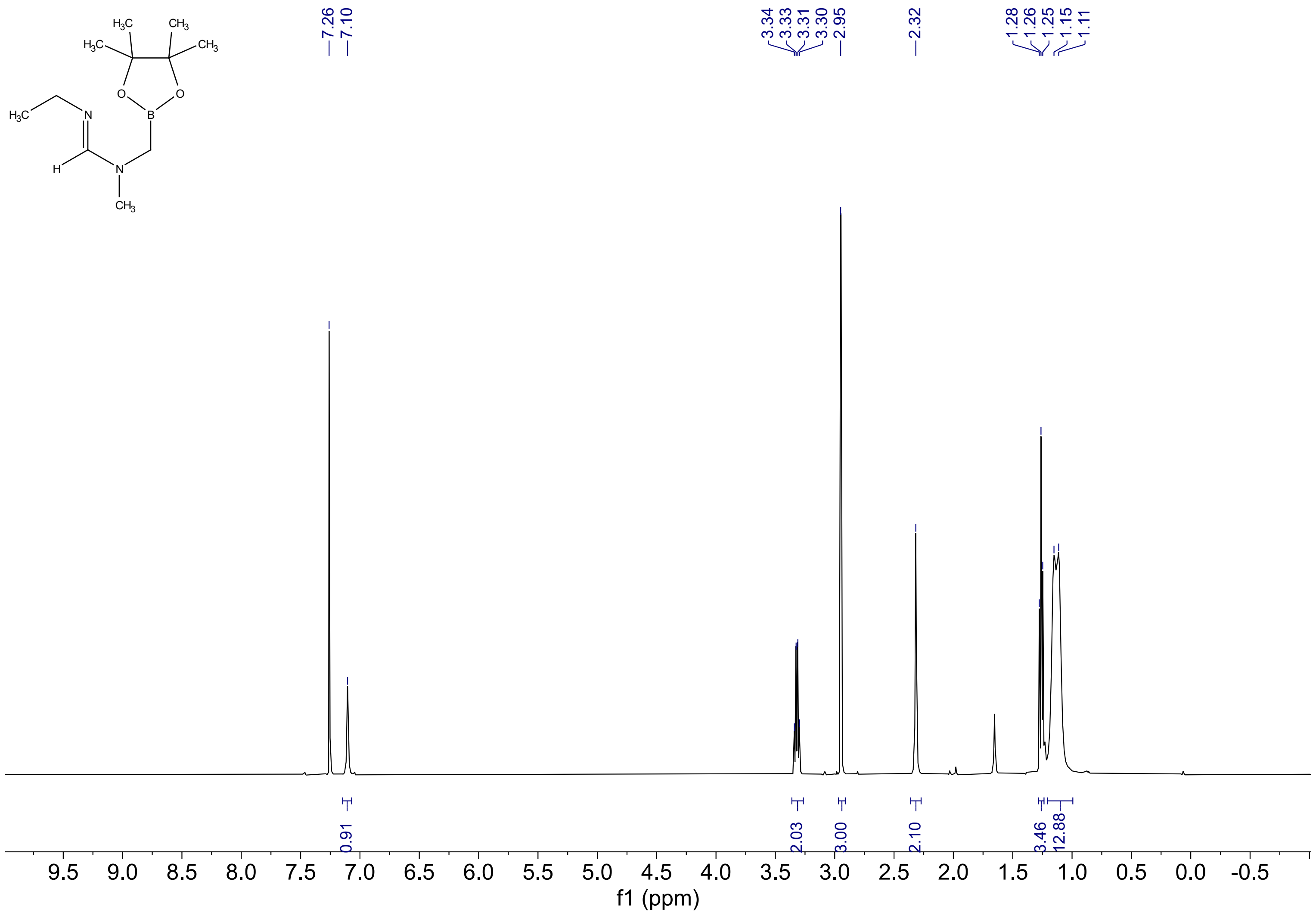

$^{13}C\{^{1}H\}$ NMR of (*E*)-*N'*-ethyl-*N*-methyl-*N*-((4,4,5,5-tetramethyl-1,3,2-dioxaborolan-2-yl)methyl)formimidamide (2c) ($CDCl_3$, 126 MHz)

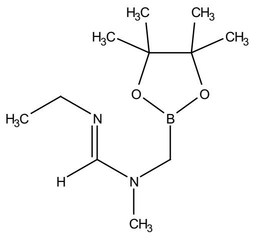

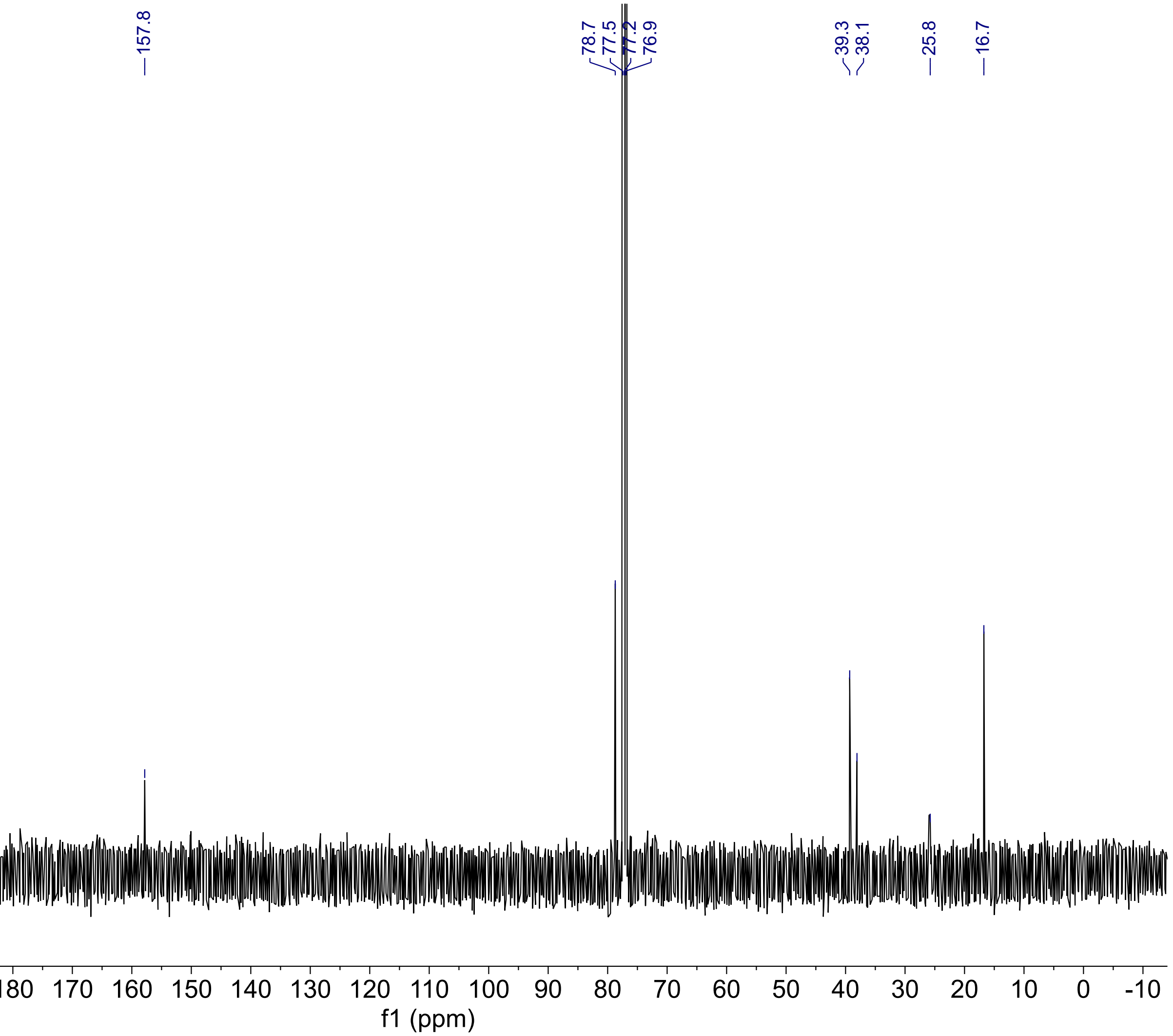

^{11}B NMR of (*E*)-*N'*-ethyl-*N*-methyl-*N*-((4,4,5,5-tetramethyl-1,3,2-dioxaborolan-2-yl)methyl)formimidamide (2c) ($CDCl_3$, 160 MHz)

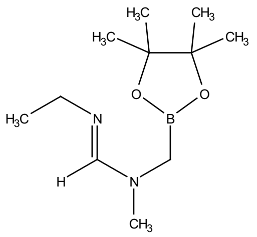

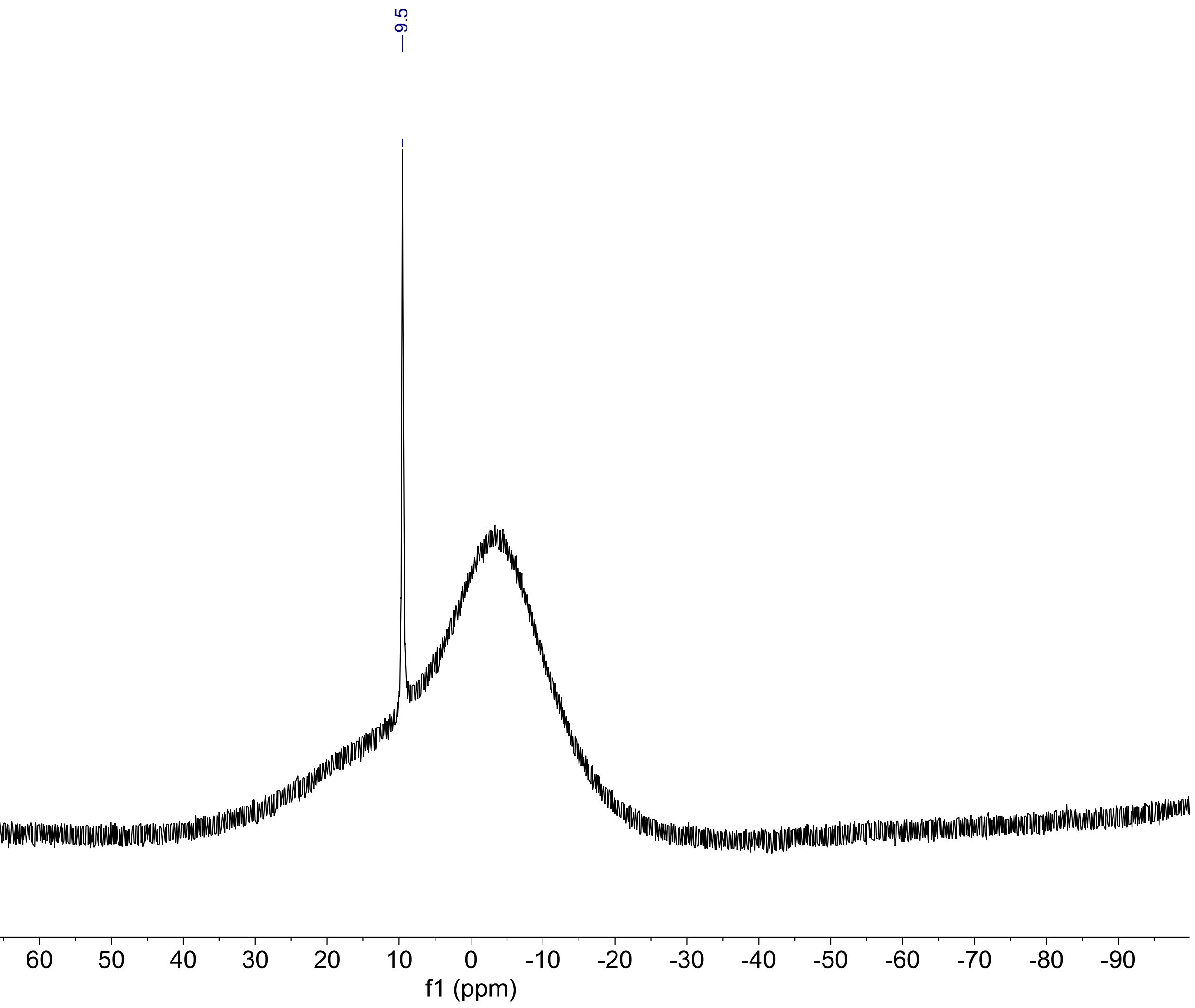

^{1}H NMR of (*E*)-*N*,*N'*-dimethyl-*N*-((4,4,5,5-tetramethyl-1,3,2-dioxaborolan-2-yl)methyl)propionimidamide (2d) ($CDCl_3$, 500 MHz)

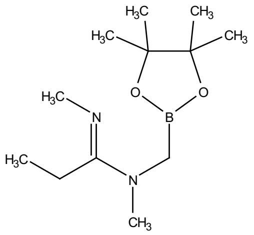

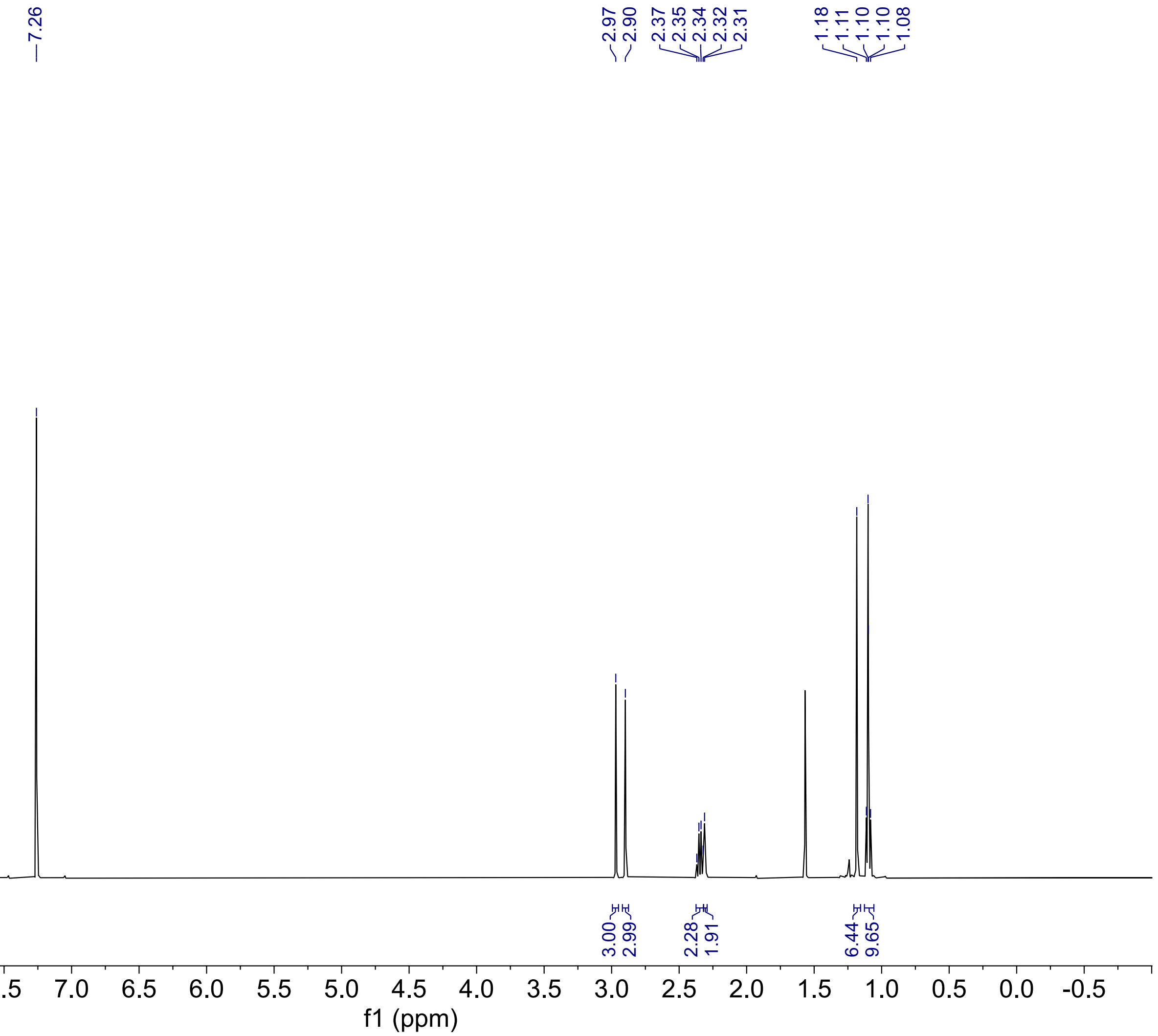

$^{13}C\{^{1}H\}$ NMR of (*E*)-*N*,*N'*-dimethyl-*N*-((4,4,5,5-tetramethyl-1,3,2-dioxaborolan-2-yl)methyl)propionimidamide (2d) ($CDCl_3$, 126 MHz)

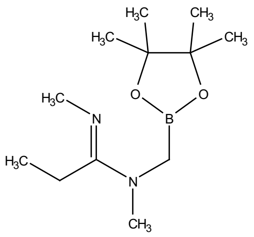

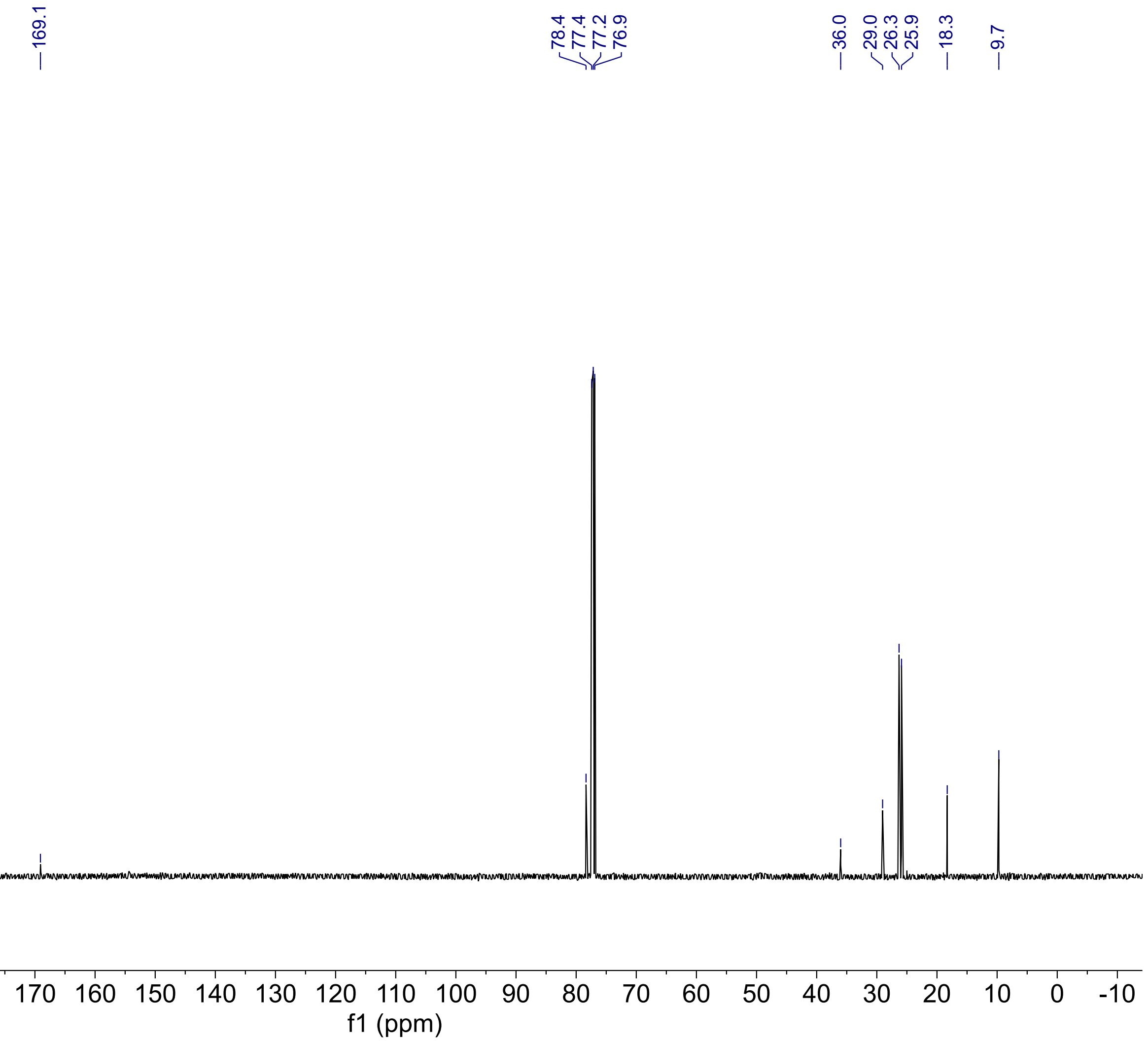

^{11}B NMR of (*E*)-*N*,*N'*-dimethyl-*N*-((4,4,5,5-tetramethyl-1,3,2-dioxaborolan-2-yl)methyl)propionimidamide (2d) ($CDCl_3$, 160 MHz)

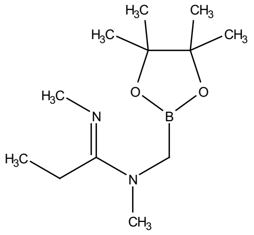

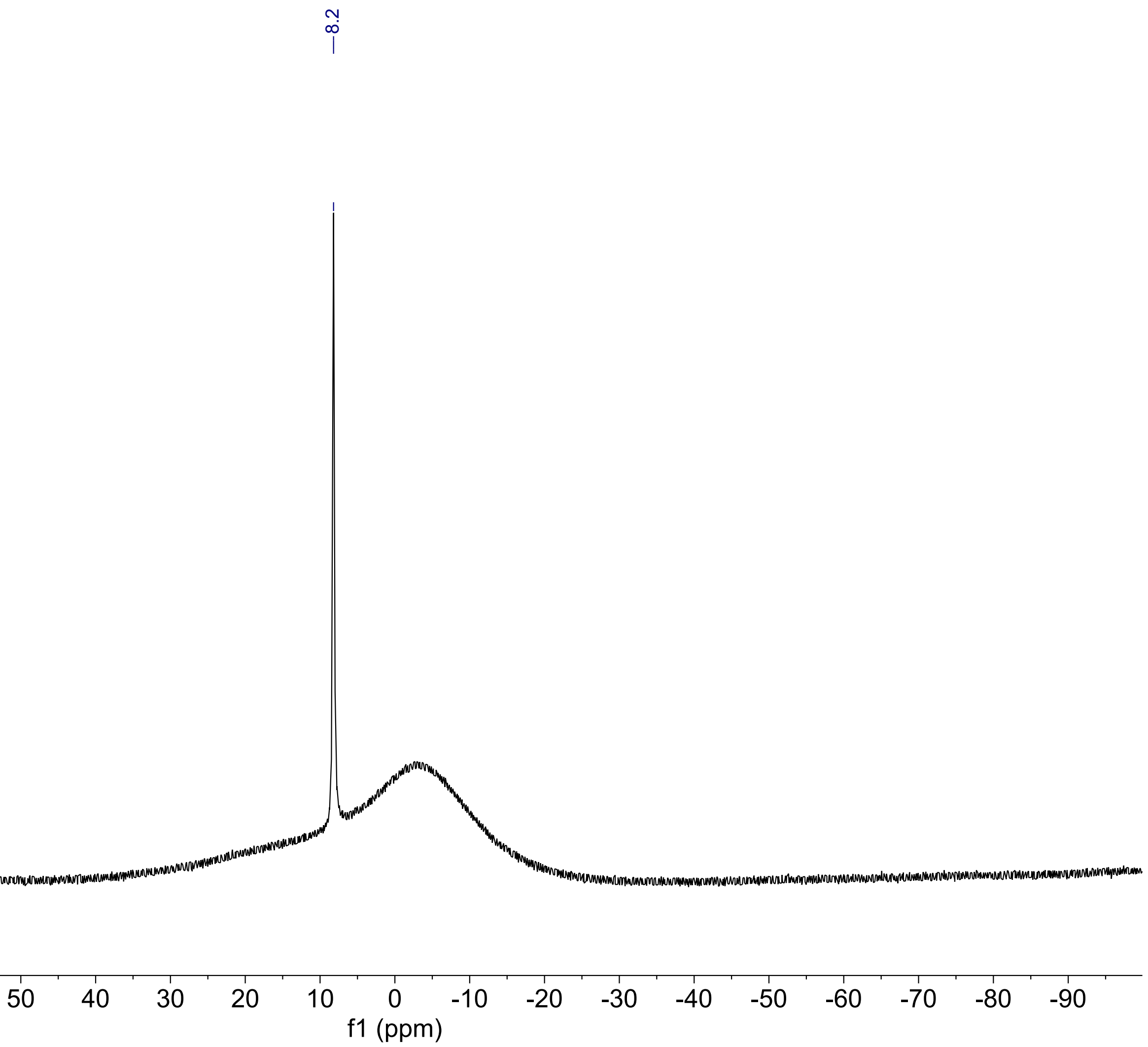

^{1}H NMR of (*E*)-*N'*-ethyl-*N*-methyl-*N*-((4,4,5,5-tetramethyl-1,3,2-dioxaborolan-2-yl)methyl)acetimidamide (2e) ($CDCl_3$, 500 MHz)

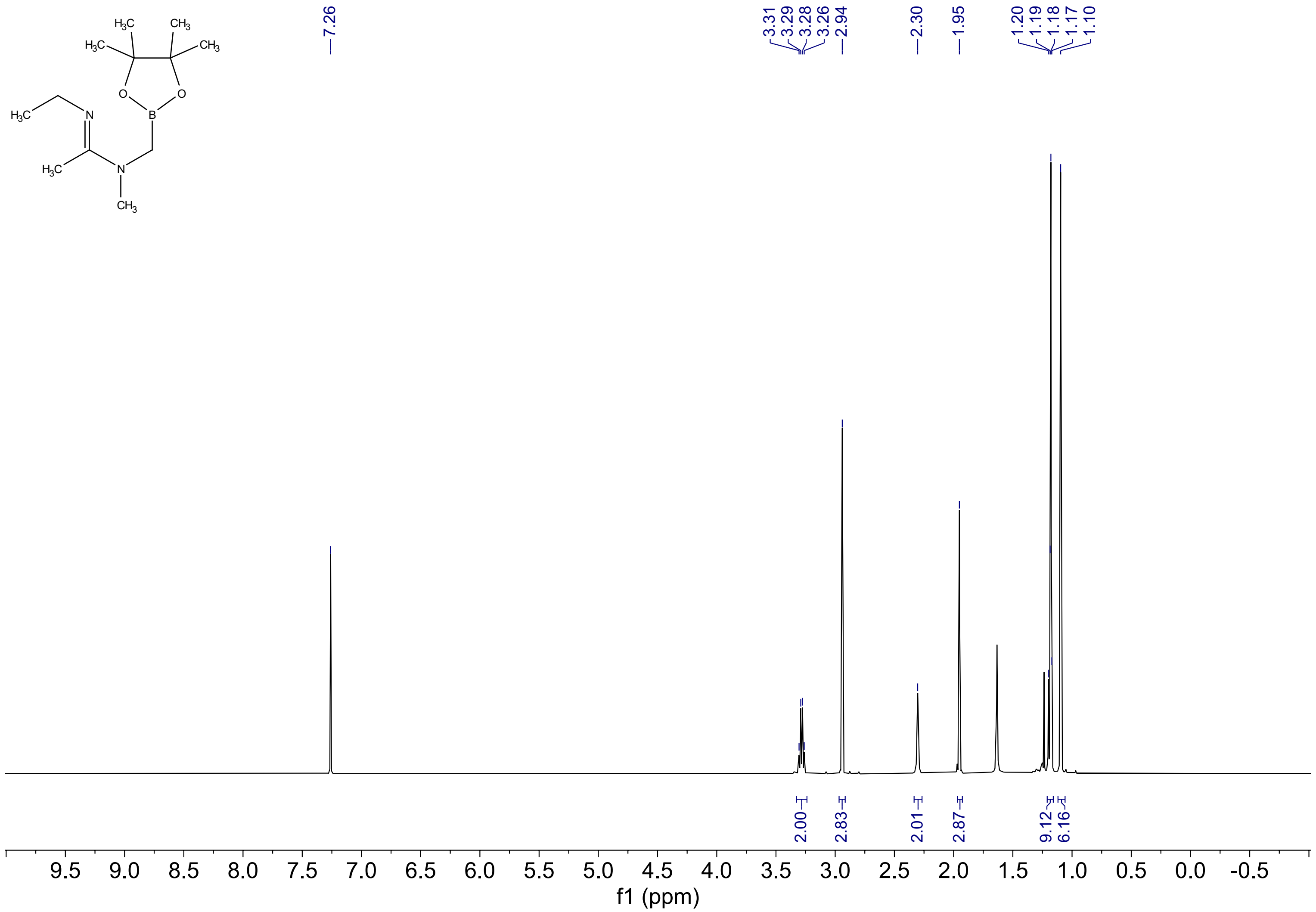

$^{13}C\{^{1}H\}$ NMR of (*E*)-*N'*-ethyl-*N*-methyl-*N*-((4,4,5,5-tetramethyl-1,3,2-dioxaborolan-2-yl)methyl)acetimidamide (2e) ($CDCl_3$, 126 MHz)

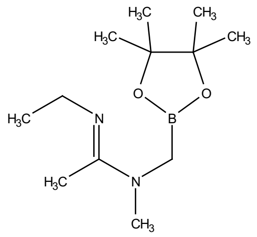

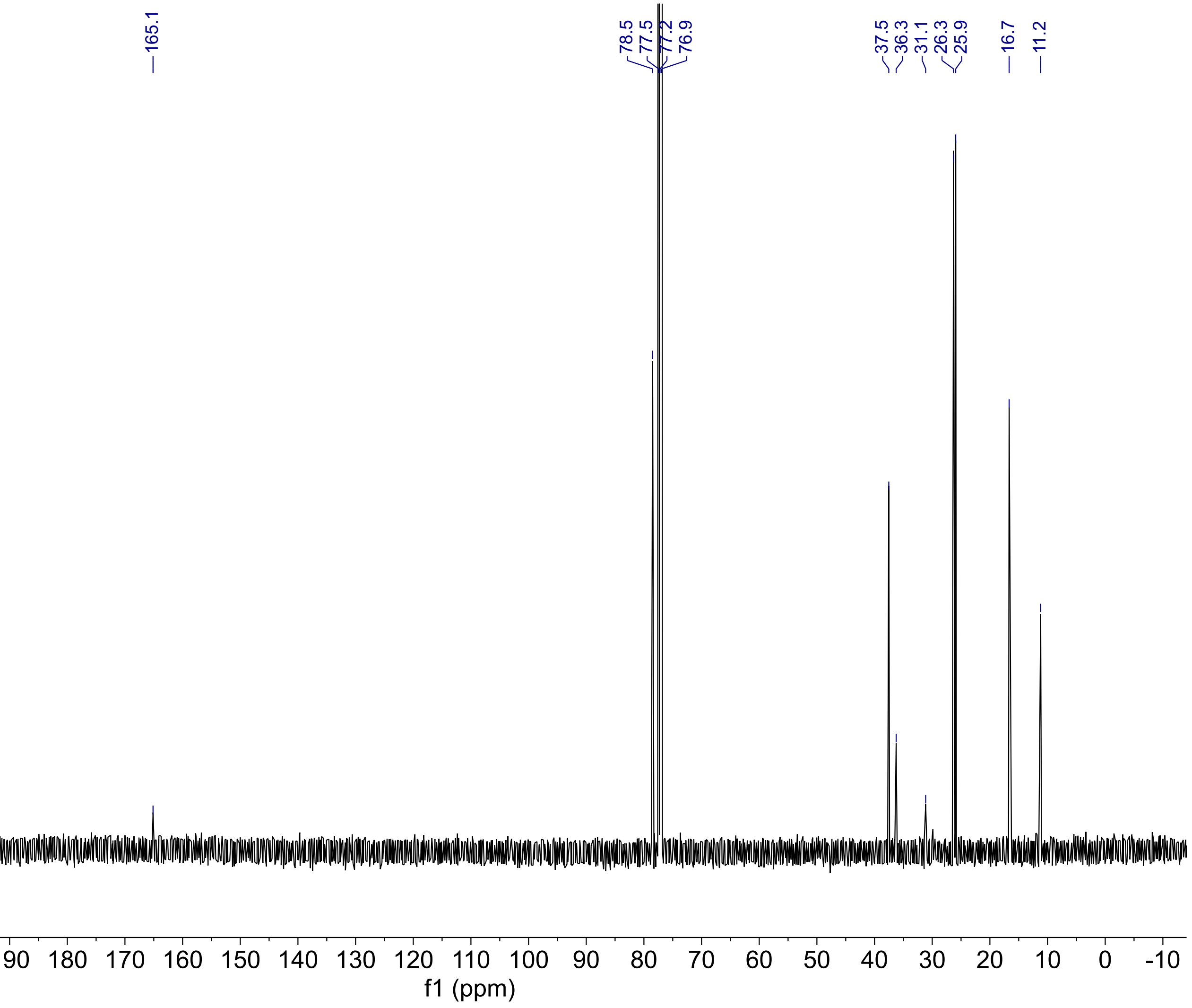

^{11}B NMR of (*E*)-*N'*-ethyl-*N*-methyl-*N*-((4,4,5,5-tetramethyl-1,3,2-dioxaborolan-2-yl)methyl)acetimidamide (2e) ($CDCl_3$, 160 MHz)

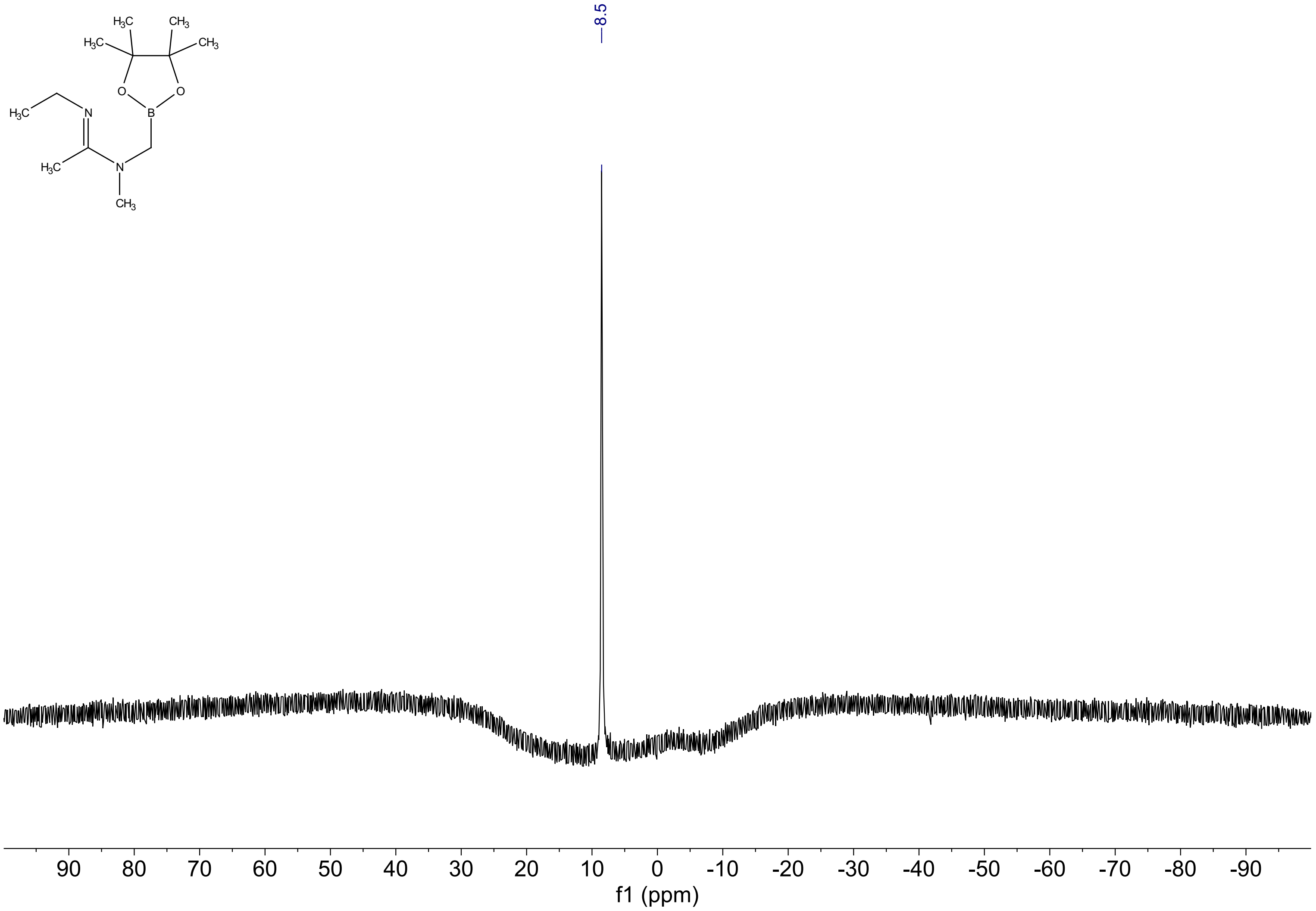

^{1}H NMR of (*E*)-*N'*-isopropyl-*N*-methyl-*N*-((4,4,5,5-tetramethyl-1,3,2-dioxaborolan-2-yl)methyl)acetimidamide (2f) ($CDCl_3$, 500 MHz)

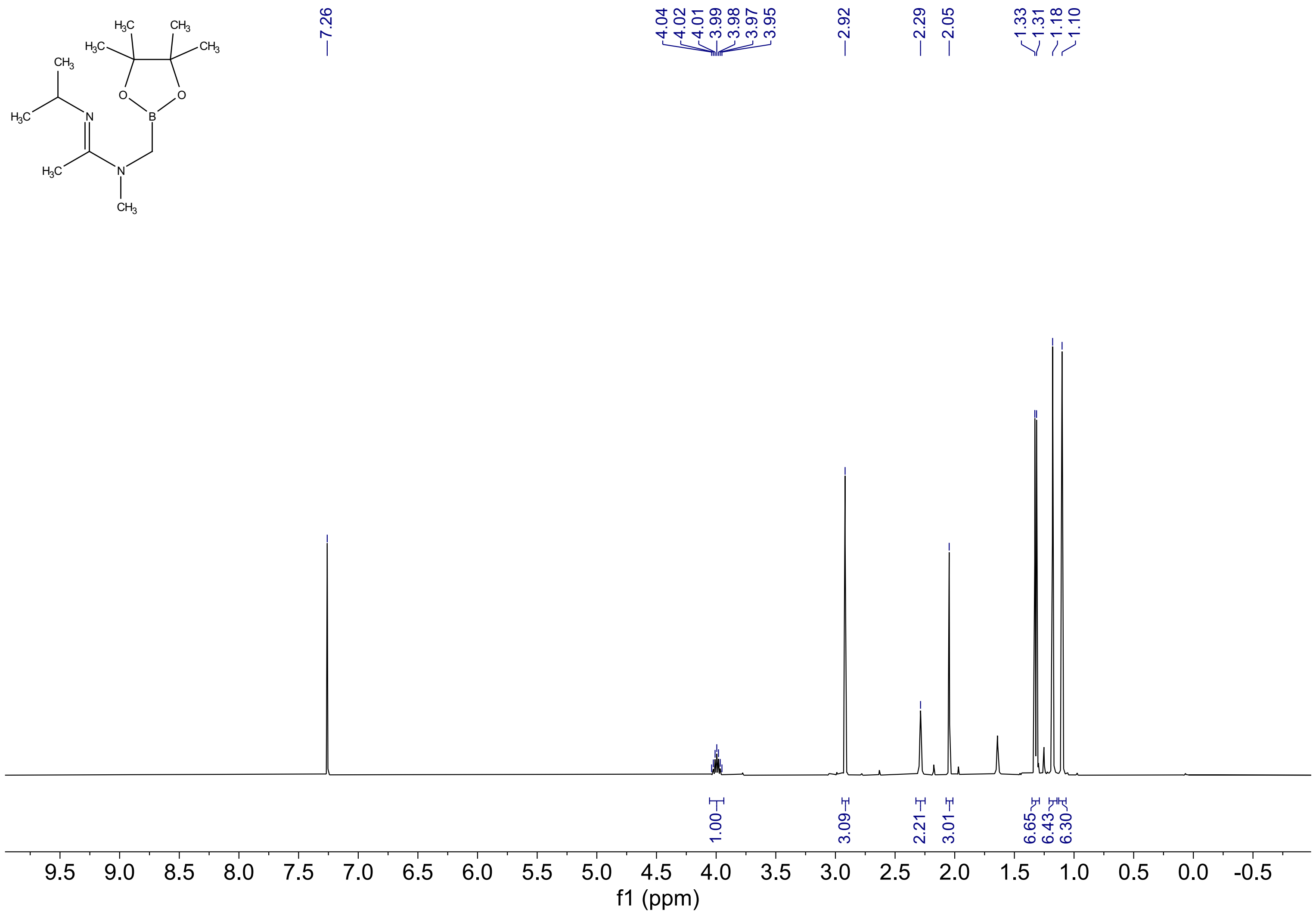

$^{13}C\{^1H\}$ NMR of (*E*)-*N'*-isopropyl-*N*-methyl-*N*-((4,4,5,5-tetramethyl-1,3,2-dioxaborolan-2-yl)methyl)acetimidamide (2f) ($CDCl_3$, 126 MHz)

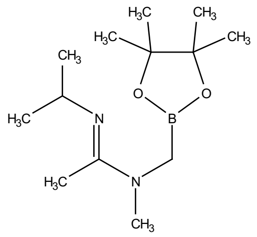

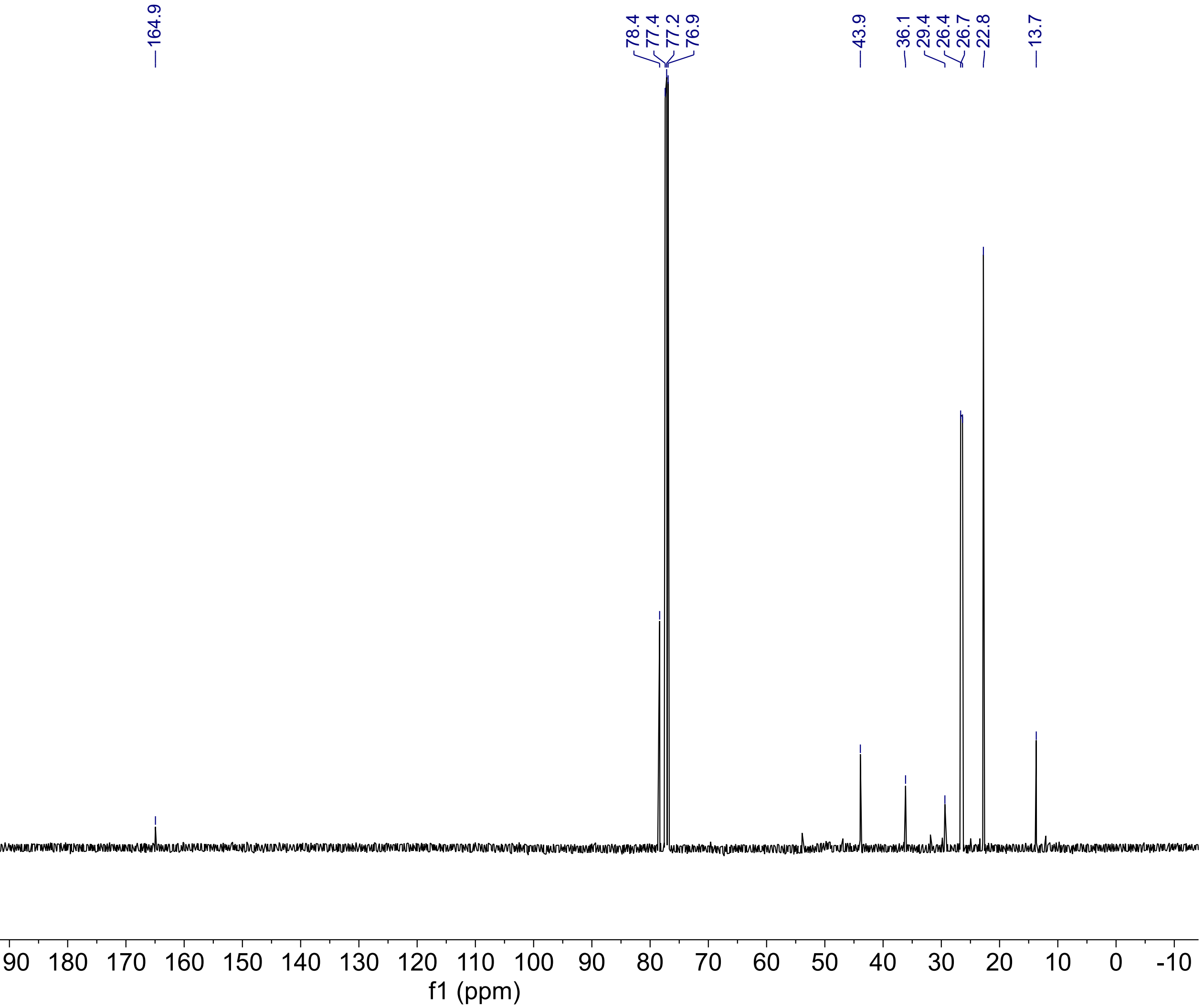

^{11}B NMR of (*E*)-*N'*-isopropyl-*N*-methyl-*N*-((4,4,5,5-tetramethyl-1,3,2-dioxaborolan-2-yl)methyl)acetimidamide (2f) ($CDCl_3$, 160 MHz)

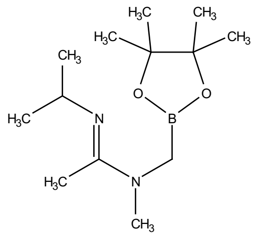

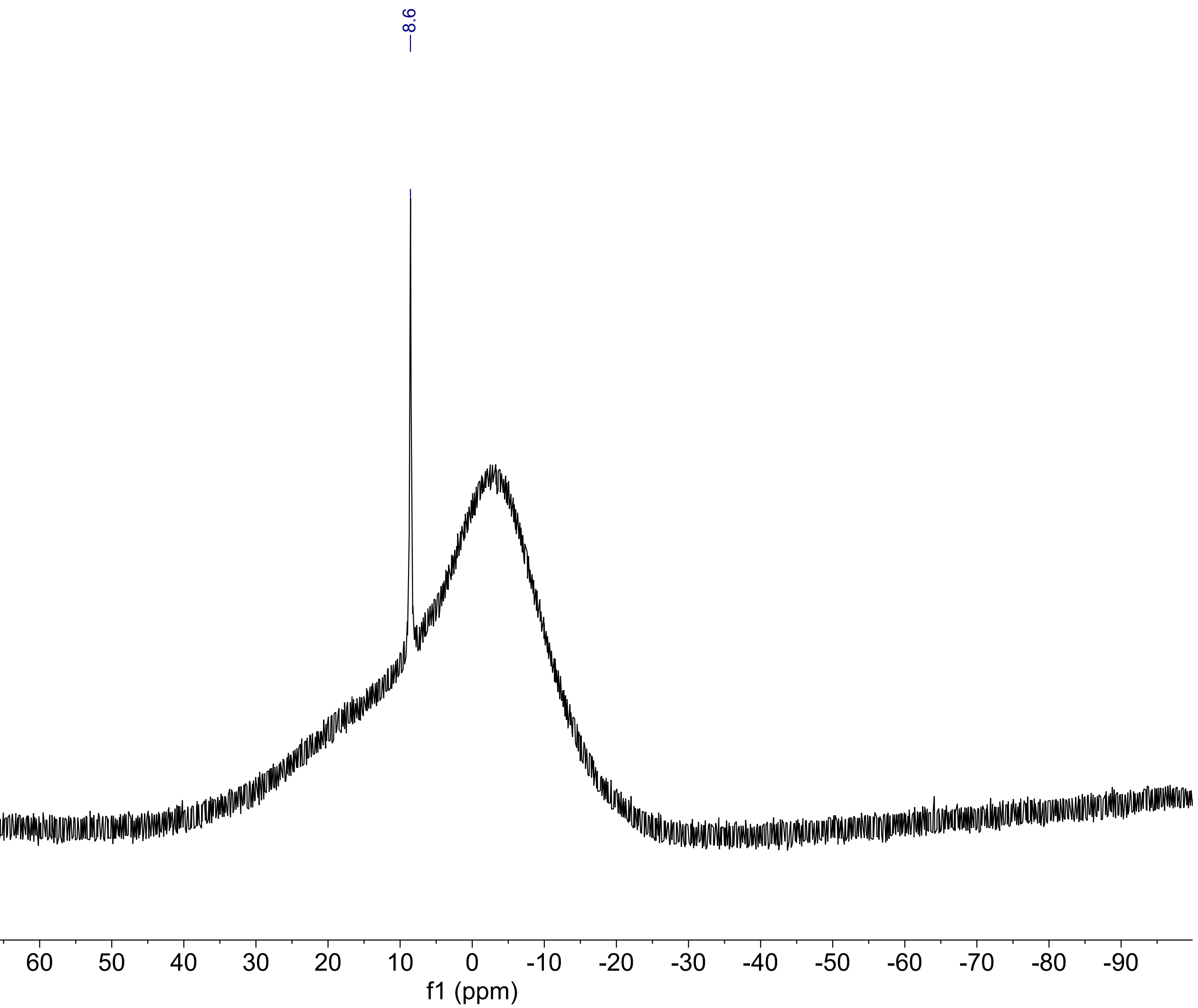

^{1}H NMR of (*E*)-*N'*-(*tert*-butyl)-*N*-methyl-*N*-((4,4,5,5-tetramethyl-1,3,2-dioxaborolan-2-yl)methyl)acetimidamide (2g) ($CDCl_3$, 500 MHz)

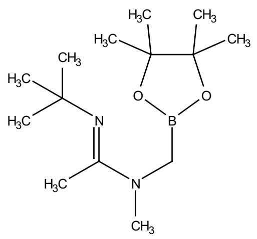

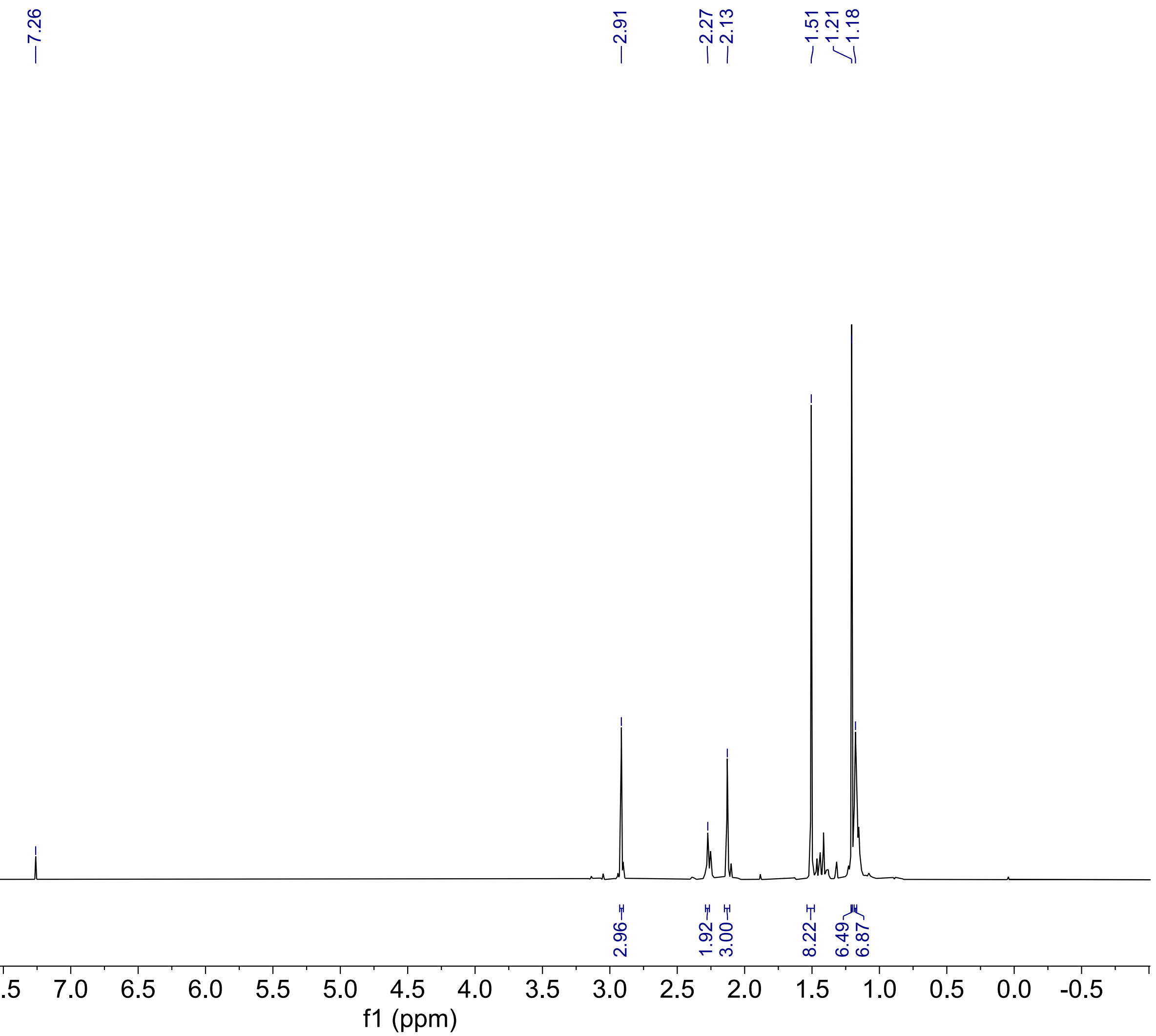

$^{13}C\{^{1}H\}$ NMR of (*E*)-*N'*-(*tert*-butyl)-*N*-methyl-*N*-((4,4,5,5-tetramethyl-1,3,2-dioxaborolan-2-yl)methyl)acetimidamide (2g) ($CDCl_3$, 126 MHz)

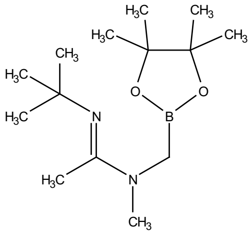

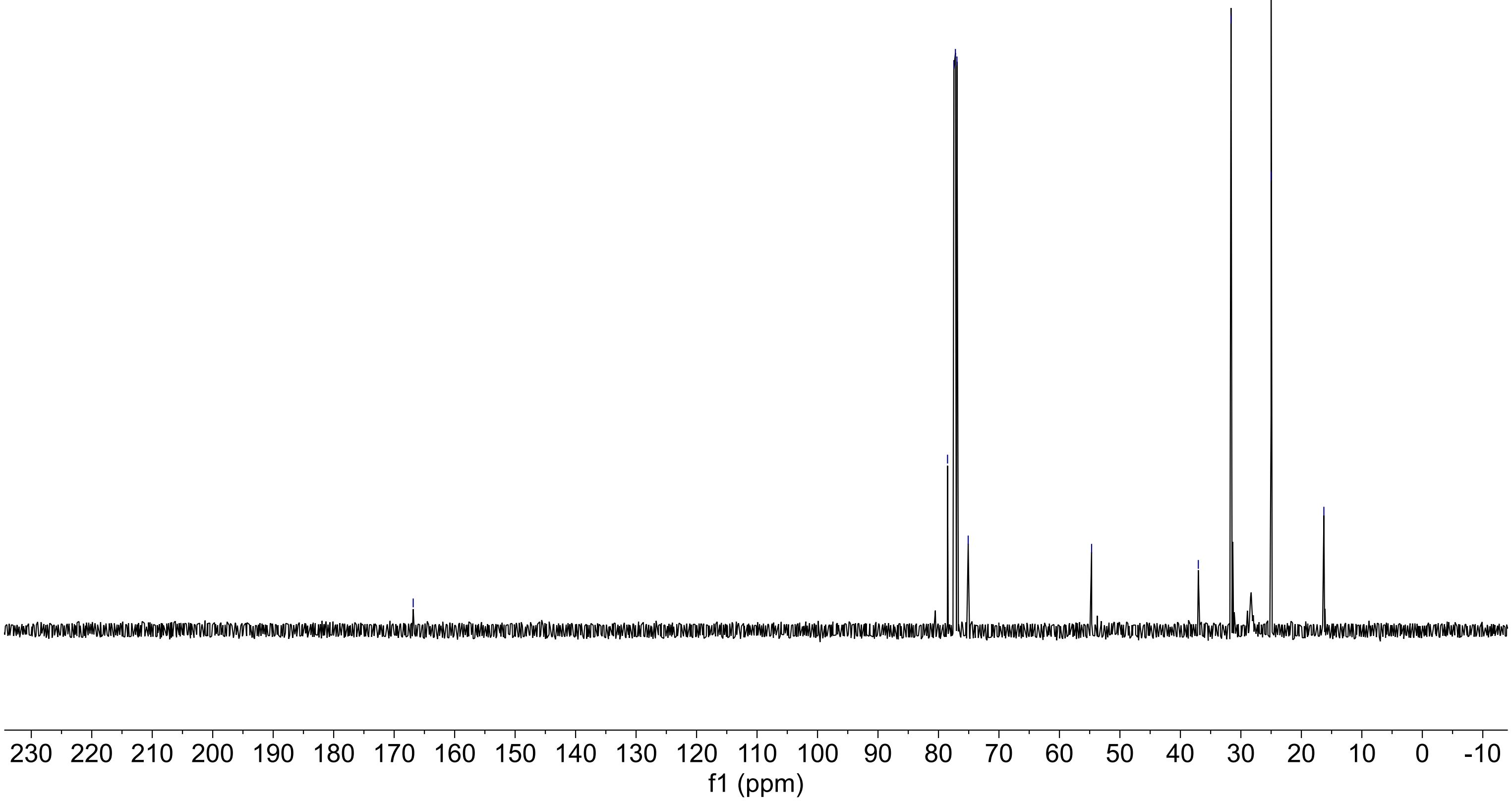

^{11}B NMR of (*E*)-*N'*-(*tert*-butyl)-*N*-methyl-*N*-((4,4,5,5-tetramethyl-1,3,2-dioxaborolan-2-yl)methyl)acetimidamide (2g) ($CDCl_3$, 160 MHz)

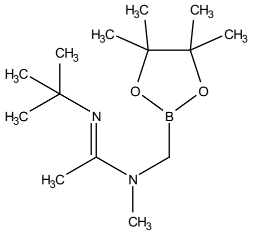

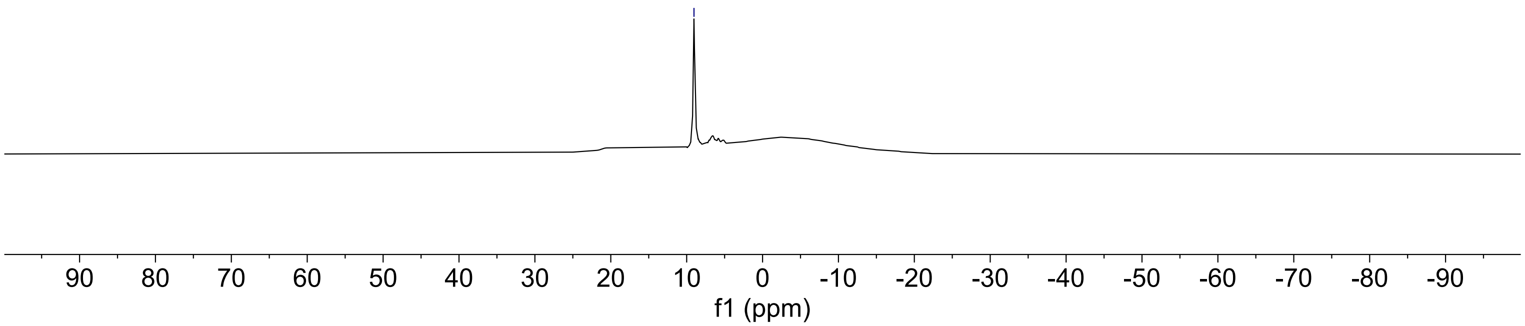

^{1}H NMR of (*E*)-*N'*-isobutyl-*N*-methyl-*N*-((4,4,5,5-tetramethyl-1,3,2-dioxaborolan-2-yl)methyl)acetimidamide (2h) ($CDCl_3$, 500 MHz)

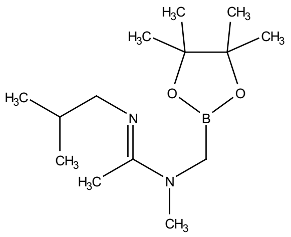

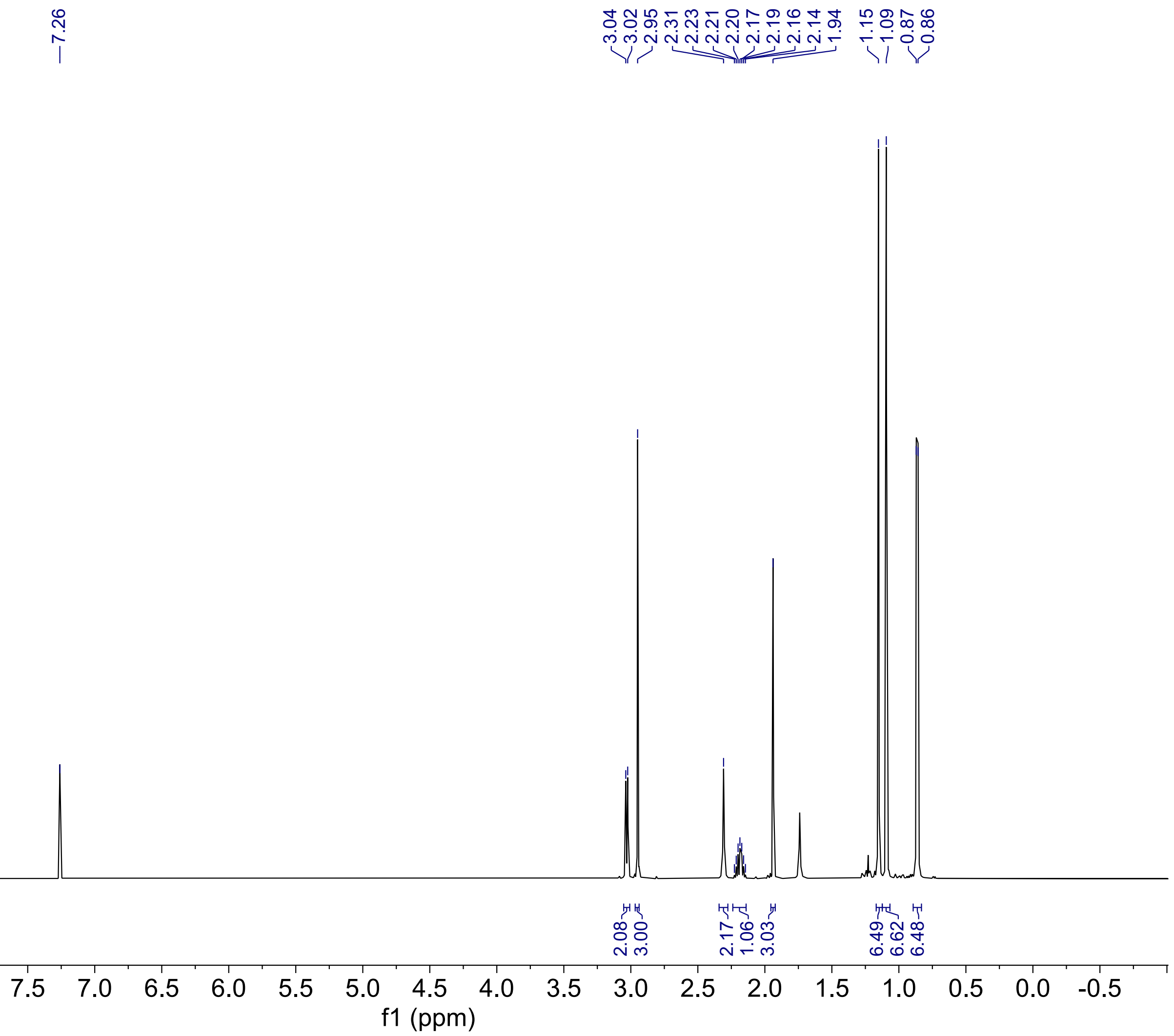

$^{13}C\{^1H\}$ NMR of (*E*)-*N'*-isobutyl-*N*-methyl-*N*-((4,4,5,5-tetramethyl-1,3,2-dioxaborolan-2-yl)methyl)acetimidamide (2h) ($CDCl_3$, 126 MHz)

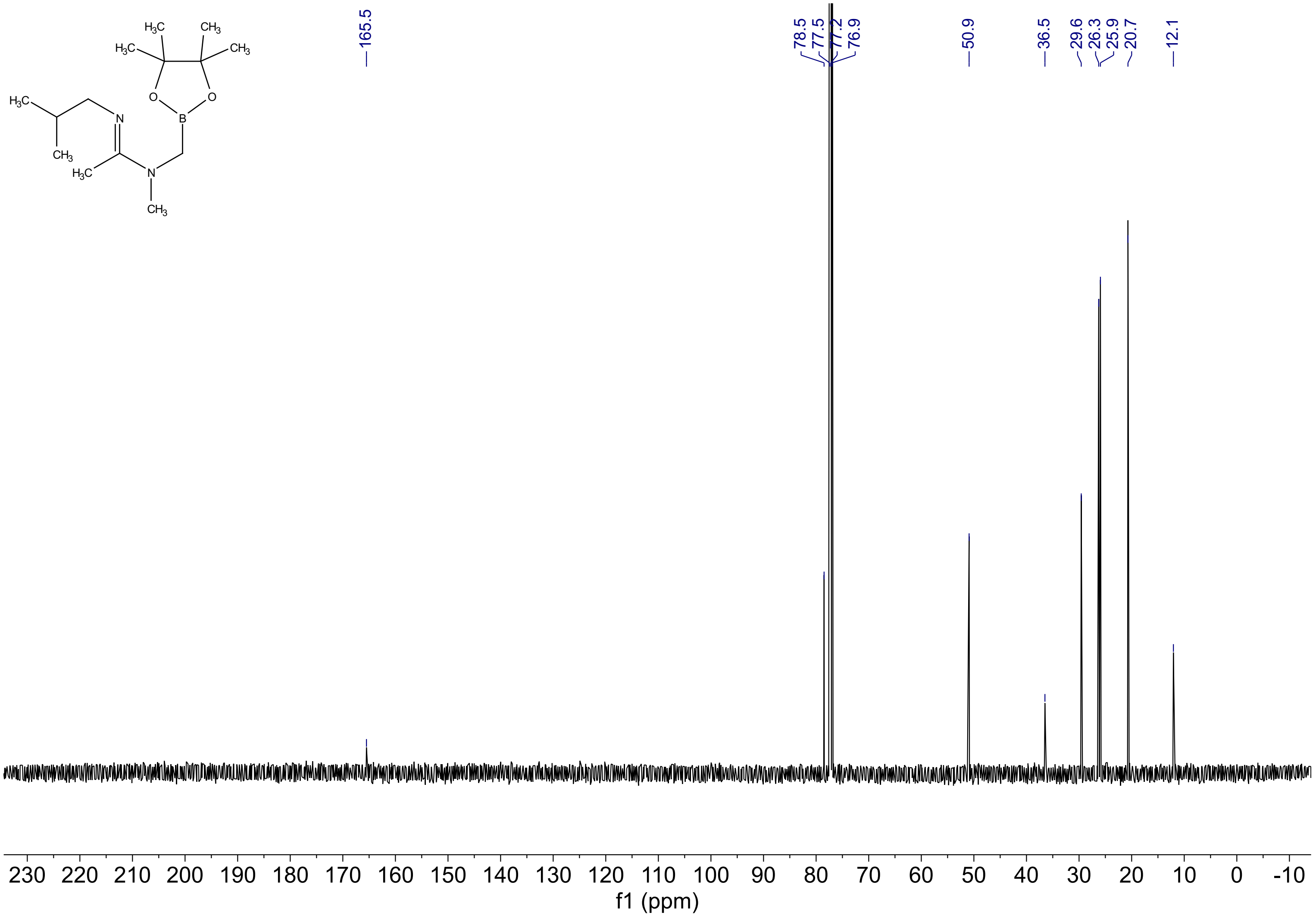

^{11}B NMR of (*E*)-*N'*-isobutyl-*N*-methyl-*N*-((4,4,5,5-tetramethyl-1,3,2-dioxaborolan-2-yl)methyl)acetimidamide (2h) ($CDCl_3$, 160 MHz)

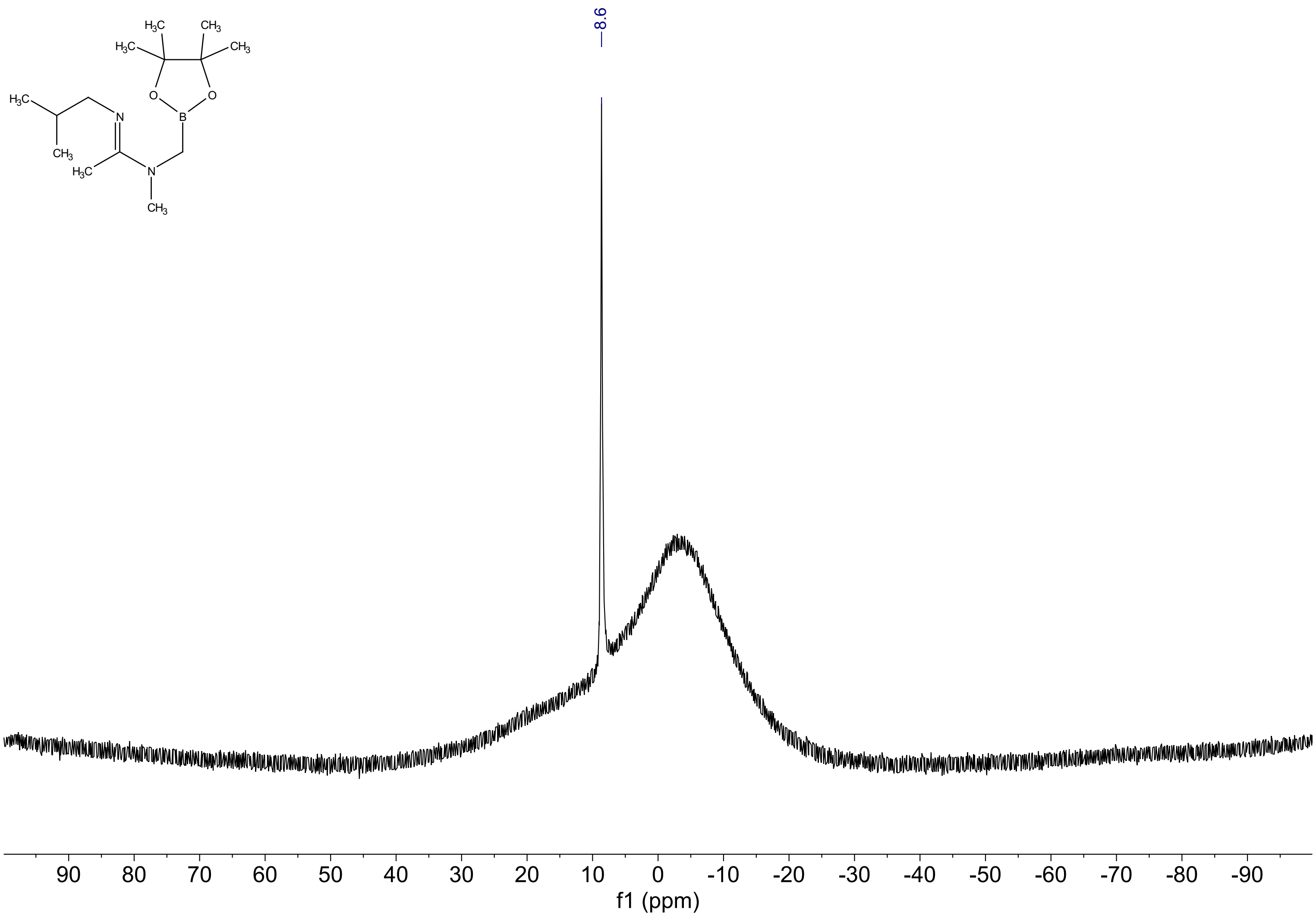

^{1}H NMR of *N*-methyl-*N*-((4,4,5,5-tetramethyl-1,3,2-dioxaborolan-2-yl)methyl)-3,4-dihydro-2*H*-pyrrol-5-amine (2i) ($CDCl_3$, 500 MHz)

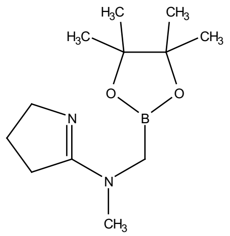

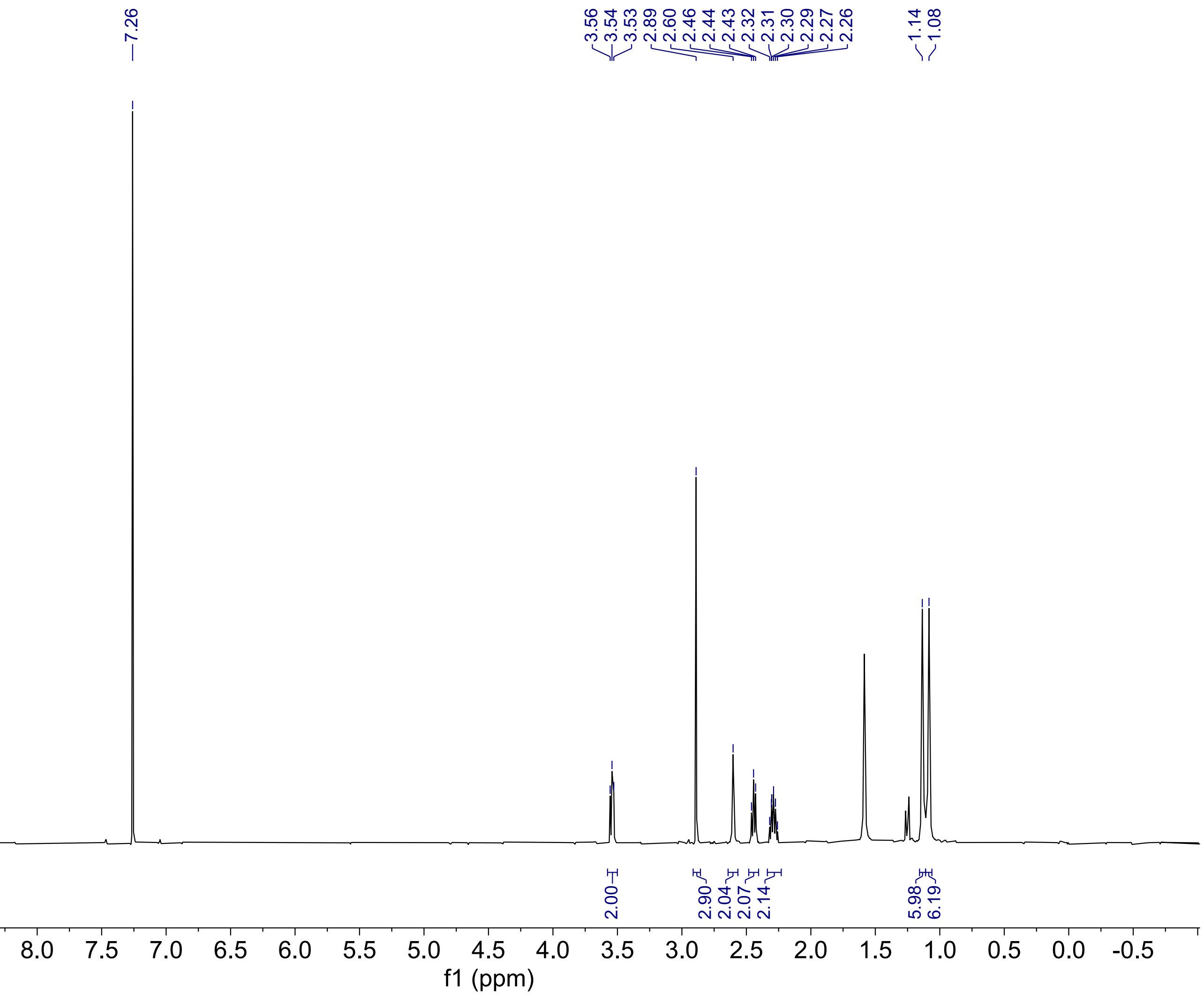

$^{13}C\{^1H\}$ NMR of *N*-methyl-*N*-((4,4,5,5-tetramethyl-1,3,2-dioxaborolan-2-yl)methyl)-3,4-dihydro-2*H*-pyrrol-5-amine (2i) ($CDCl_3$, 126 MHz)

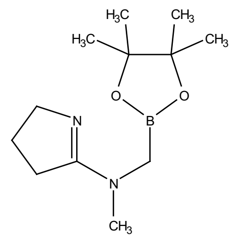

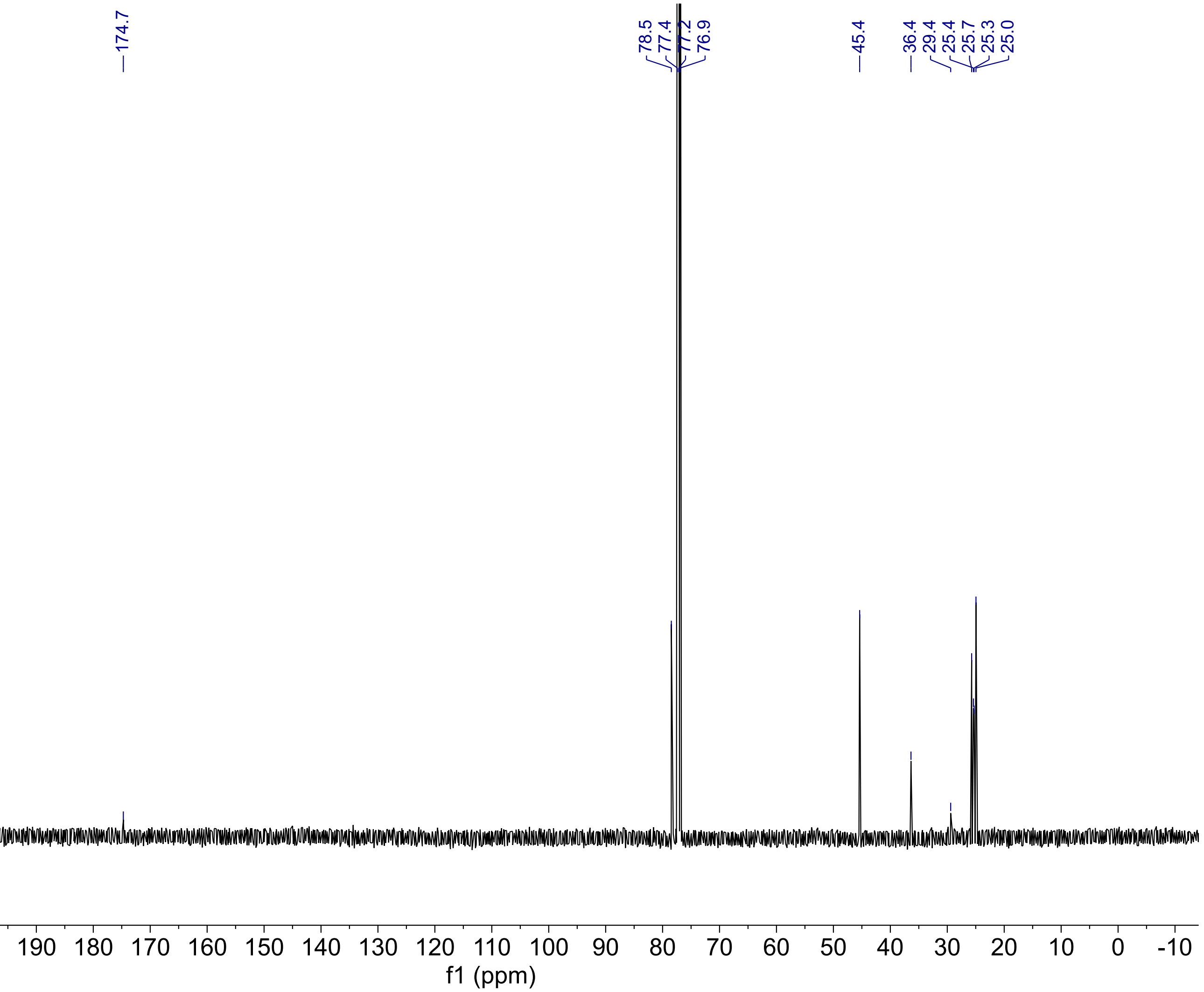

^{11}B NMR of *N*-methyl-*N*-((4,4,5,5-tetramethyl-1,3,2-dioxaborolan-2-yl)methyl)-3,4-dihydro-2*H*-pyrrol-5-amine (2i) ($CDCl_3$, 160 MHz)

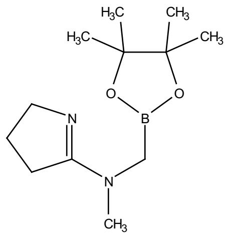

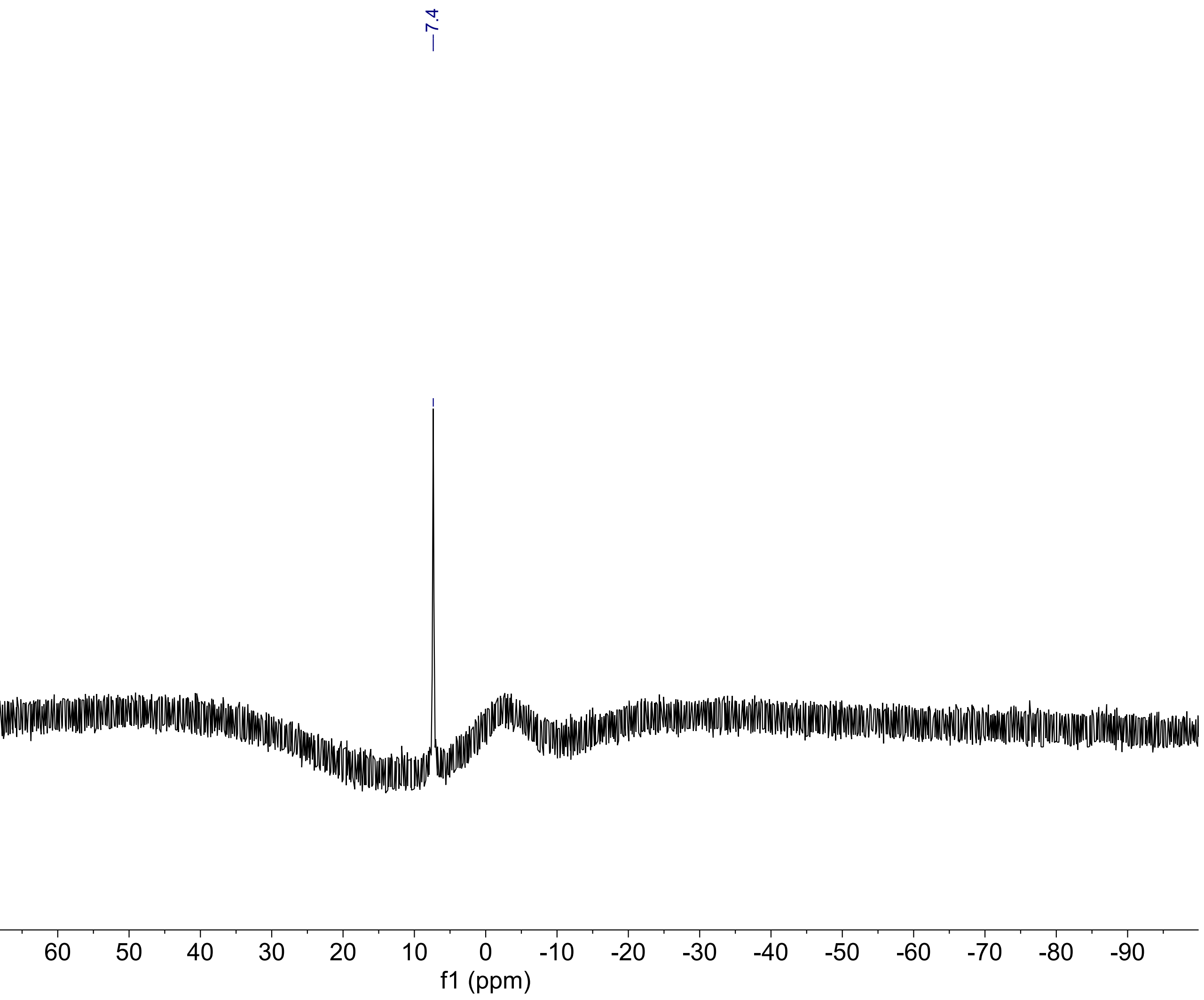

^{1}H NMR of *N*-methyl-*N*-((4,4,5,5-tetramethyl-1,3,2-dioxaborolan-2-yl)methyl)-3,4,5,6-tetrahydropyridin-2-amine (2j) ($CDCl_3$, 500 MHz)

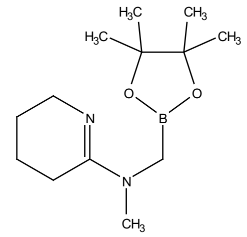

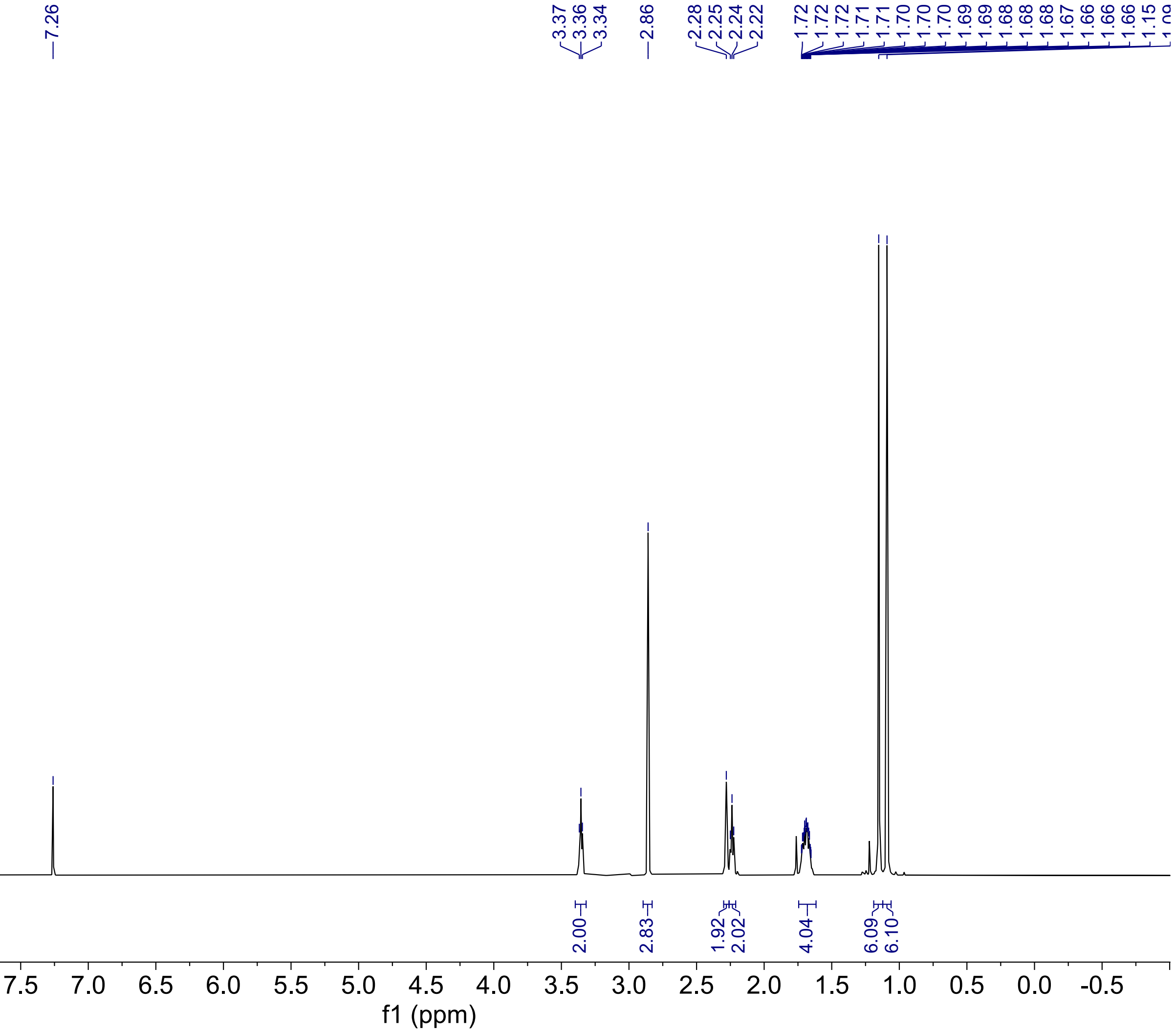

$^{13}C\{^1H\}$ NMR of *N*-methyl-*N*-((4,4,5,5-tetramethyl-1,3,2-dioxaborolan-2-yl)methyl)-3,4,5,6-tetrahydropyridin-2-amine (2j) ($CDCl_3$, 126 MHz)

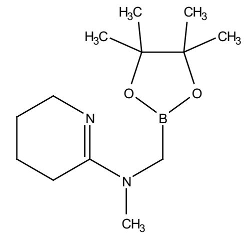

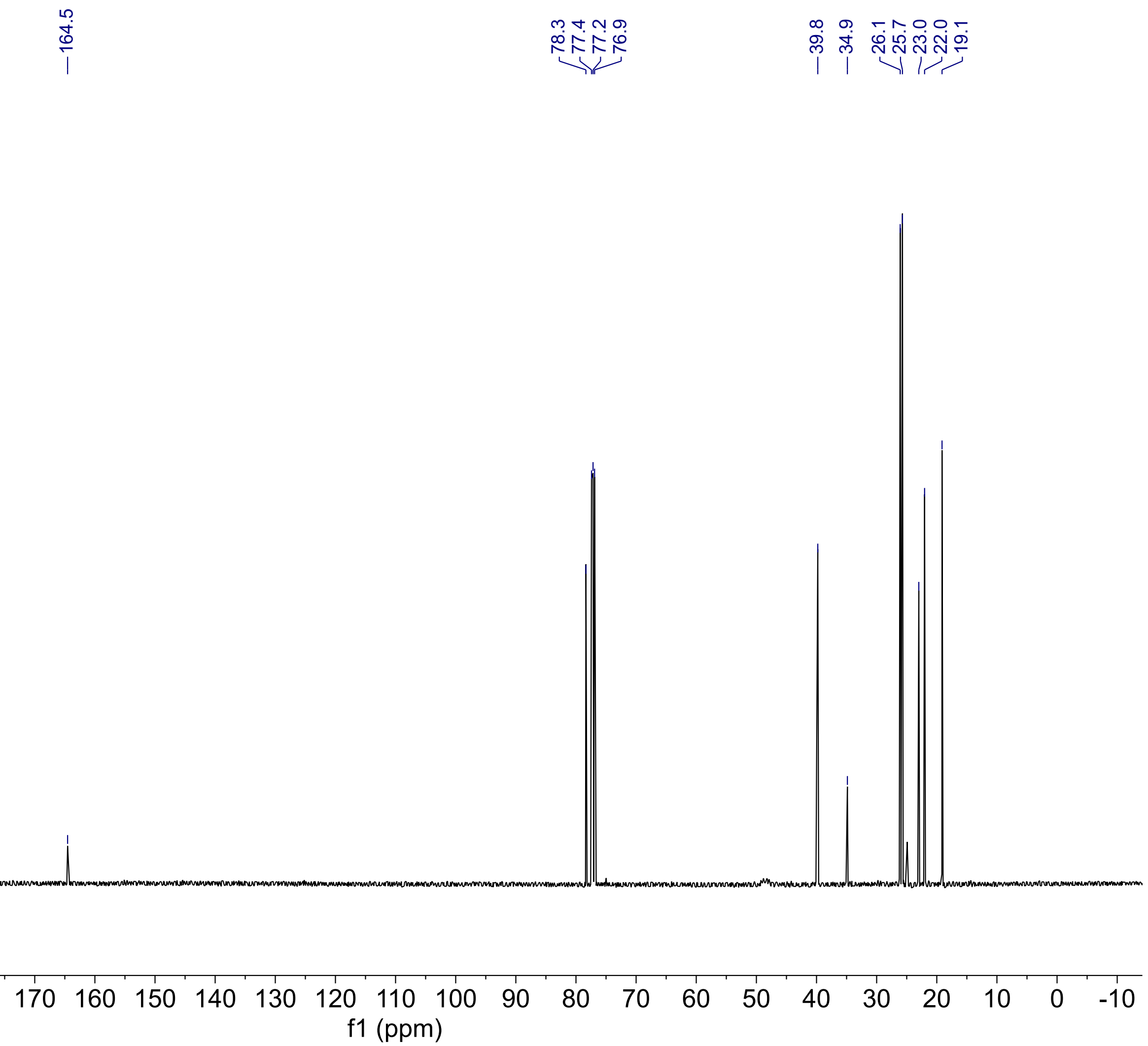

^{11}B NMR of *N*-methyl-*N*-((4,4,5,5-tetramethyl-1,3,2-dioxaborolan-2-yl)methyl)-3,4,5,6-tetrahydropyridin-2-amine (2j) ($CDCl_3$, 160 MHz)

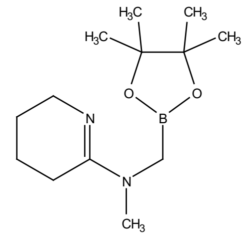

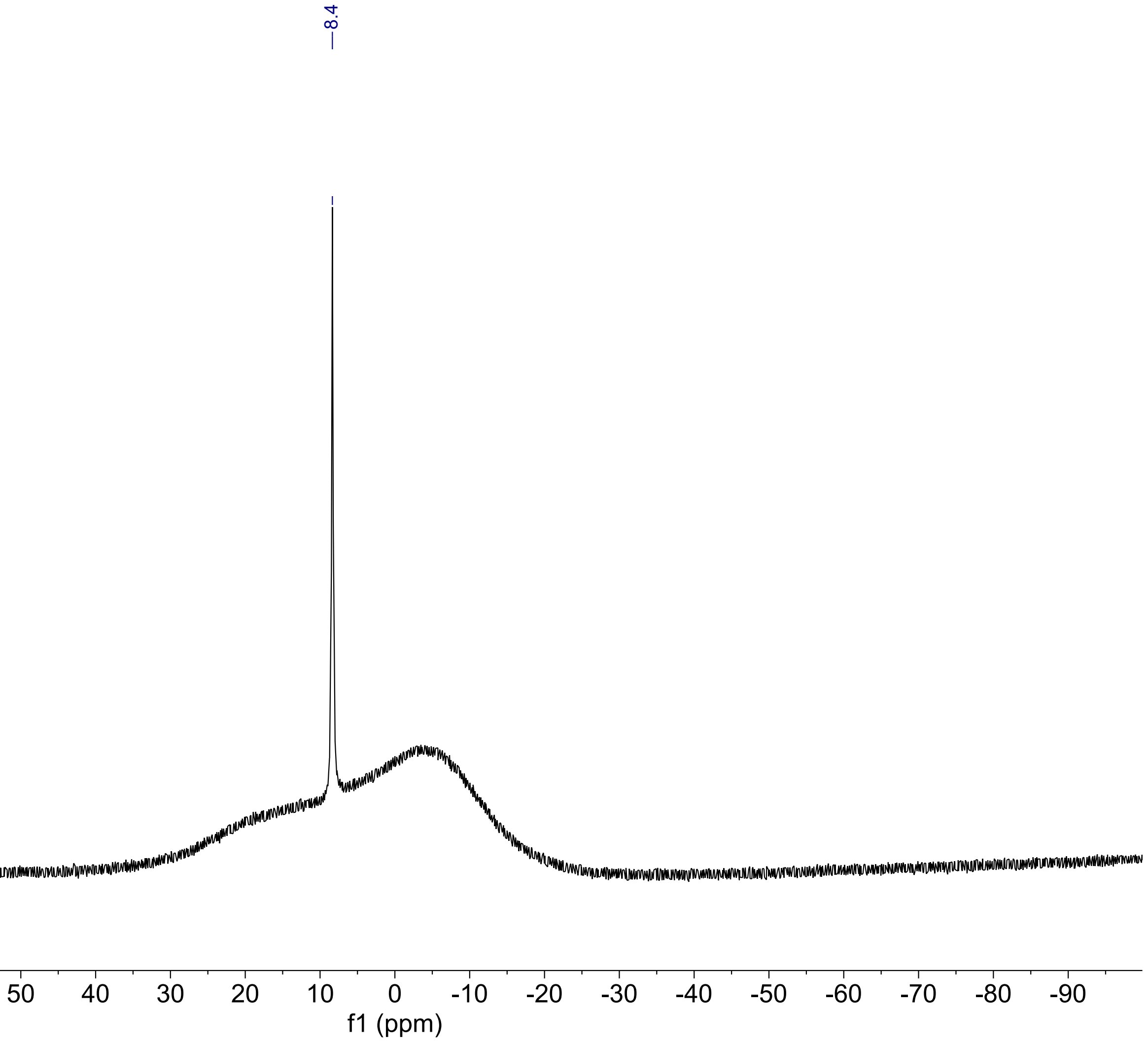

^{1}H NMR of *N*-methyl-*N*-((4,4,5,5-tetramethyl-1,3,2-dioxaborolan-2-yl)methyl)-3,4,5,6-tetrahydro-*2H*-azepin-7-amine (2k) ($CDCl_3$, 500 MHz)

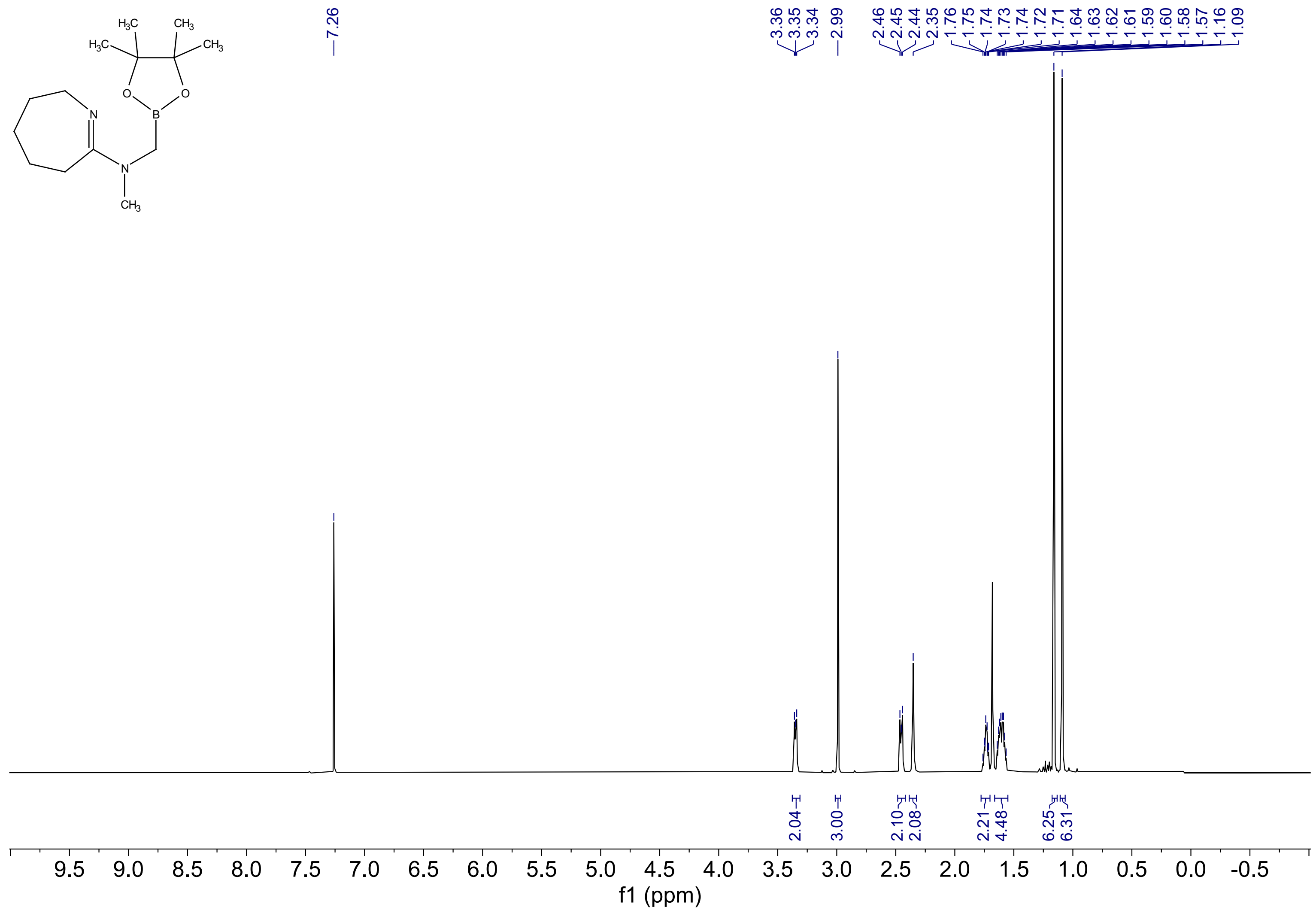

$^{13}C\{^{1}H\}$ NMR of *N*-methyl-*N*-((4,4,5,5-tetramethyl-1,3,2-dioxaborolan-2-yl)methyl)-3,4,5,6- tetrahydro-*2H*-azepin-7-amine (2k) ($CDCl_3$, 126 MHz)

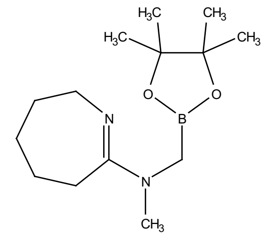

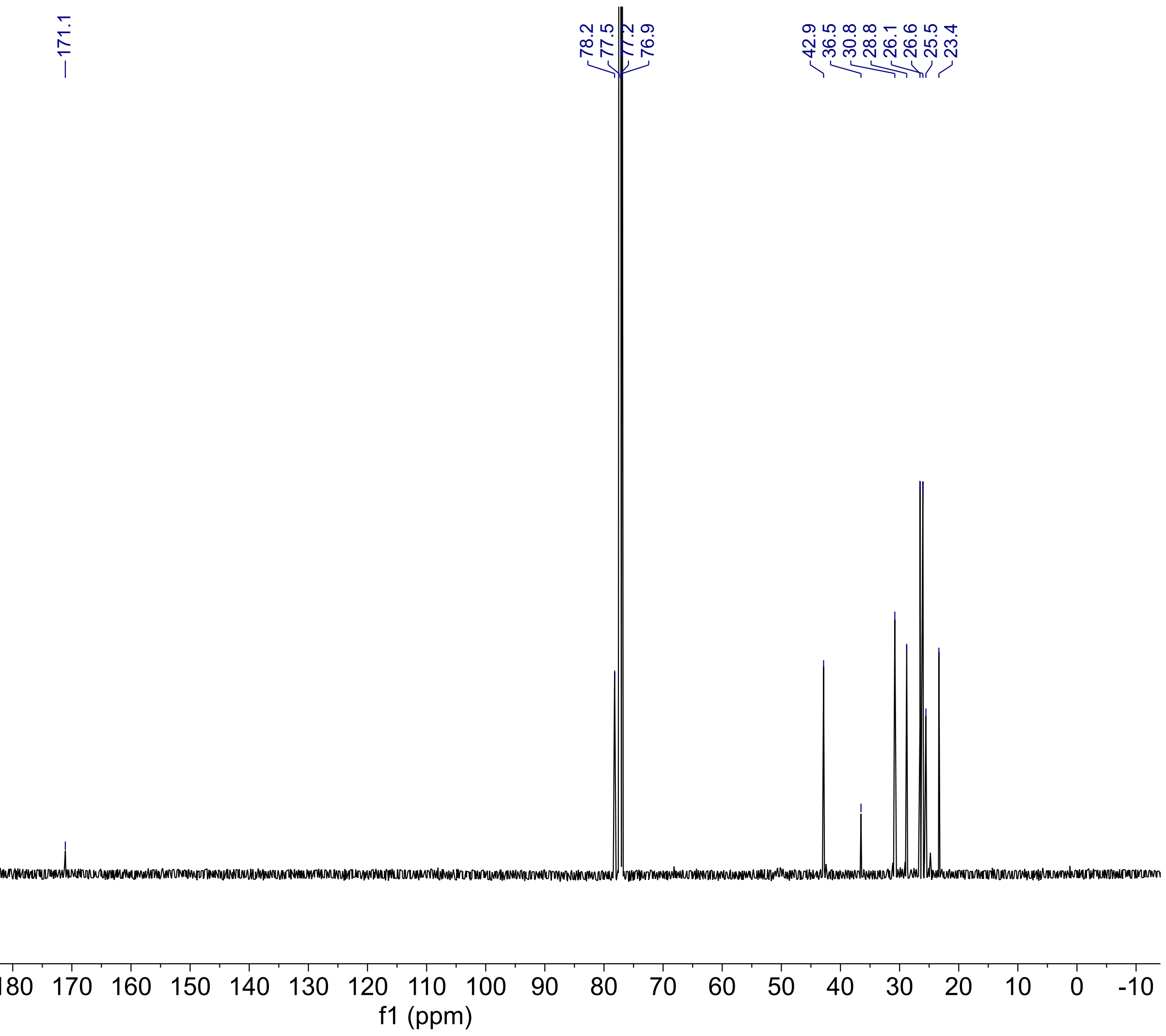

^{11}B NMR of *N*-methyl-*N*-((4,4,5,5-tetramethyl-1,3,2-dioxaborolan-2-yl)methyl)-3,4,5,6- tetrahydro-*2H*-azepin-7-amine (2k) ($CDCl_3$, 160 MHz)

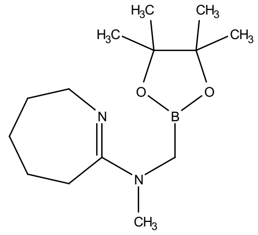

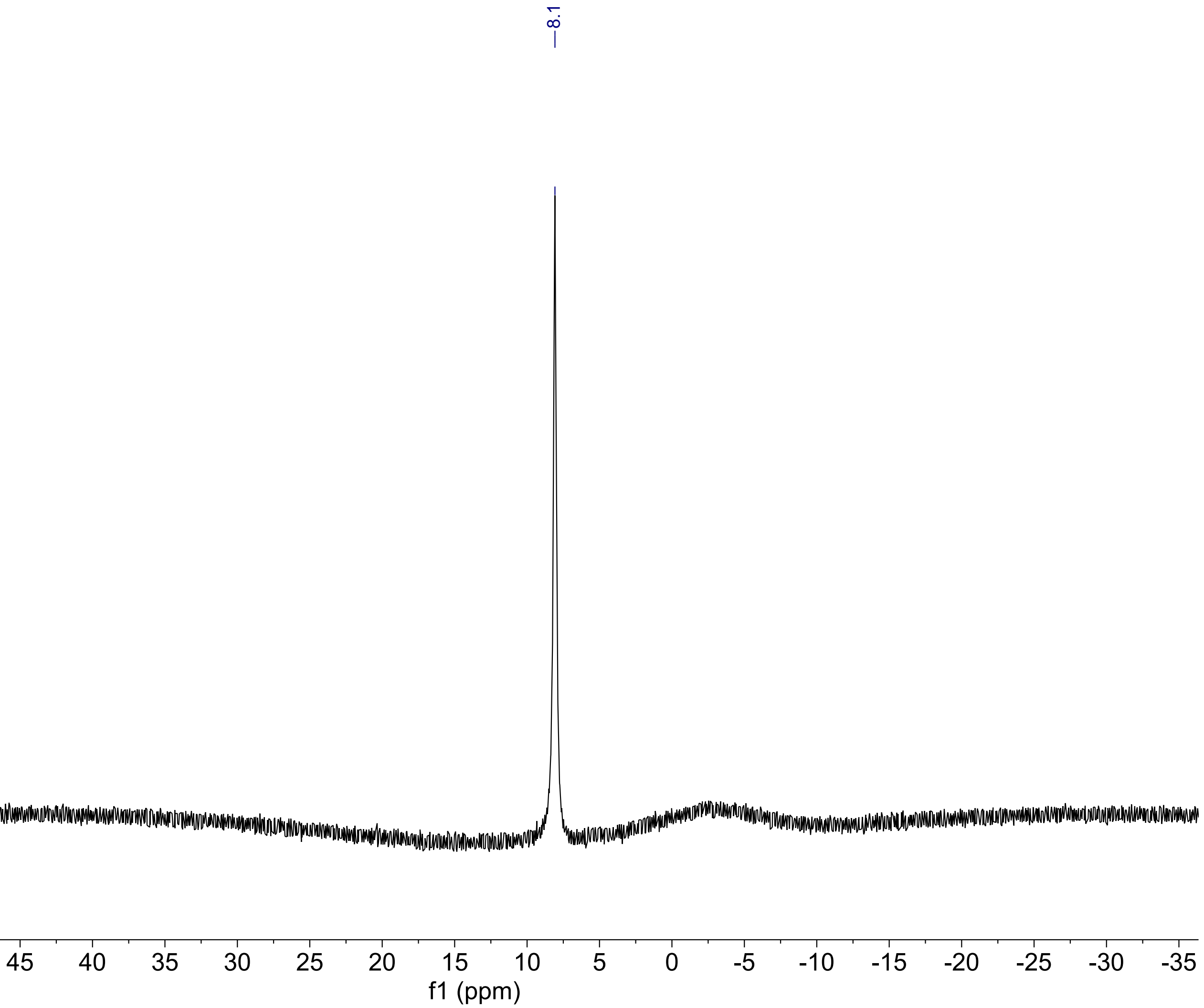

^{1}H NMR of *N'*-cyclohexyl-*N*-methyl-*N*-((4,4,5,5-tetramethyl-1,3,2-dioxaborolan-2-yl)methyl)acetimidamide (2l) ($CDCl_3$, 500 MHz)

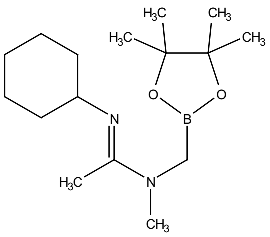

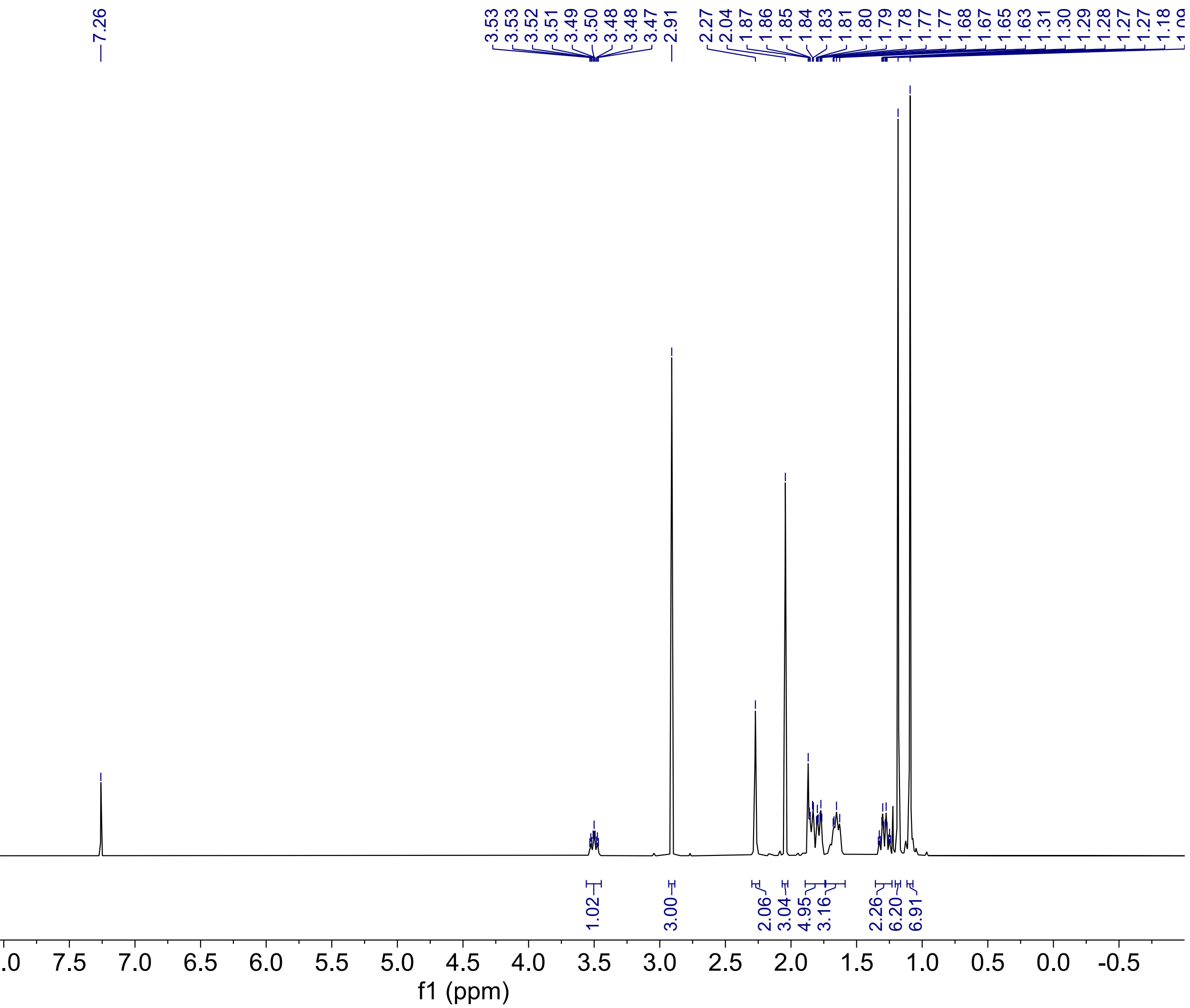

$^{13}C\{^1H\}$ NMR of *N'*-cyclohexyl-*N*-methyl-*N*-((4,4,5,5-tetramethyl-1,3,2-dioxaborolan-2-yl)methyl)acetimidamide (2l) ($CDCl_3$, 126 MHz)

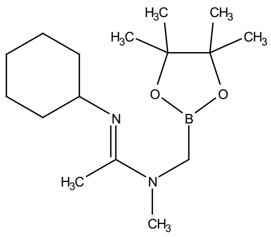

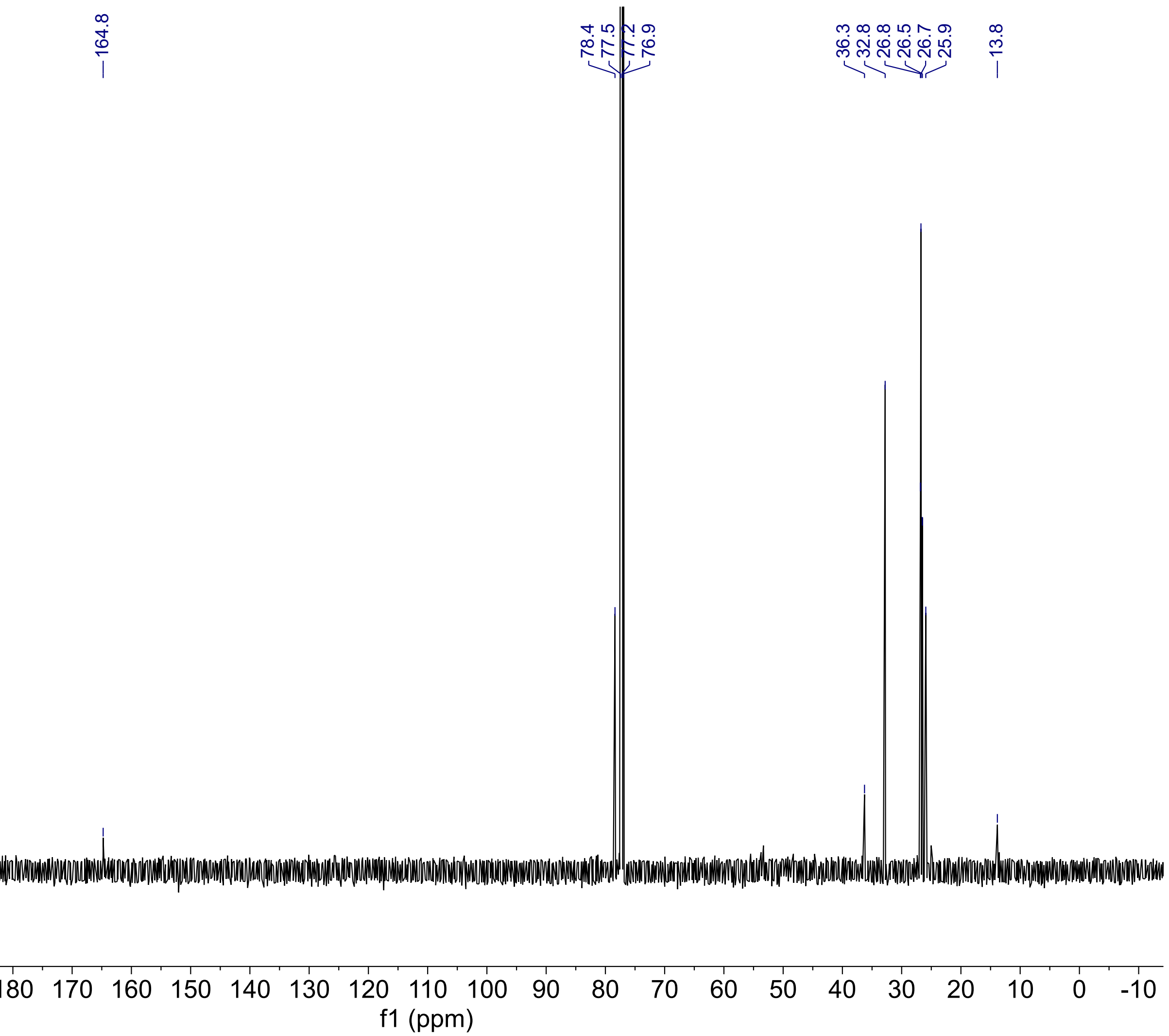

^{11}B NMR of *N'*-cyclohexyl-*N*-methyl-*N*-((4,4,5,5-tetramethyl-1,3,2-dioxaborolan-2-yl)methyl)acetimidamide (2l) ($CDCl_3$, 160 MHz)

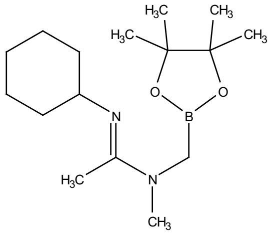

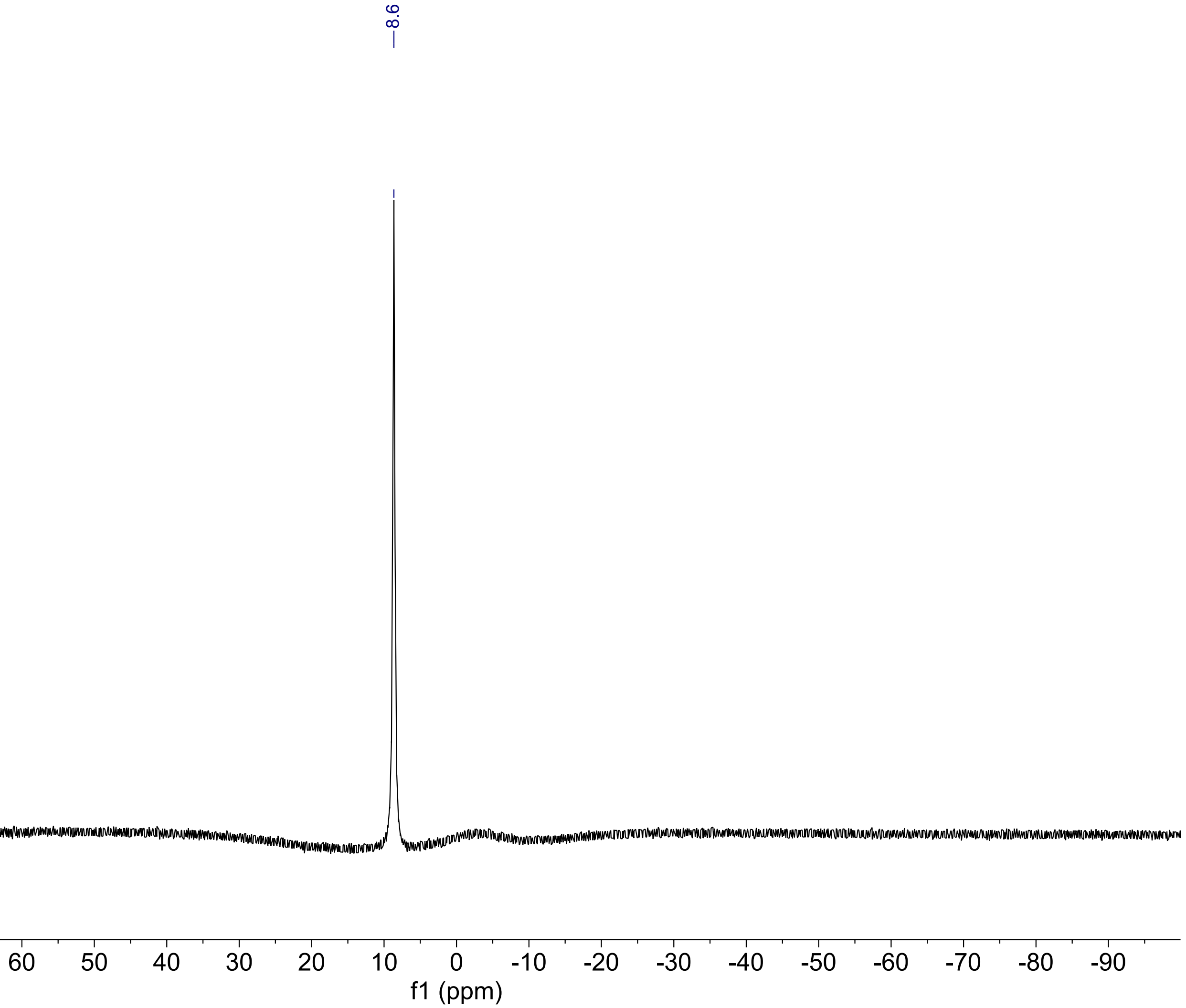

^{1}H NMR of *N'*-benzyl-*N*-methyl-*N*-((4,4,5,5-tetramethyl-1,3,2-dioxaborolan-2-yl)methyl)acetimidamide (2m) ($CDCl_3$, 500 MHz)

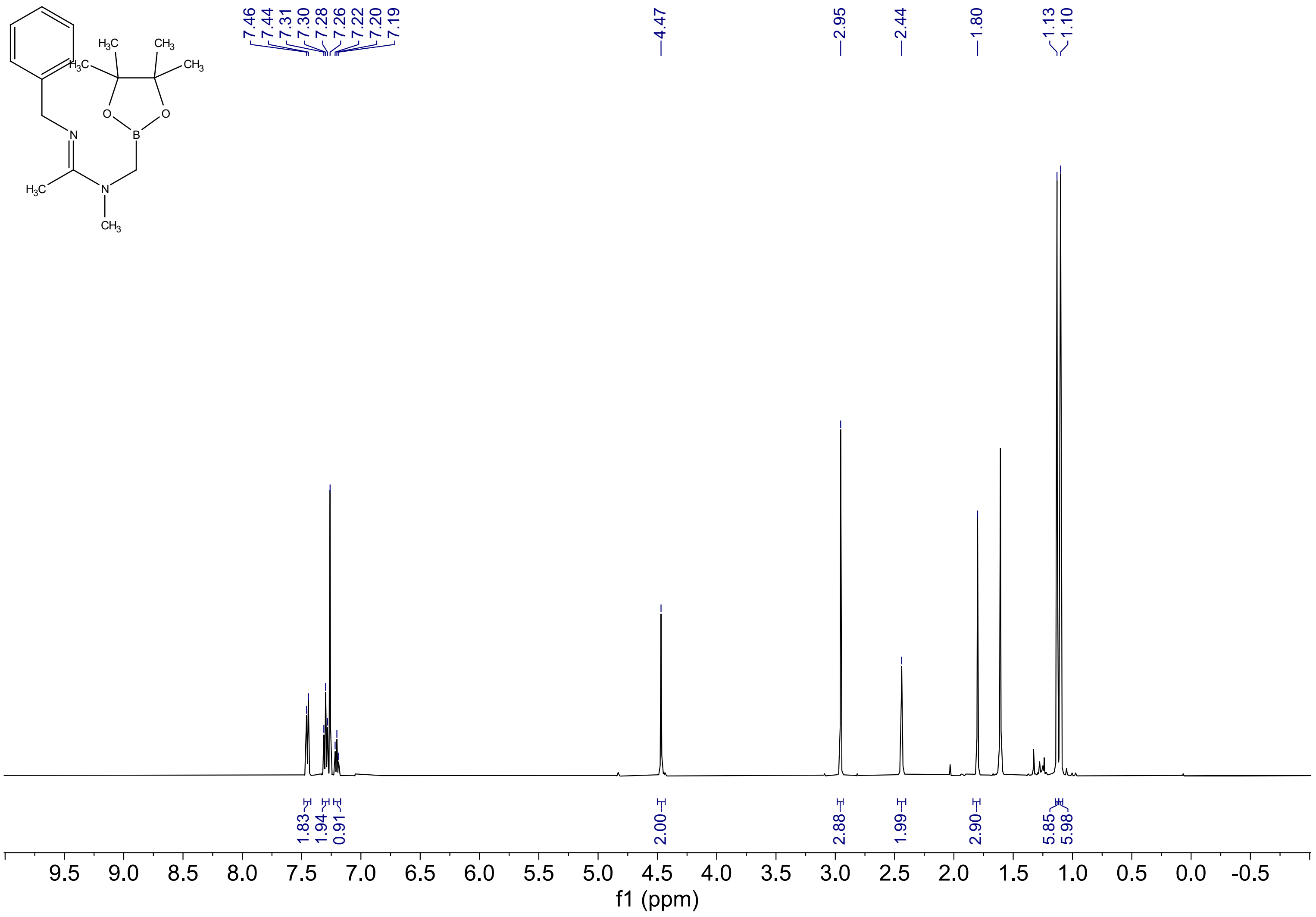

$^{13}C\{^{1}H\}$ NMR of *N'*-benzyl-*N*-methyl-*N*-((4,4,5,5-tetramethyl-1,3,2-dioxaborolan-2-yl)methyl)acetimidamide (2m) ($CDCl_3$, 126 MHz)

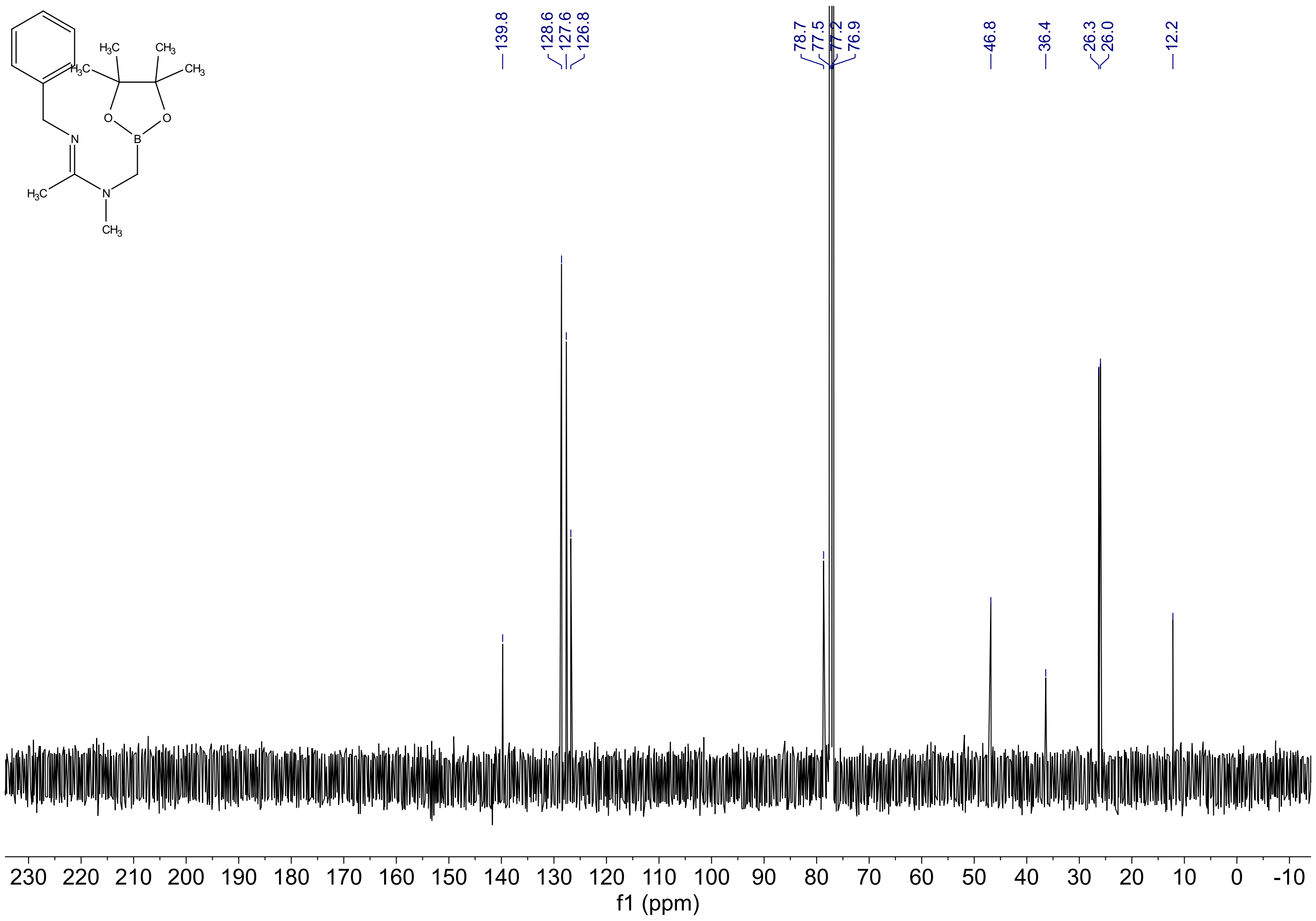

^{11}B NMR of *N'*-benzyl-*N*-methyl-*N*-((4,4,5,5-tetramethyl-1,3,2-dioxaborolan-2-yl)methyl)acetimidamide (2m) ($CDCl_3$, 160 MHz)

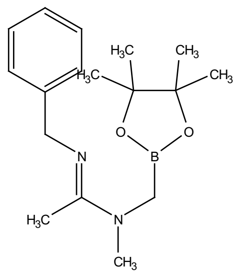

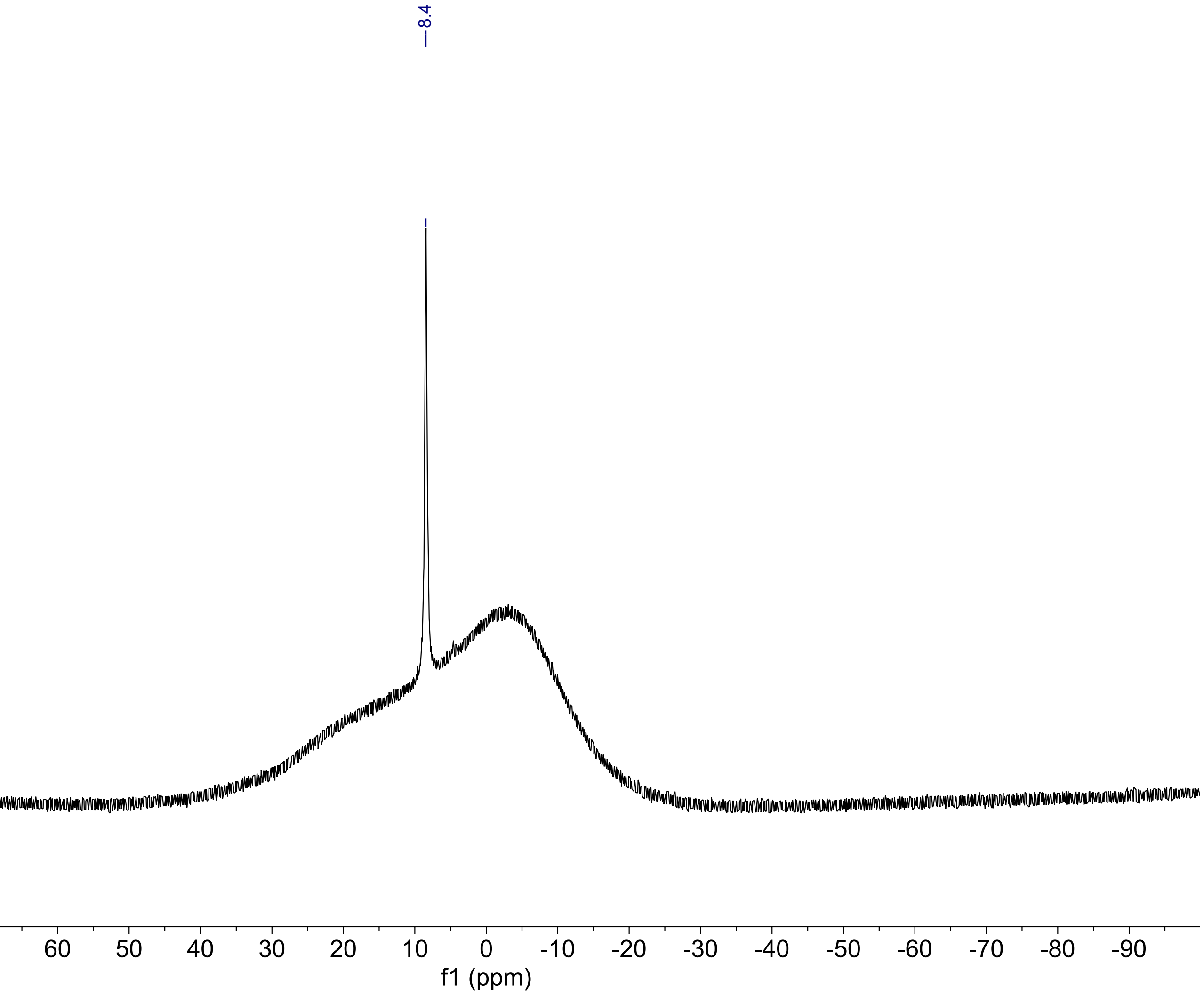

^{1}H NMR of *N'*-methyl-*N*-phenyl-*N*-((4,4,5,5-tetramethyl-1,3,2-dioxaborolan-2-yl)methyl)acetimidamide (2n) ($CDCl_3$, 500 MHz)

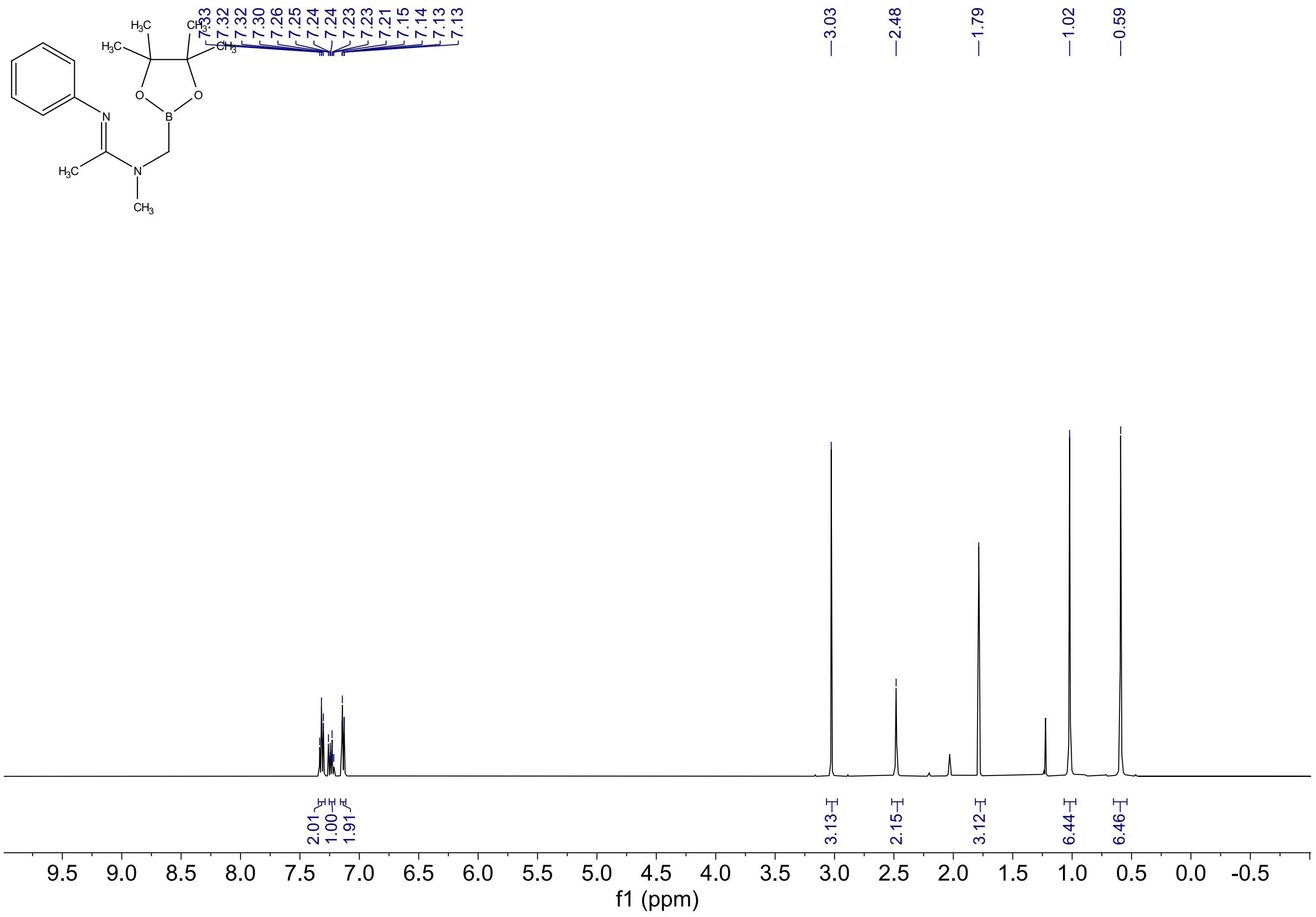

$^{13}C\{^1H\}$ NMR of *N'*-methyl-*N*-phenyl-*N*-((4,4,5,5-tetramethyl-1,3,2-dioxaborolan-2-yl)methyl)acetimidamide (2n) ($CDCl_3$, 126 MHz)

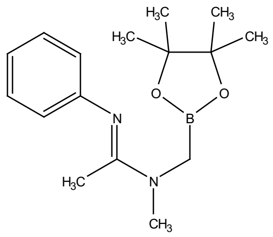

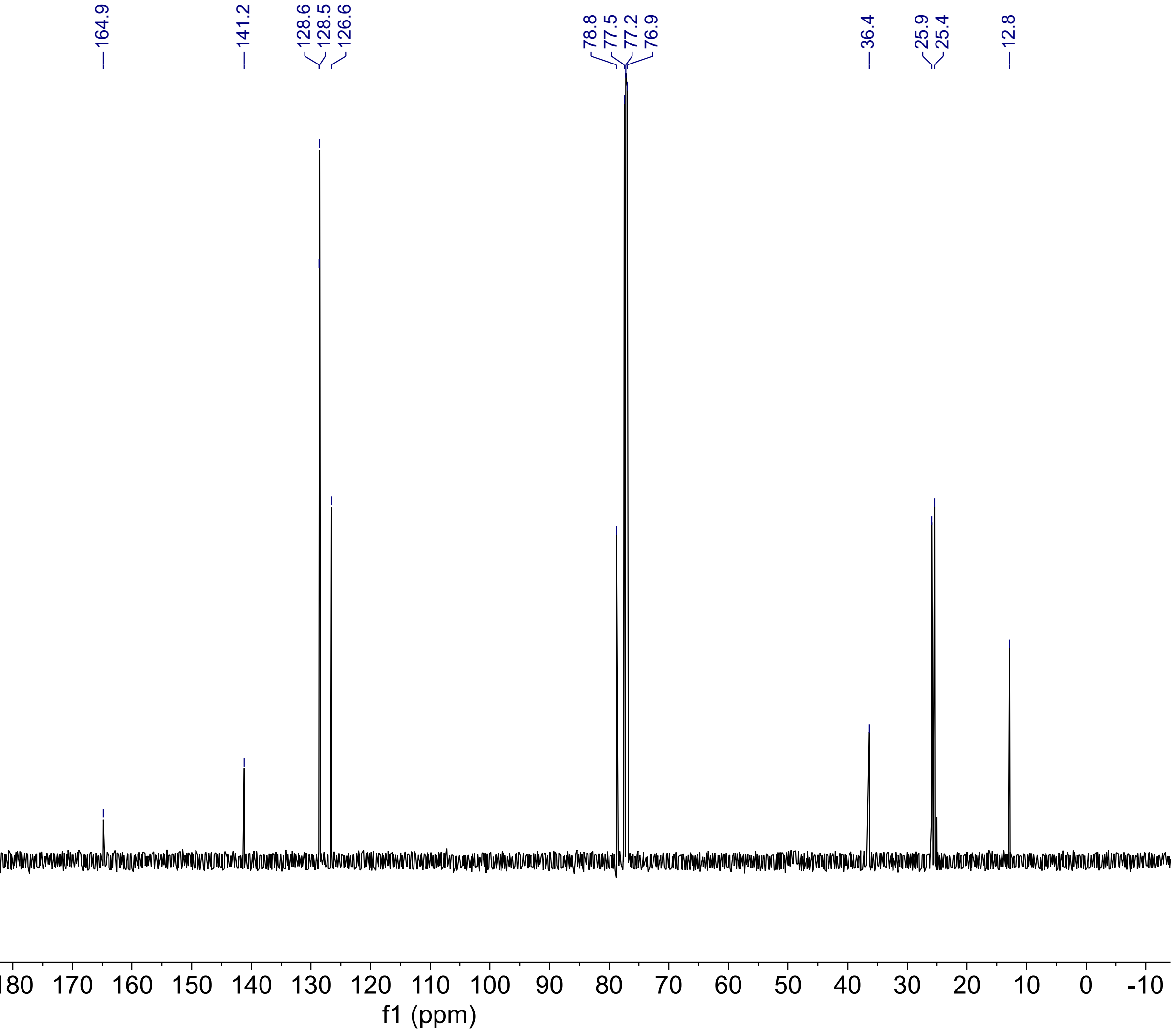

^{11}B NMR of *N'*-methyl-*N*-phenyl-*N*-((4,4,5,5-tetramethyl-1,3,2-dioxaborolan-2-yl)methyl)acetimidamide (2n) ($CDCl_3$, 160 MHz)

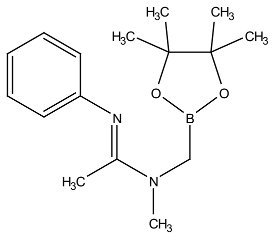

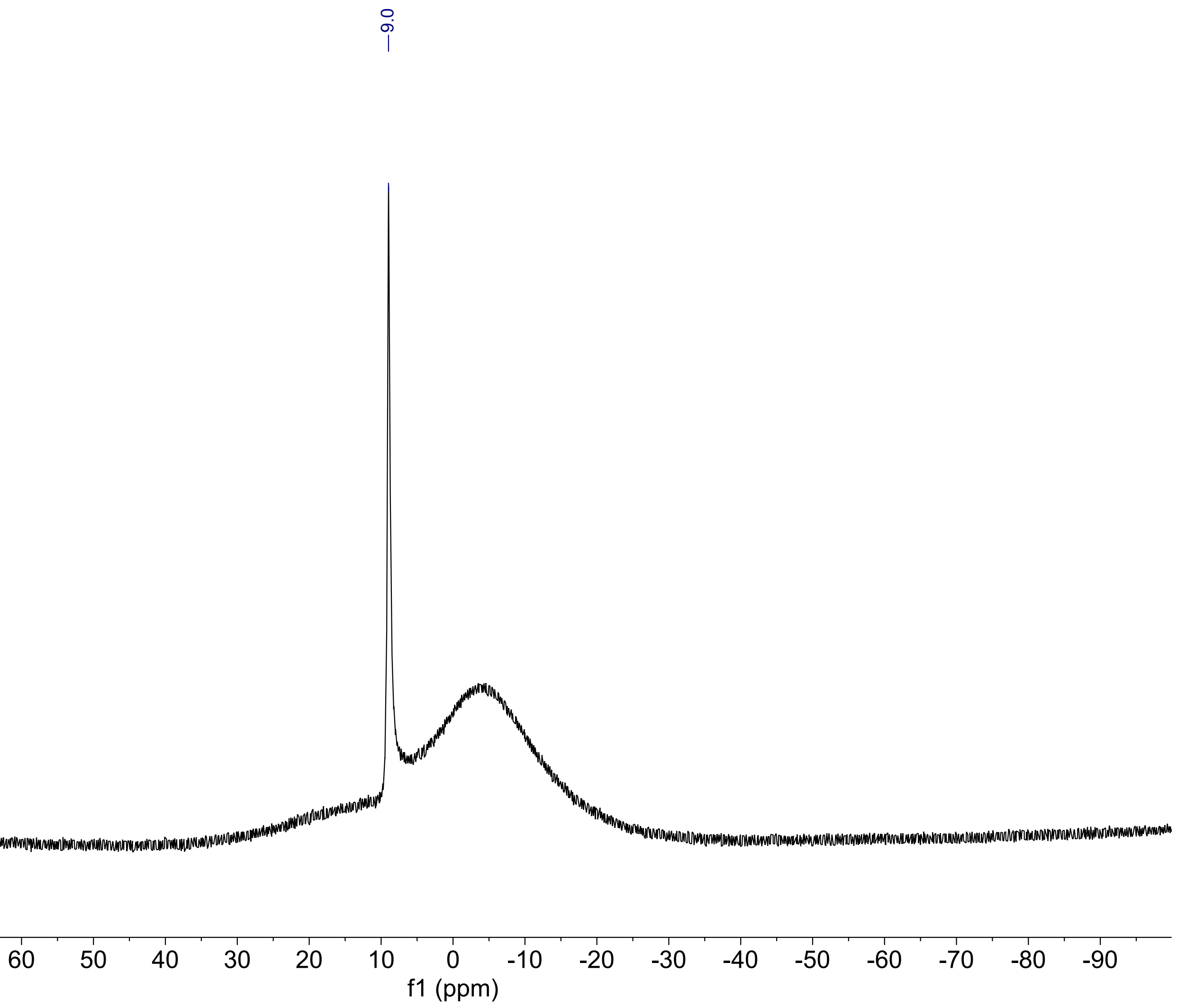

^{1}H NMR of (*E*)-*N*,*N*-diethyl-*N'*-methylacetimidamide (3a) ($CDCl_3$, 500 MHz)

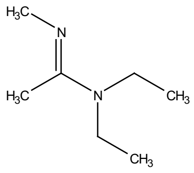

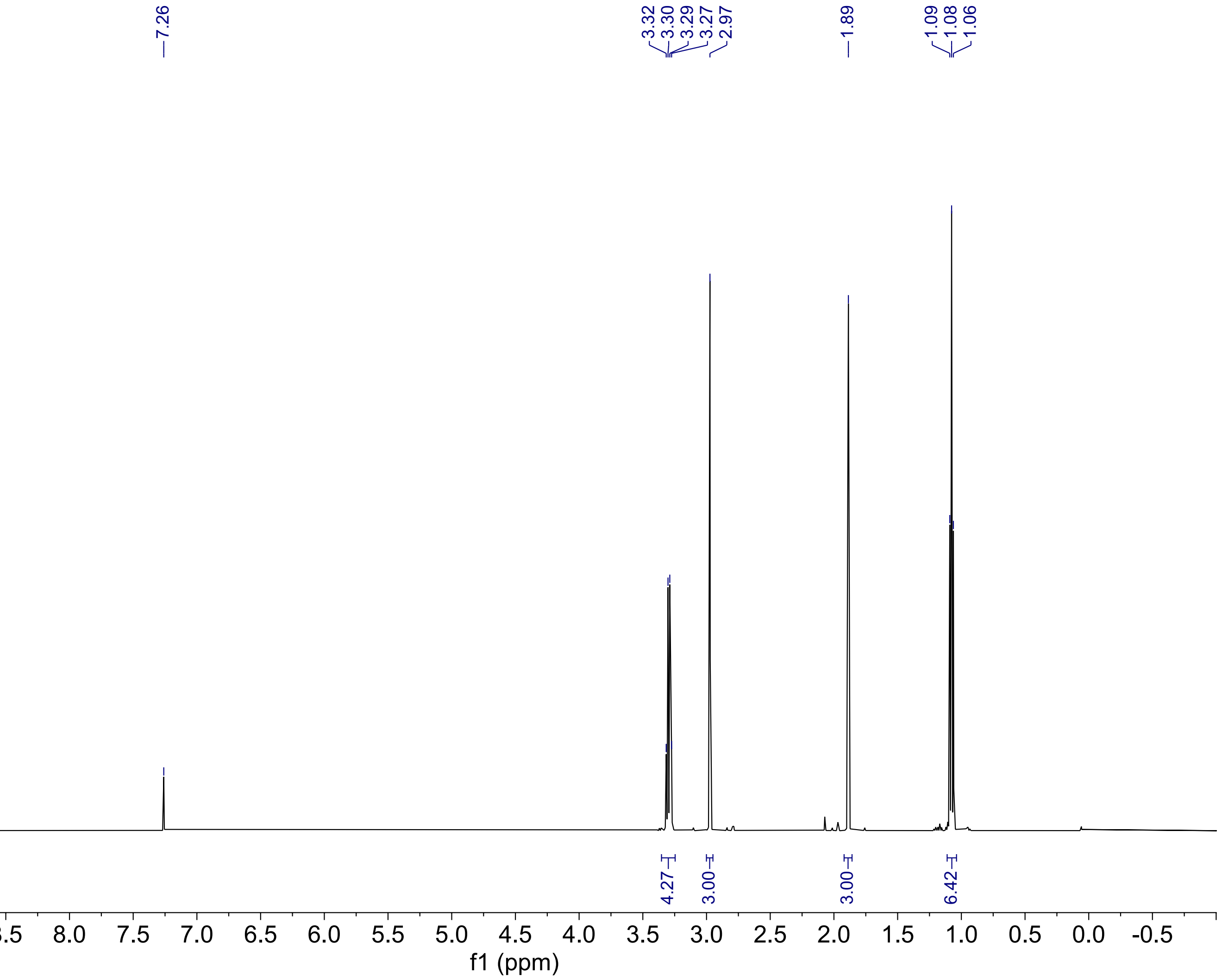

$^{13}C\{^{1}H\}$ NMR of (*E*)-*N*,*N*-diethyl-*N*'-methylacetimidamide (3a) ($CDCl_3$, 126 MHz)

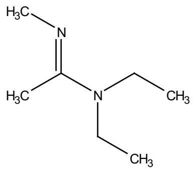

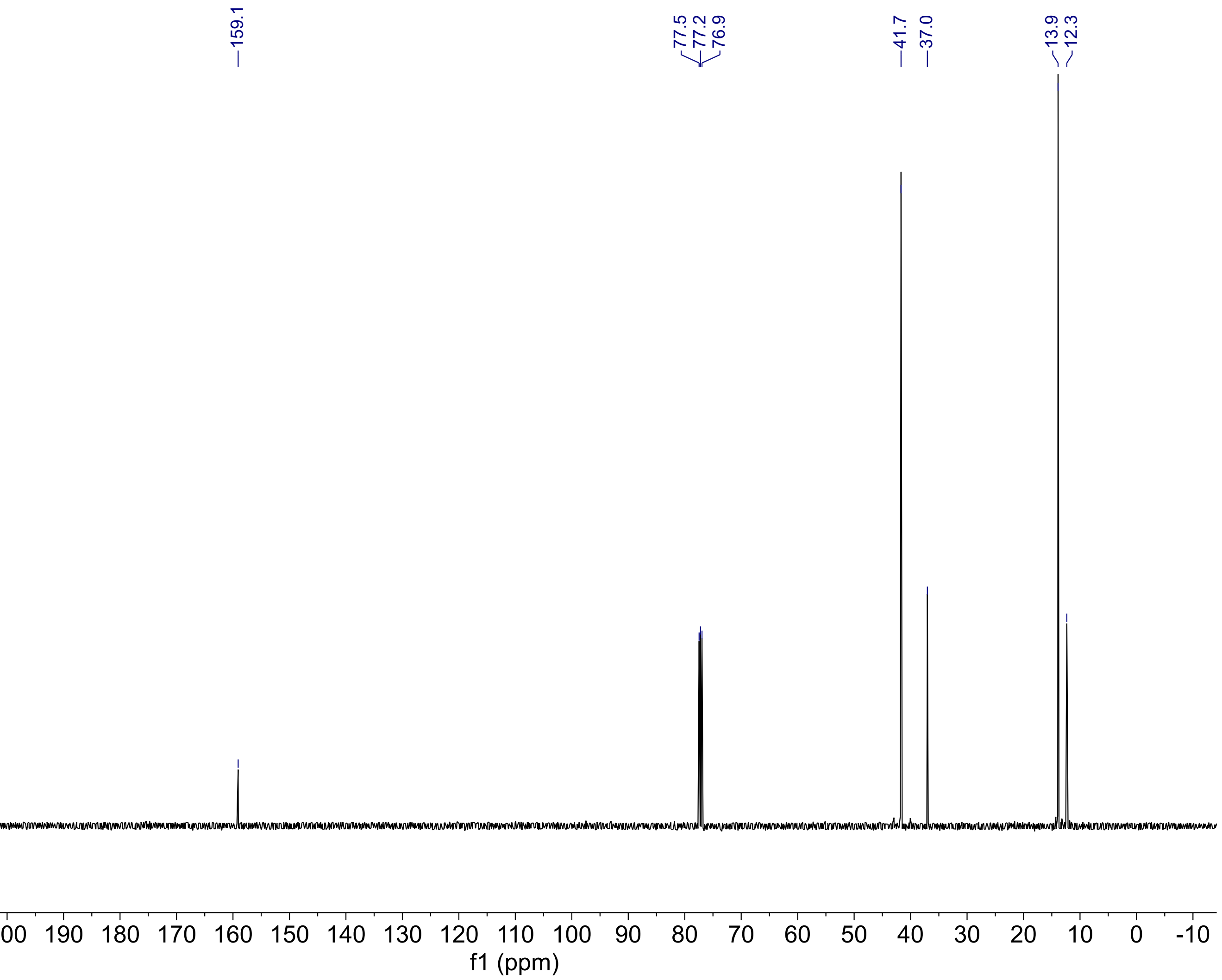

^{1}H NMR of (*E*)-*N*,*N*-diethyl-*N'*-methylpropionimidamide (3b) ($CDCl_3$, 500 MHz)

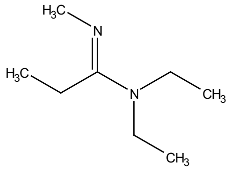

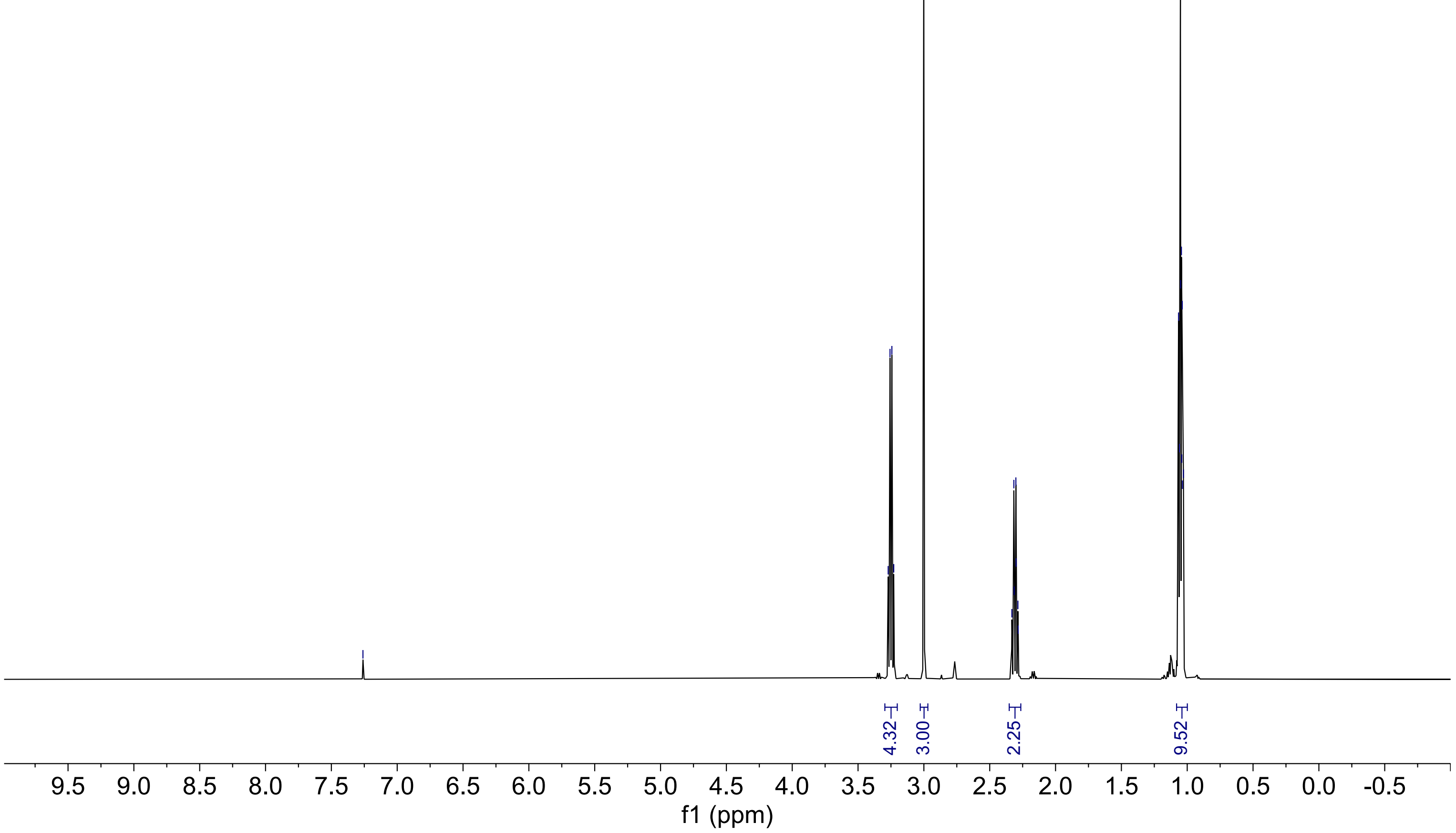

$^{13}C\{^1H\}$ NMR of (*E*)-*N*,*N*-diethyl-*N*'-methylpropionimidamide (3b) ($CDCl_3$, 126 MHz)

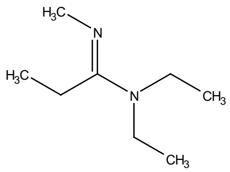

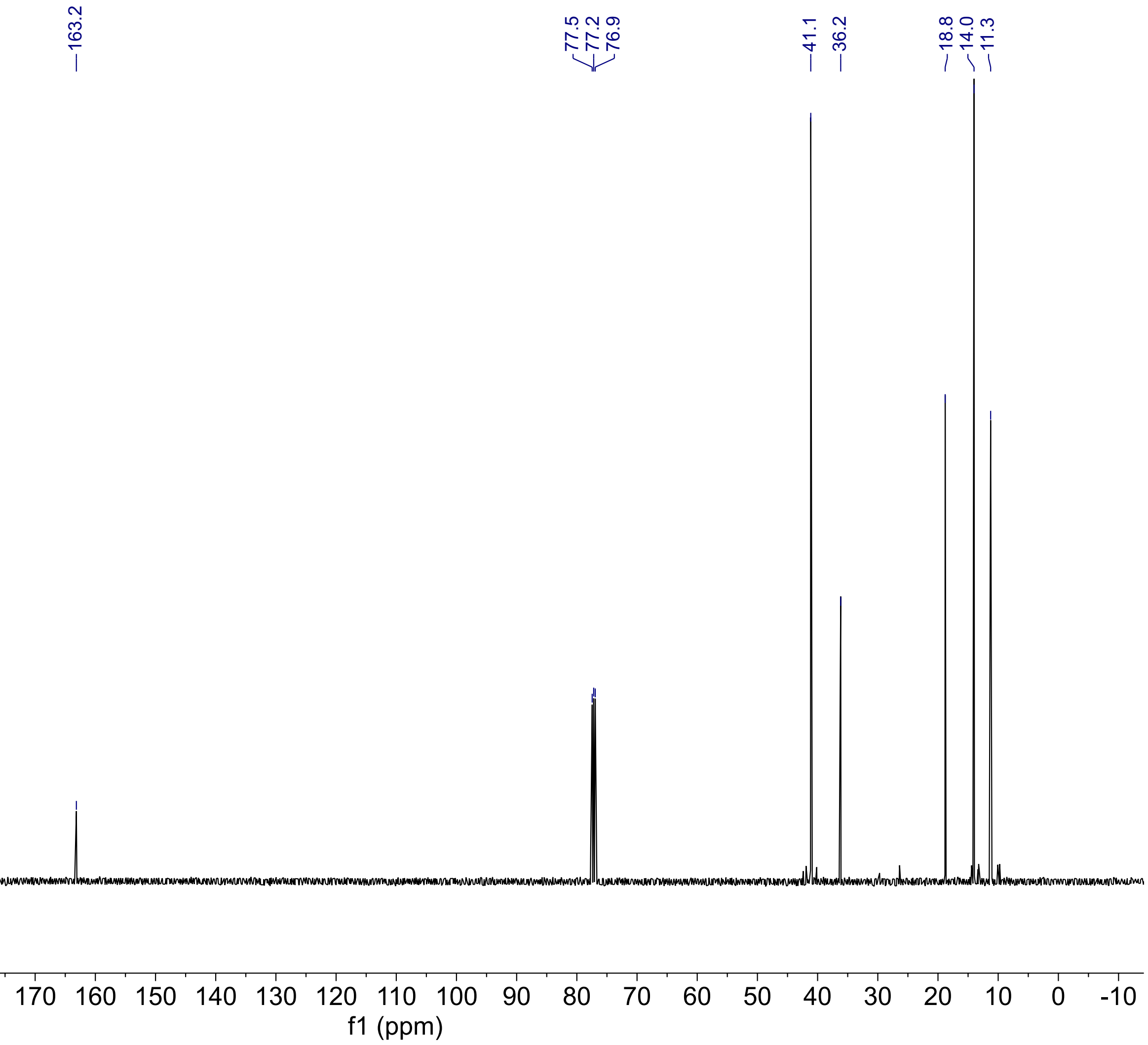

^{1}H NMR of (*E*)-*N*,*N*,*N*'-triethylacetimidamide (3c) ($CDCl_3$, 500 MHz)

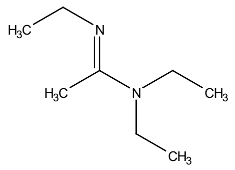

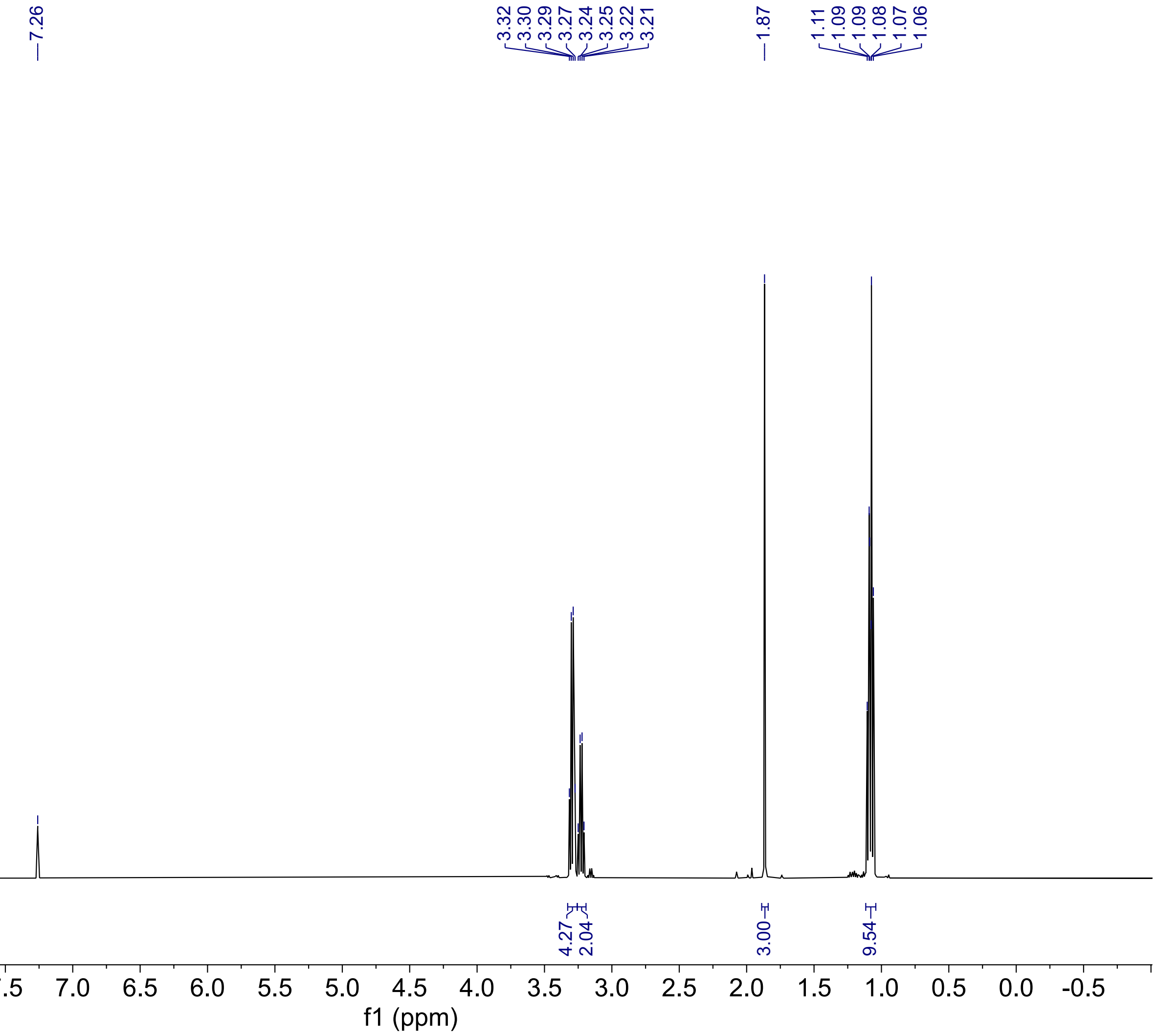

$^{13}C\{^1H\}$ NMR of (*E*)-*N*,*N*,*N'*-triethylacetimidamide (3c) ($CDCl_3$, 126 MHz)

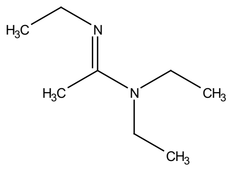

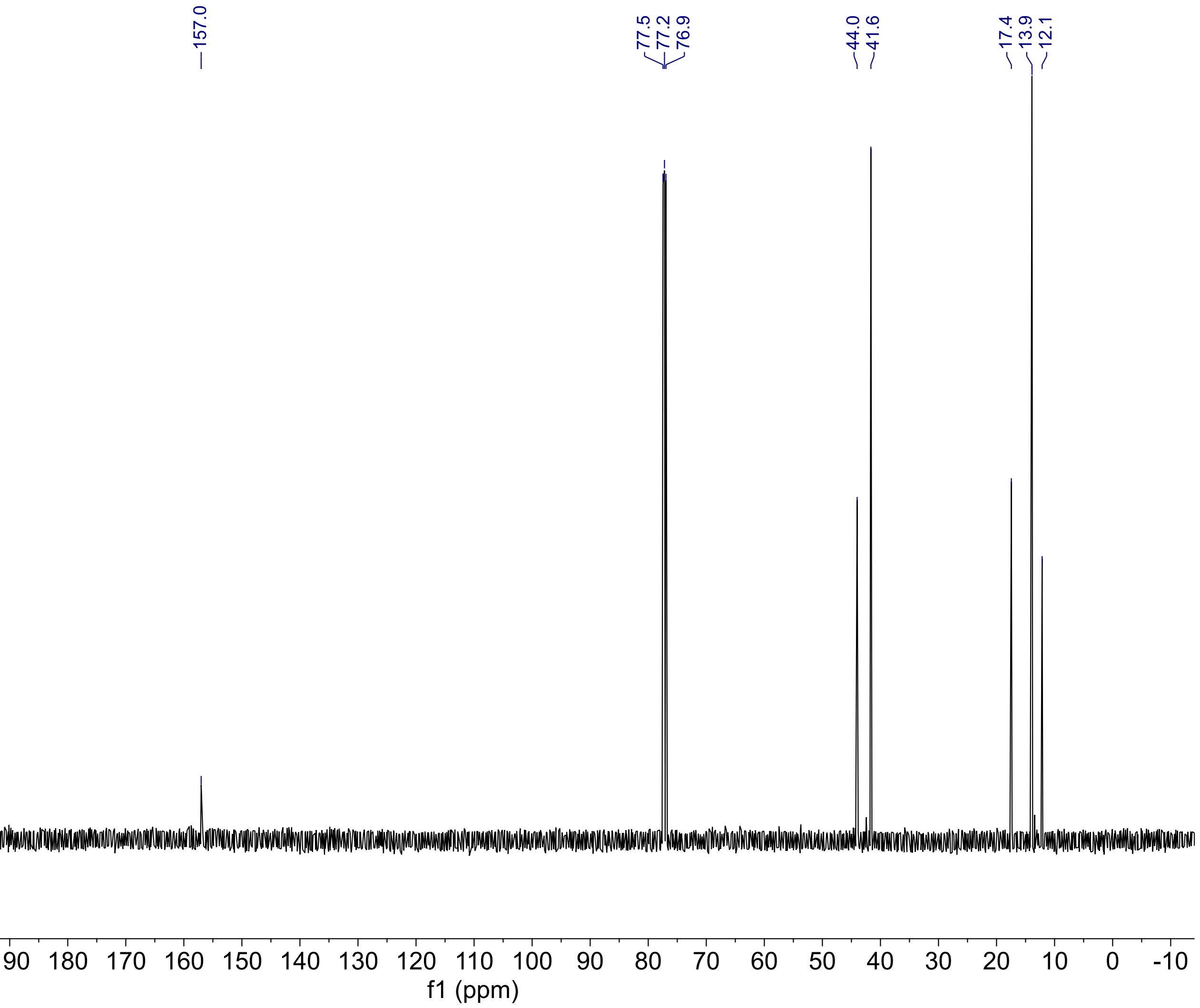

^{1}H NMR of (*E*)-*N*,*N*,*N*'-triethylpropionimidamide (3d) ($CDCl_3$, 500 MHz)

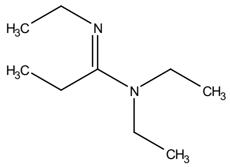

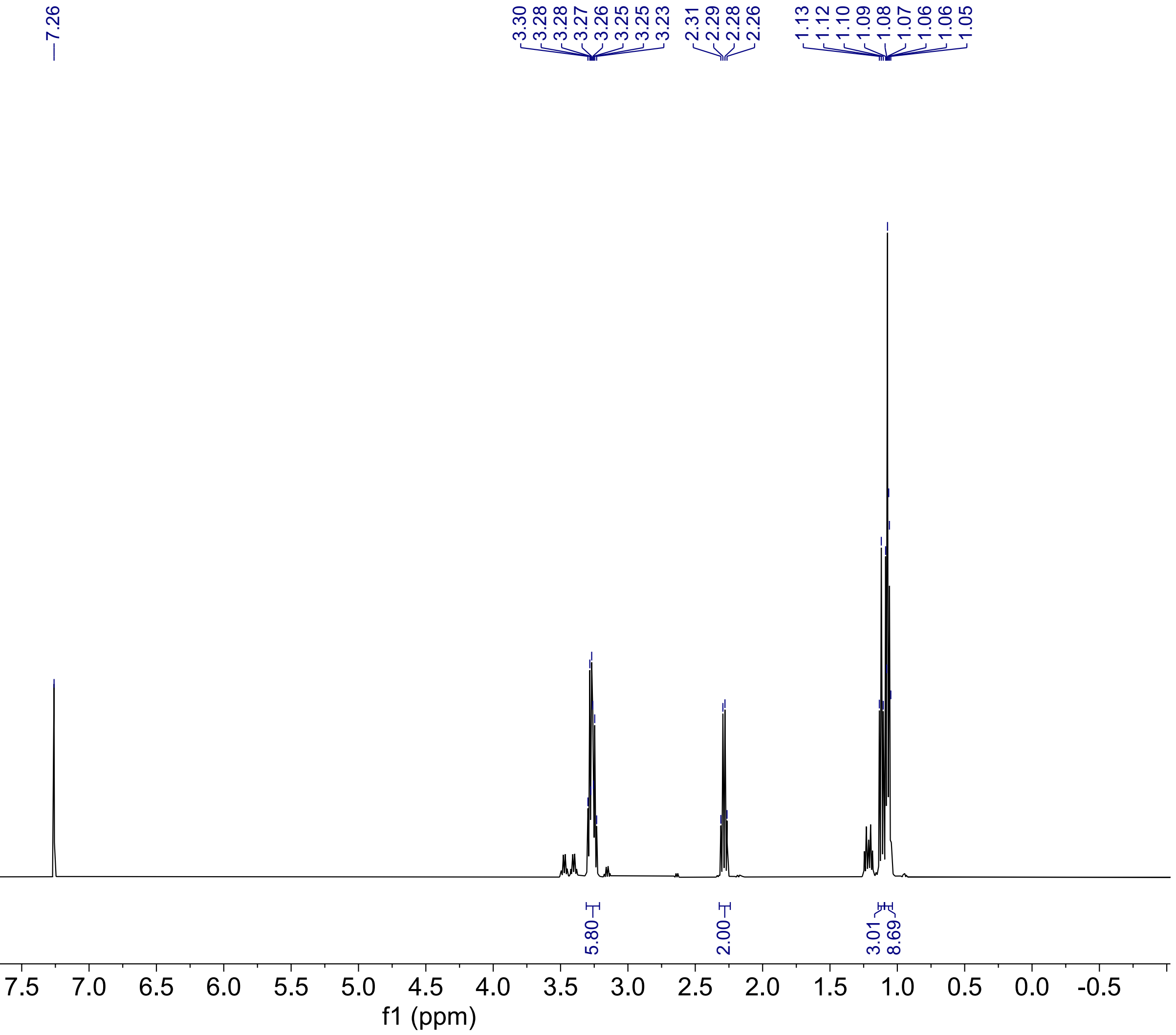

$^{13}C\{^1H\}$ NMR of (*E*)-*N*,*N*,*N'*-triethylpropionimidamide (3d) ($CDCl_3$, 126 MHz)

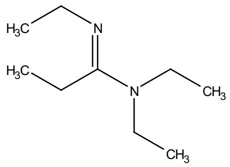

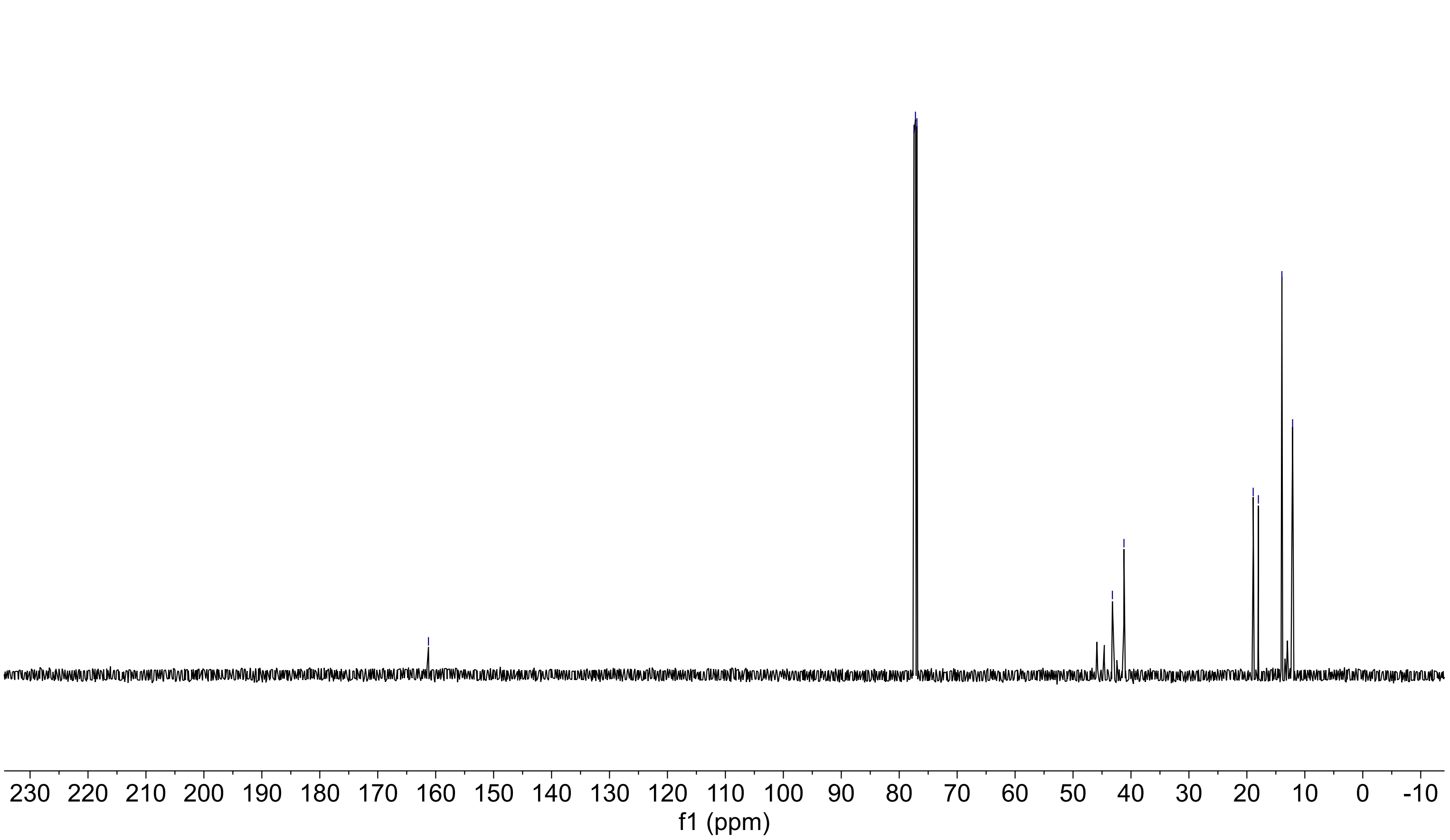

^{1}H NMR of (*E*)-*N*-ethyl-1-(piperidin-1-yl)ethan-1-imine (3e) ($CDCl_3$, 500 MHz)

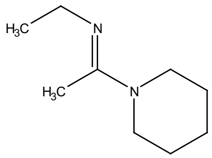

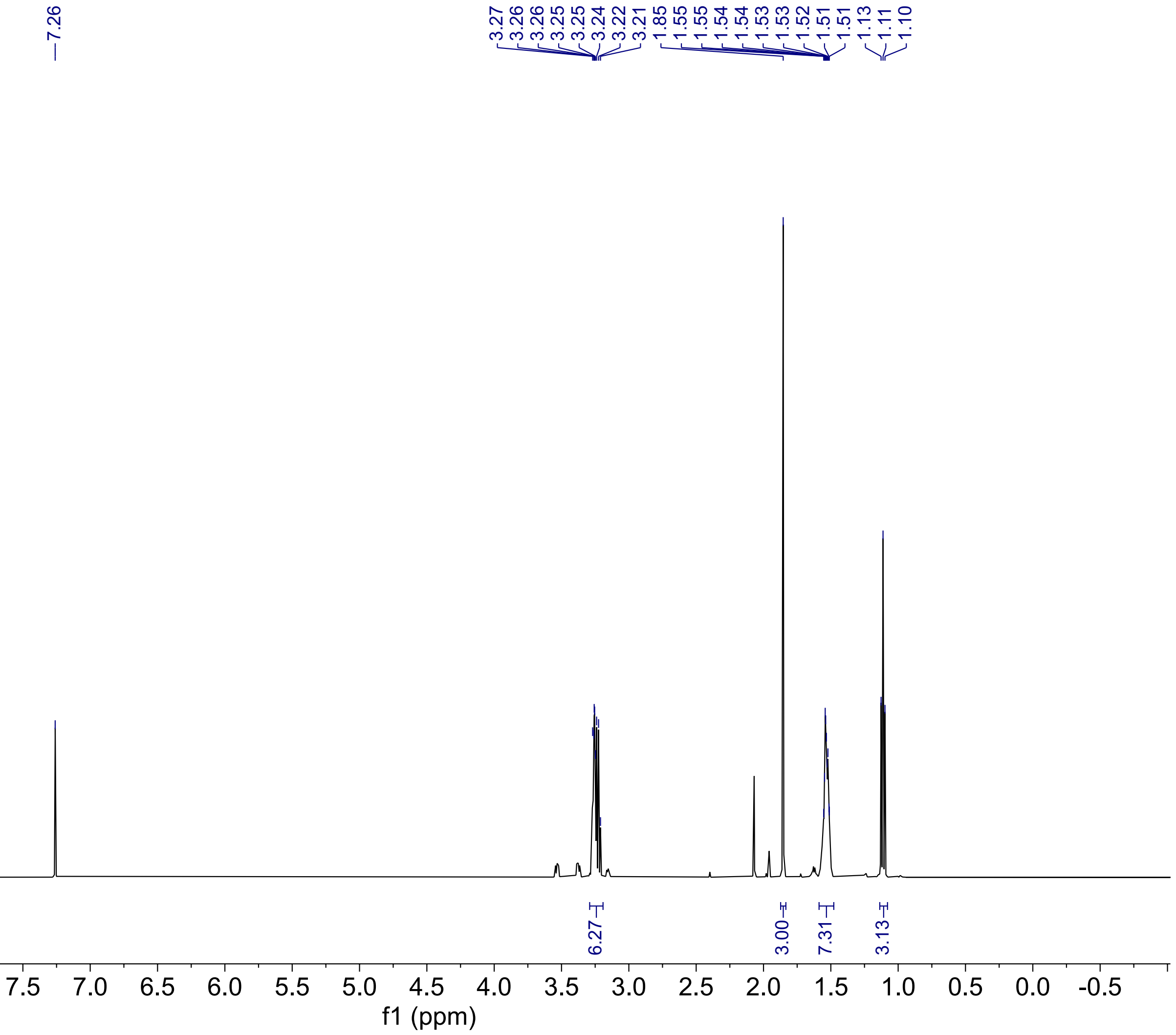

$^{13}C\{^{1}H\}$ NMR of (*E*)-*N*-ethyl-1-(piperidin-1-yl)ethan-1-imine (3e) ($CDCl_3$, 126 MHz)

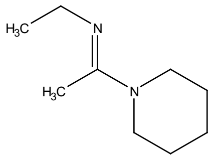

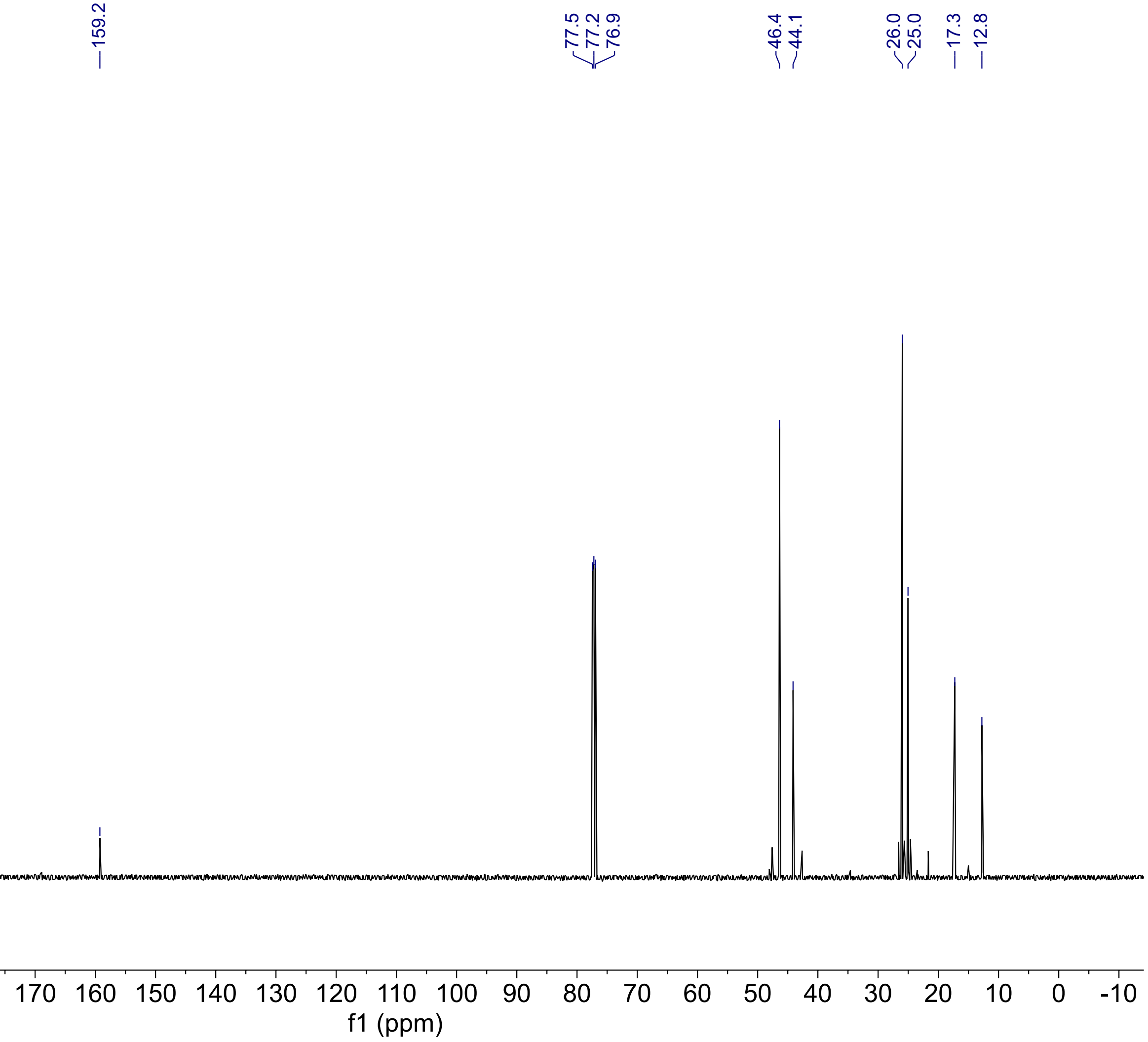

^{1}H NMR of *N*,*N*-diethyl-3,4-dihydro-2*H*-pyrrol-5-amine (3f) ($CDCl_3$, 500 MHz)

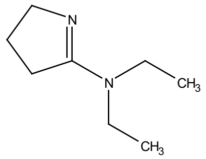

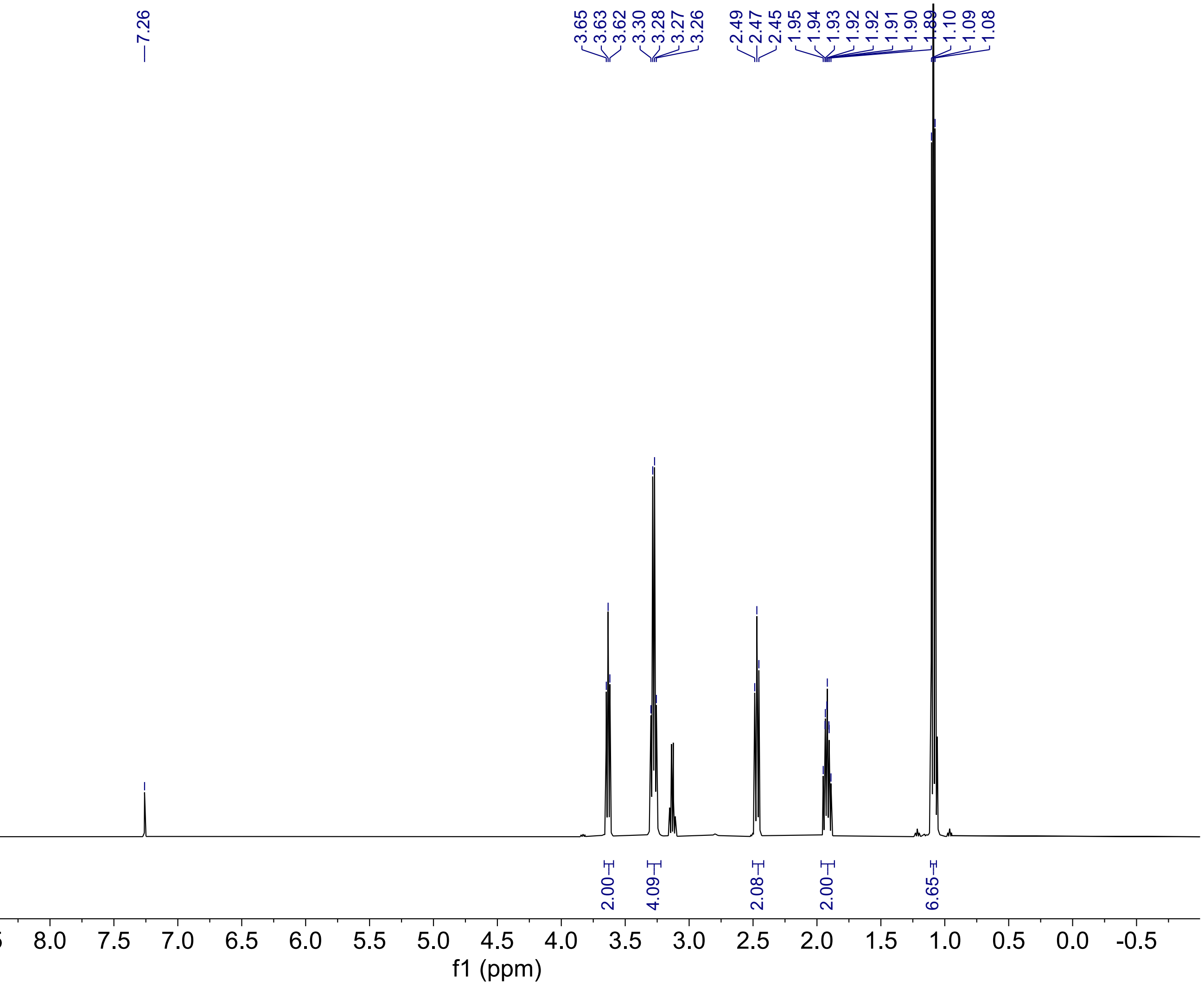

$^{13}C\{^1H\}$ NMR of *N*,*N*-diethyl-3,4-dihydro-2*H*-pyrrol-5-amine (3f) ($CDCl_3$, 126 MHz)

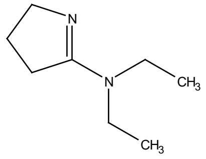

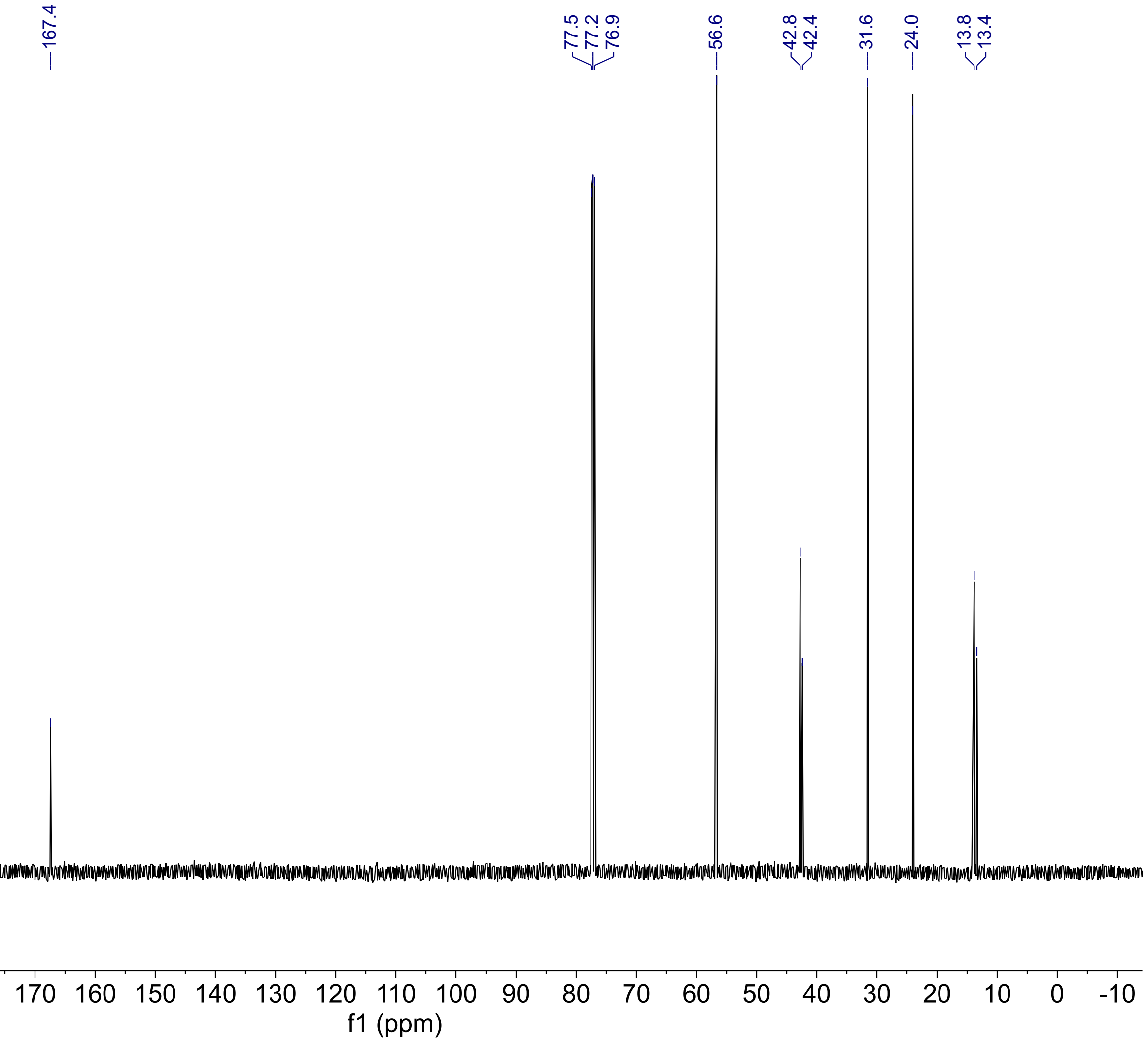

^{1}H NMR of *N*,*N*-diethyl-3,4,5,6-tetrahydropyridin-2-amine (3g) ($CDCl_3$, 500 MHz)

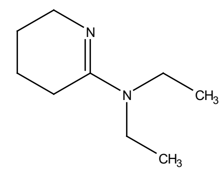

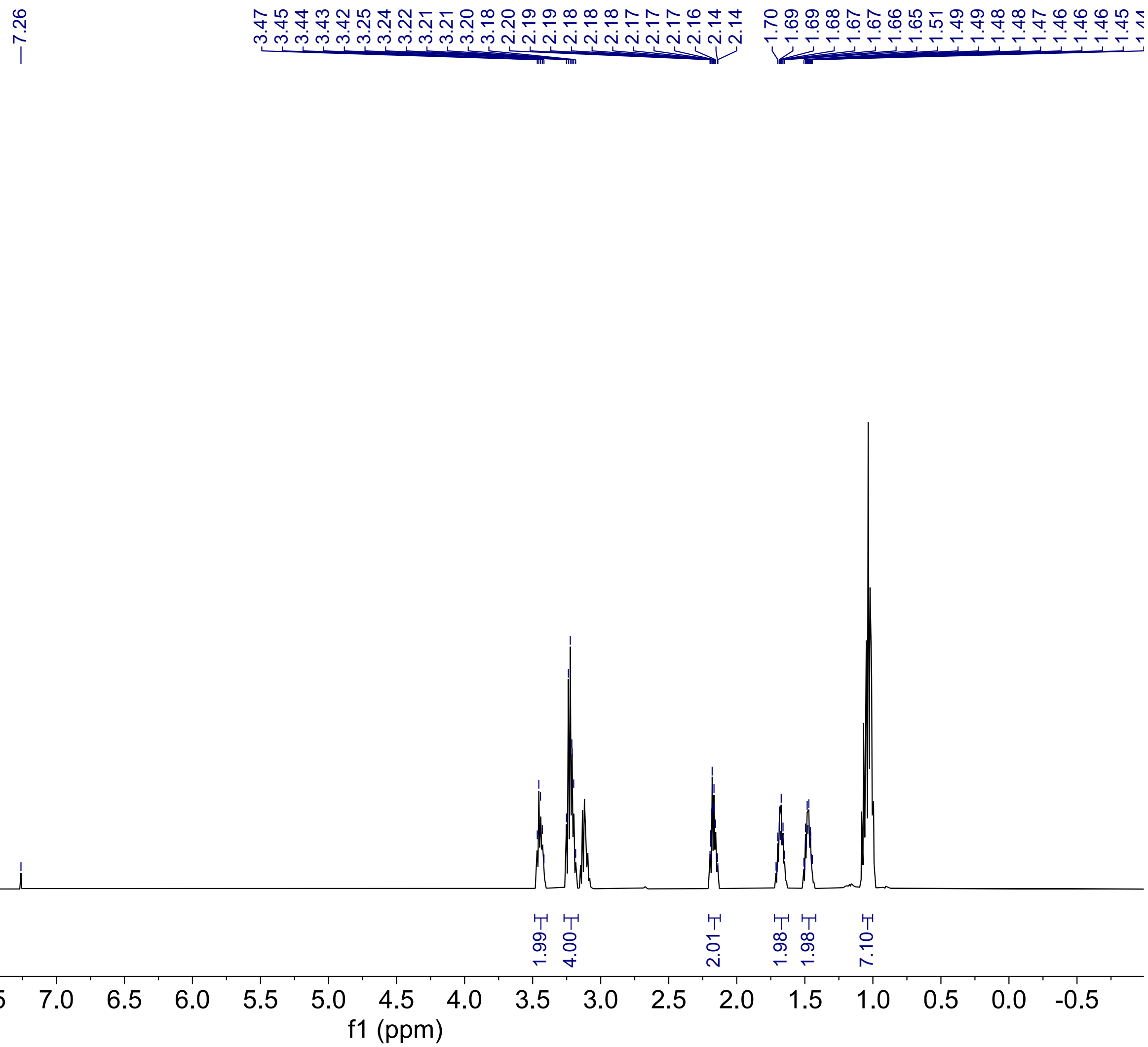

$^{13}C\{^{1}H\}$ NMR of *N*,*N*-diethyl-3,4,5,6-tetrahydropyridin-2-amine (3g) ($CDCl_3$, 126 MHz)

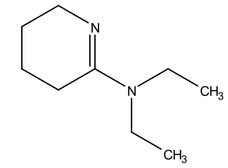

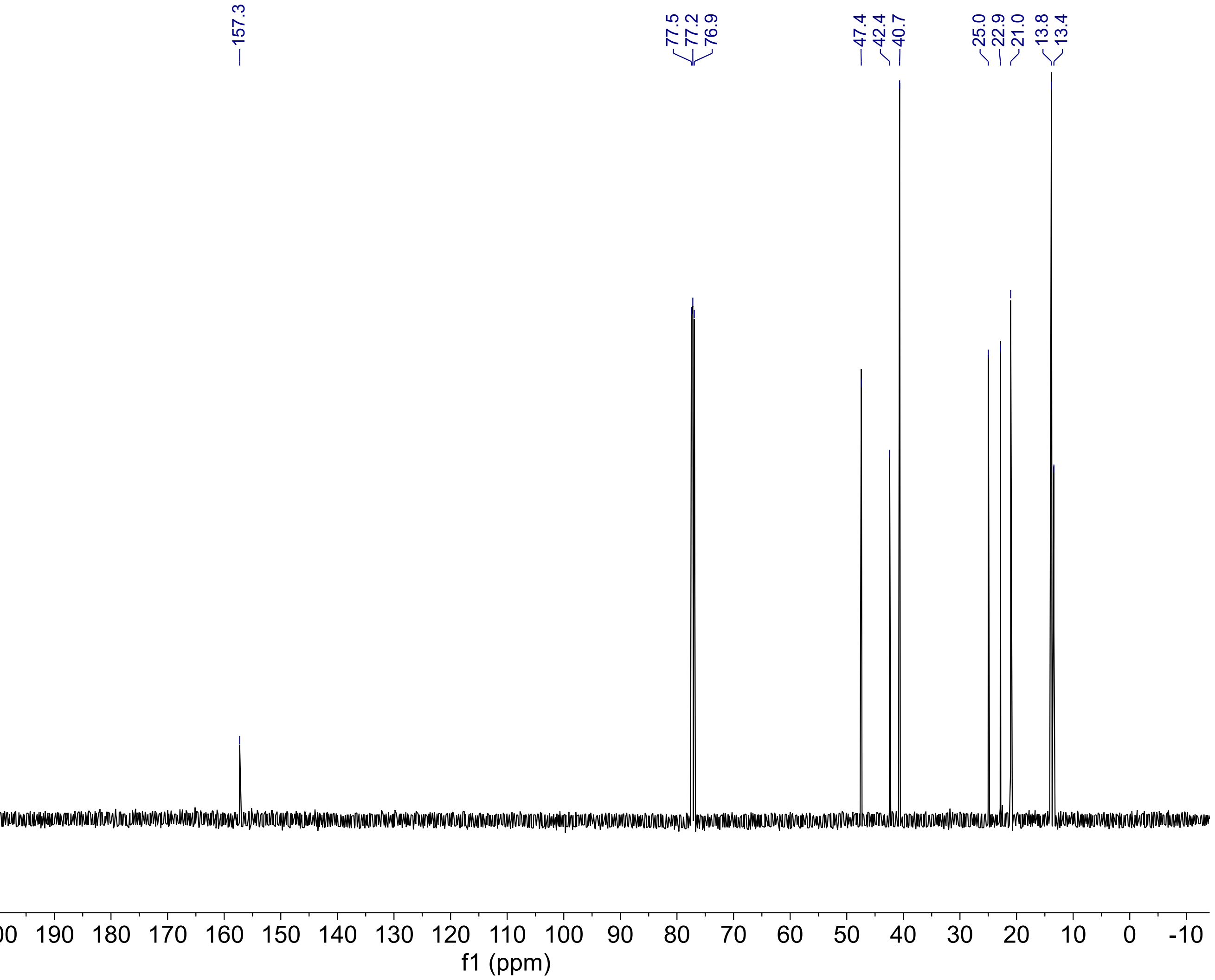

^{1}H NMR of *N,N-diethyl-3,4,5,6-tetrahydro-2H-azepin-7-amine* (3h) ($CDCl_3$, 500 MHz)

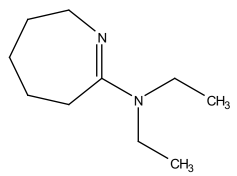

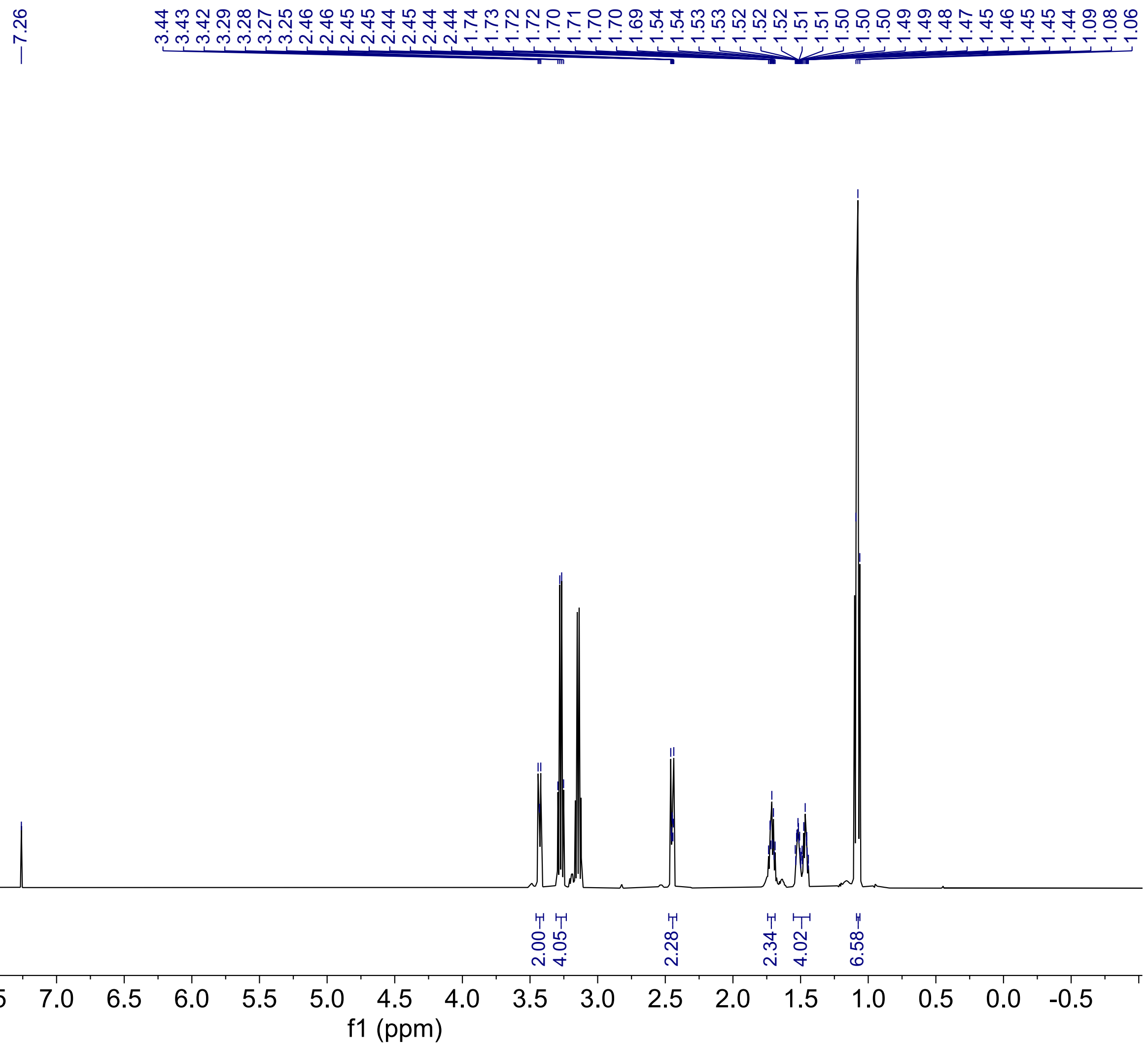

$^{13}C\{^1H\}$ NMR of *N,N-diethyl-3,4,5,6-tetrahydro-2H-azepin-7-amine* (3h) ($CDCl_3$, 126 MHz)

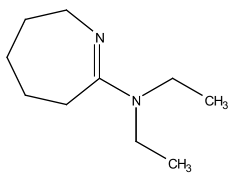

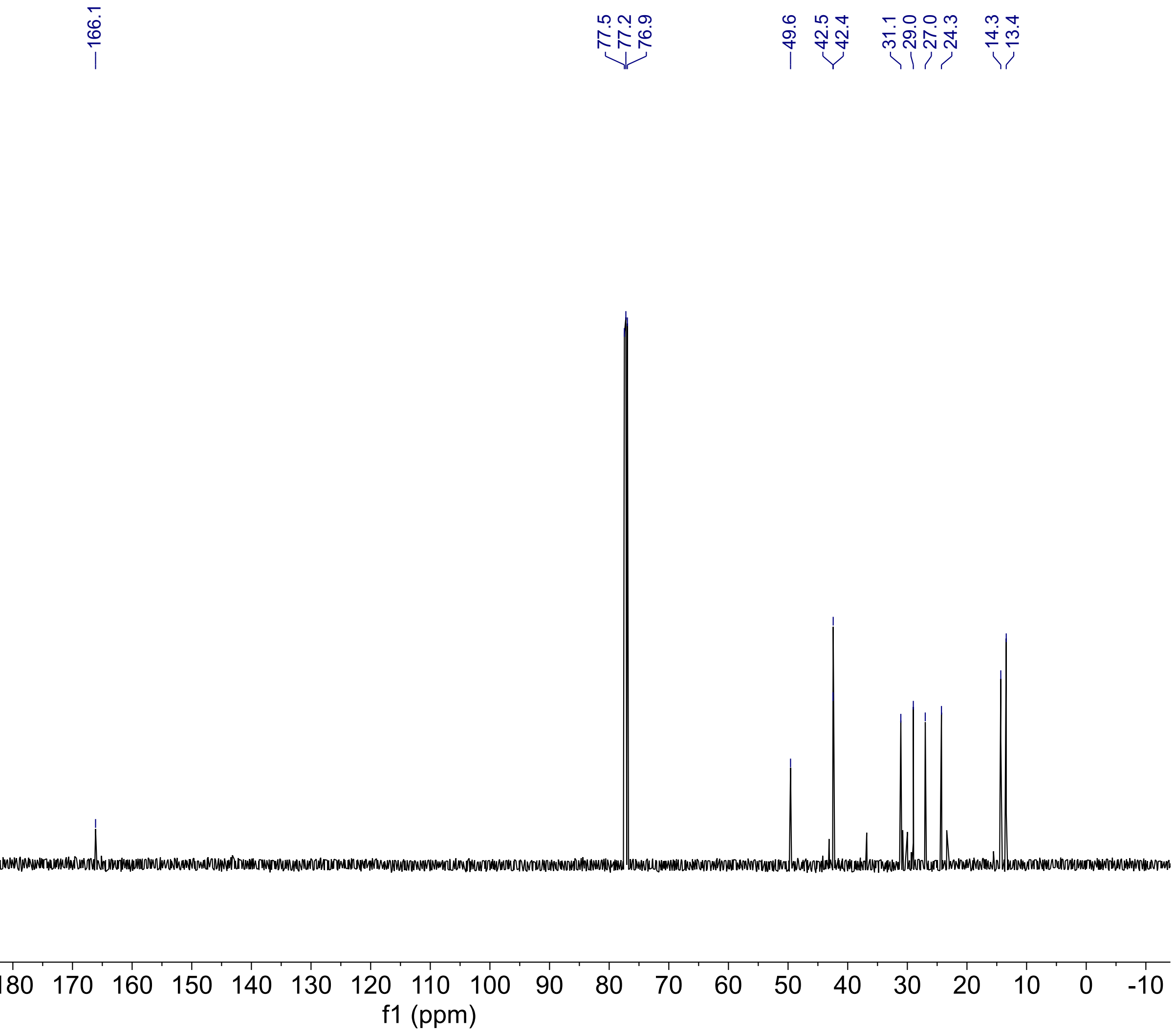

^{1}H NMR of B_2eg_2 (2,2'-Bi(1,3,2-dioxaborolane)) ($CDCl_3$, 500 MHz)

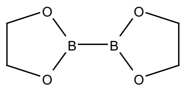

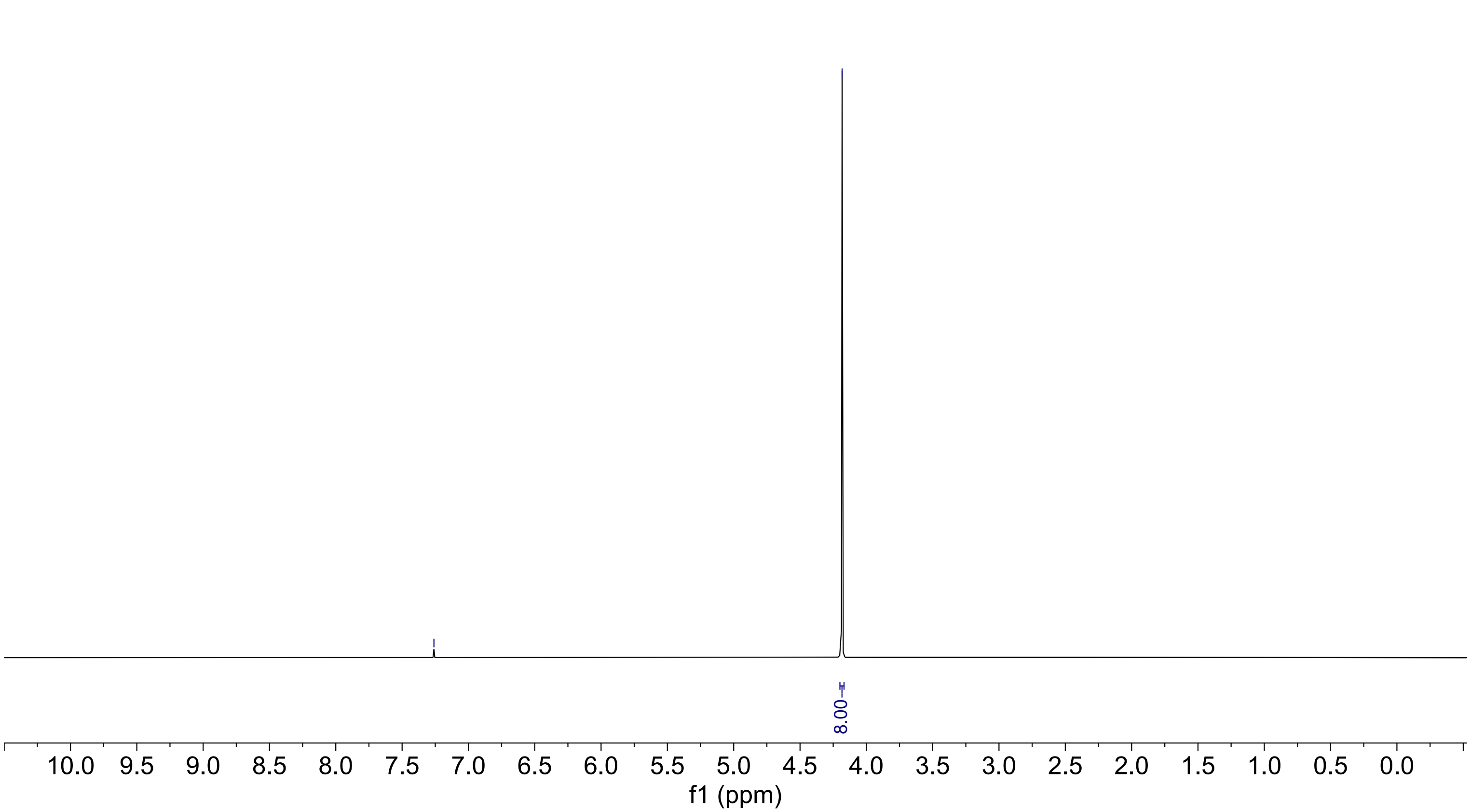

$^{13}C\{^1H\}$ NMR of B_2eg_2 (2,2'-Bi(1,3,2-dioxaborolane)) ($CDCl_3$, 126 MHz)

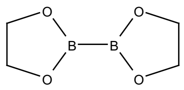

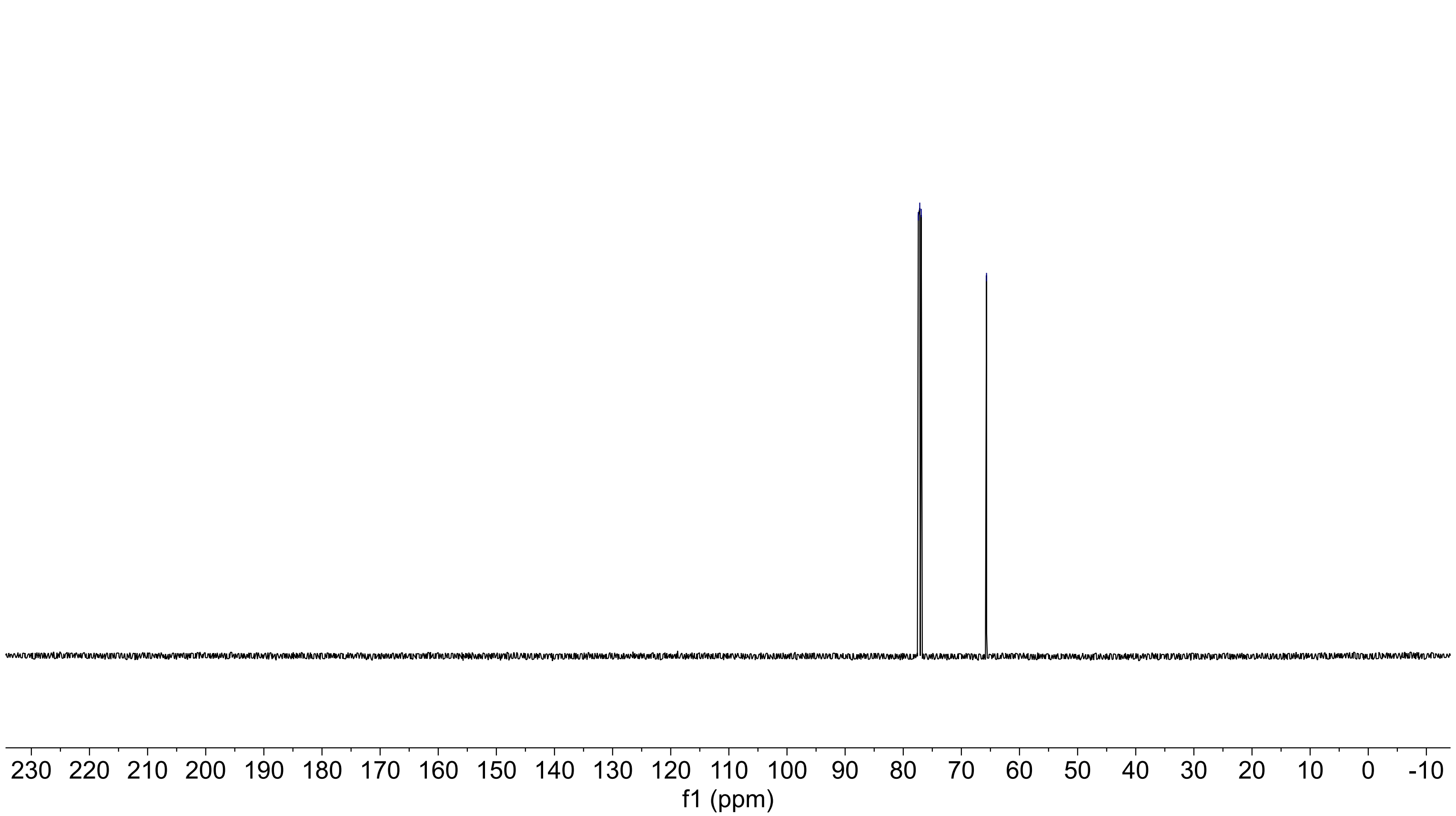

^{11}B NMR of B_2eg_2 (2,2'-Bi(1,3,2-dioxaborolane)) ($CDCl_3$, 160 MHz)

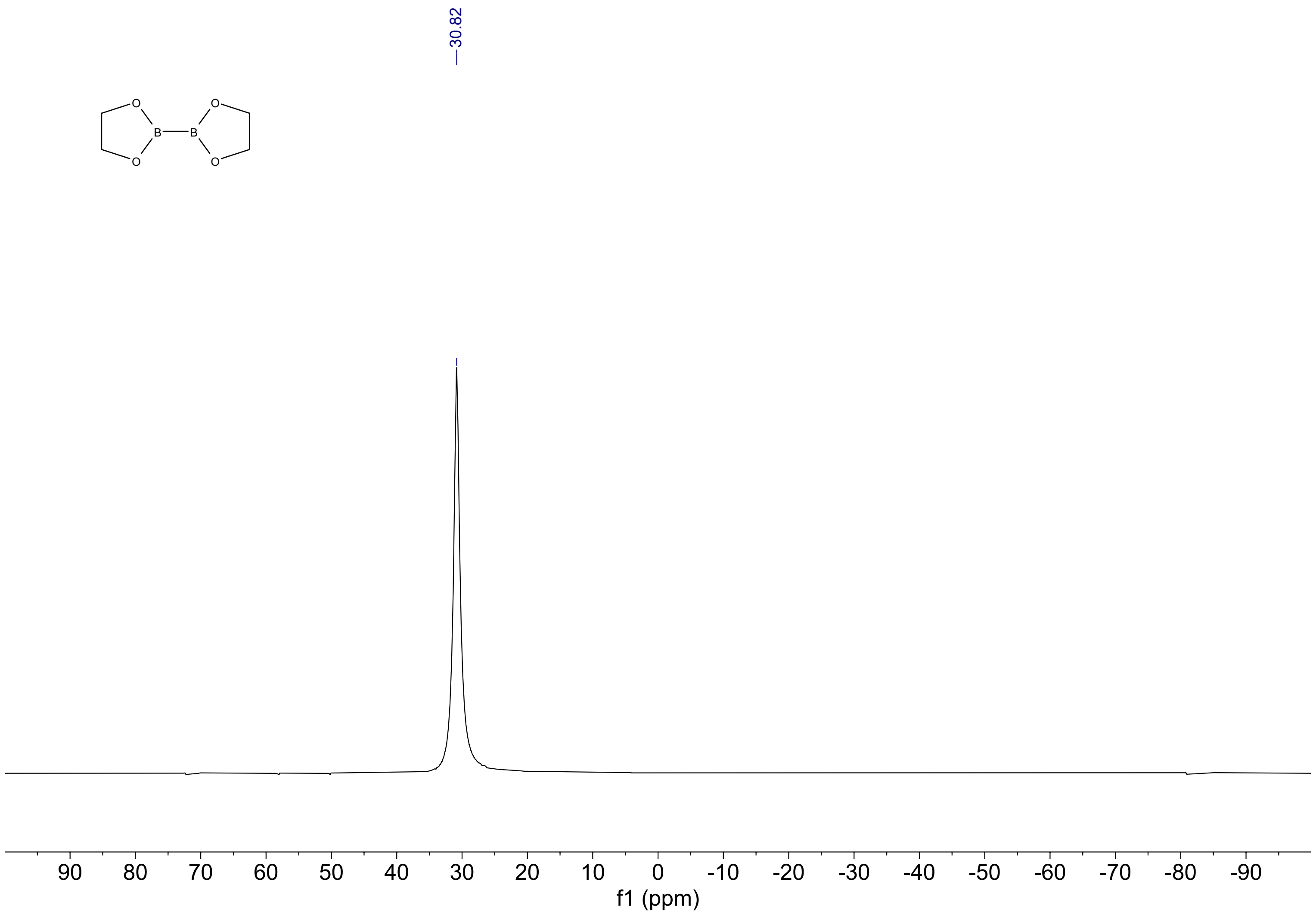

^{1}H NMR of B_2pg_2 (4,4'-Dimethyl-2,2'-bi(1,3,2-dioxaborolane)) ($CDCl_3$, 500 MHz)

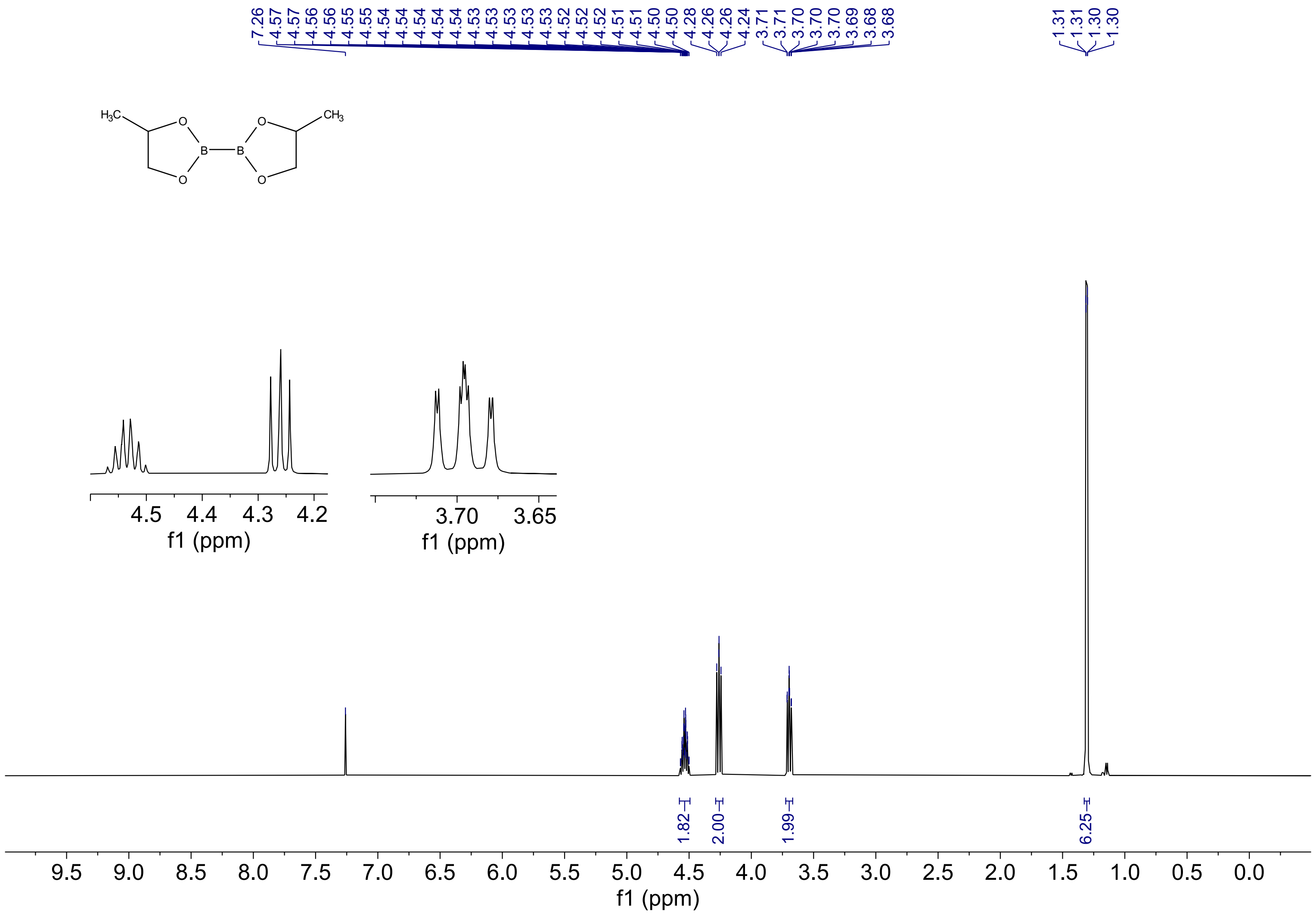

$^{13}C\{^1H\}$ NMR of B_2pg_2 (4,4'-Dimethyl-2,2'-bi(1,3,2-dioxaborolane)) ($CDCl_3$, 126 MHz)

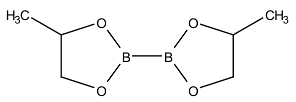

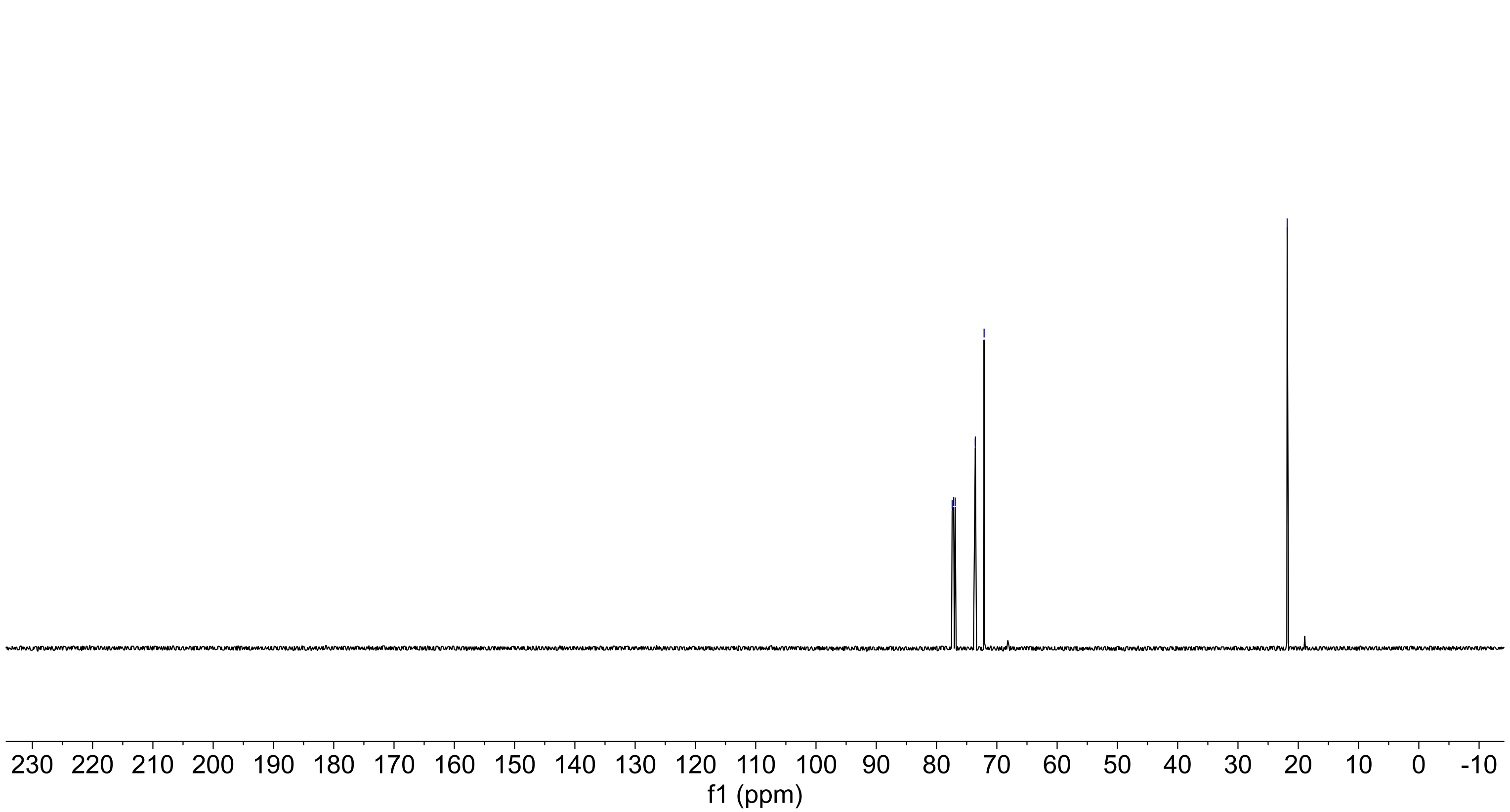

^{11}B NMR of B_2pg_2 (4,4'-Dimethyl-2,2'-bi(1,3,2-dioxaborolane)) ($CDCl_3$, 160 MHz)

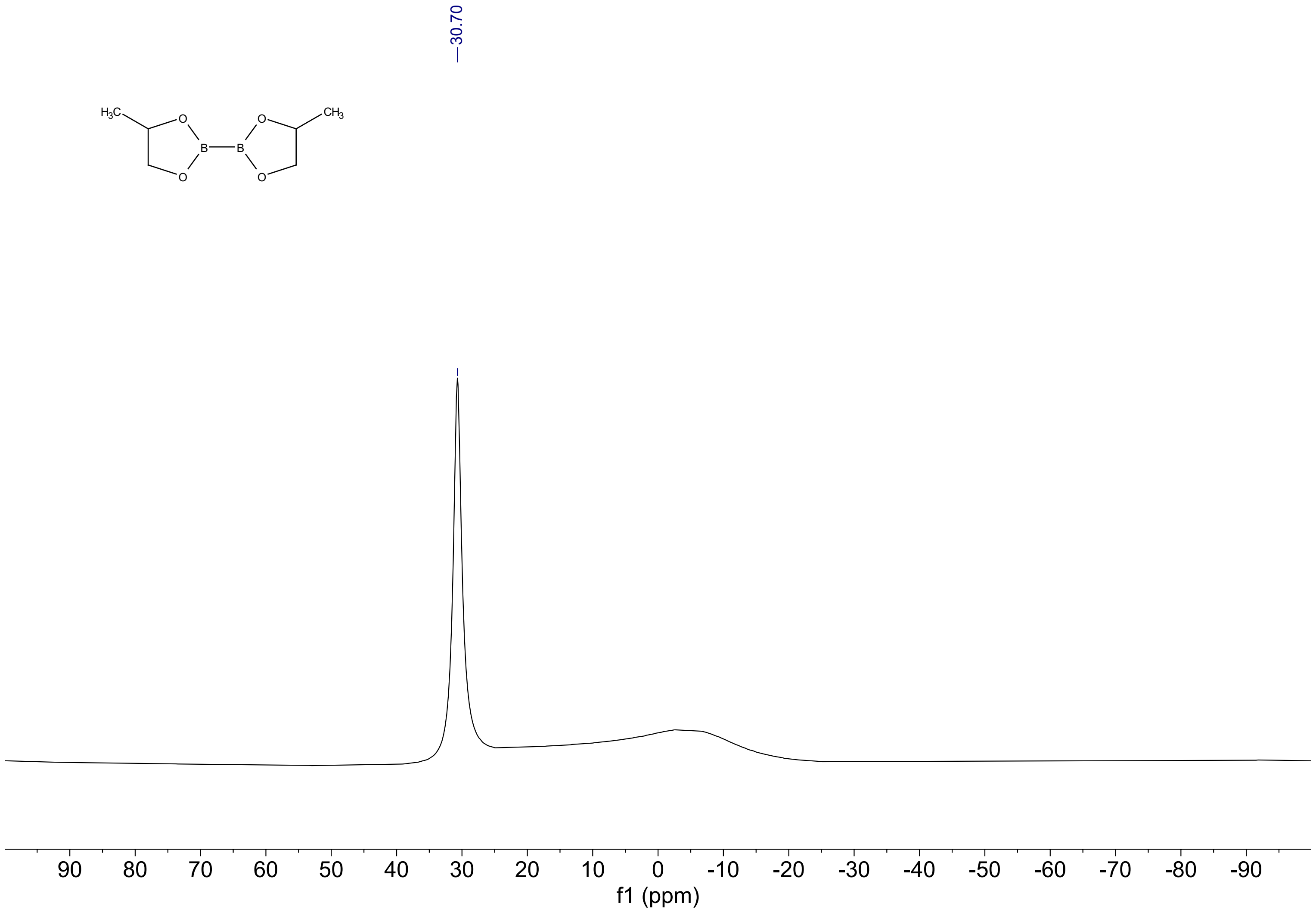

^{1}H NMR of B_2bg_2 (4,4'-Diethyl-2,2'-bi(1,3,2-dioxaborolane)) (CDCl3, 500 MHz)

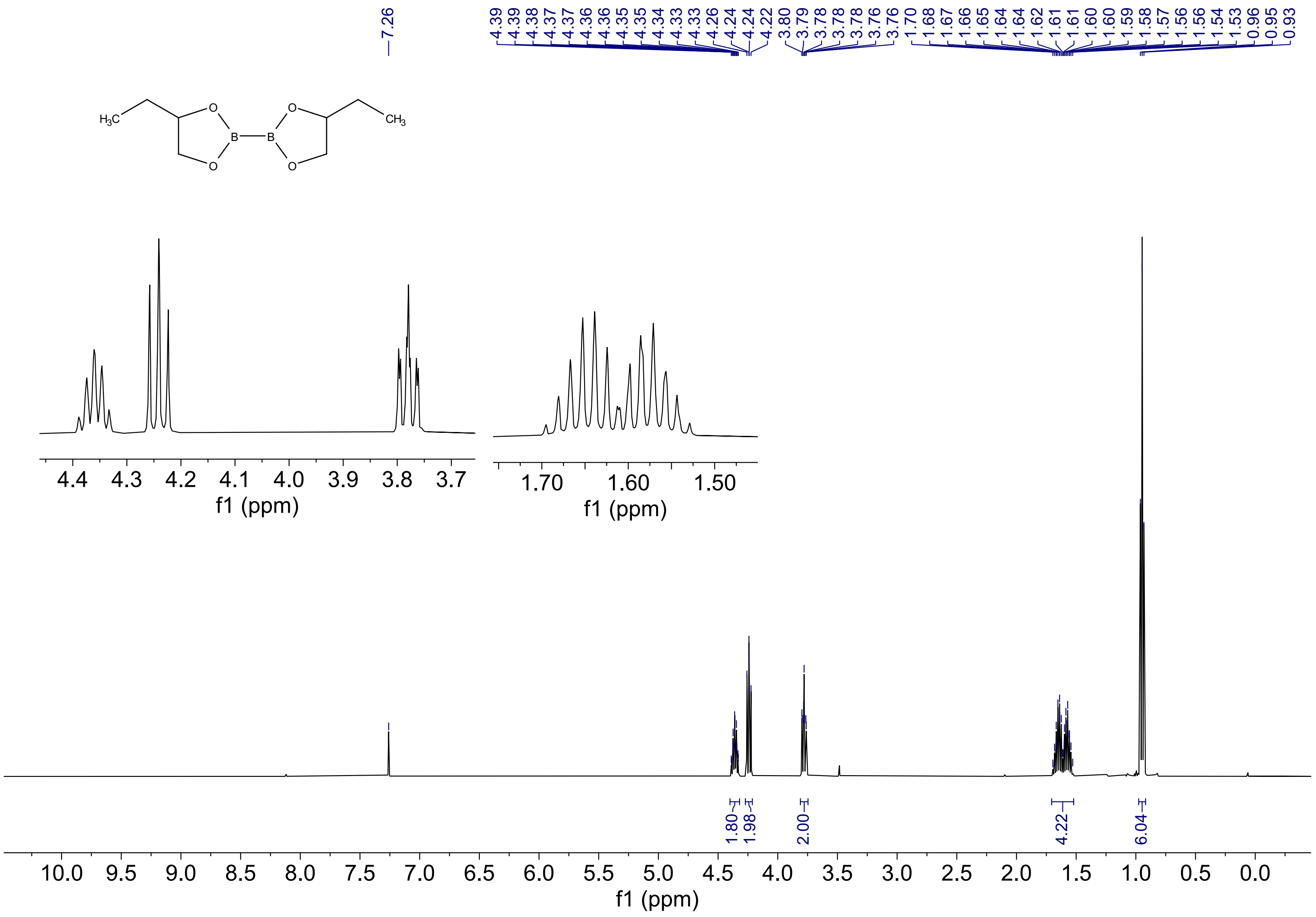

$^{13}C\{^1H\}$ NMR of B_2bg_2 (4,4'-Diethyl-2,2'-bi(1,3,2-dioxaborolane)) ($CDCl_3$, 126 MHz)

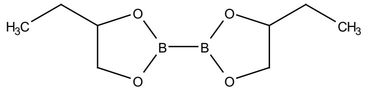

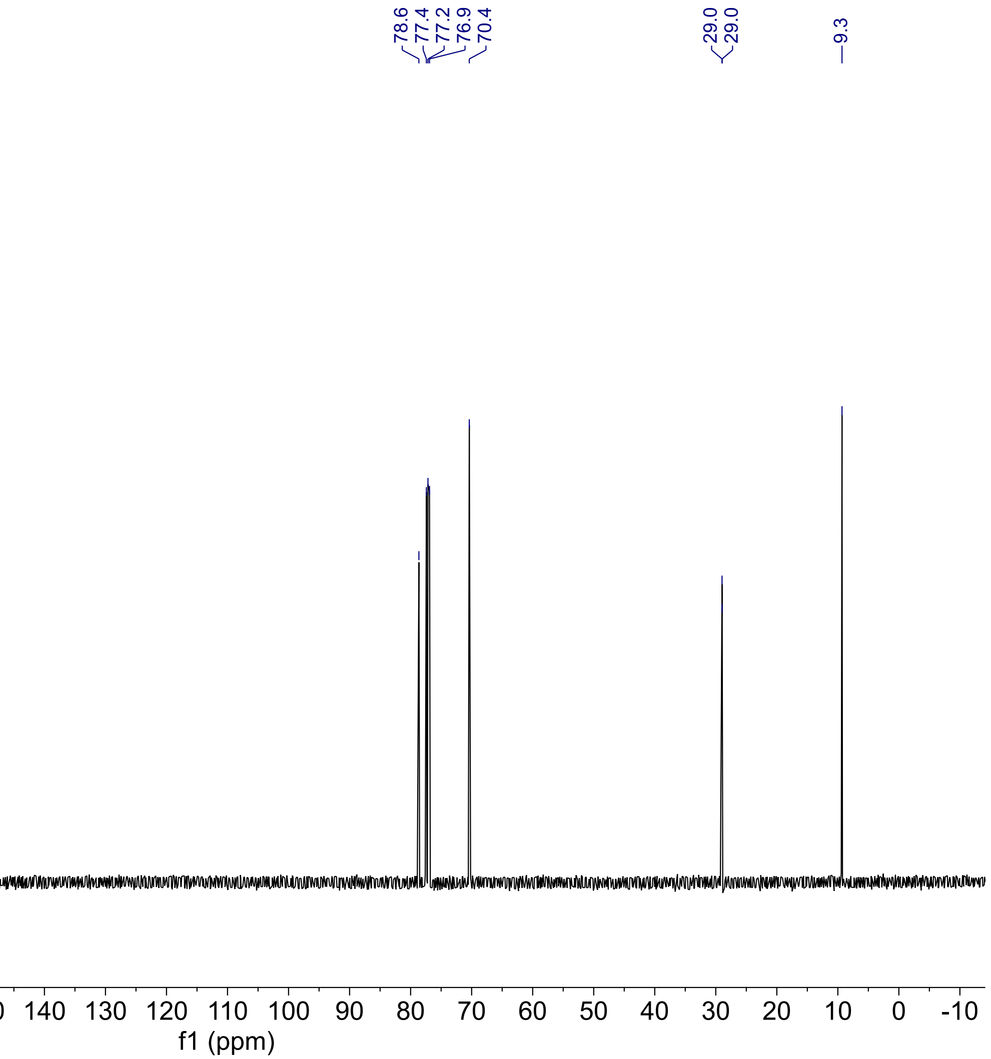

^{11}B NMR of B_2bg_2 (4,4'-Diethyl-2,2'-bi(1,3,2-dioxaborolane)) ($CDCl_3$, 160 MHz)

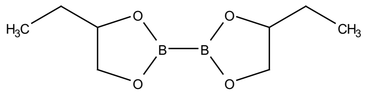

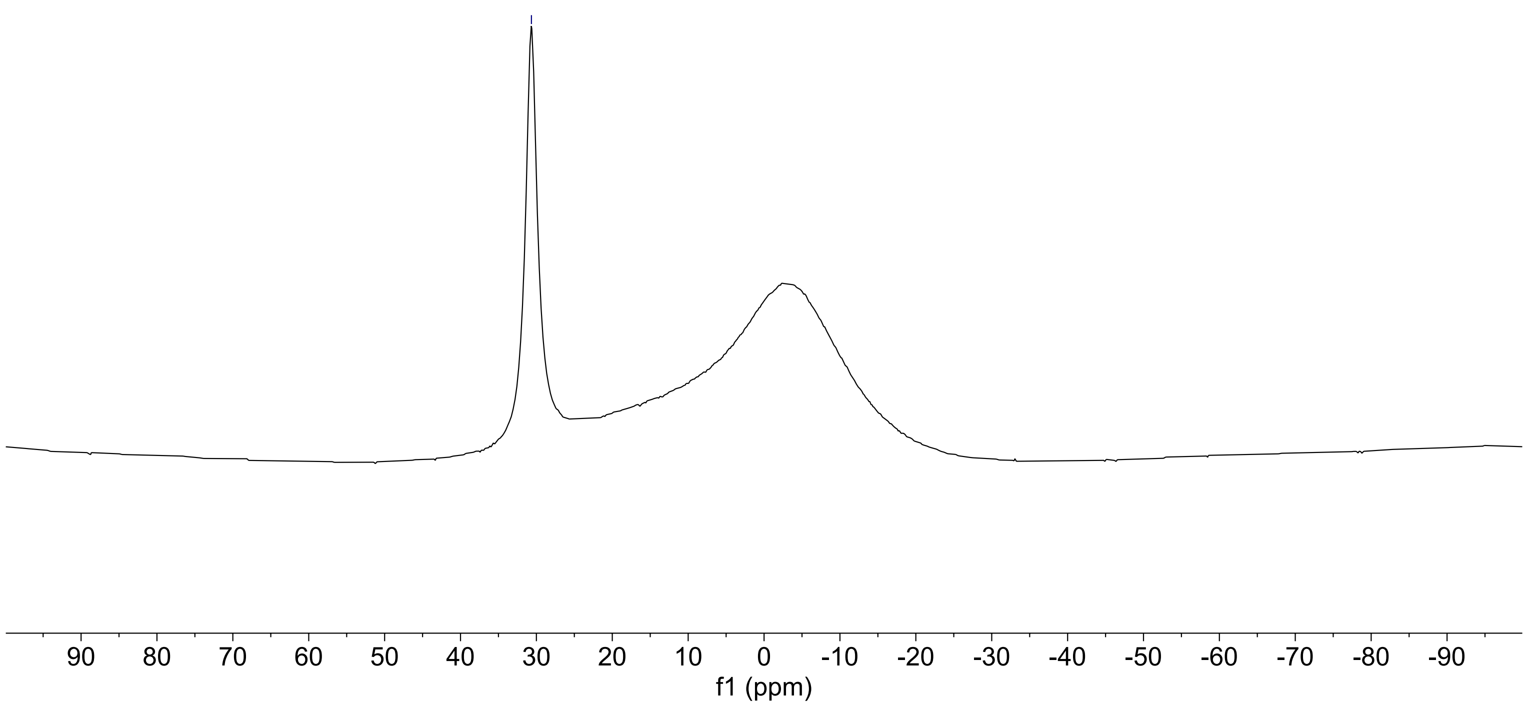

^{1}H NMR of B_2mbg_2 (4,4,4',4'-tetramethyl-2,2'-bi(1,3,2)dioxaborinane) ($CDCl_3$, 500 MHz)

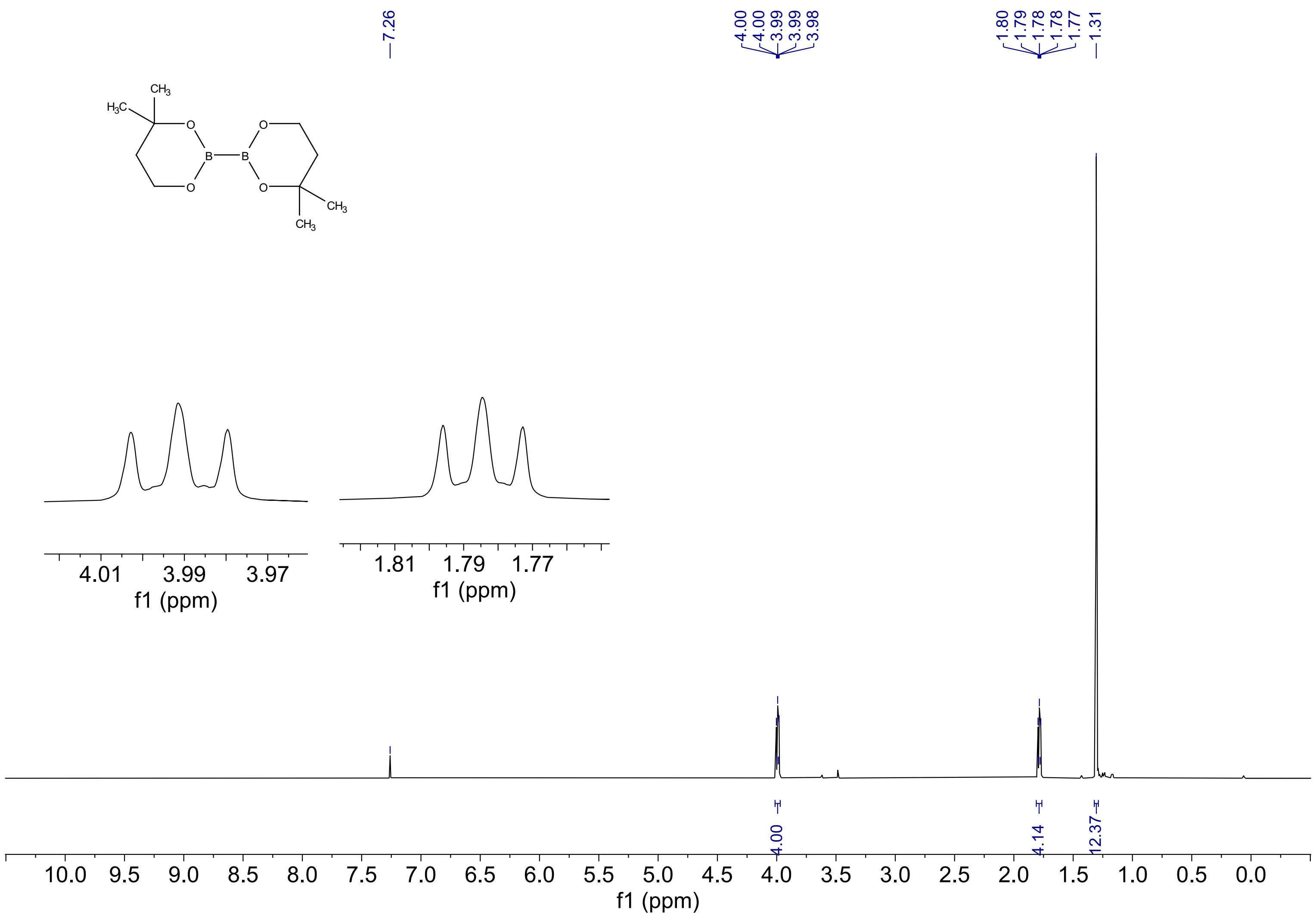

^{13}C NMR of B_2mbg_2 (4,4,4',4'-tetramethyl-2,2'-bi(1,3,2)dioxaborinane) ($CDCl_3$, 126 MHz)

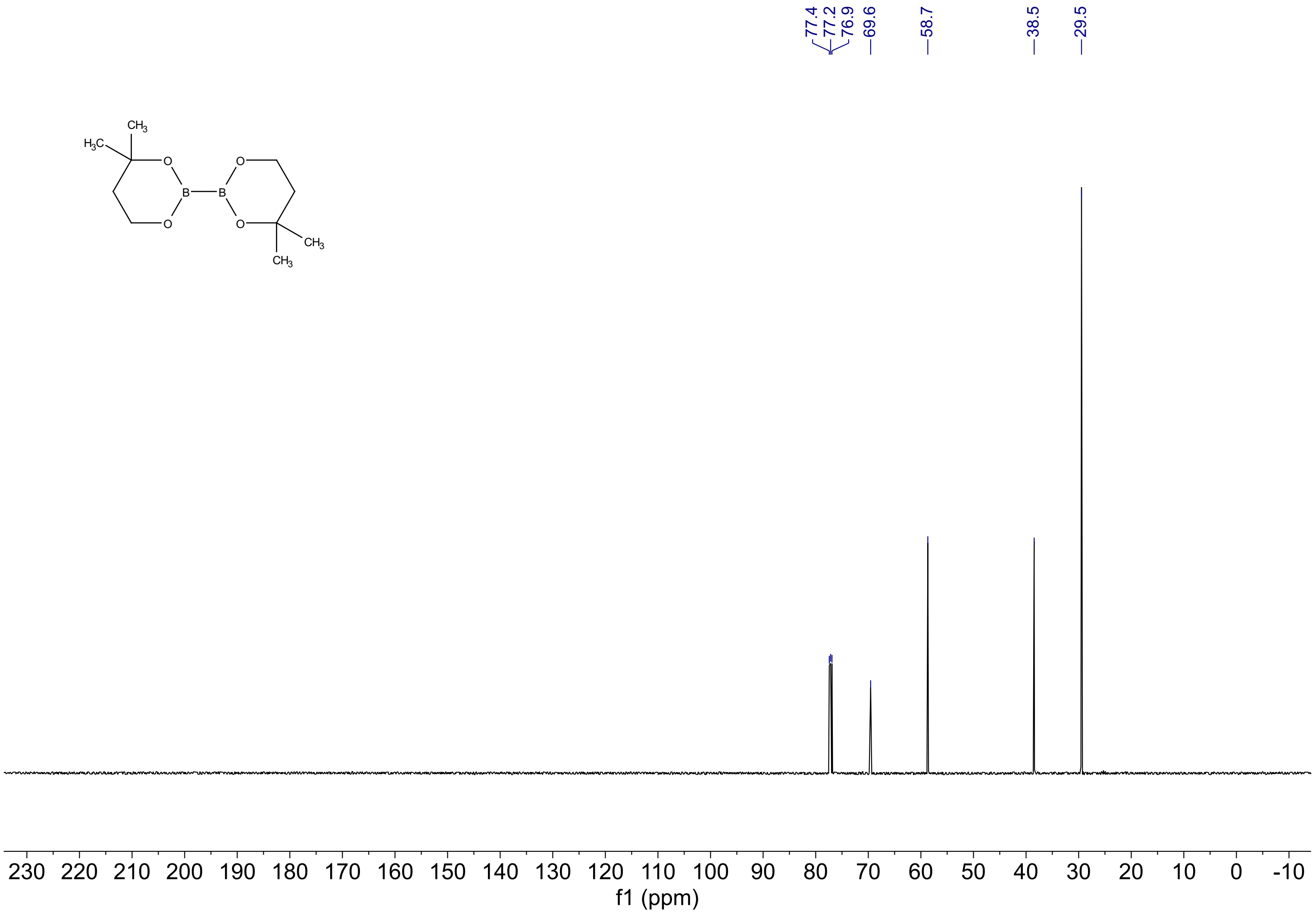

^{11}B NMR of B_2mbg_2 (4,4,4',4'-tetramethyl-2,2'-bi(1,3,2)dioxaborinane) ($CDCl_3$, 160 MHz)

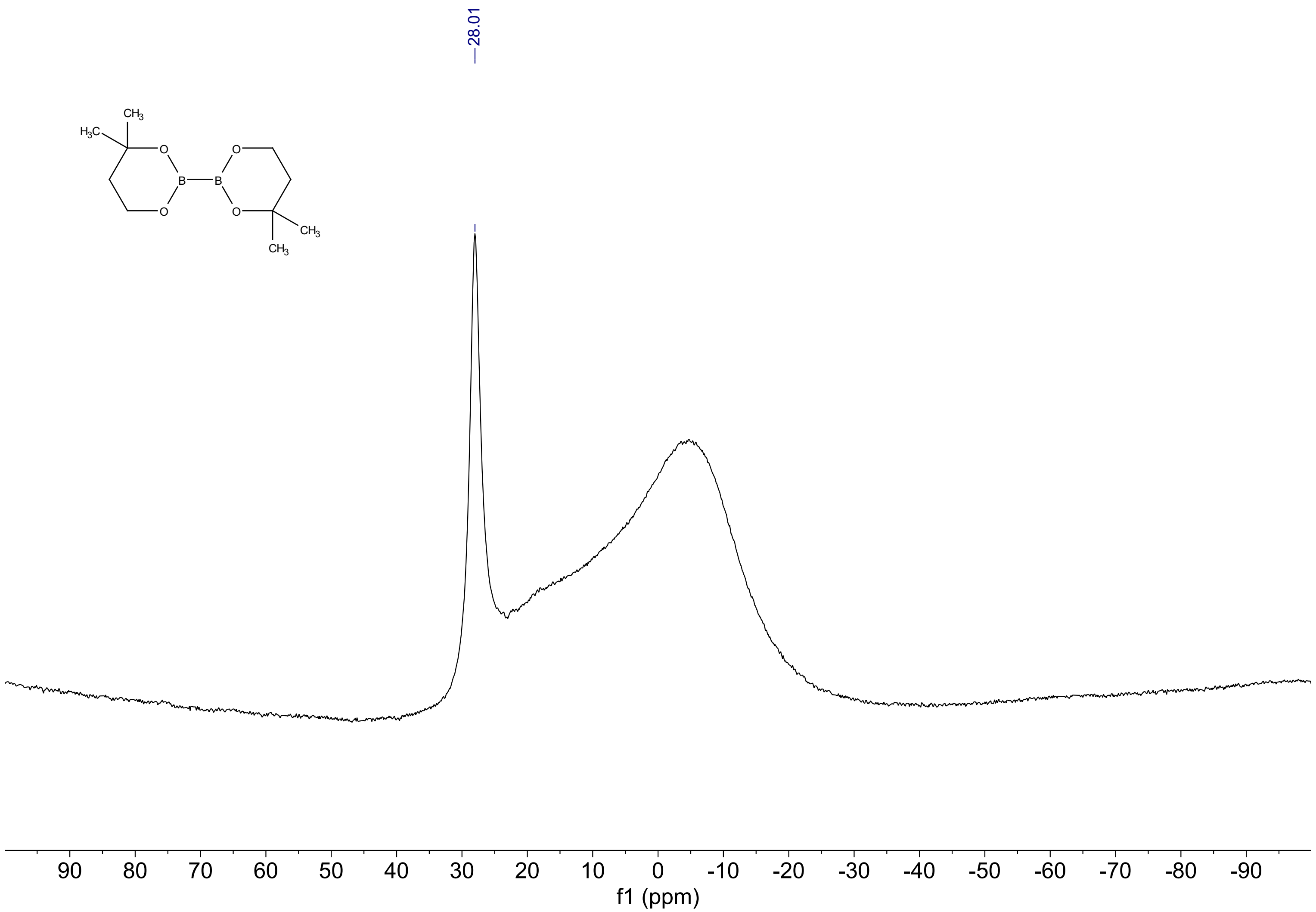

^{1}H NMR of (*E*)-*N*-(1-(difluoroboraneyl)ethyl)-*N*-ethyl-*N*'-methylacetimidamide (4a) ($CDCl_3$, 500 MHz)

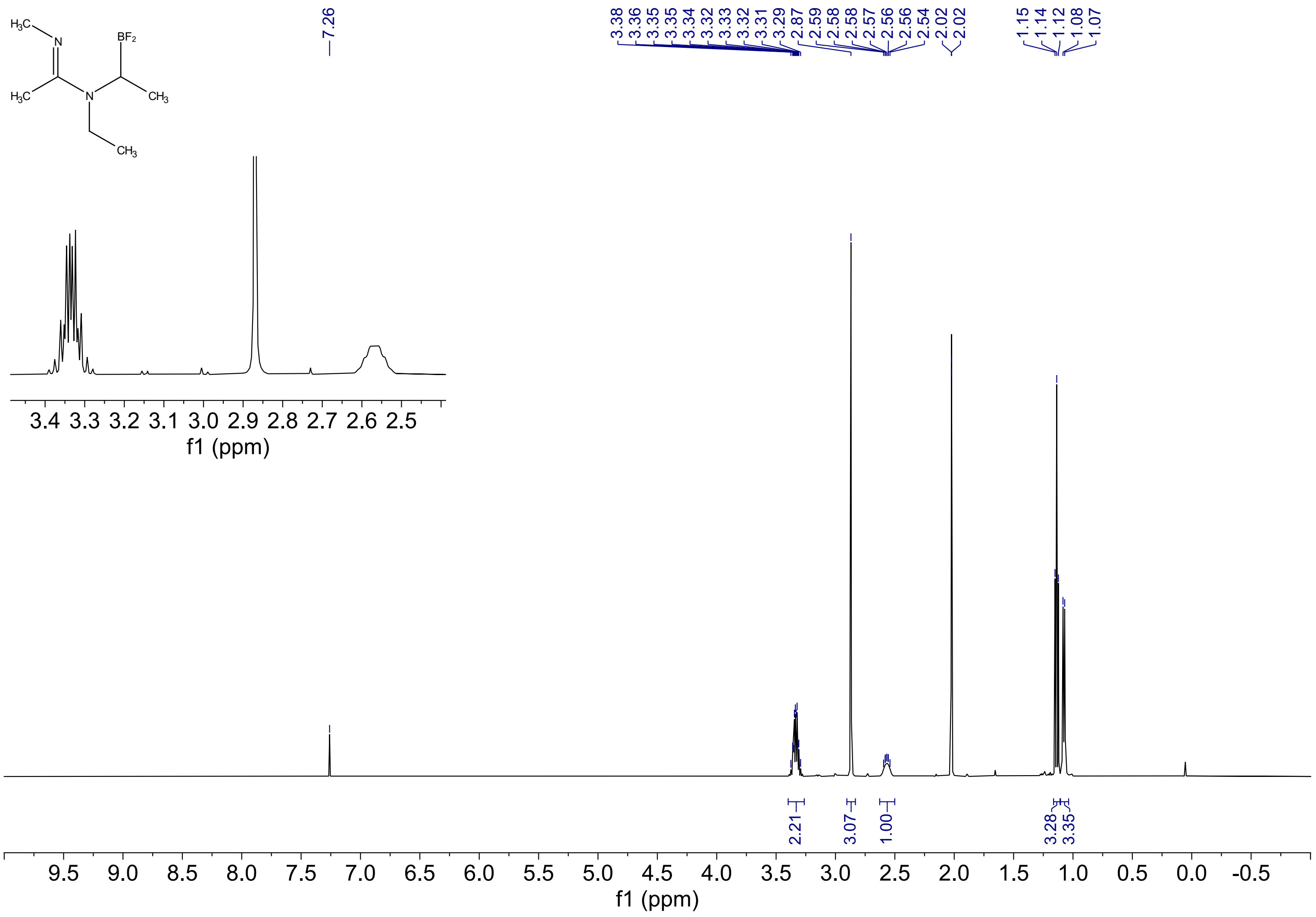

$^{13}C\{^1H\}$ NMR of (*E*)-*N*-(1-(difluoroboraneyl)ethyl)-*N*-ethyl-*N*'-methylacetimidamide (4a) ($CDCl_3$, 126 MHz)

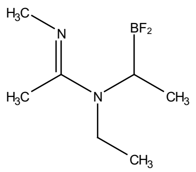

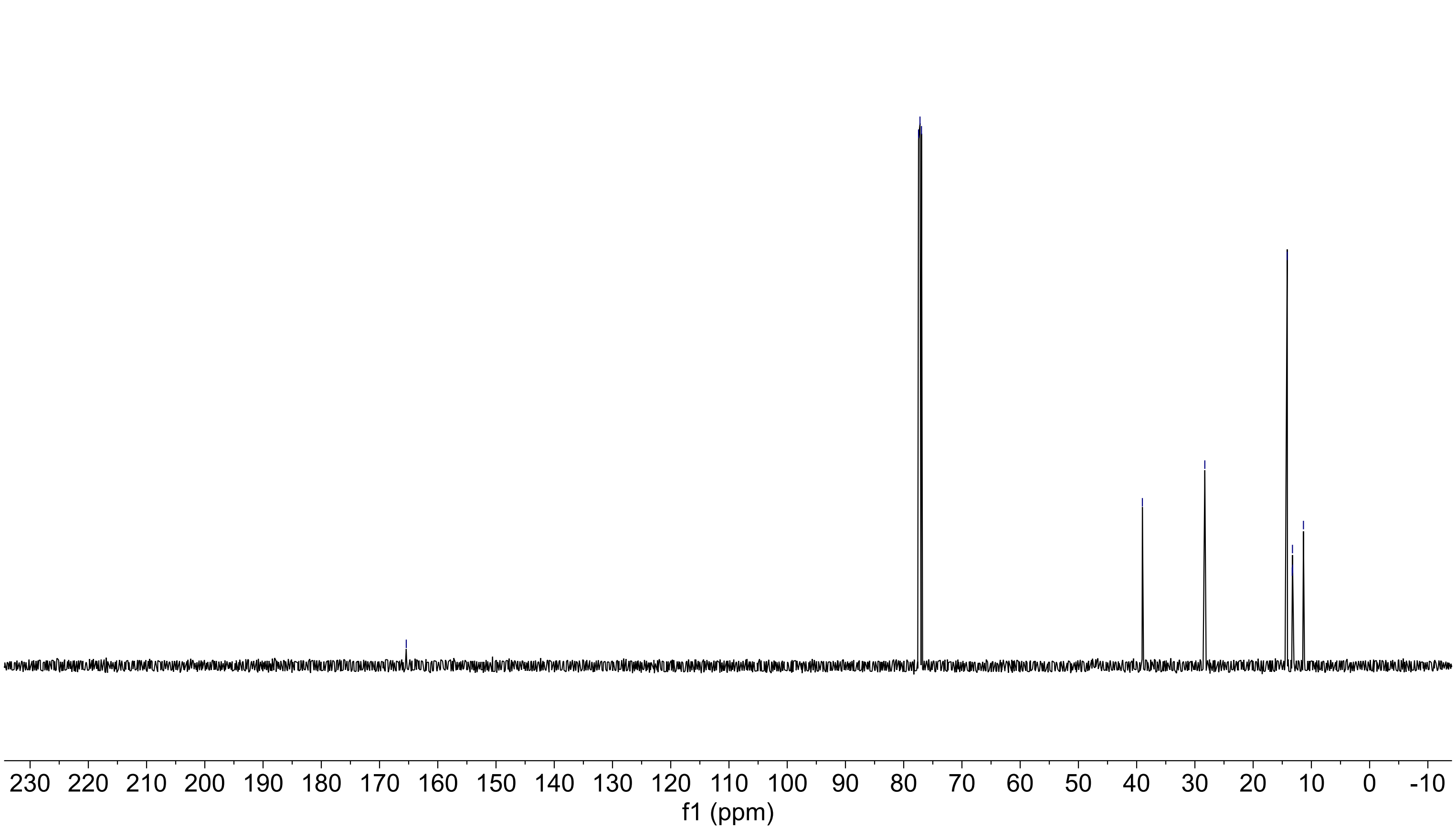

^{11}B NMR of (*E*)-*N*-(1-(difluoroboraneyl)ethyl)-*N*-ethyl-*N*'-methylacetimidamide (4a) ($CDCl_3$, 160 MHz)

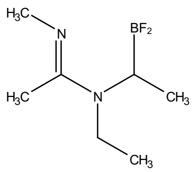

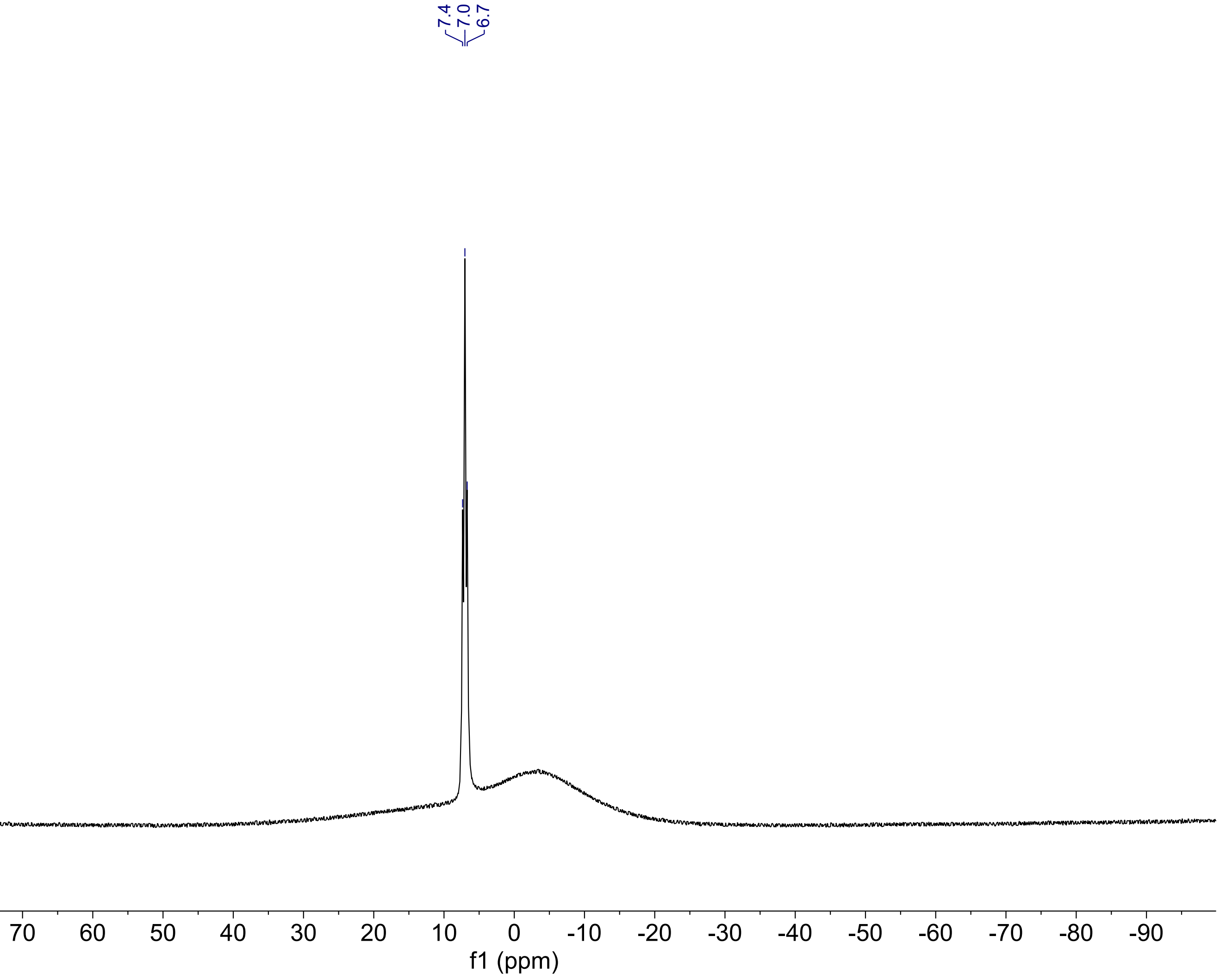

^{19}F NMR of (*E*)-*N*-(1-(difluoroboraneyl)ethyl)-*N*-ethyl-*N'*-methylacetimidamide (4a) ($CDCl_3$, 470 MHz)

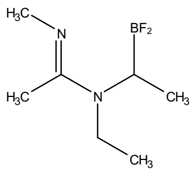

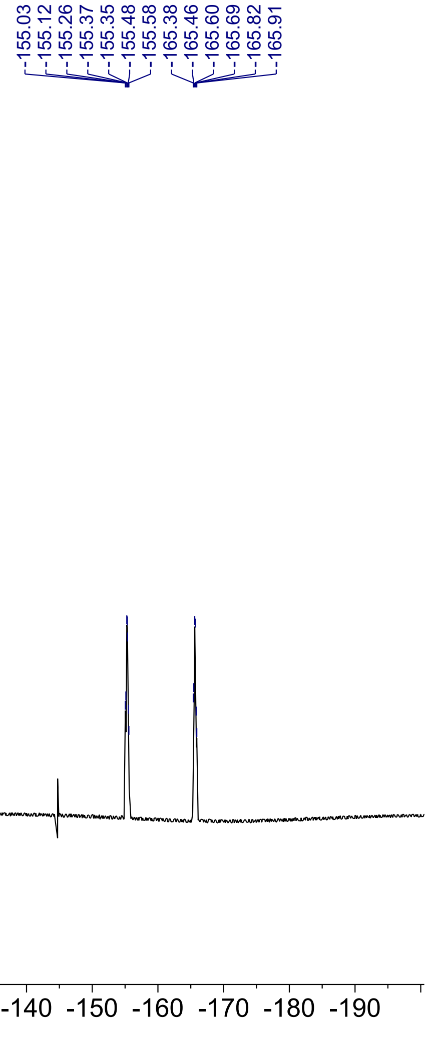

^{1}H NMR of (*E*)-*N*-(1-(difluoroboraneyl)ethyl)-*N*-ethyl-*N*'-methylpropionimidamide (4b) ($CDCl_3$, 500 MHz)

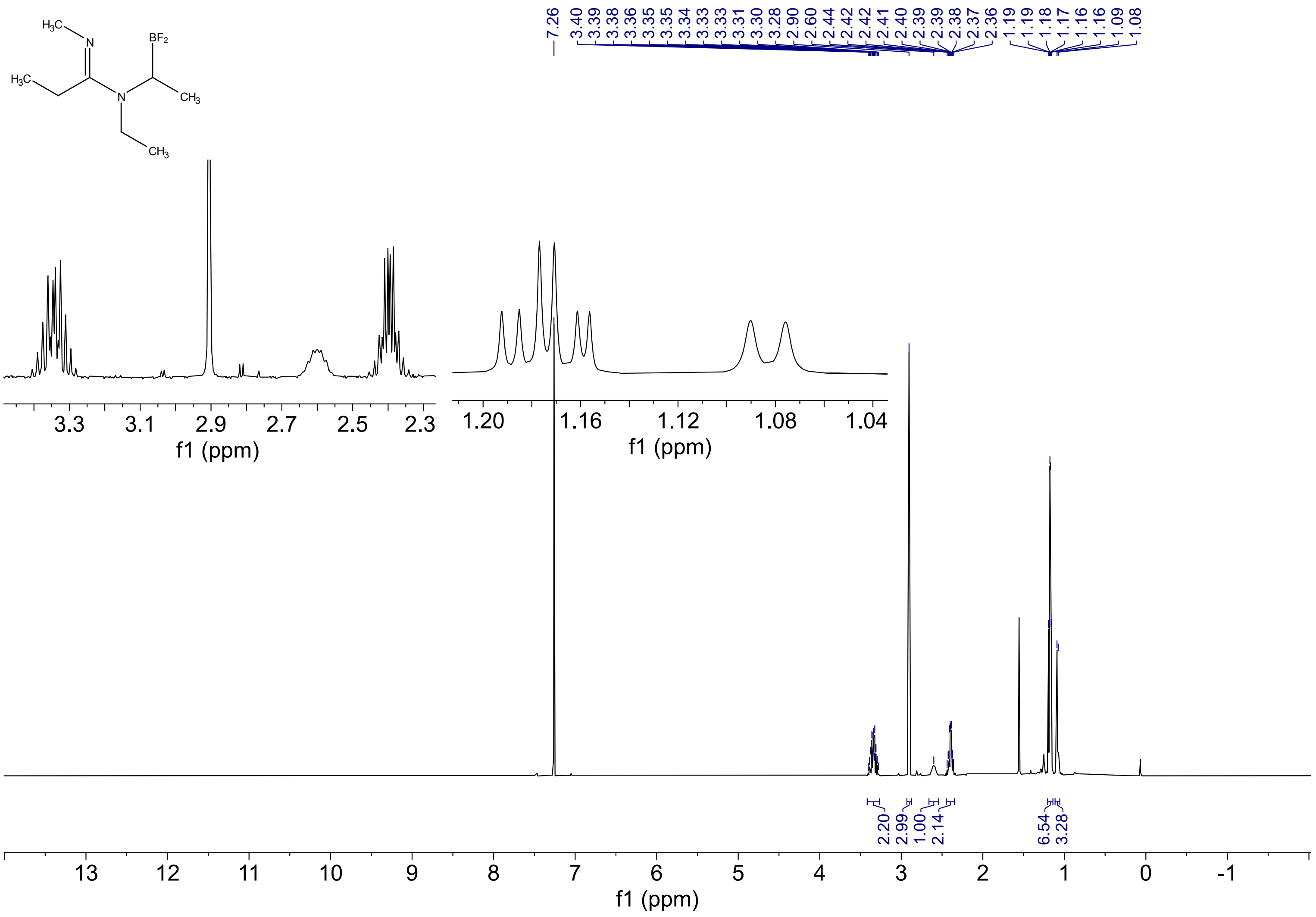

$^{13}C\{^1H\}$ NMR of (*E*)-*N*-(1-(difluoroboraneyl)ethyl)-*N*-ethyl-*N'*-methylpropionimidamide (4b) ($CDCl_3$, 126 MHz)

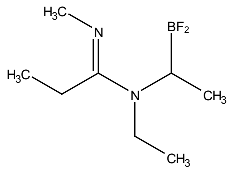

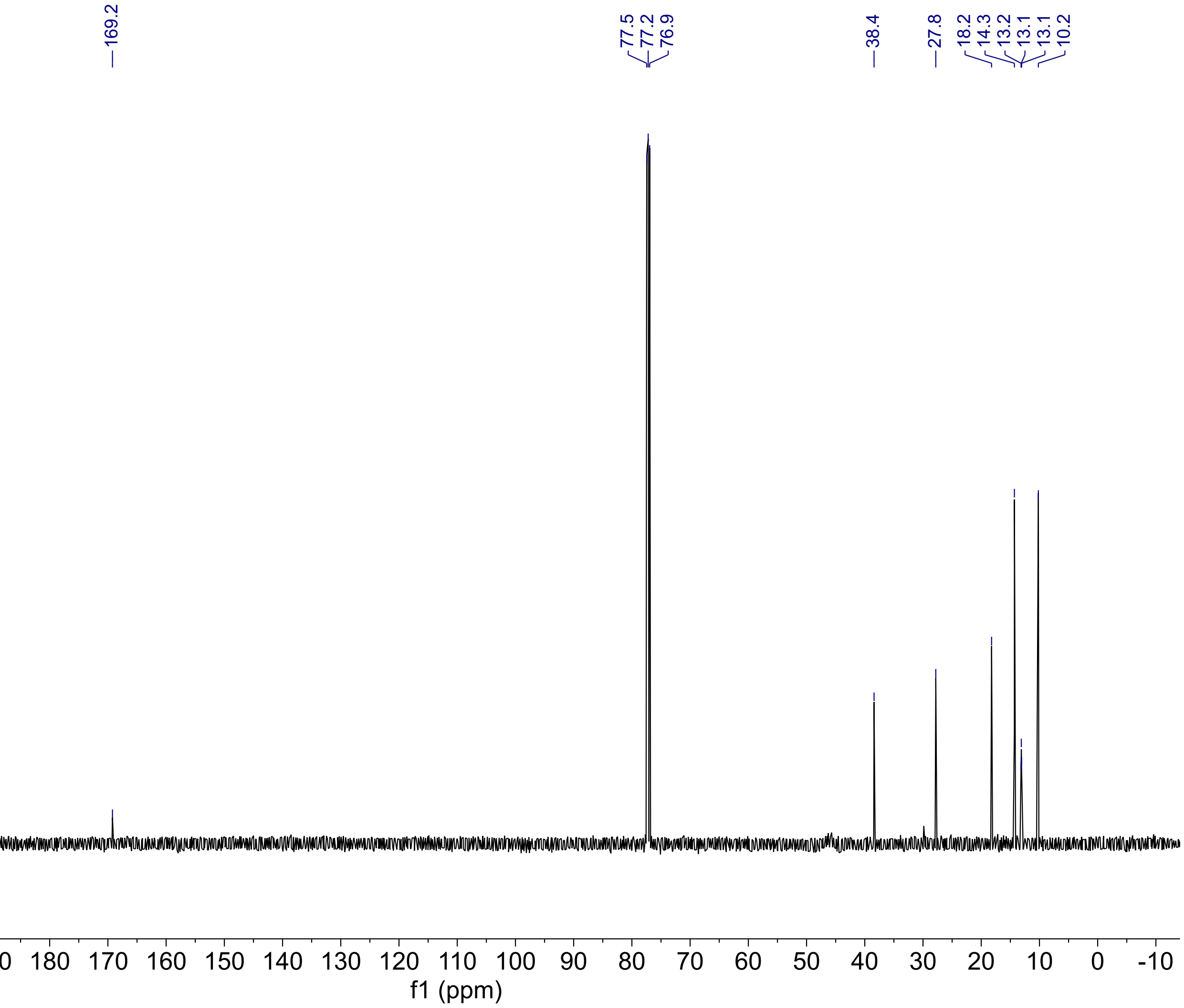

^{11}B NMR of (*E*)-*N*-(1-(difluoroboraneyl)ethyl)-*N*-ethyl-*N'*-methylpropionimidamide (4b) ($CDCl_3$, 160 MHz)

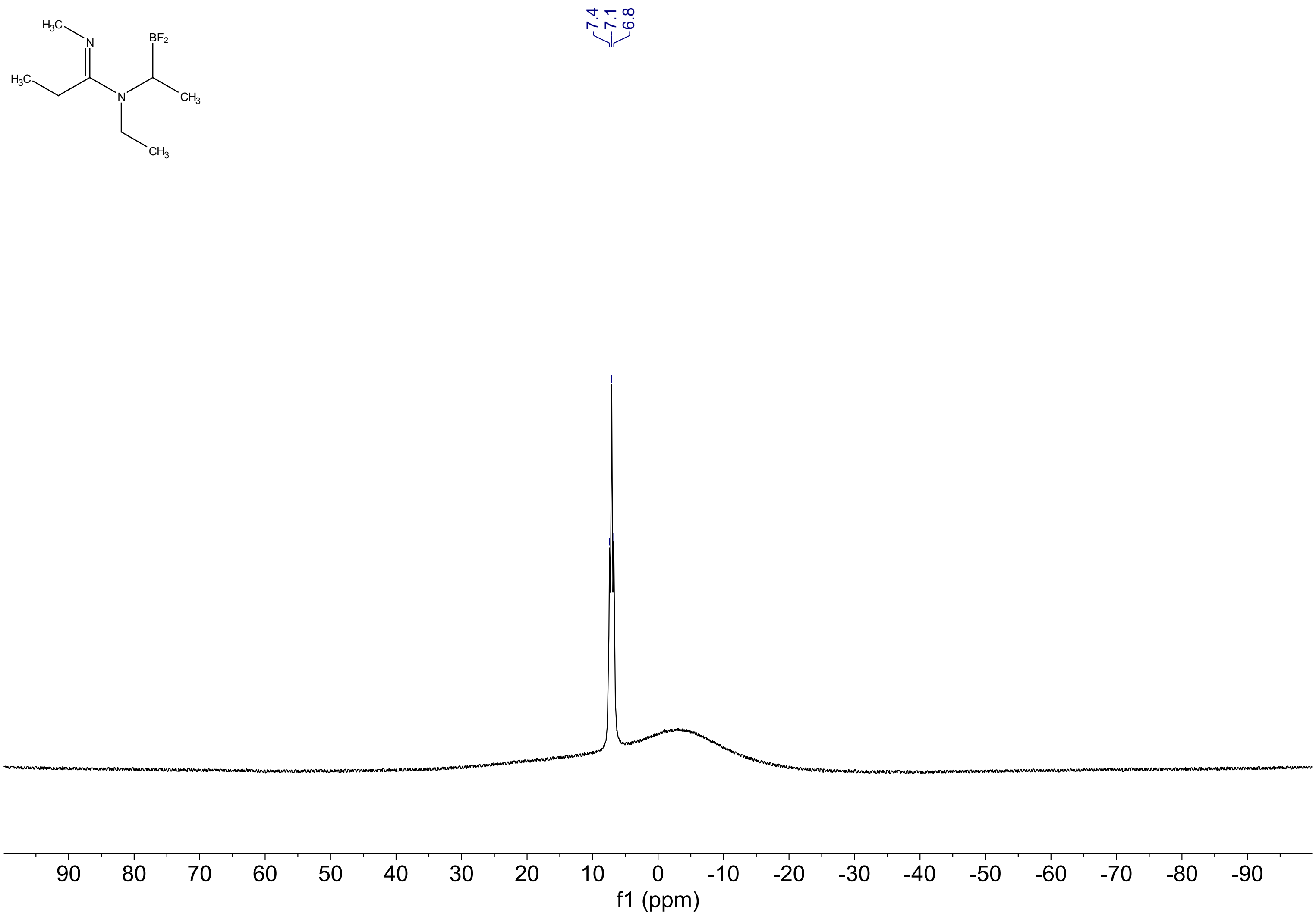

^{19}F NMR of (*E*)-*N*-(1-(difluoroboraneyl)ethyl)-*N*-ethyl-*N'*-methylpropionimidamide (4b) ($CDCl_3$, 470 MHz)

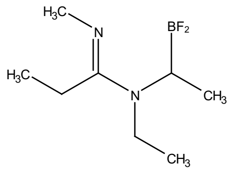

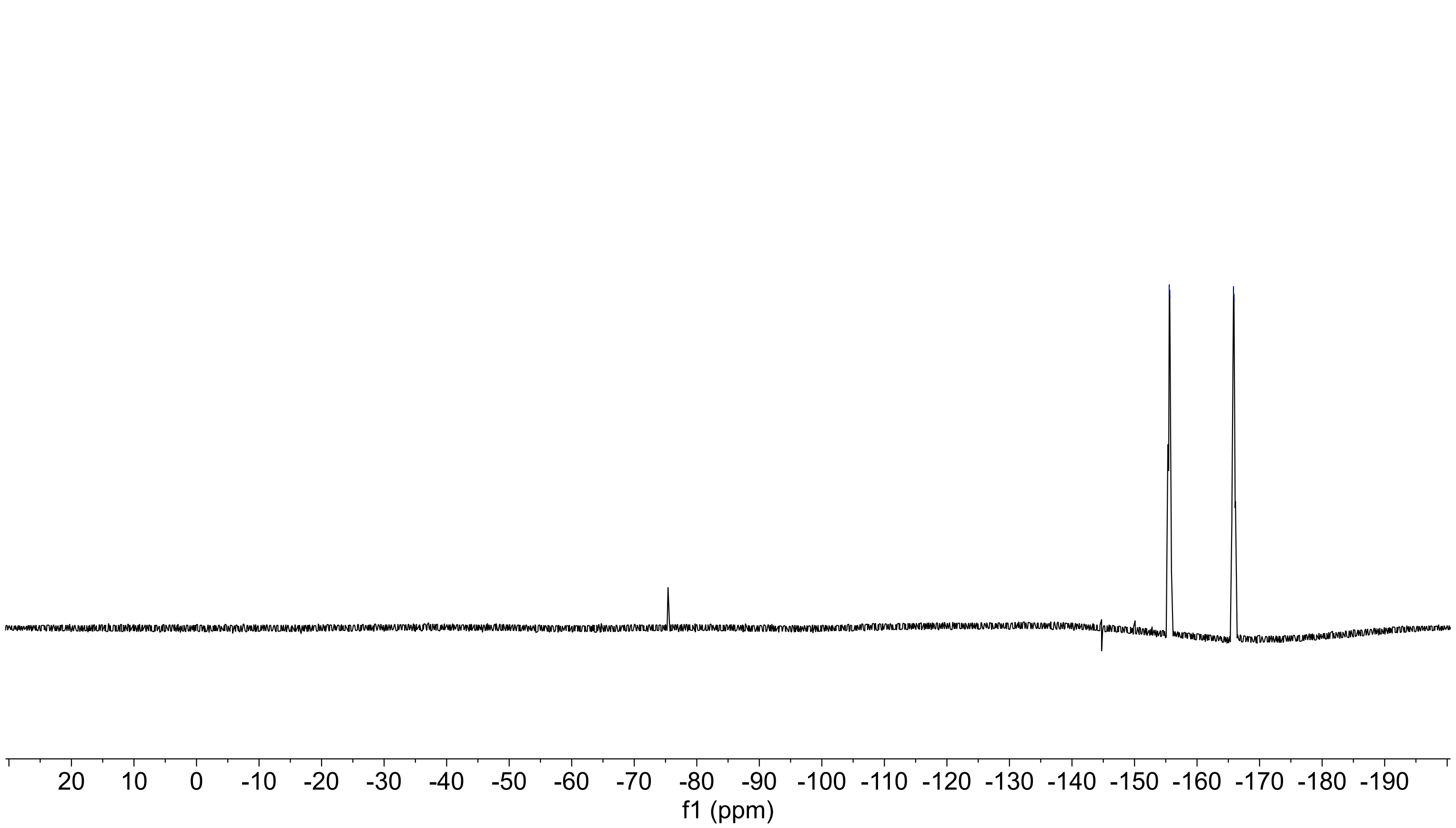

^{1}H NMR of (*E*)-*N*-(1-(difluoroboraneyl)ethyl)-*N*,*N*'-diethylacetimidamide (4c) ($CDCl_3$, 500 MHz)

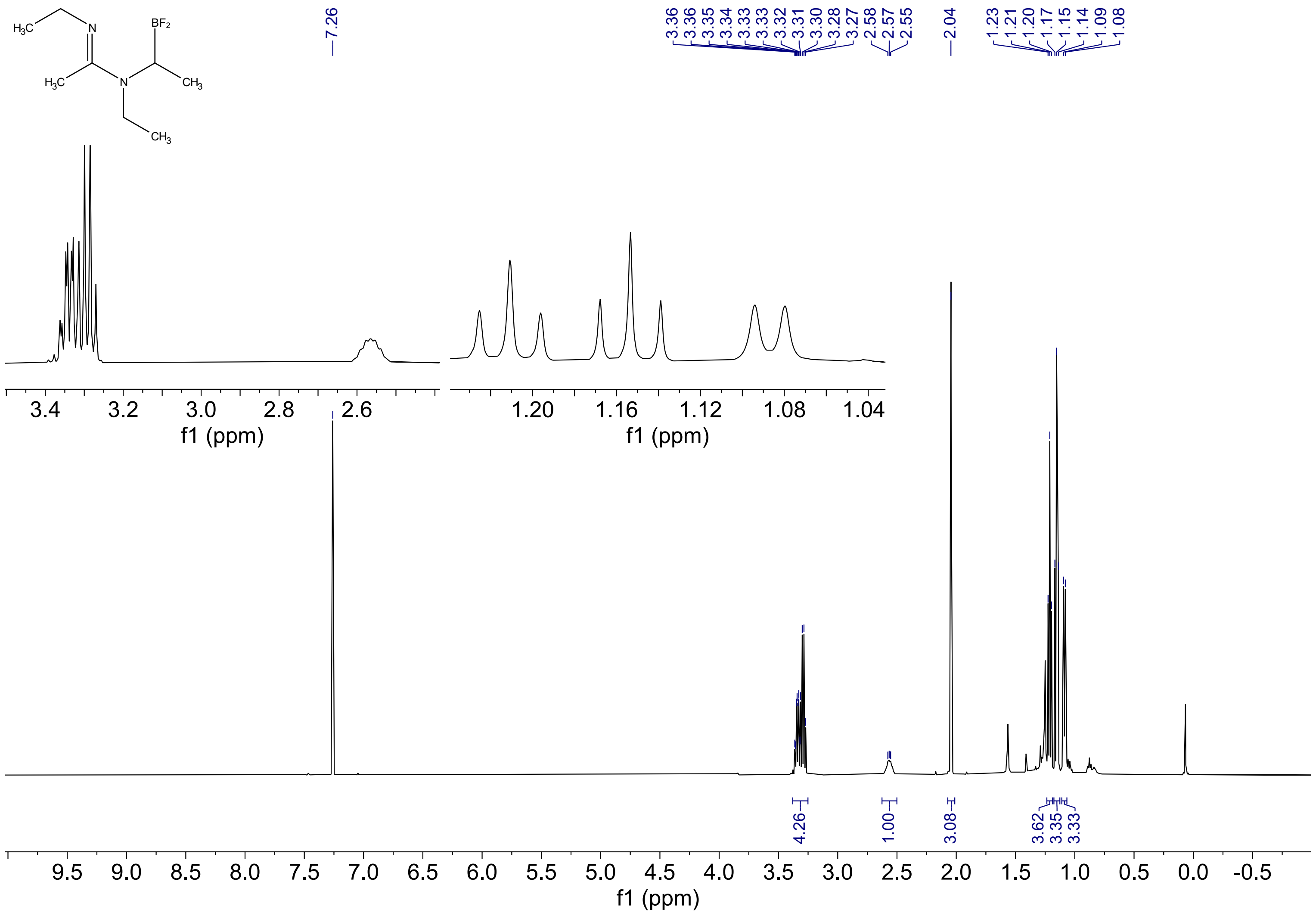

$^{13}C\{^1H\}$ NMR of (*E*)-*N*-(1-(difluoroboraneyl)ethyl)-*N*,*N*'-diethylacetimidamide (4c) ($CDCl_3$, 126 MHz)

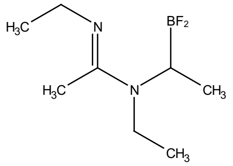

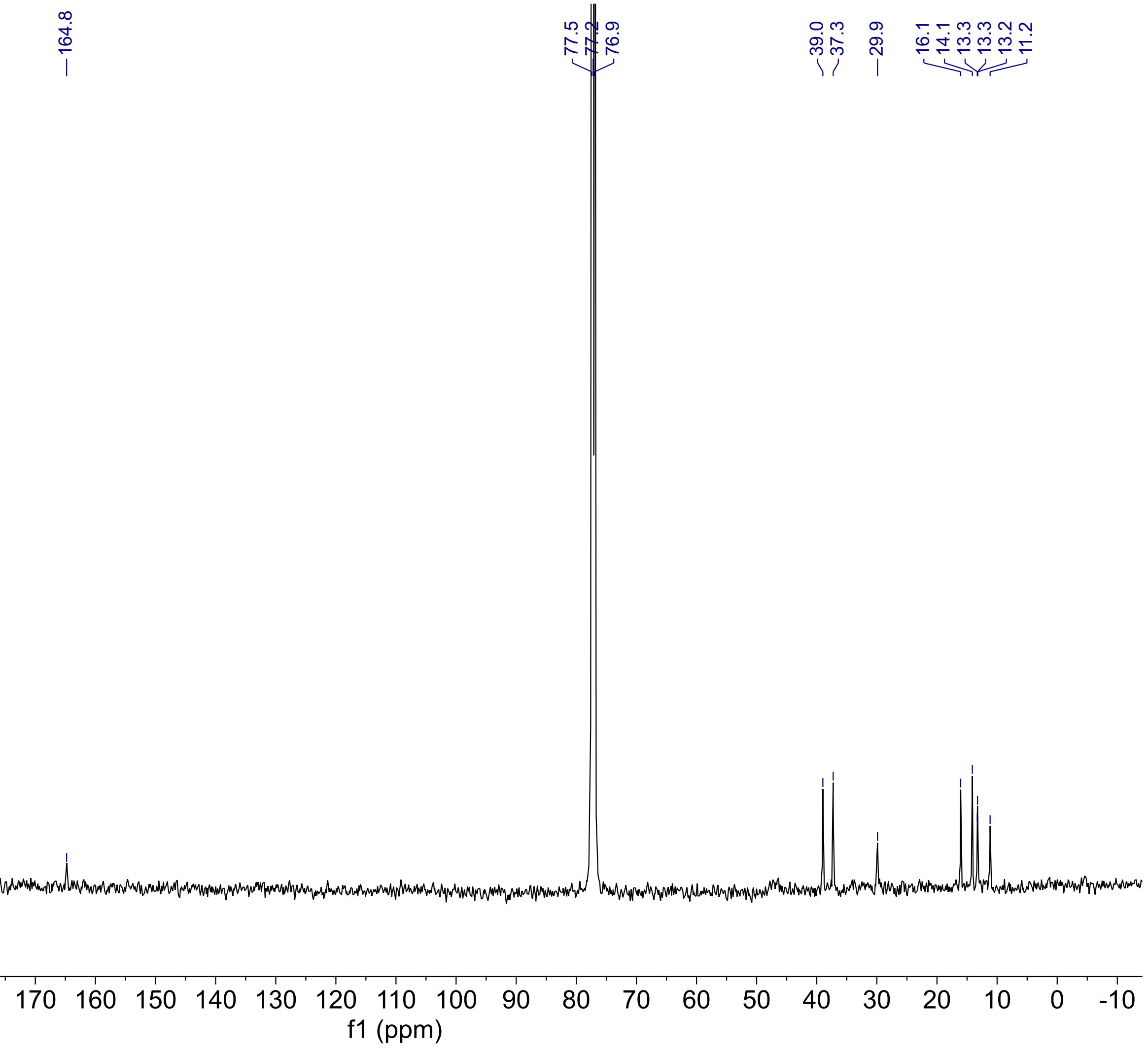

^{11}B NMR of (*E*)-*N*-(1-(difluoroboraneyl)ethyl)-*N*,*N'*-diethylacetimidamide (4c) ($CDCl_3$, 160 MHz)

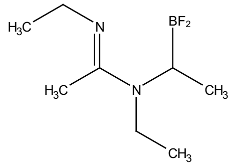

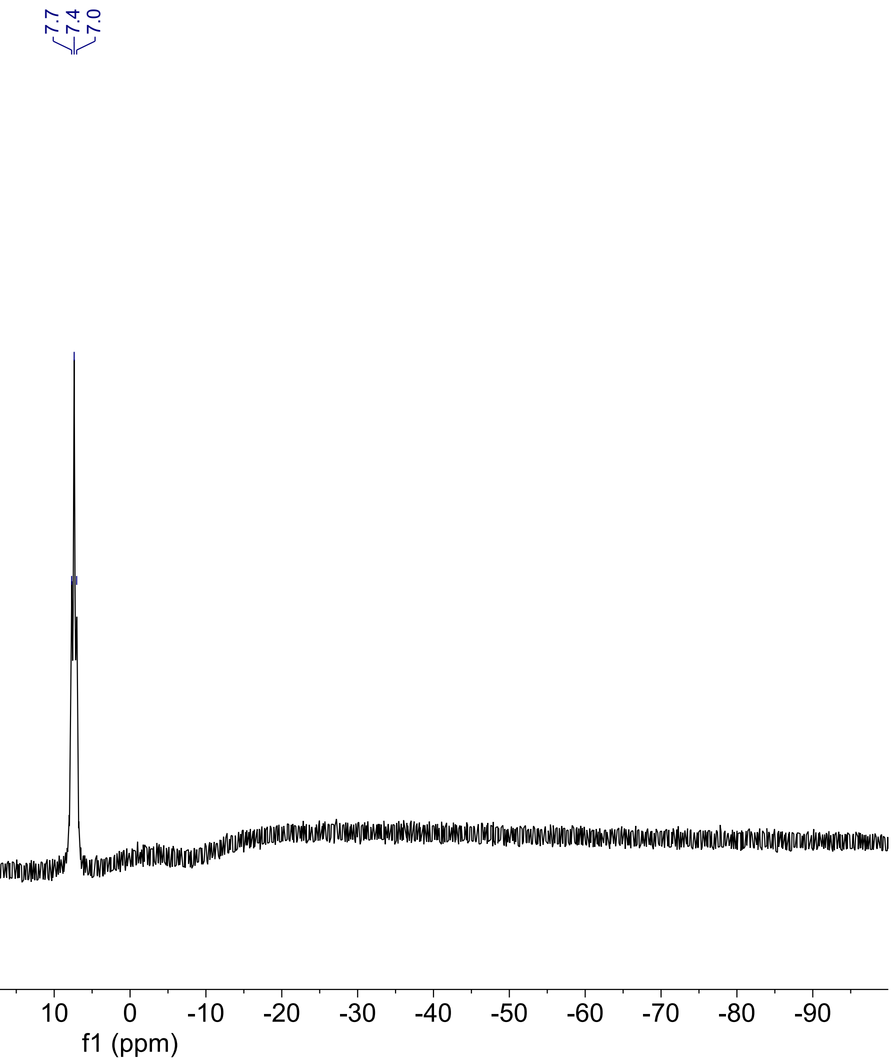

^{19}F NMR of (*E*)-*N*-(1-(difluoroboraneyl)ethyl)-*N*,*N*'-diethylacetimidamide (4c) ($CDCl_3$, 470 MHz)

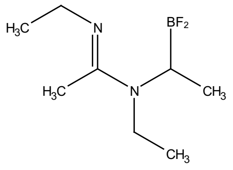

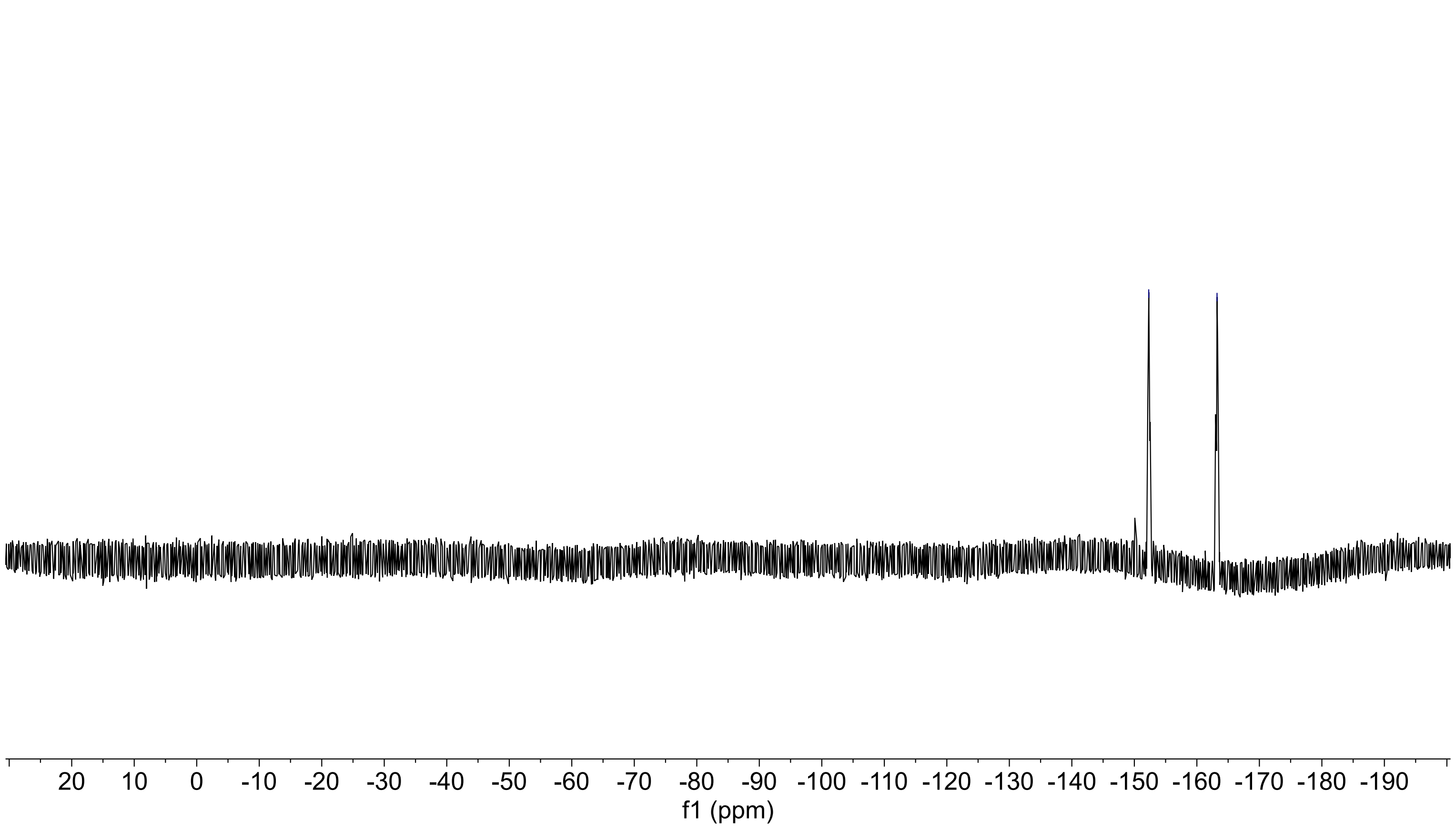

^{1}H NMR of (*E*)-*N*-(1-(difluoroboraneyl)ethyl)-*N*,*N*'-diethylpropionimidamide (4d) ($CDCl_3$, 500 MHz)

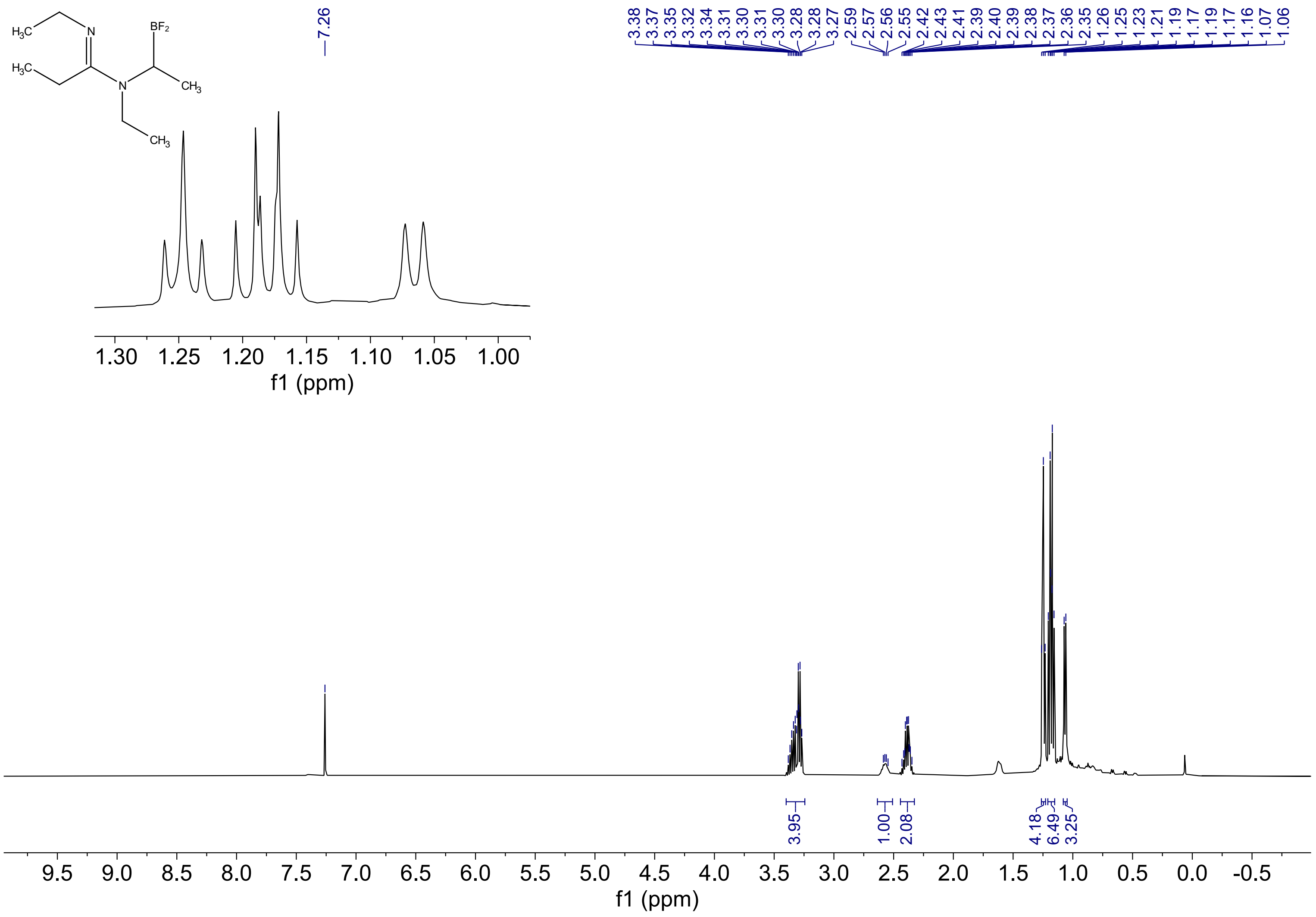

$^{13}C\{^{1}H\}$ NMR of (*E*)-*N*-(1-(difluoroboraneyl)ethyl)-*N*,*N*'-diethylpropionimidamide (4d) ($CDCl_3$, 126 MHz)

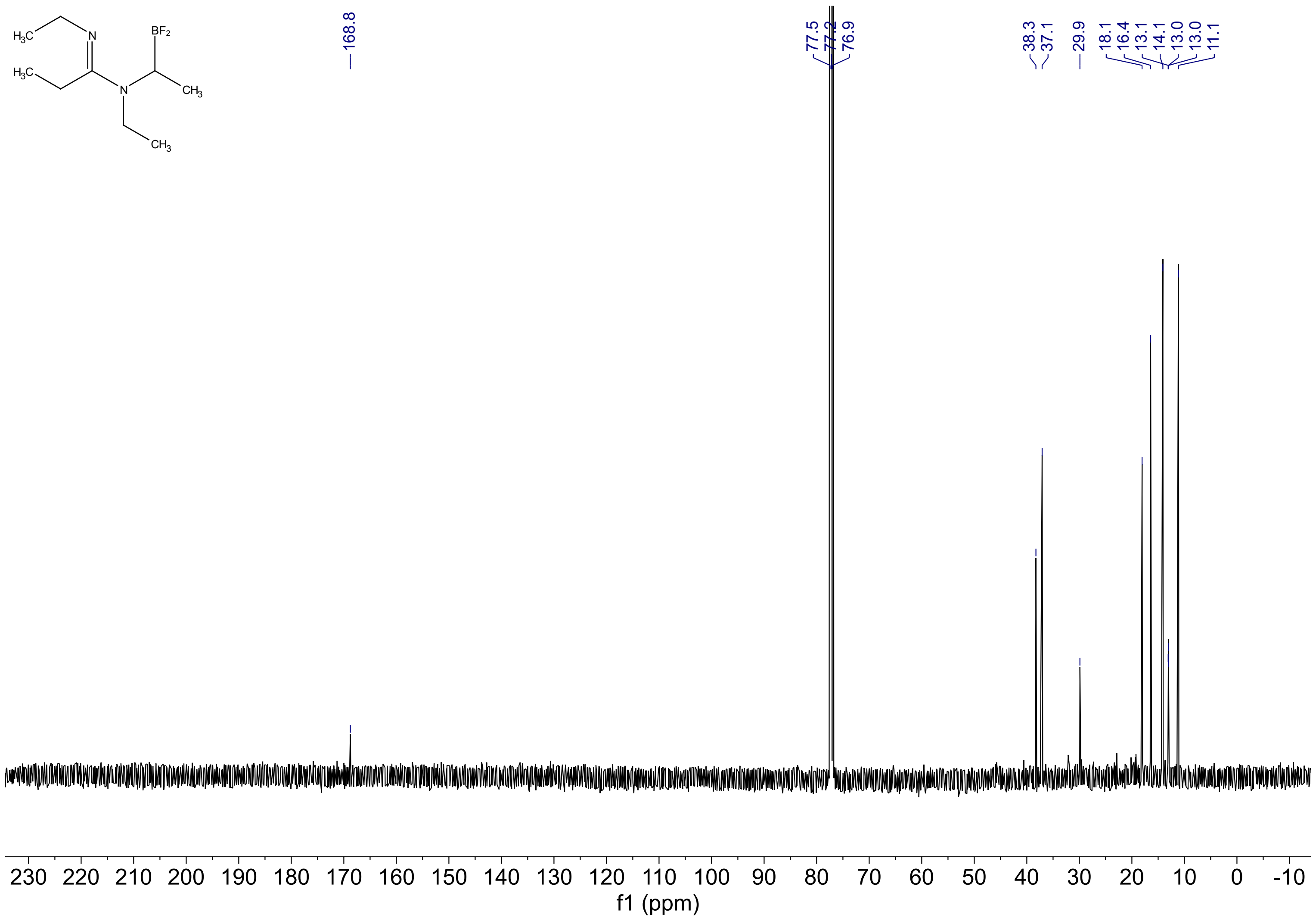

^{11}B NMR of (*E*)-*N*-(1-(difluoroboraneyl)ethyl)-*N*,*N*'-diethylpropionimidamide (4d) ($CDCl_3$, 160 MHz)

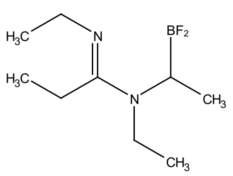

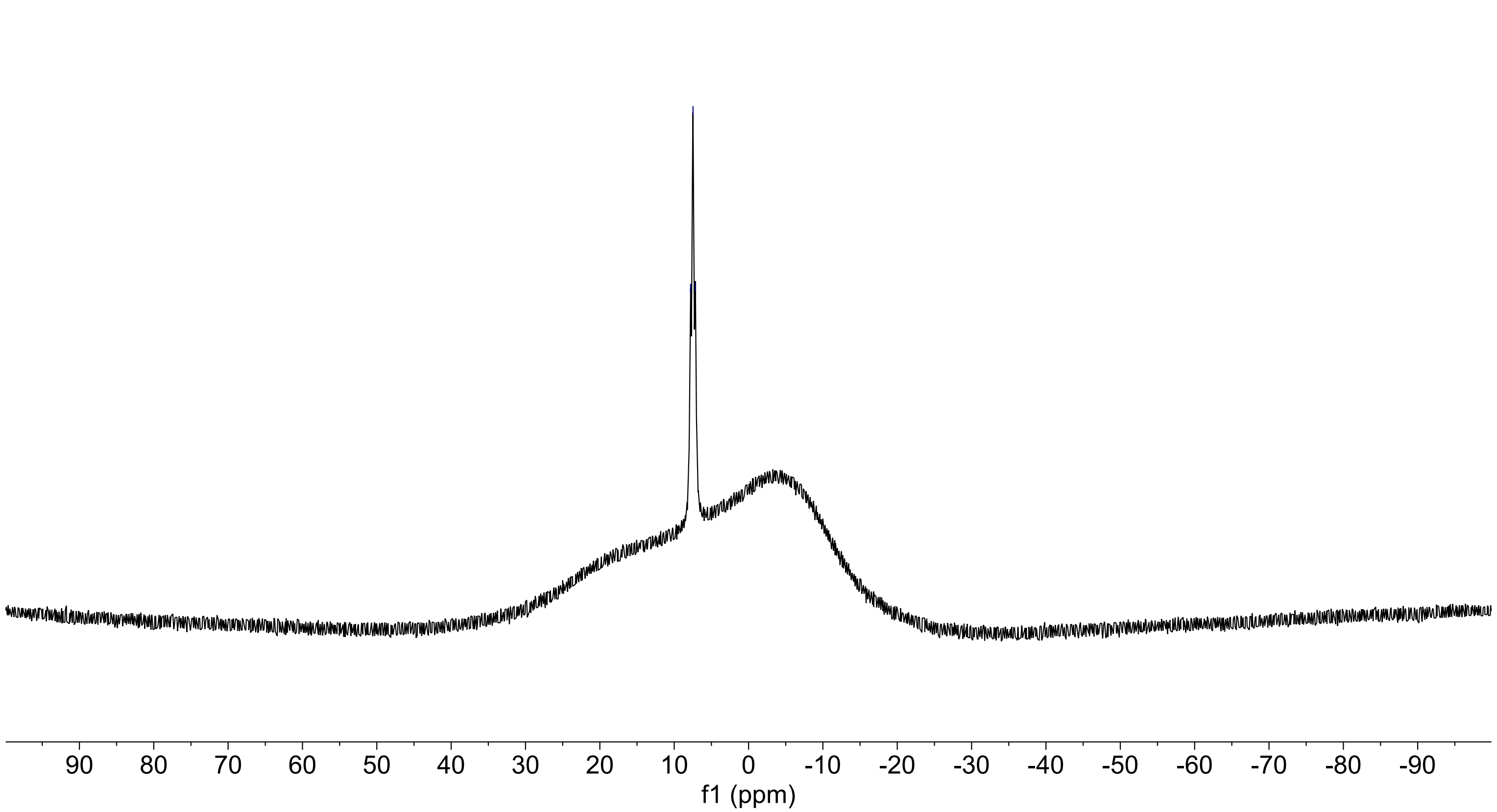

^{19}F NMR of (*E*)-*N*-(1-(difluoroboraneyl)ethyl)-*N*,*N*'-diethylpropionimidamide (4d) ($CDCl_3$, 470 MHz)

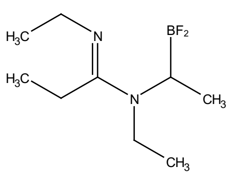

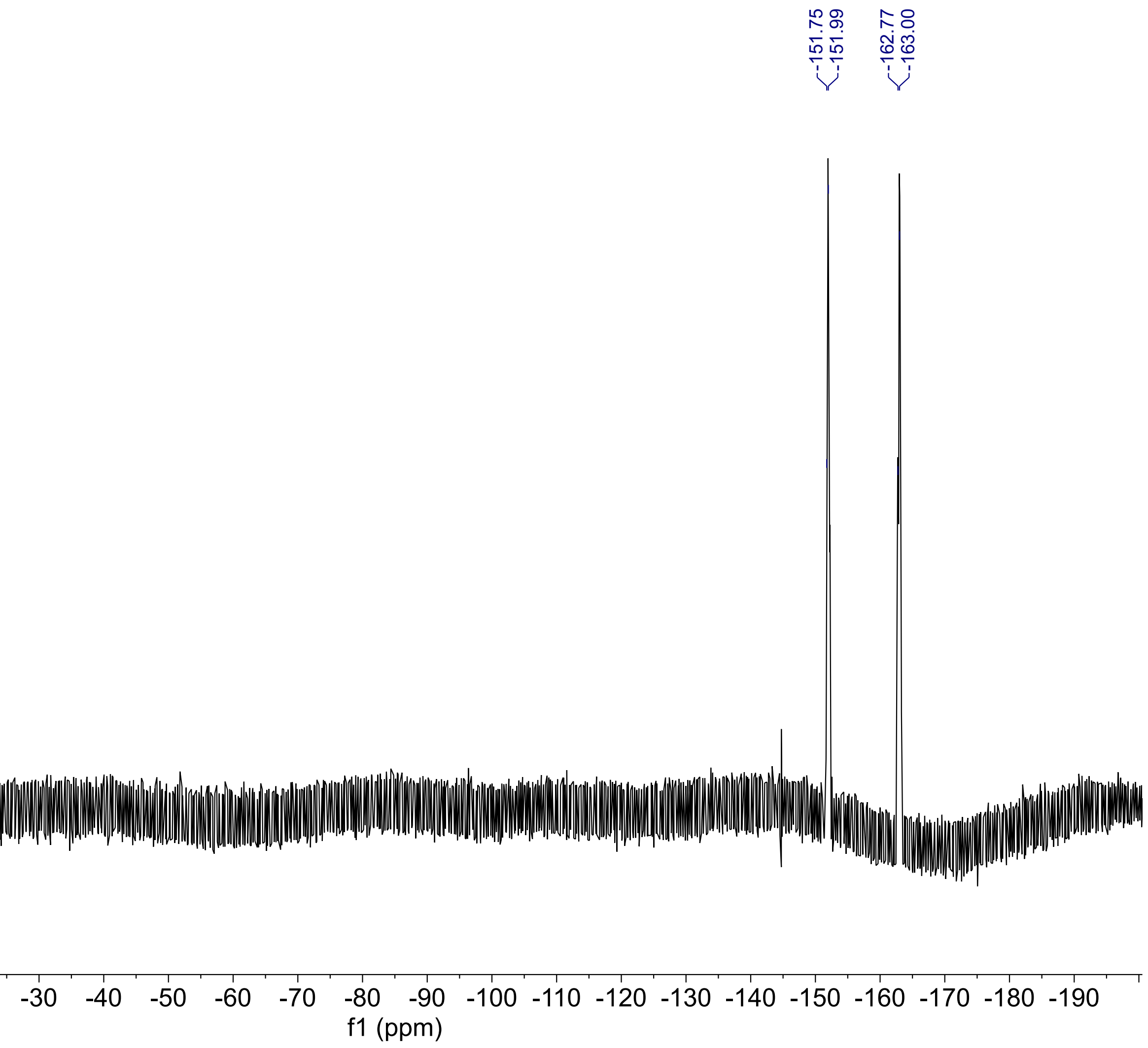

^{1}H NMR of (*E*)-1-(2-(difluoroboraneyl)piperidin-1-yl)-*N*-ethylethan-1-imine (4e) ($CDCl_3$, 500 MHz)

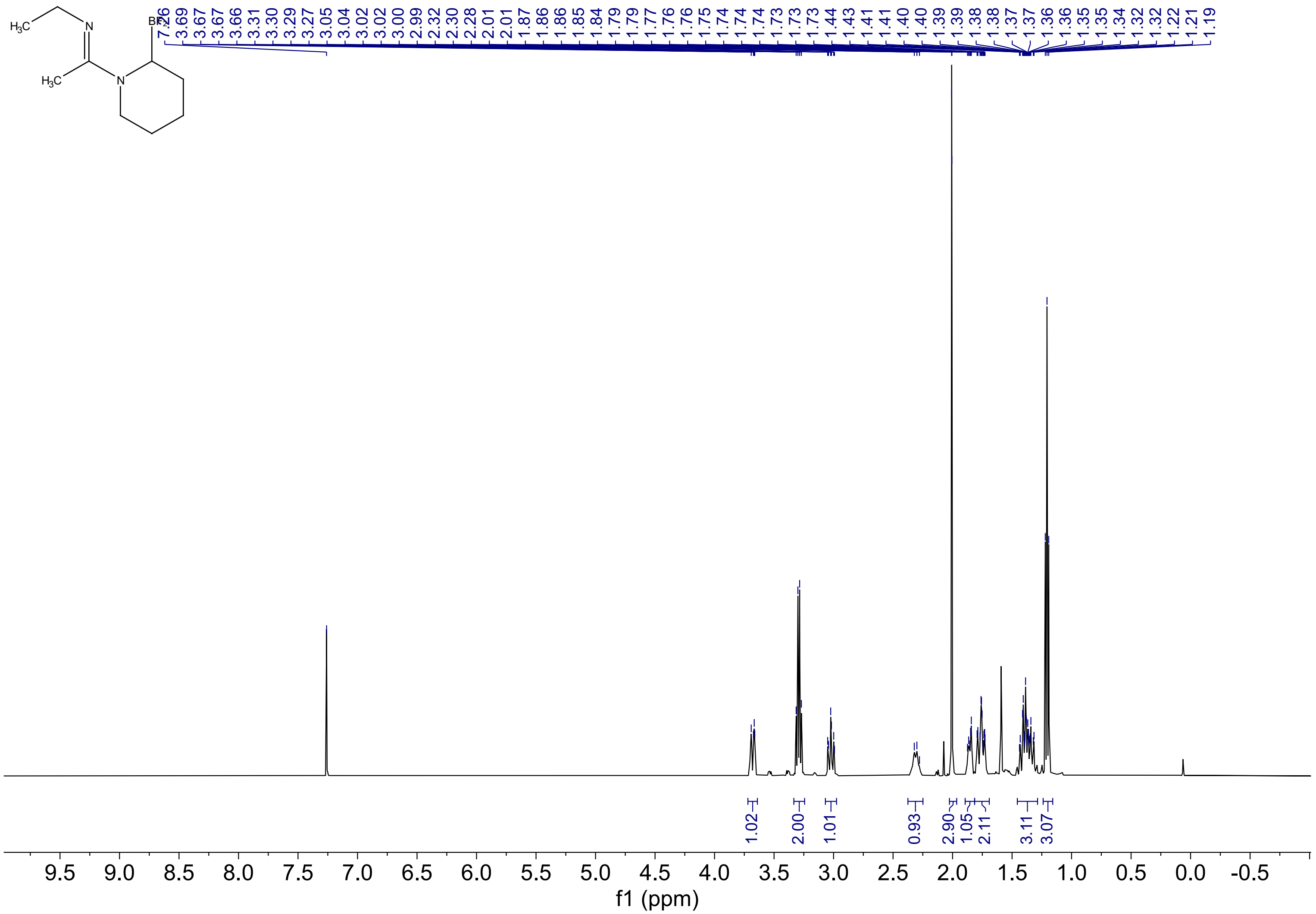

$^{13}C\{^{1}H\}$ NMR of (*E*)-1-(2-(difluoroboraneyl)piperidin-1-yl)-*N*-ethylethan-1-imine (4e) ($CDCl_3$, 126 MHz)

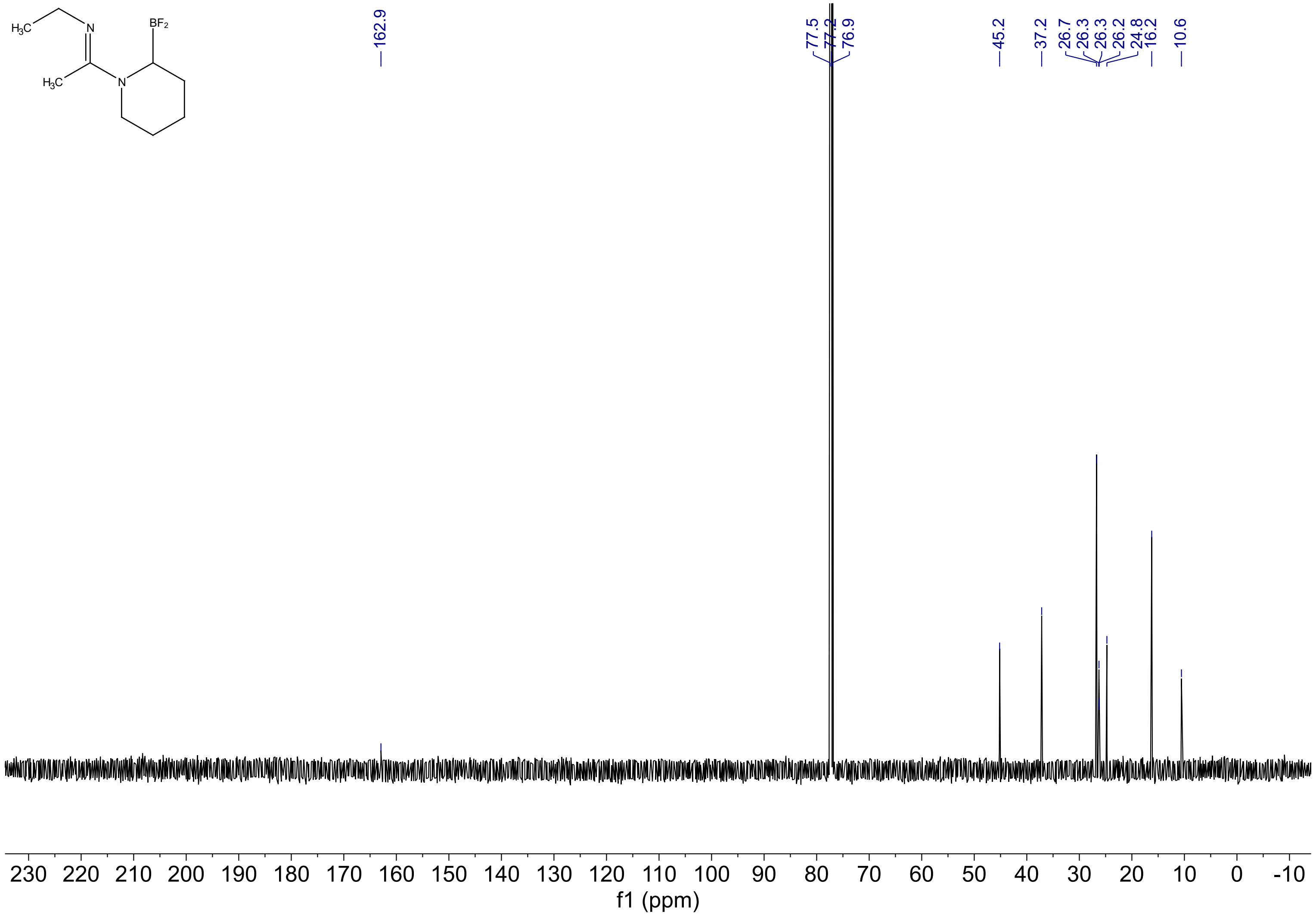

^{11}B NMR of (*E*)-1-(2-(difluoroboraneyl)piperidin-1-yl)-*N*-ethylethan-1-imine (4e) ($CDCl_3$, 160 MHz)

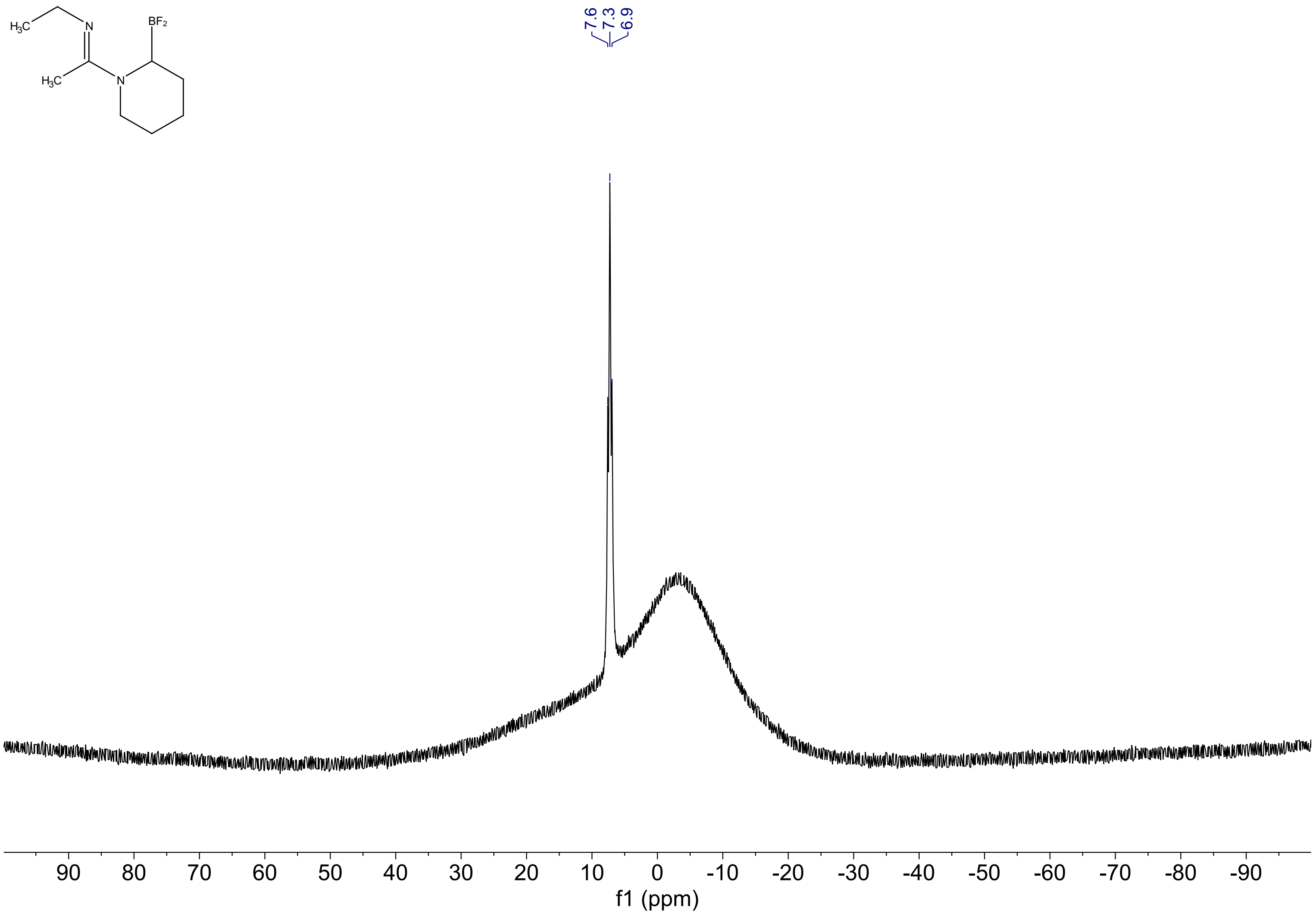

^{19}F NMR of (*E*)-1-(2-(difluoroboraneyl)piperidin-1-yl)-*N*-ethylethan-1-imine (4e) ($CDCl_3$, 470 MHz)

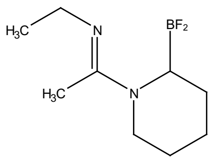

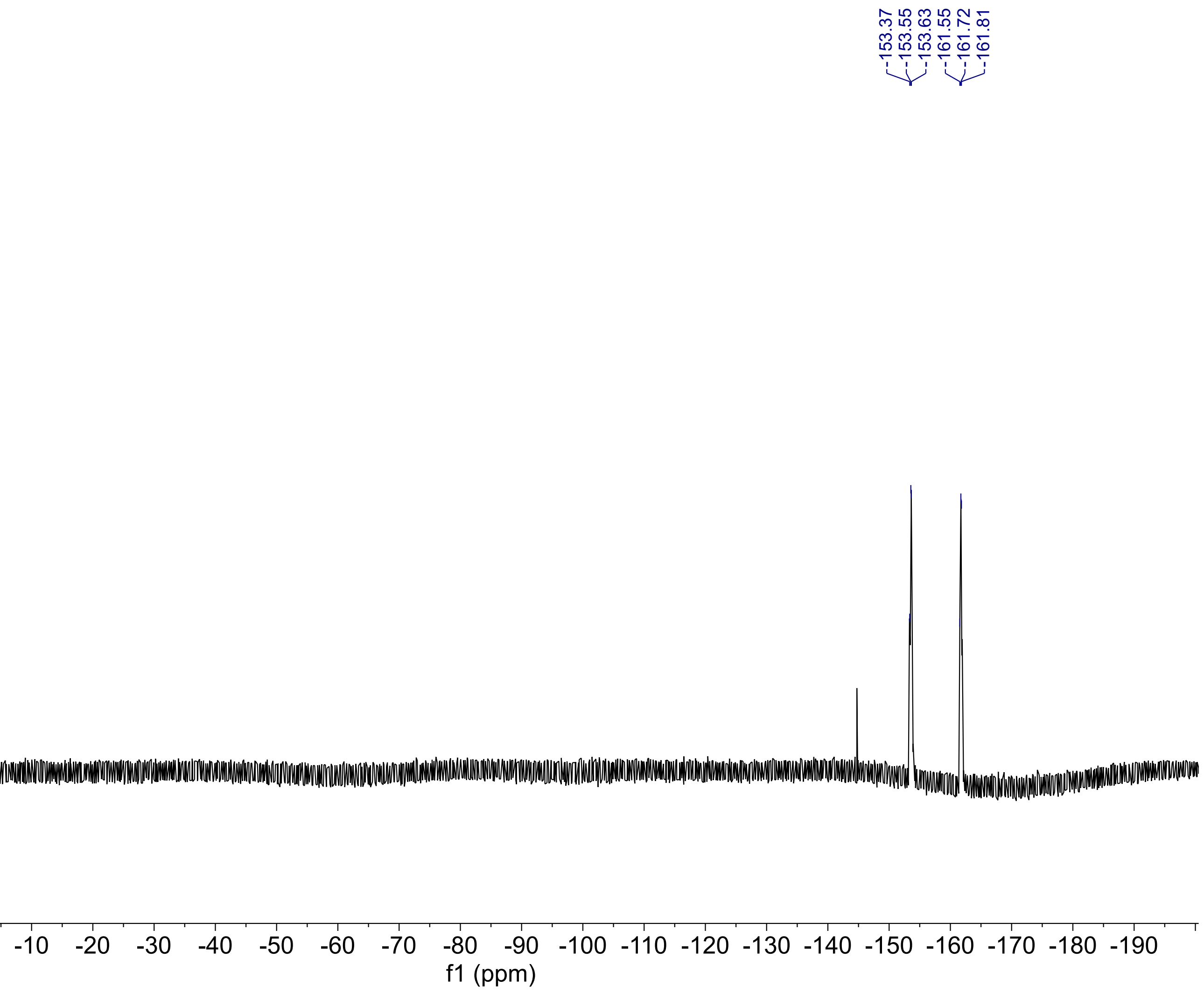

^{1}H NMR of *N*-(1-(difluoroboraneyl)ethyl)-*N*-ethyl-3,4-dihydro-2*H*-pyrrol-5-amine (4f) ($CDCl_3$, 500 MHz)

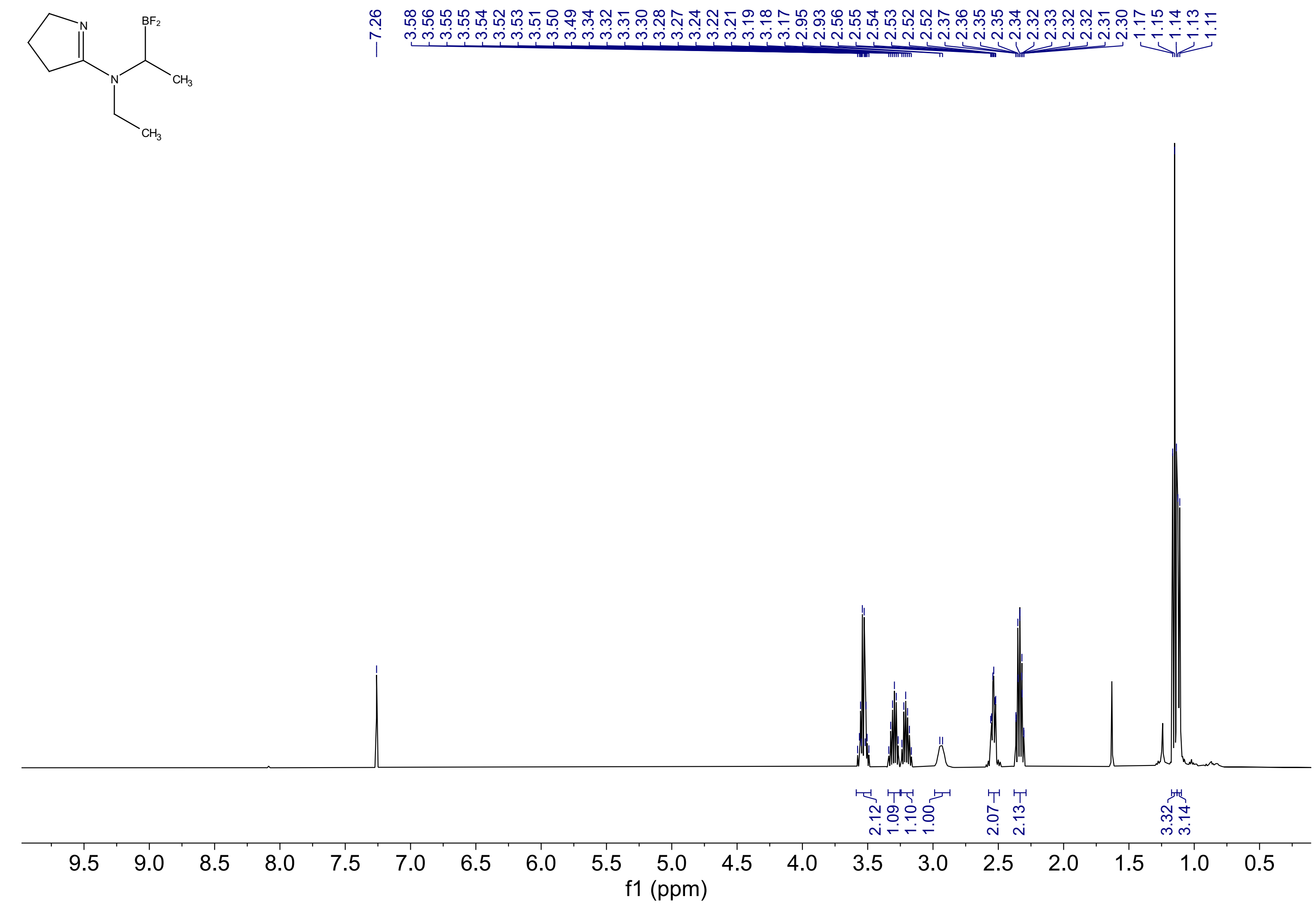

$^{13}C\{^1H\}$ NMR of *N*-(1-(difluoroboraneyl)ethyl)-*N*-ethyl-3,4-dihydro-2*H*-pyrrol-5-amine (4f) ($CDCl_3$, 126 MHz)

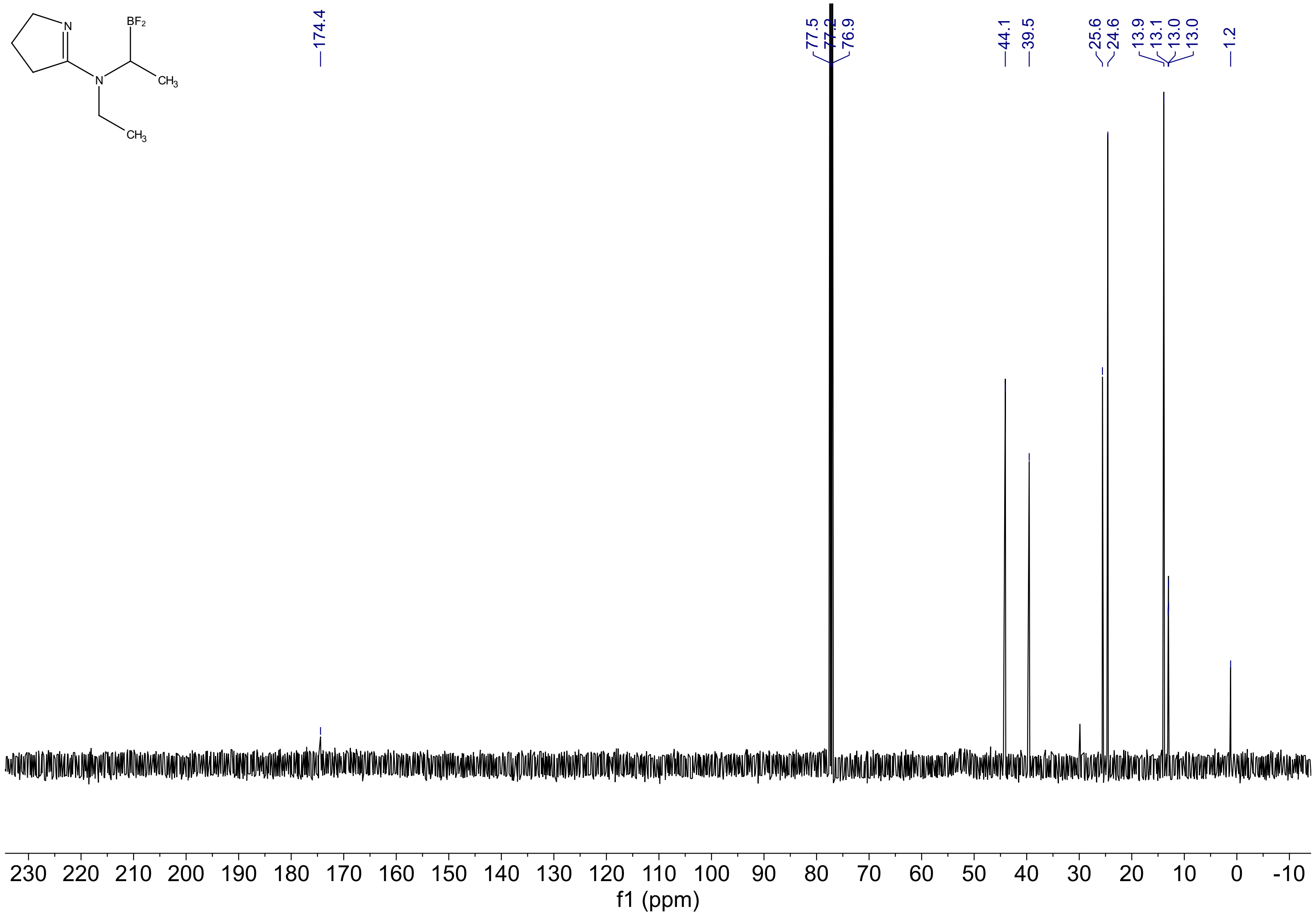

^{11}B NMR of *N*-(1-(difluoroboraneyl)ethyl)-*N*-ethyl-3,4-dihydro-2*H*-pyrrol-5-amine (4f) ($CDCl_3$, 160 MHz)

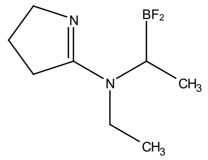

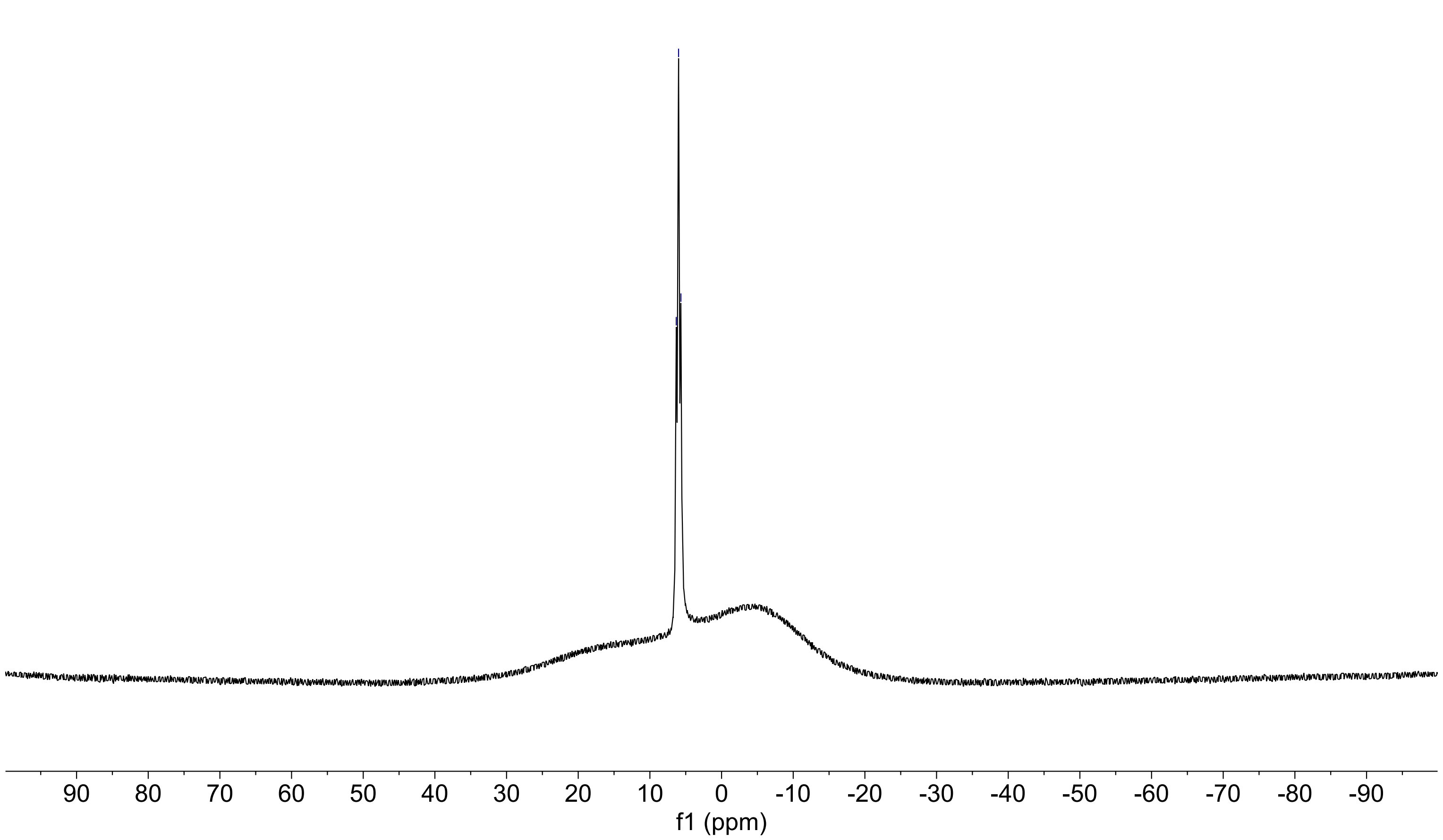

^{19}F NMR of *N*-(1-(difluoroboraneyl)ethyl)-*N*-ethyl-3,4-dihydro-2*H*-pyrrol-5-amine (4f) ($CDCl_3$, 470 MHz)

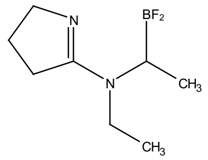

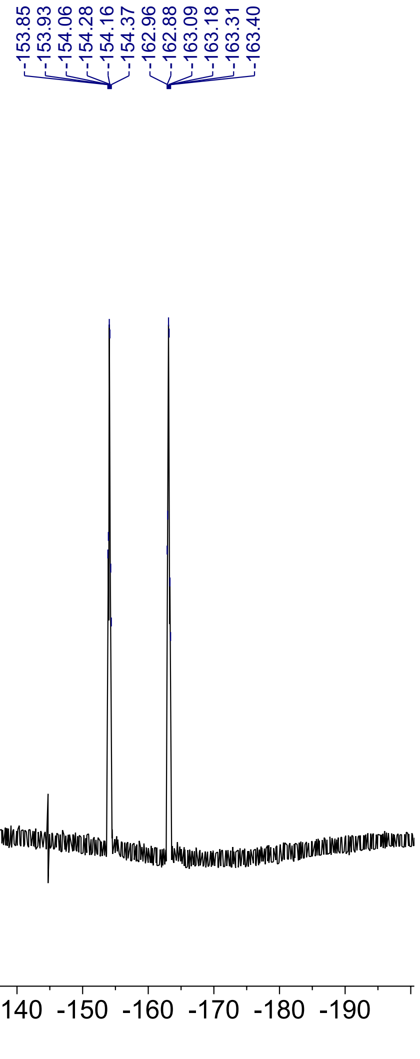

^{1}H NMR of *N*-(1-(difluoroboraneyl)ethyl)-*N*-ethyl-3,4,5,6-tetrahydropyridin-2-amineamine (4g) ($CDCl_3$, 500 MHz)

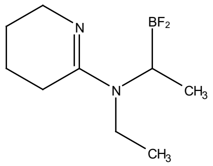

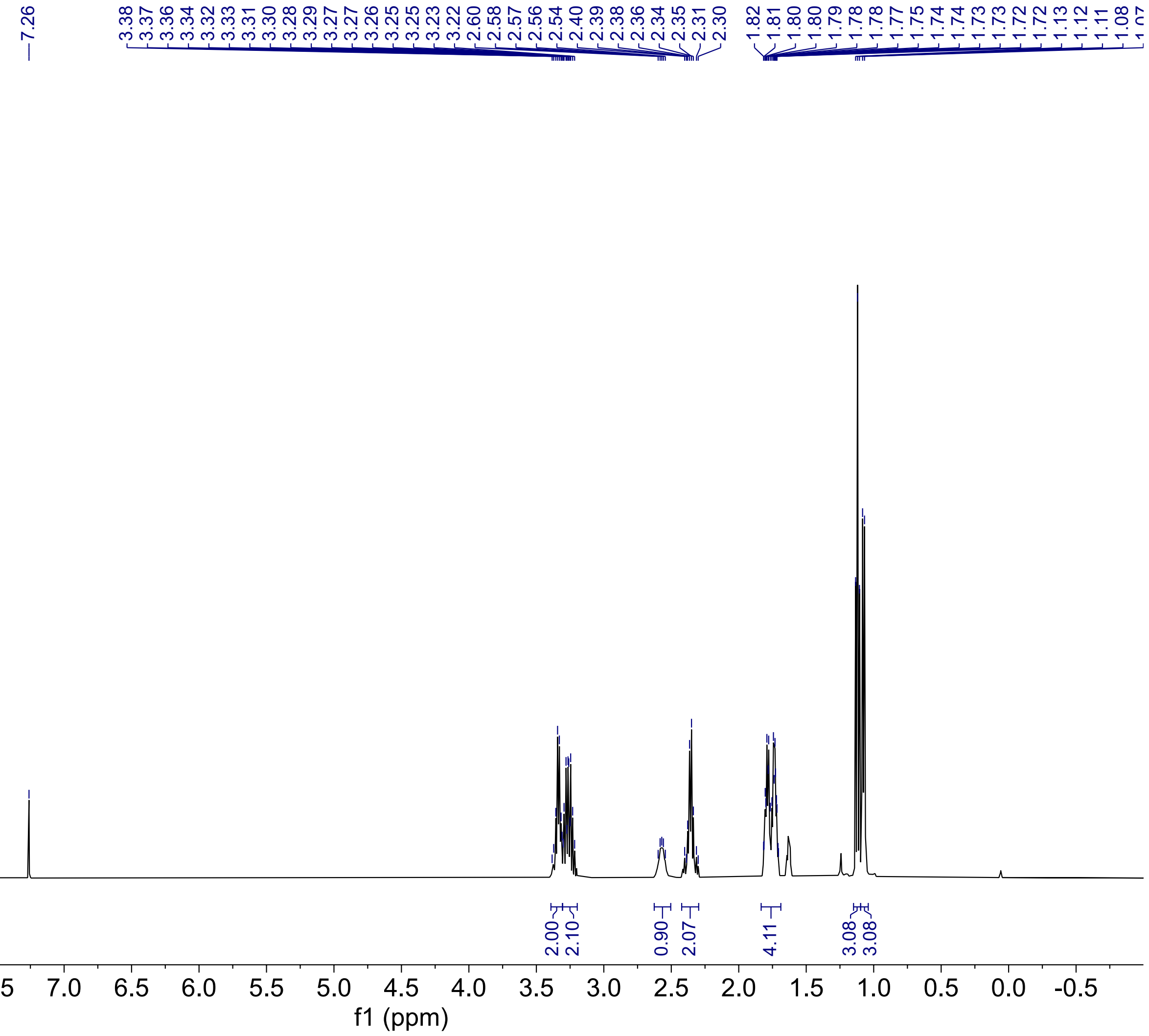

$^{13}C\{^1H\}$ NMR of *N*-(1-(difluoroboraneyl)ethyl)-*N*-ethyl-3,4,5,6-tetrahydropyridin-2-amineamine (4g) ($CDCl_3$, 126 MHz)

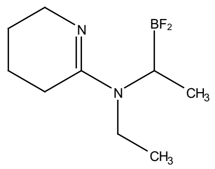

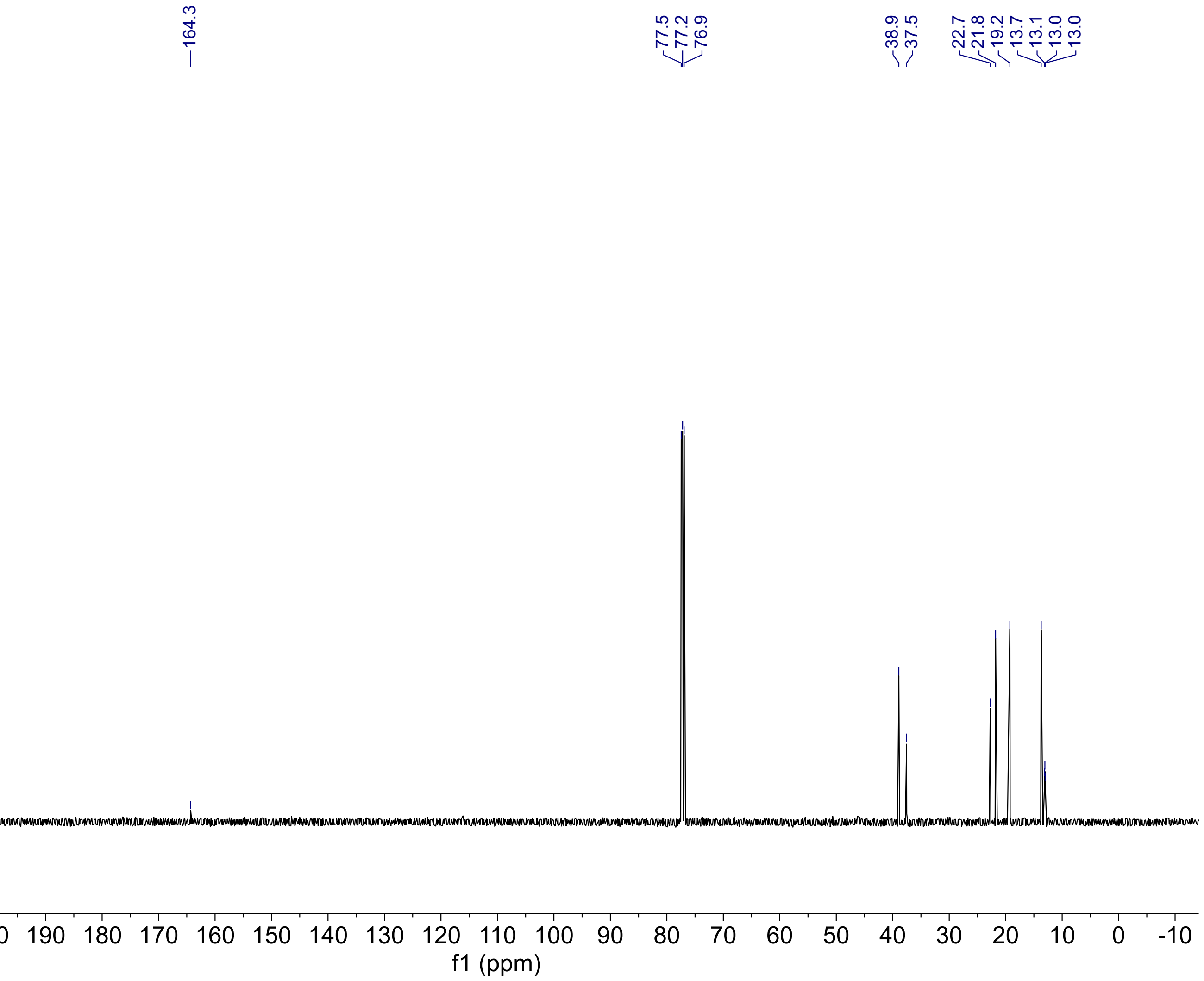

^{11}B NMR of *N*-(1-(difluoroboraneyl)ethyl)-*N*-ethyl-3,4,5,6-tetrahydropyridin-2-amineamine (4g) ($CDCl_3$, 160 MHz)

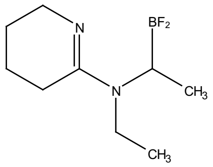

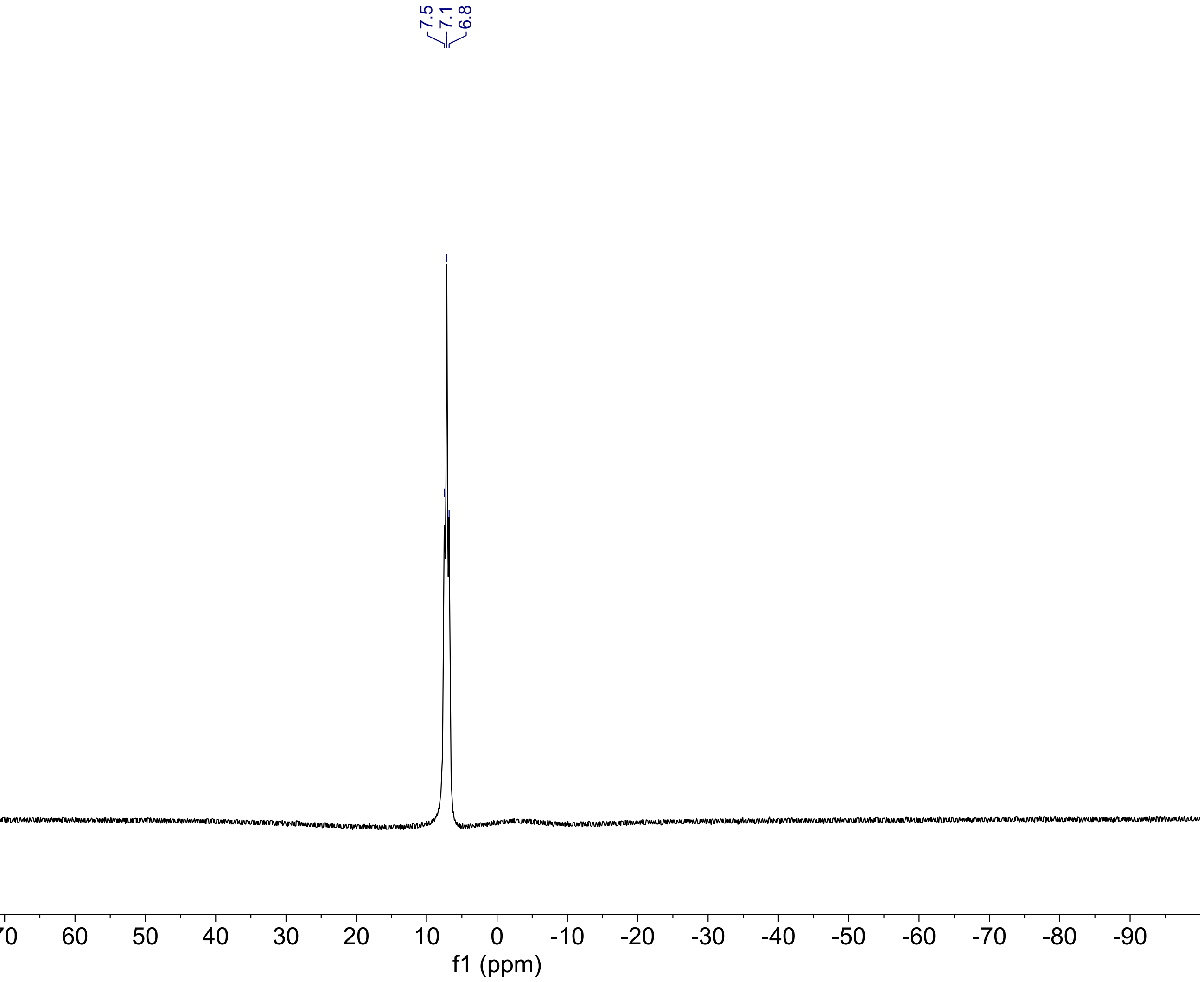

^{19}F NMR of *N*-(1-(difluoroboraneyl)ethyl)-*N*-ethyl-3,4,5,6-tetrahydropyridin-2-amineamine (4g) ($CDCl_3$, 470 MHz)

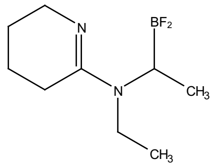

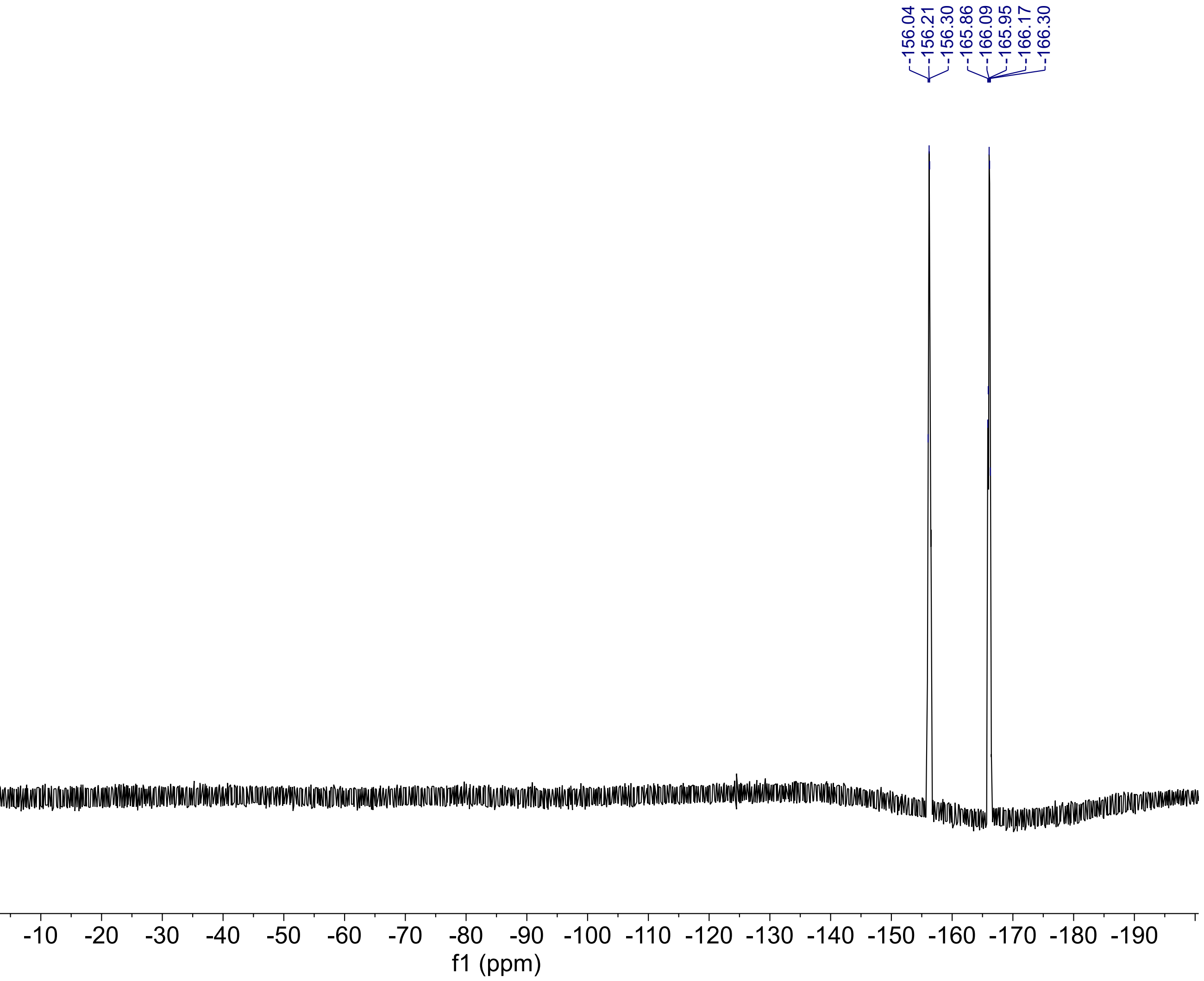

^{1}H NMR of *N*-(1-(difluoroboraneyl)ethyl)-*N*-ethyl-3,4,5,6-tetrahydro-2*H*-azepin-7-amine (4h) ($CDCl_3$, 500 MHz)

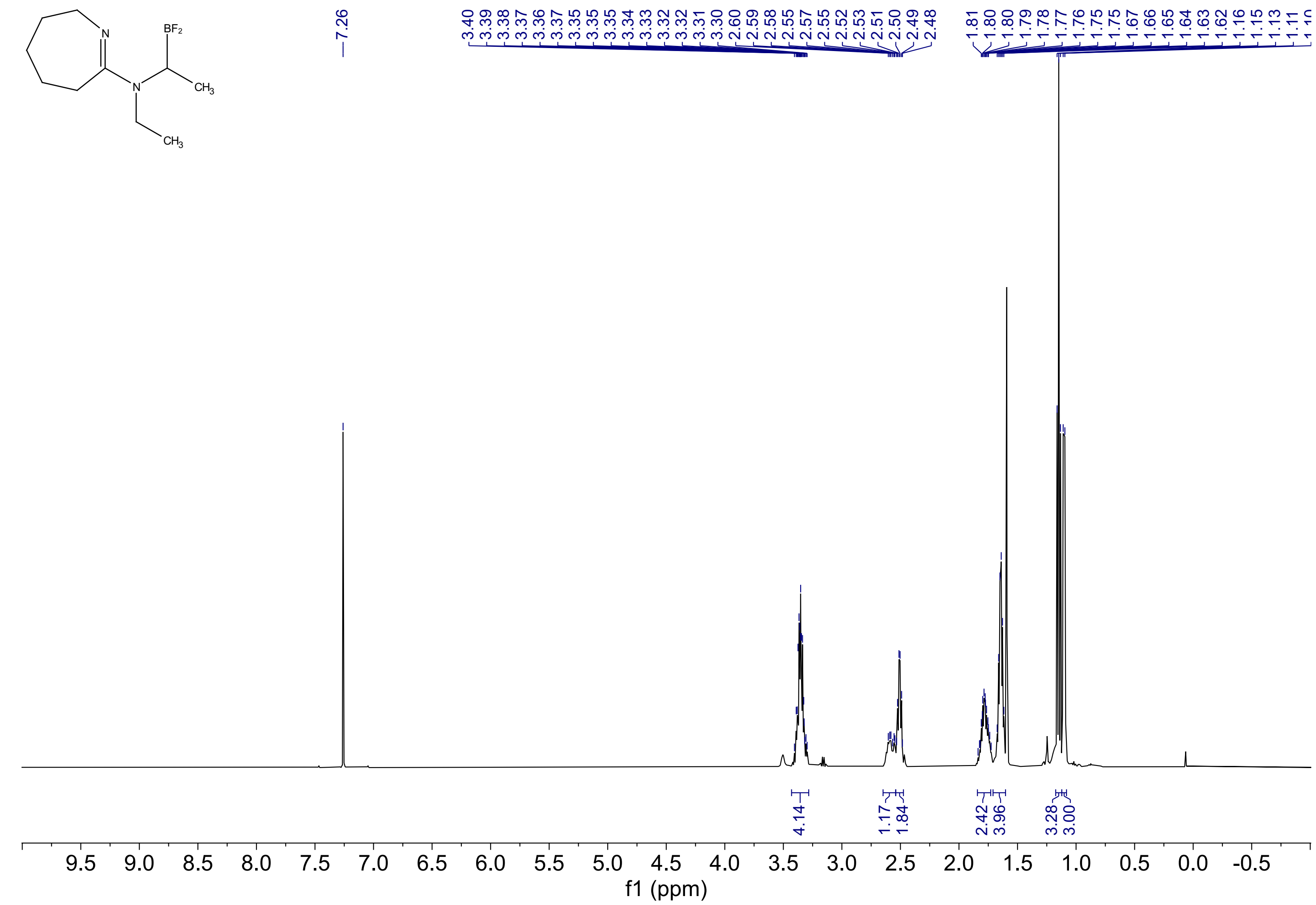

$^{13}C\{^{1}H\}$ NMR of *N*-(1-(difluoroboraneyl)ethyl)-*N*-ethyl-3,4,5,6-tetrahydro-2*H*-azepin-7-amine (4h) ($CDCl_3$, 126 MHz)

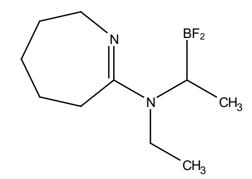

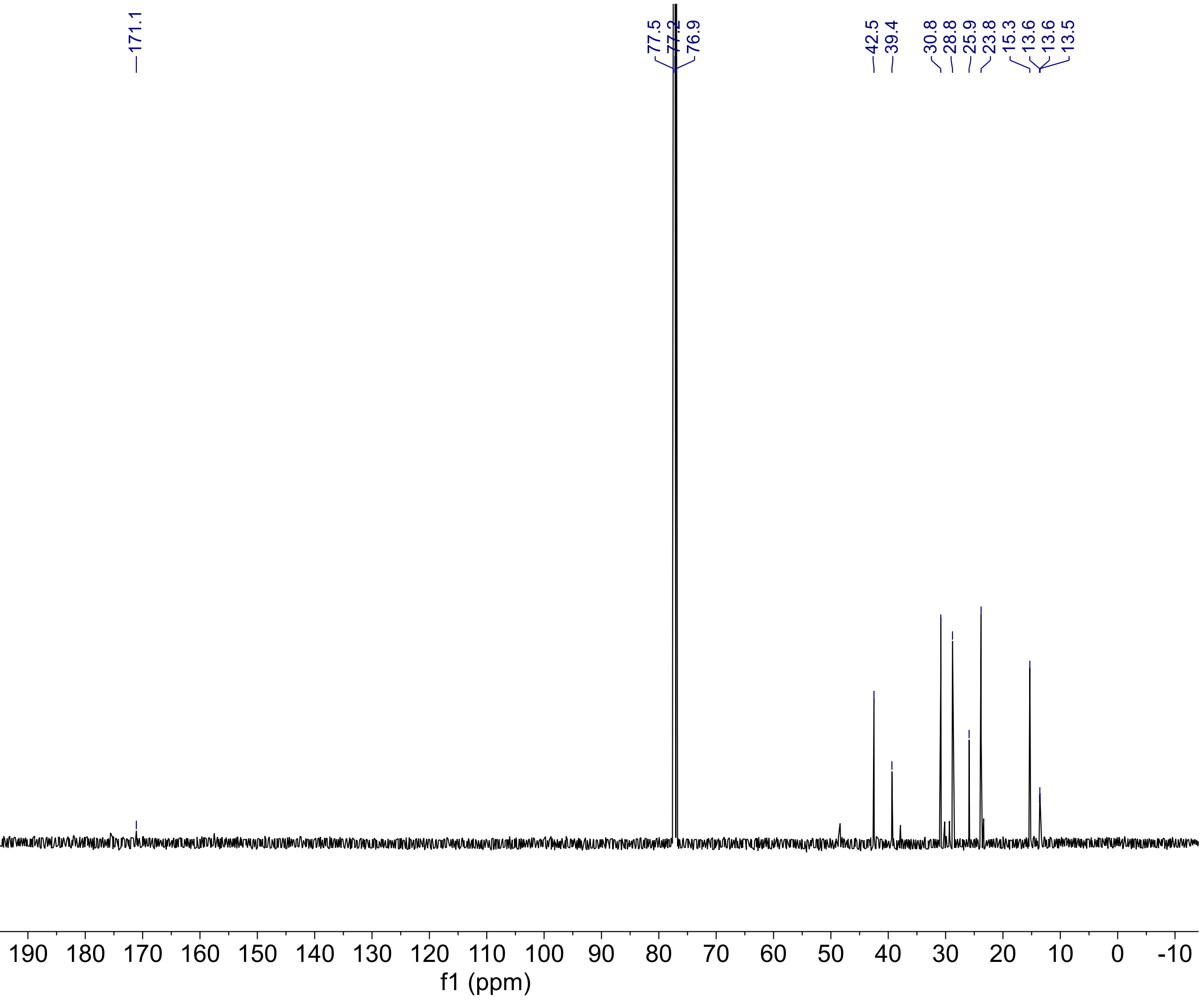

^{11}B NMR of *N*-(1-(difluoroboraneyl)ethyl)-*N*-ethyl-3,4,5,6-tetrahydro-2*H*-azepin-7-amine (4h) ($CDCl_3$, 160 MHz)

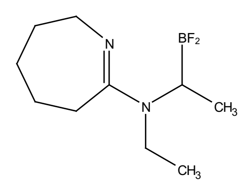

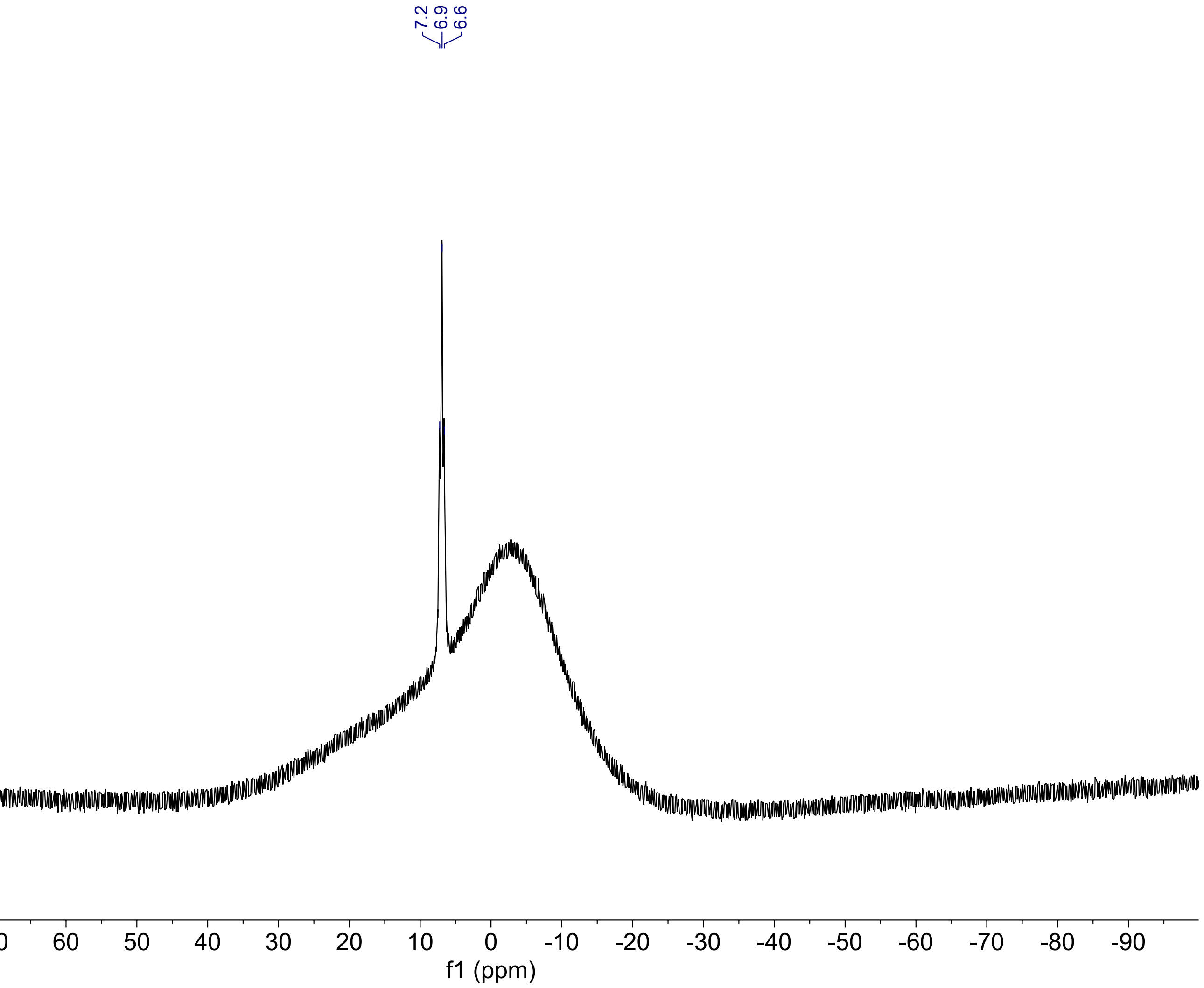

^{19}F NMR of *N*-(1-(difluoroboraneyl)ethyl)-*N*-ethyl-3,4,5,6-tetrahydro-2*H*-azepin-7-amine (4h) ($CDCl_3$, 470 MHz)

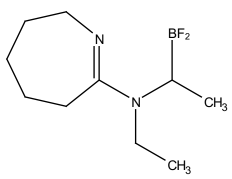

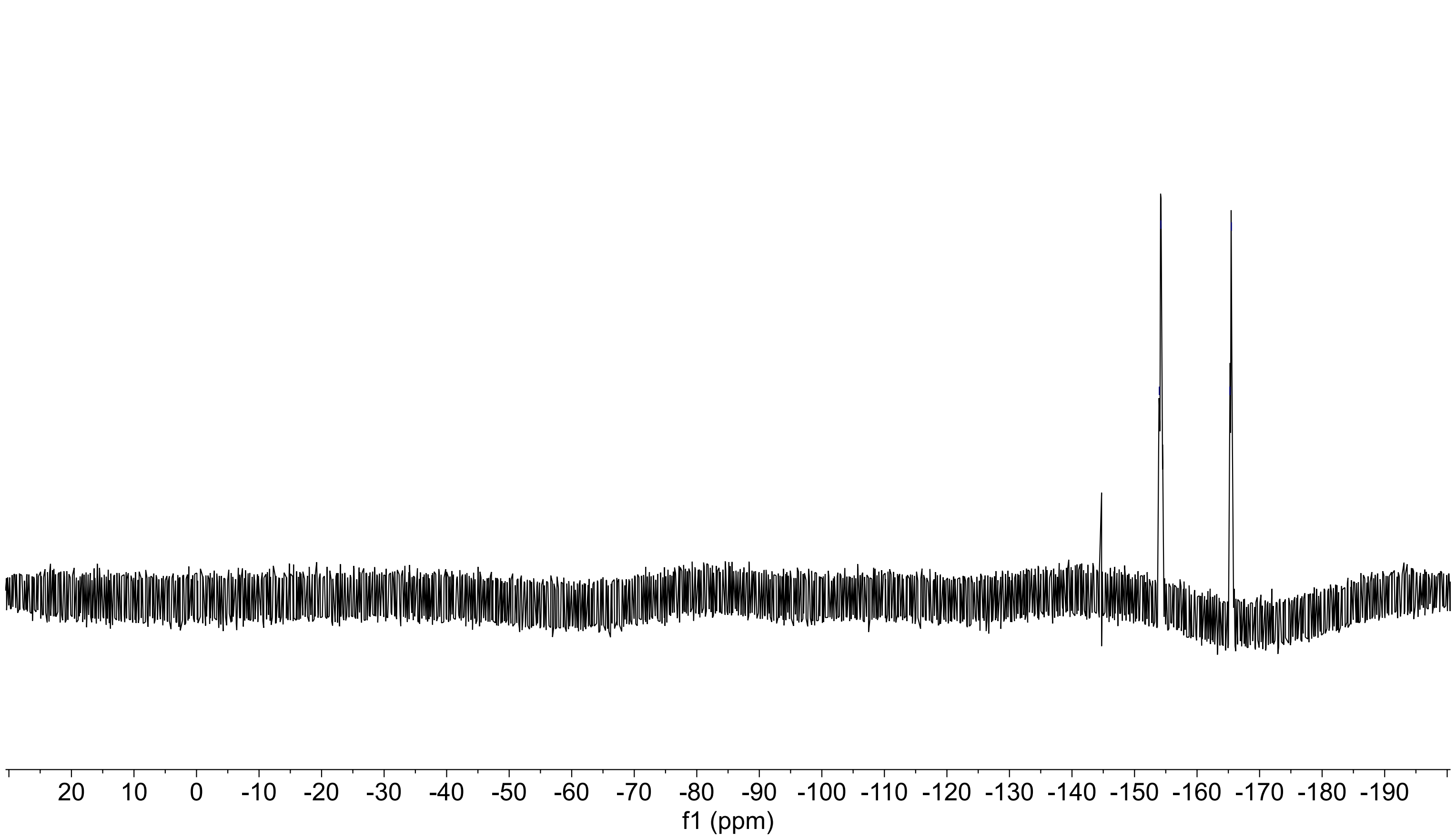

^{1}H NMR of (*E*)-1-(3-(difluoroboraneyl)morpholino)-*N*-ethylethan-1-imine (4i) ($CDCl_3$, 500 MHz)

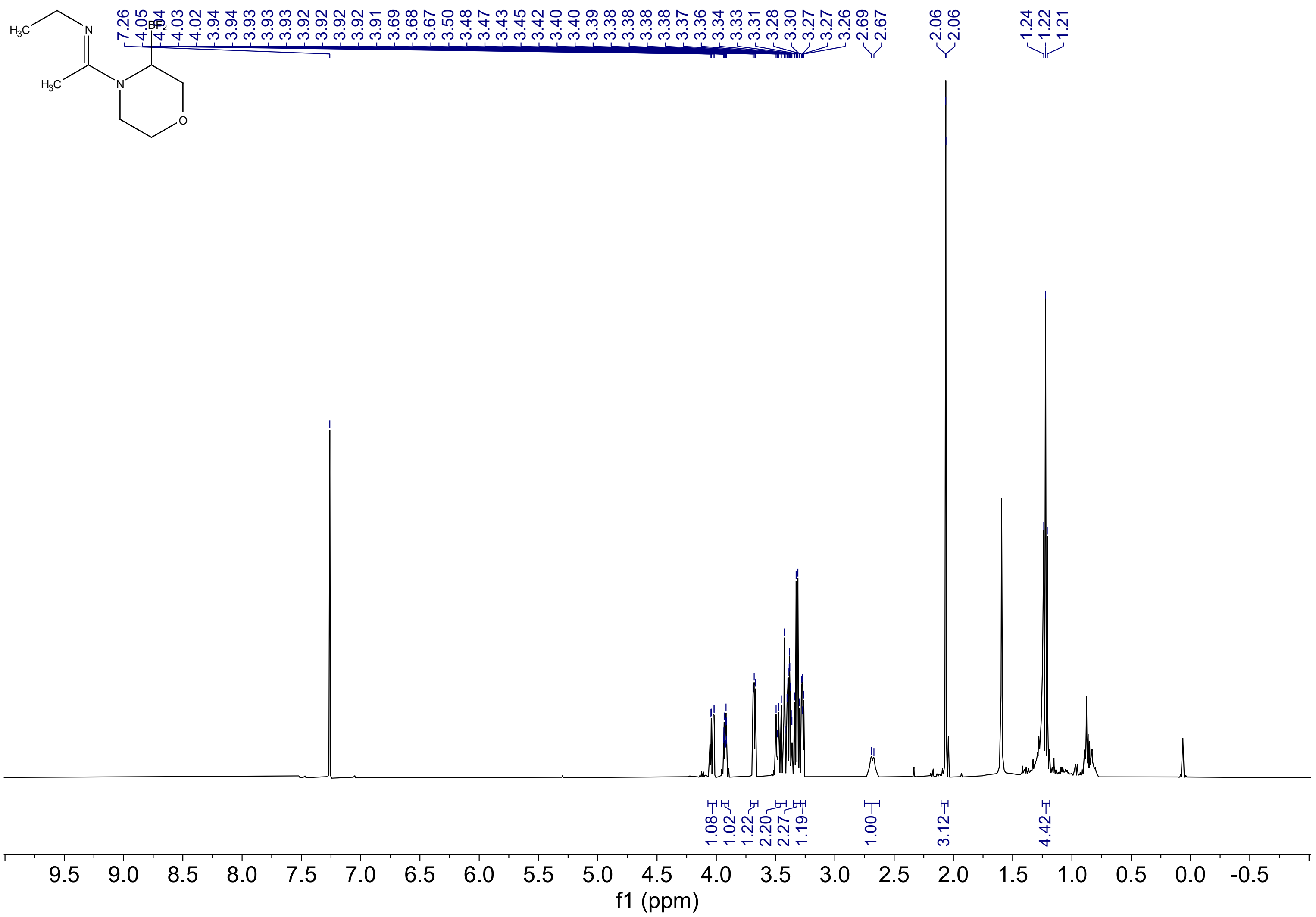

$^{13}C\{^{1}H\}$ NMR of (*E*)-1-(3-(difluoroboraneyl)morpholino)-*N*-ethylethan-1-imine (4i) ($CDCl_3$, 126 MHz)

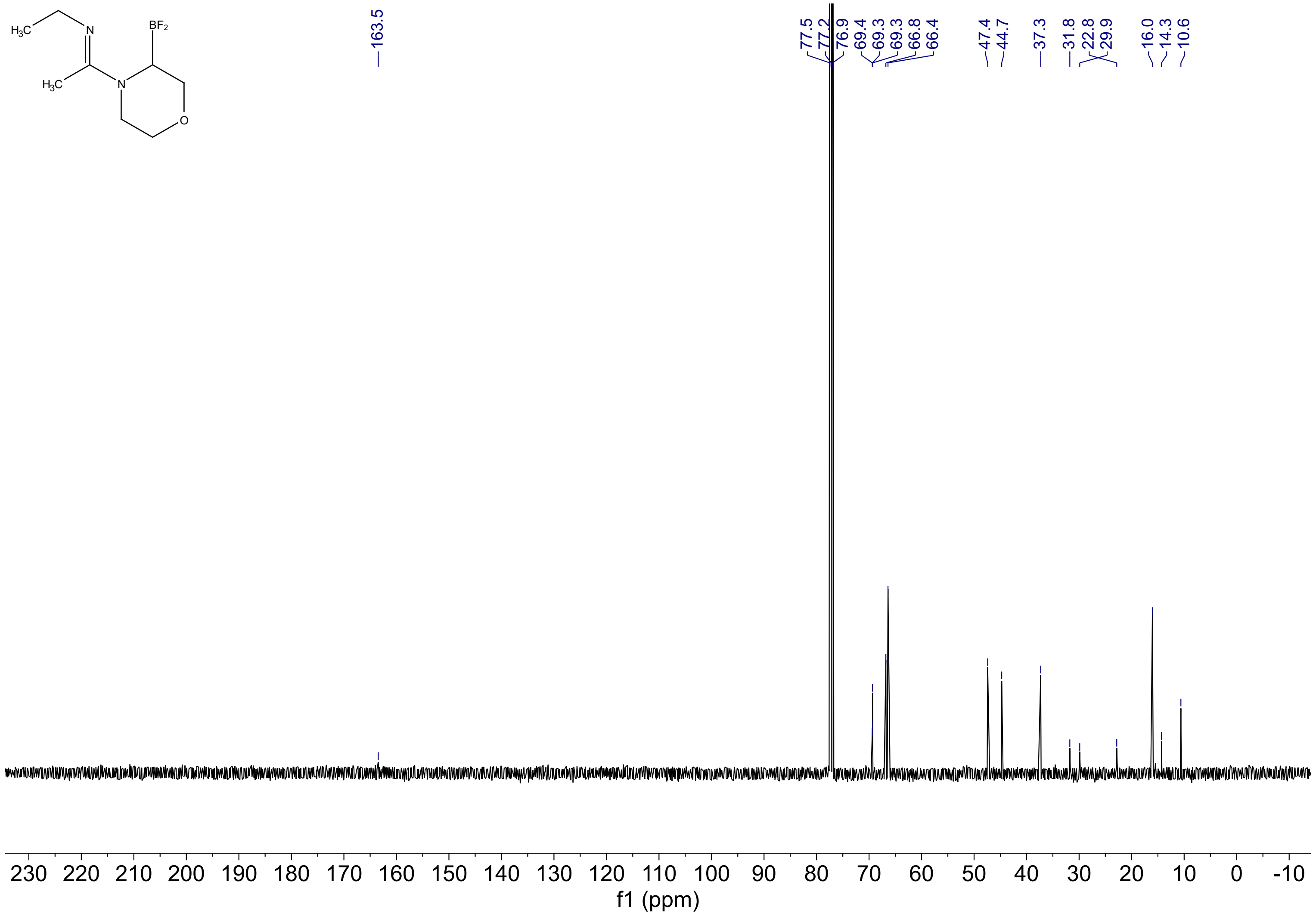

^{11}B NMR of (*E*)-1-(3-(difluoroboraneyl)morpholino)-*N*-ethylethan-1-imine (4i) ($CDCl_3$, 160 MHz)

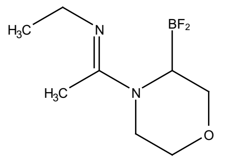

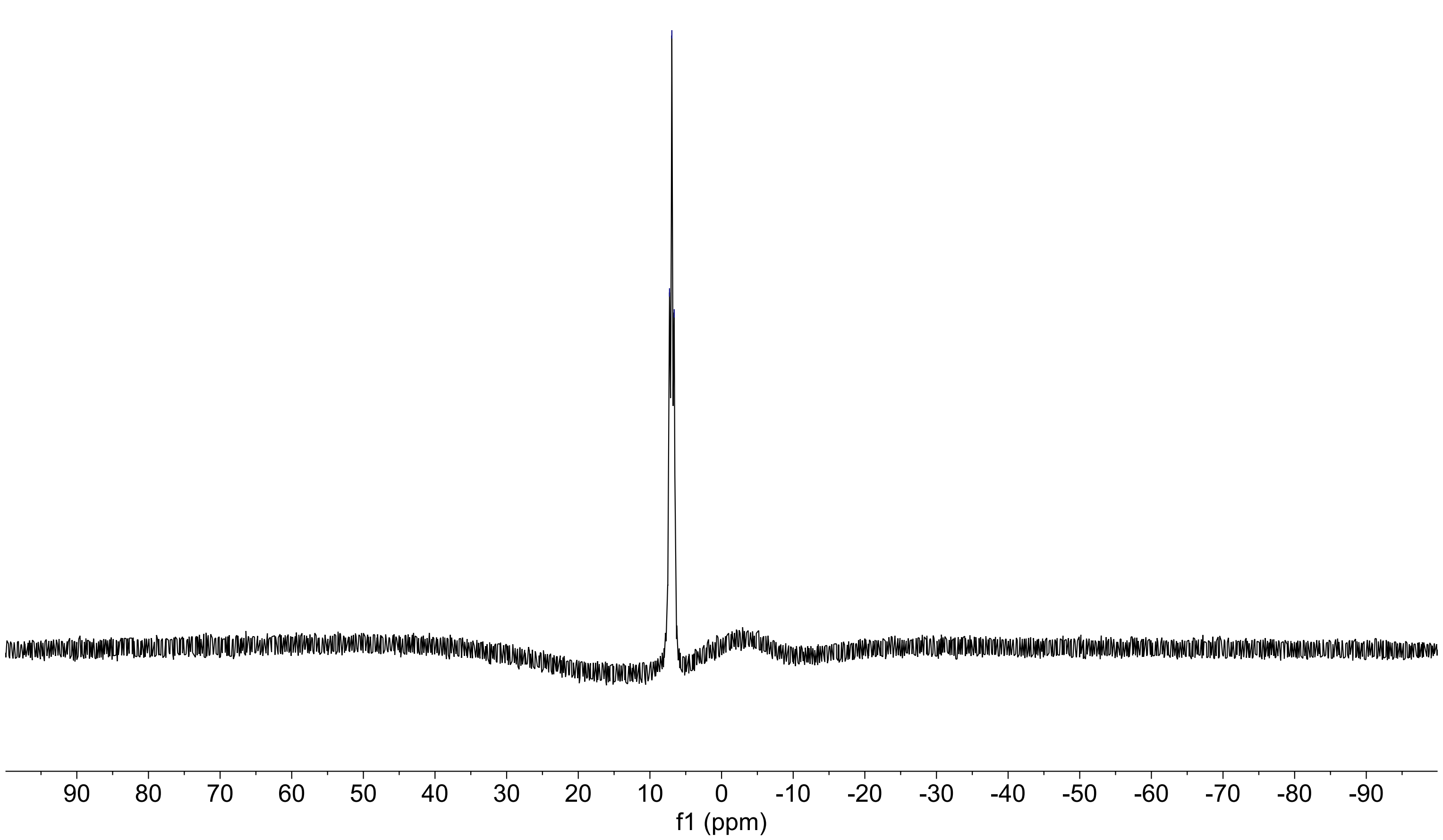

^{19}F NMR of (*E*)-1-(3-(difluoroboraneyl)morpholino)-*N*-ethylethan-1-imine (4i) ($CDCl_3$, 470 MHz)

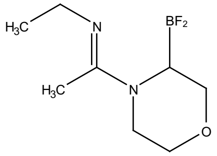

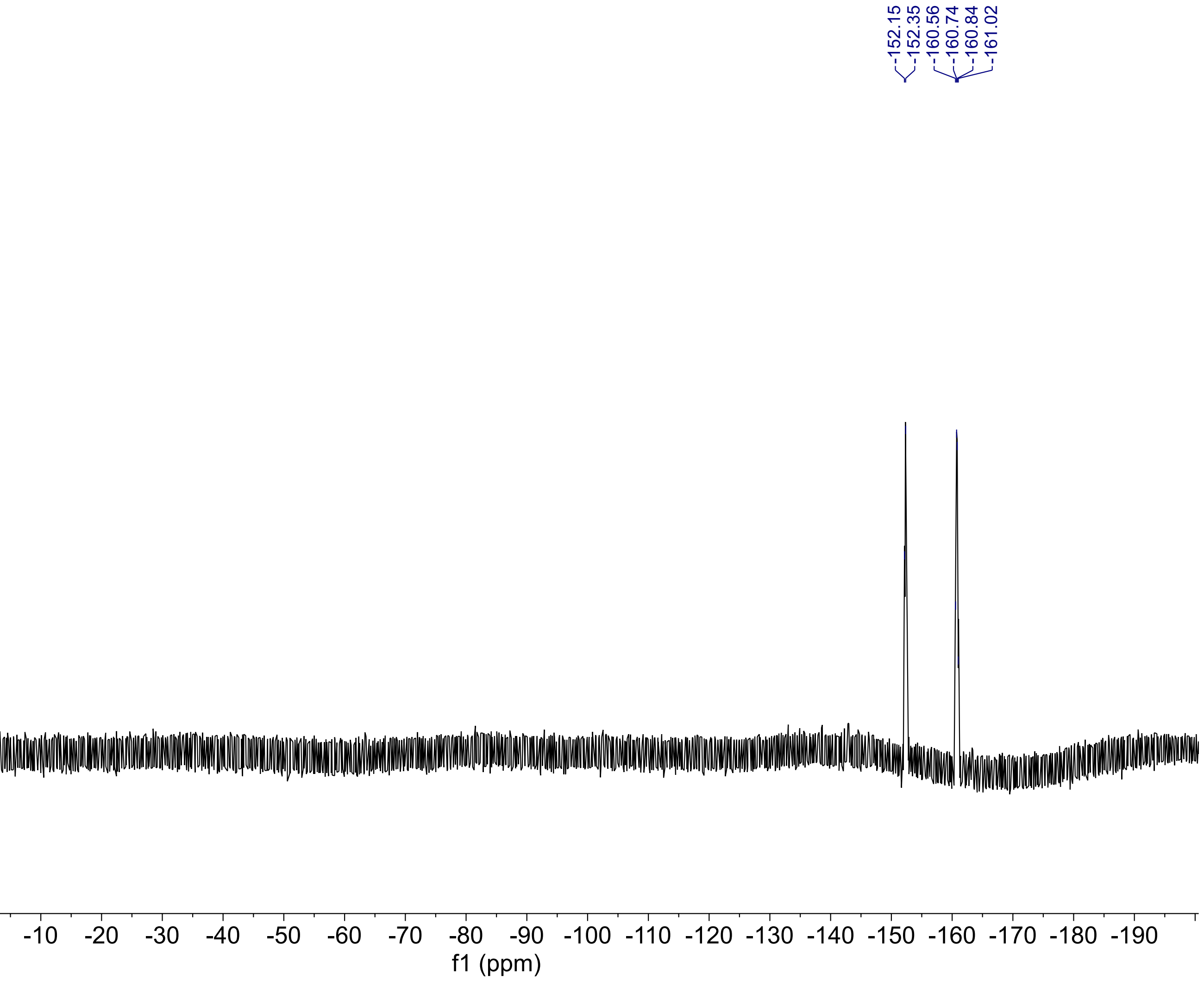

^1H NMR of (E)-N-((difluoroboraneyl)methyl)-N,N'-dimethylacetimidamide (4j) ($CDCl_3$, 500 MHz)

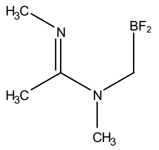

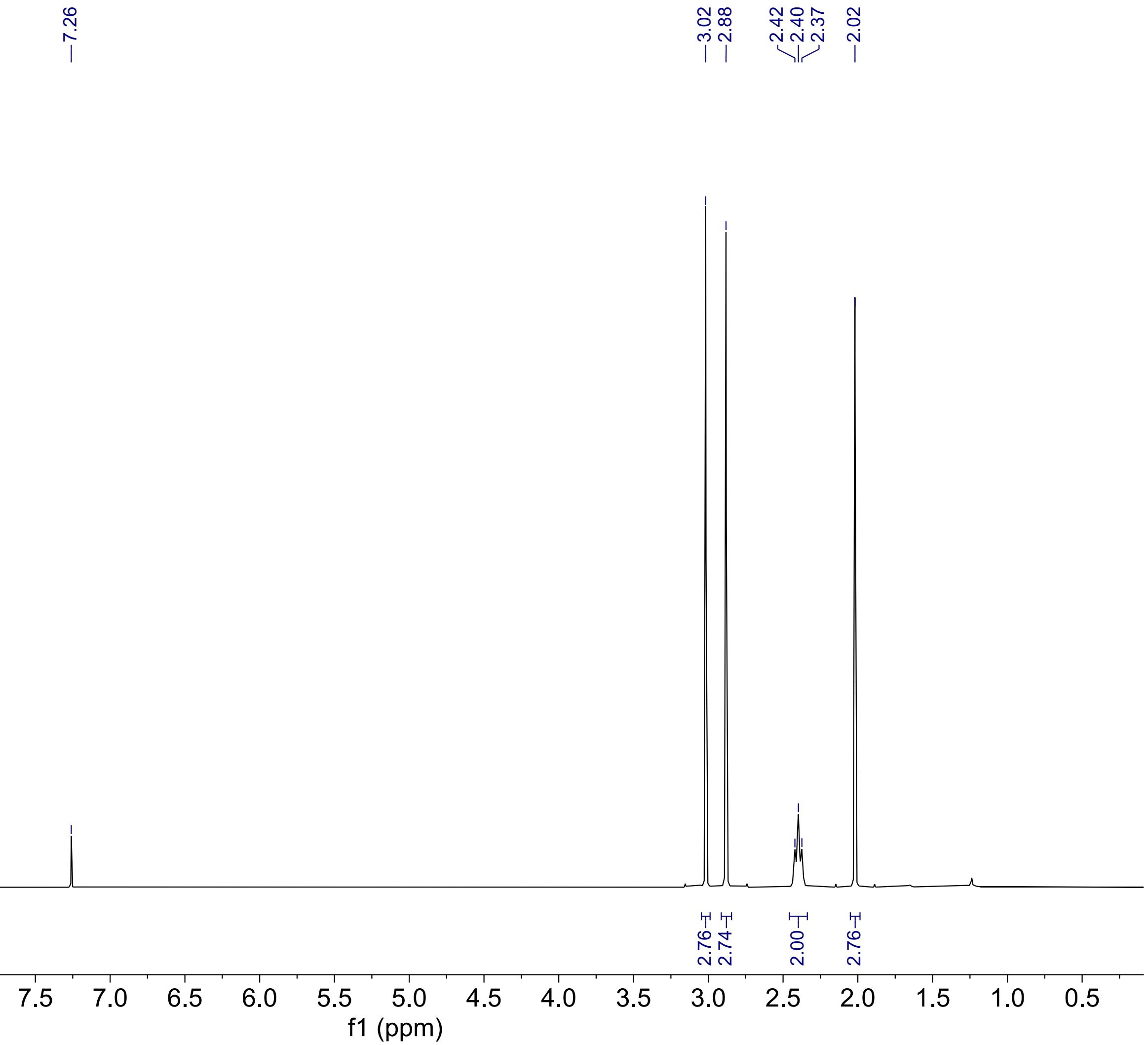

$^{13}C\{^{1}H\}$ NMR of (E)-N-((difluoroboraneyl)methyl)-N,N'-dimethylacetimidamide (4j) ($CDCl_3$, 126 MHz)

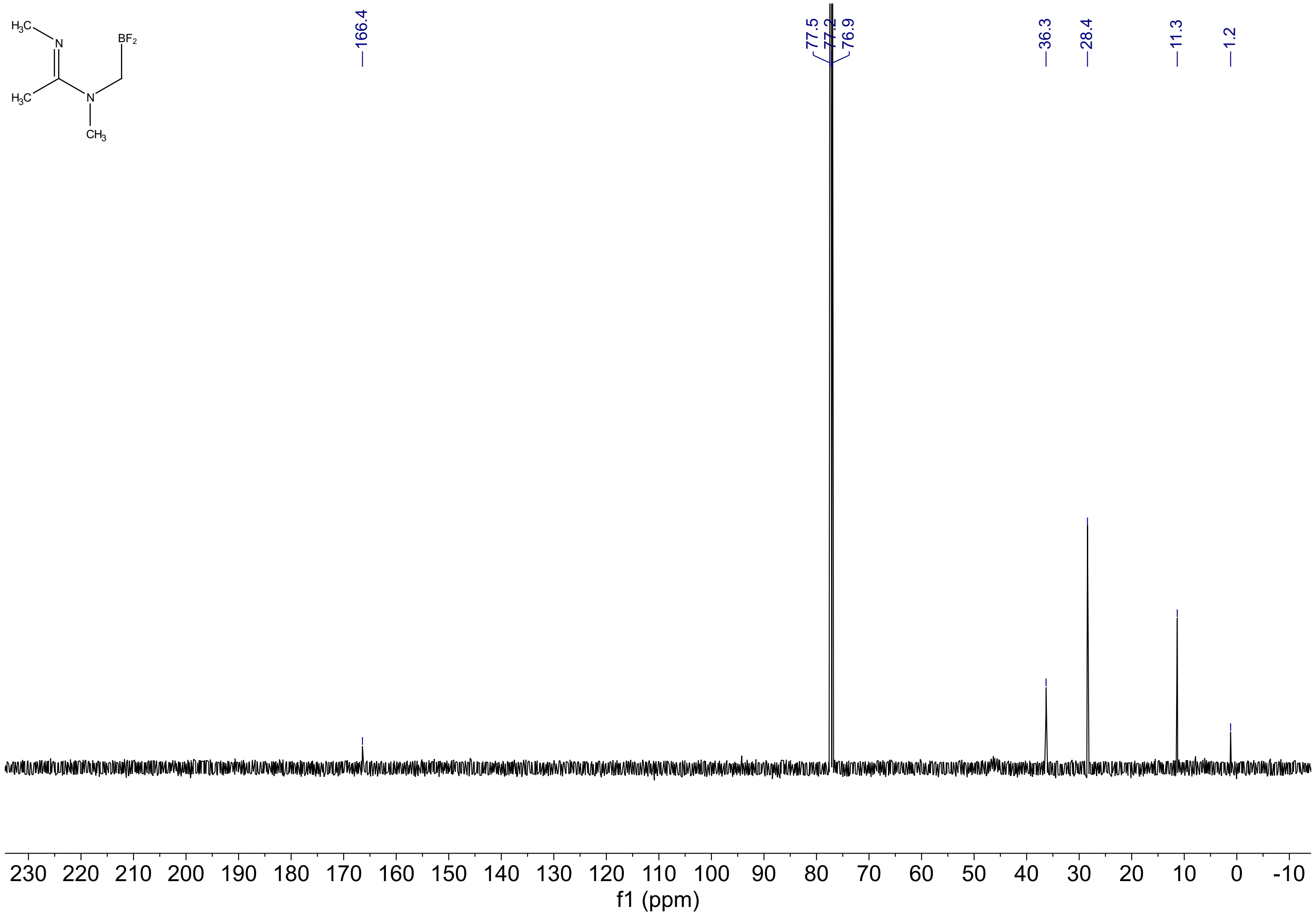

^{11}B NMR of (E)-N-((difluoroboraneyl)methyl)-N,N'-dimethylacetimidamide (4j) ($CDCl_3$, 160 MHz)

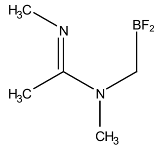

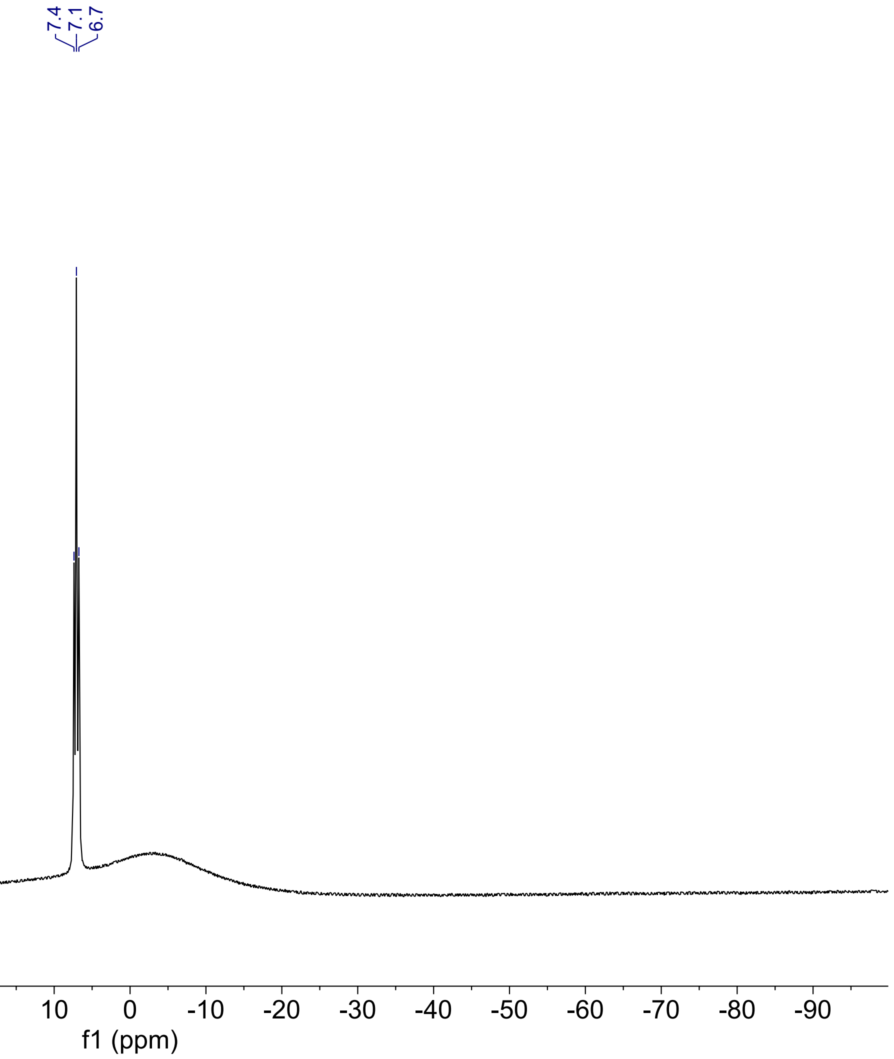

^{19}F NMR of (E)-N-((difluoroboraneyl)methyl)-N,N'-dimethylacetimidamide (4j) ($CDCl_3$, 470 MHz)

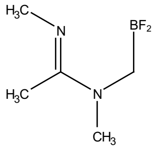

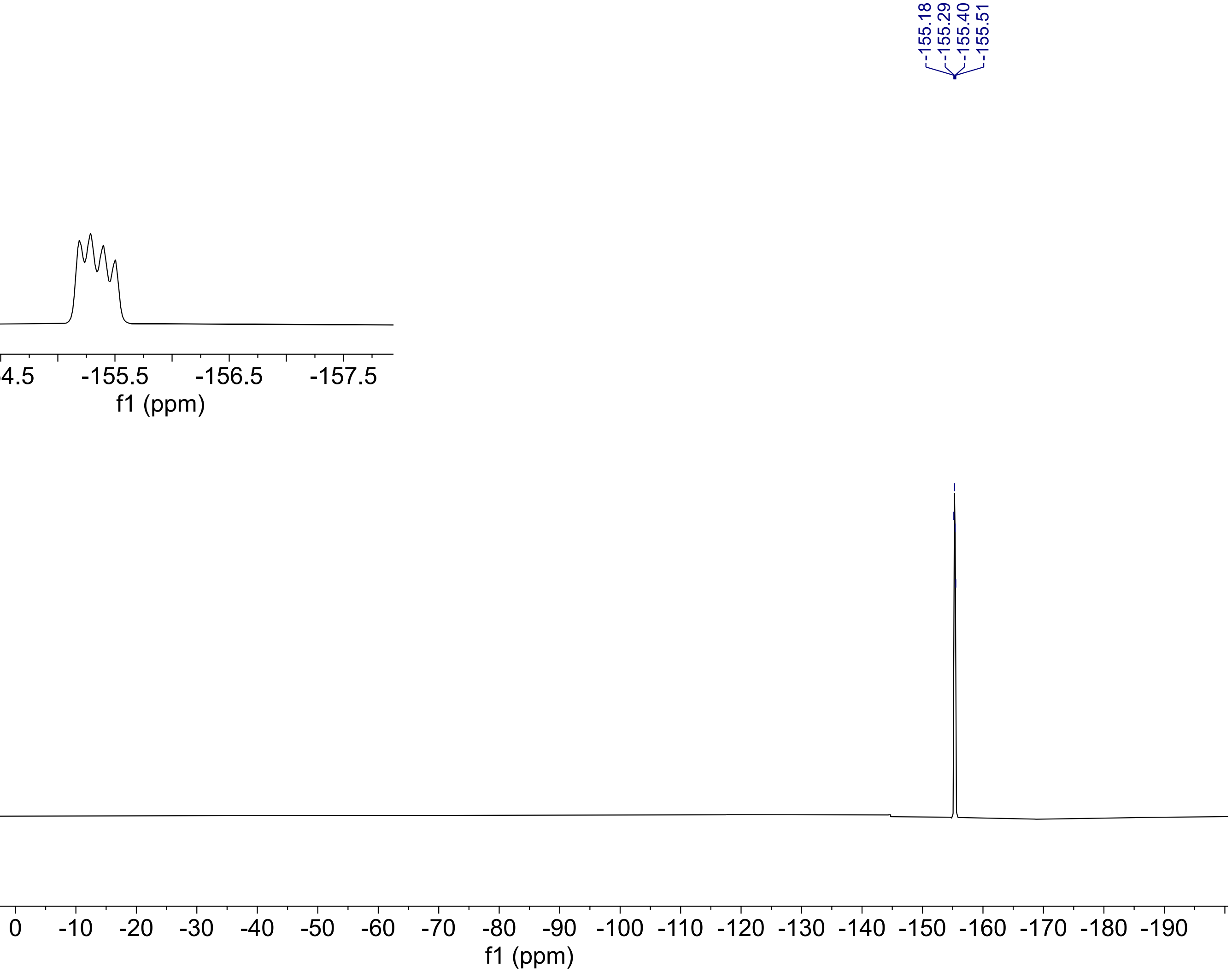

Supplement: Supplementary file 1 [file ol6c02012_si_001.pdf]
